# Mechanism of Herb Pairs *Astragalus mongholicus* and *Curcuma phaeocaulis* Valeton in Treating Gastric Carcinoma: A Network Pharmacology Combines with Differential Analysis and Molecular Docking

**DOI:** 10.1155/2022/8361431

**Published:** 2022-03-14

**Authors:** Zixuan Wu, Xiyang Pan, Chaosheng Deng, Minjie Cai, Kai Yuan, Peidong Huang, Guoqi Shi

**Affiliations:** ^1^Guangzhou University of Chinese Medicine, Guangzhou, Guangdong 510006, China; ^2^Shantou Health School, Shantou, Guangdong 515061, China; ^3^Yunnan University of Chinese Medicine, Kunming, Yunnan 650500, China

## Abstract

**Background:**

Gastric carcinoma (GC) is a kind of digestive tract tumor that is highly malignant and has a very poor prognosis. Although both *Astragalus mongholicus* (AM, *huáng qí*) and *Curcuma phaeocaulis* Valeton (CPV, *é zhú*) can slow the onset and progression of GC, the mechanism by which AM-CPV works in the treatment of GC is uncertain.

**Materials and Methods:**

The traditional Chinese medicine network databases TCMSP, TCMID, and ETCM were used to identify the key functional components and associated targets of AM and CPV. To establish a theoretical foundation, the development of gastric cancer (GC) was predicted utilizing a GEO gene chip and TCGA difference analysis mixed with network pharmacology. A herbal-ingredient-target network and a core target-signal pathway network were created using GO and KEGG enrichment analyses. The molecular docking method was used to evaluate seventeen main targets and their compounds.

**Results:**

Cell activity, reactive oxygen species modification, metabolic regulation, and systemic immune activation may all be involved in the action mechanism of the AM-CPV drug-pair in the treatment of GC. It inhibits the calcium signaling route, the AGE-RAGE signaling system, the cAMP signaling pathway, the PI3K-Akt signaling network, and the MAPK signaling pathway, slowing the progression of GC. The number of inflammatory substances in the tumor microenvironment is reduced, GC cell proliferation is deprived, apoptosis is promoted, and GC progression is retarded through controlling the IL-17 signaling route, TNF signaling pathway, and other inflammation-related pathways.

**Conclusions:**

The AM-CPV pharmaceutical combination regulates GC treatment via a multitarget, component, and signal pathway with a cooperative and bidirectional regulatory mechanism. Its active constituents may treat GC by regulating the expression of STAT1, MMP9, IL6, HSP90AA1, JUN, CCL2, IFNG, CXCL8, and other targets, as well as activating or inhibiting immune-inflammatory and cancer signaling pathways.

## 1. Introduction

Gastric carcinoma (GC) is one of the five most common cancers in the world. At present, about 1.03 million patients suffer from GC [1]. How to prevent and treat effectively GC has become an urgent problem. Many scholars had proposed that GC may be attributed to autoimmunity, other bacteria, and their metabolites (such as N-nitroso compounds or acetaldehyde) [2]. Cellular and humoral immune cells interact to form an intricate tumor immune microenvironment, which affects the progression and survival of GC [3]. Modern medicine in the treatment of GC, if the conditions permit, whether in the early or late stage, should follow the treatment principle of early detection and early operation [4]. For patients who cannot operate or after operation, chemotherapy plays a crucial part in the treatment. However, chemotherapeutic drugs are cytotoxic drugs with great side effects. Continuous application could have various adverse reactions to patients. Repeated use can easily lead to drug resistance reducing the curative effect [5]. In recent years, with the progress and development of science and technology, the unique curative effect advantage of traditional Chinese medicine (TCM) has caught more and more attention [6]. TCM can effectively inhibit the proliferation of cancer and prevent the spread and metastasis of tumor through synergistic effect on numerous signal pathways such as PI3K/AKT, which has a broad application prospect in the treatment of GC [7].

It is because of the multitarget, multipathway, and multilink feature of TCM in the treatment of GC that makes it a major obstacle to the exploration of its therapeutic mechanism [8]. How to discover the effective constituents of components in the anticancer process has become the research focus. Numerous studies had investigated the mechanisms of TCM in GC treatment through the discovery in the active ingredient of TCM which could inhibit cell proliferation and induce cancer cell apoptosis, inhibit cell invasion and migration, and regulate cell signal pathways. The fundamental etiology and pathophysiology of GC, according to TCM, are a lack of vial QI and toxin, as well as blood stasis. As a result, we should be focused on increasing vial QI and removing blood stasis. AM can aid critical QI, while CPV can assist in dissolving blood clots, although research has discovered that AM-CPV is a frequent core drug pair for the treatment of precancerous lesions of gastric cancer (PLGC) [9].

**Figure 1:**
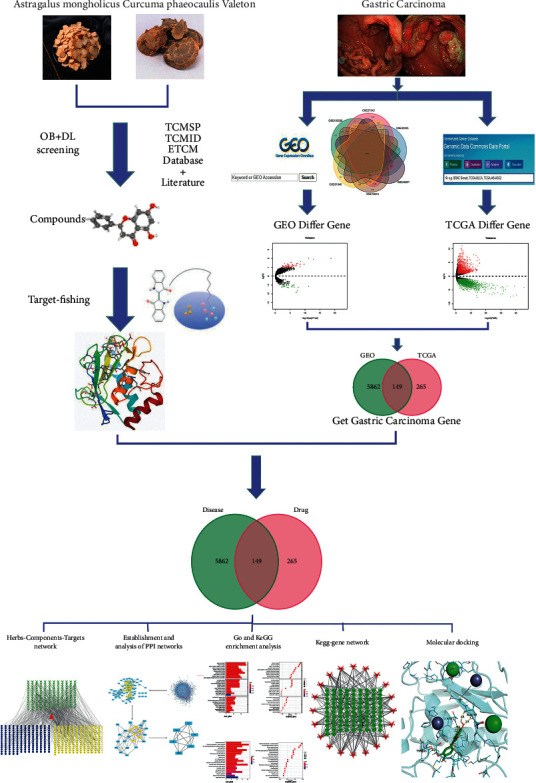
Framework based on an integration strategy of network pharmacology.

The concept of traditional Chinese medicine has developed and inherits for thousands of years, having formed its unique theoretical basis and characteristics of herb use. In terms of compatibility, two or more herbs are often selected for synergism and toxicity reduction [10]. However, there is poorly clinical research on the antitumor effect and mechanism from the view of the compatibility of two. It is based on this point of the research applying the new network pharmacology technique to investigating the interaction between drug and target through searching the database of genes, proteins, diseases, and drugs and the real experimental data, constructing the relationship network between “drug-gene-target-disease.” It is expected that the balance of biological network can be reconstructed by drugs in order to explore the effect of AM-CPV on the treatment of GC. In addition, the integrity and systematic characteristics of the network pharmacology research strategy of traditional Chinese medicine are in conformity with the principles of diagnosis and treatment in disease and feature of the synergistic effect of multicomponents, multiapproaches, and multitargets in traditional Chinese medicine and its prescriptions. Therefore, it is extremely important for providing reference ideas for basic research and clinical application in the future to study the mechanism of AM-CPV in the treatment of GC by using the method of network pharmacology combined with GEO and TCGA difference analysis. An outline of the method is shown in Figure 1.

## 2. Materials and Methods

### 2.1. Construction of a Database of Main Active Ingredients

The TCM system pharmacology database and analysis platform (TCMSP) was used to identify the active ingredients of AM and CPV. The main active ingredients were then selected according to the optimal toxicologic ADME rules reported in the literature (OB ≥ 30%; DL ≥ 0.18). If the compounds did not meet the screening criteria or does not exist in the TCMSP database, it would be supplemented from the TCMID and the active components of AM-CPV drug-pair reported in the related literature were included in [11, 12]. The related compounds were input into PubChem and PharmGKB Database to obtain the molecular structure of the compounds.

### 2.2. Potential Targets of AM and CPV

The active components of a drug performance-related biological functions through the relevant targets. In addition to obtaining the targets of the core functional ingredients of AM and CPV directly from the TCMSP database, the core active components reported in TCMID database and related literature were identified by using the information of small molecular structure. Swiss Target Prediction and STITCH identified a large number of possible targets for gastric carcinoma. According to Uniprot, the names of the collected target about AM and CPV drug-pairs were standardized by Perl.

### 2.3. Construction of a Gastric Carcinoma-Related Targets Database

Micro array data of expression of mRNAs between the GC and normal samples were screened from the GEO database. Series: GSE27342, GSE33335, GSE56807, GSE79973, GSE81948, and GSE103236. Sva and Limma of R4.1.0 were utilized to carry out joint analysis of multiple chips and correct data batches (batchNormalize). The Venn map was drawn by Venn of Rmur4.1.0. Genes with an adjusted *P* < 0.05 and |log2FC| ≥ 1 were examined significantly differentially expressed and GC-related targets. In addition, GC-related disease targets were extracted in the TCGA database, combined with the TCGA and GEO different analysis results to eliminate repeated disease targets and establish the disease target database of GC. Then, it was checked by the intersection of different genes in the two databases.

### 2.4. Construction of the PPI Interaction Network

The core active ingredient target of drug-pairs of AM and CPV was matched with the disease target of GC to obtain the compound target. The Venn map was drawn by the Venn of R4.1.0. In order to study the functional relationship of DEGs, the PPI network of the target was obtained by using the String, with protein type “homosapiens” and the highest confidence level (0.700) as the setting standard of the drug-disease intersection gene.

### 2.5. Construction of “Herbs-Components-Targets” Network of AM-CPV

The “herbs-components-targets” network (H-C-T network) of AM and CPV in the treatment of GC was constructed by using Cytoscape3.7.2. According to the topological characteristics of the network, three most important parameters were selected by CytoNCA to screen the core targets of AM-LA drug-pairs in the treatment of GC: degree centrality (DC), closeness centrality (CC), and betweenness centrality (BC). They indicated the function and impact of the associated nodes in the overall network, and their relevance was positively connected with the network's output value. According to relevant literature, the target with the median value for DC was chosen [13], and the target with the median value for BC and CC was picked to achieve more accurate core targets [14].

### 2.6. GO and KEGG Enrichment Analysis

The biological pathways associated with the DEGs were then examined using gene ontology (GO). Biological processes (BP), molecular functions (MF), and cellular components (CC) are controlled by differentially expressed PRGs. PRGs were further investigated using *R* based on KEGG data.

### 2.7. Active Components-Targets Docking

The protein configurations of the core targets were obtained from the Uniprot by using the minimum resolution (Resolution) and the source (Method), and the crystal structure of these protein configurations was obtained from the RCSB PDB database. 2D structures of 6 active components of core targets were available from PubChem database, and these 2D structures were minimized by chem3d software. The binding strength and activity of active components and targets were evaluated by SYBYL2.0 software, and the functional components of binding TotalScore greater than 3 were selected for subdocking. Then, crystal was imported into the Pymol 2.4 for dehydration, hydrogenation, and separation of ligands; it then imported AutoDockTools 1.5.6 to construct the docking grid box for each target. Docking was completed by Vina 1.1.2, and the molecules with the lowest binding energy in the docking conformation were selected to observe the binding effect by matching with the original ligands and intermolecular interactions (such as hydrophobicity, cation-*π*, anion-*π*, *π*-*π* stacking, and hydrogen bonding). Finally, the Pymol 2.4 software was used to visualize the molecular docking.

## 3. Results

### 3.1. Targets Prediction and Analysis of AM and CPV

428 core active components of AM and 24 CPV (Supplementary Table 2) were obtained from TCMSP. 24 AM and 9 CPV ingredients were obtained from TCMID. 2 CPV ingredients were obtained from relating literature. Through SwissTargetPrediction and STITCH database, 166 active components of AM and 81 active components of CPV (Supplementary Table 3) were obtained. 30 active components of AM and 38 active components of CPV (Supplementary Table 4) were obtained from ETCM. Although the active components obtained from TCMID and ETCM databases and related literature are not clear about OB and DL, they are still included. Finally, fifteen core active components (Table 1 or Supplementary Table 5) were included [15]. All in all, 1534 targets were identified by target fishing and by integrating the data obtained (Supplementary Tables 2–4).

### 3.2. Differentially Expressed Gene Search, Identification, and Analysis

**Figure 2:**
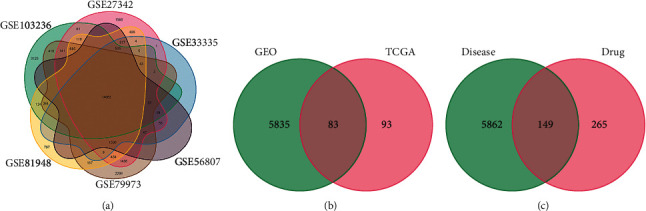
Venn diagram. (a) Six GEO chips. (b) Differential genes of GEO with TCGA. (c) The targets in GC and AM-CPV.

**Figure 3:**
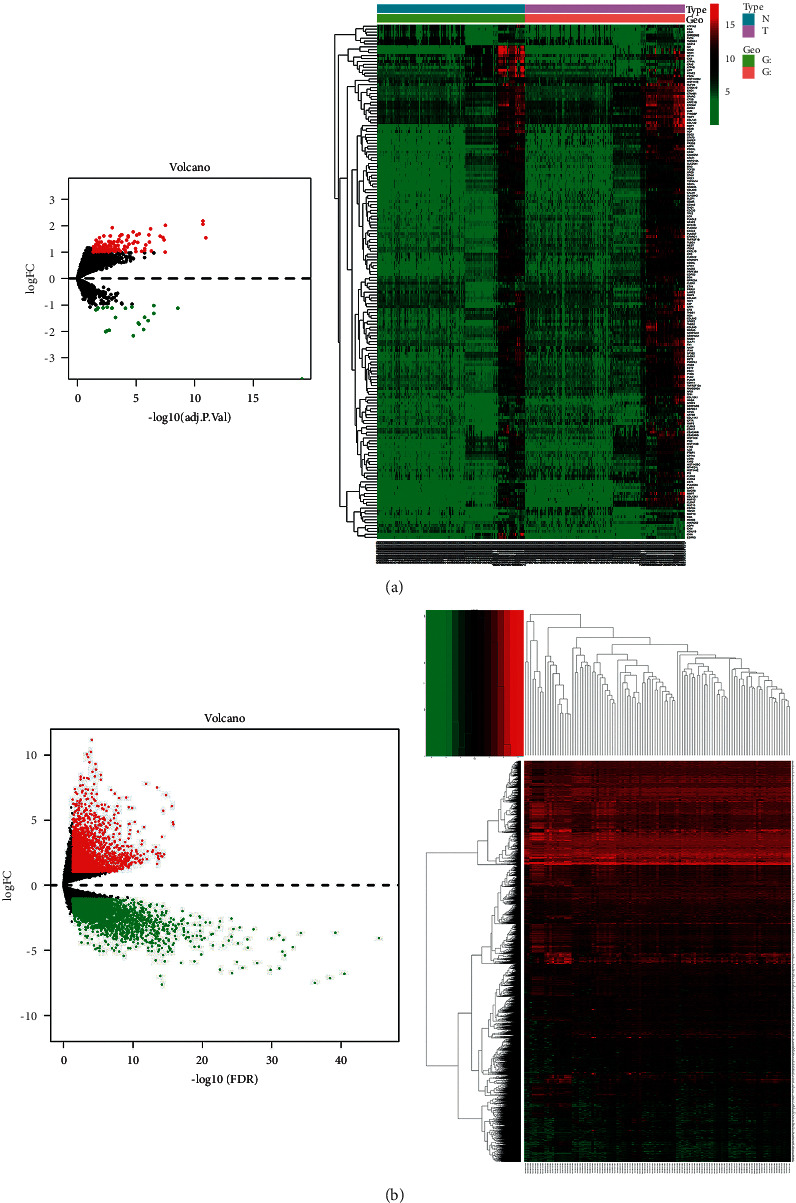
Differential genes volcano and heat map. (a) GEO. (b) TCGA.

134 healthy samples and 145 GC samples were available from the GEO database. Samples: GSE27342: 80/80; GSE33335: 25/25; GSE56807: 5/5; GSE79973: 10/10; GSE81948: 5/15; and GSE103236: 9/10. Using R4.1.0 to draw the Venn map (Figure 2(a)), 29438 genes were generated. With *P* < 0.05 and |log2(FC)| > 1 as screening conditions, the volcano and heat map were used to display 5918 DEGs (Figure 3(a)). 413 samples were obtained from TCGA database, including 92 healthy samples and 321 GC samples. With *P* < 0.05 and |log2(FC)| > 1 as screening conditions, a total of 176 DEGs were gathered and depicted using a volcano map and a heat map (Figure 3(b)). 6011 DEGs of union and 83 DEGs of intersection were obtained (Figure 2(b)) (Supplementary Table 6).

### 3.3. “Herbs-Components-Targets” Network of AM-CPV Analysis

**Figure 4:**
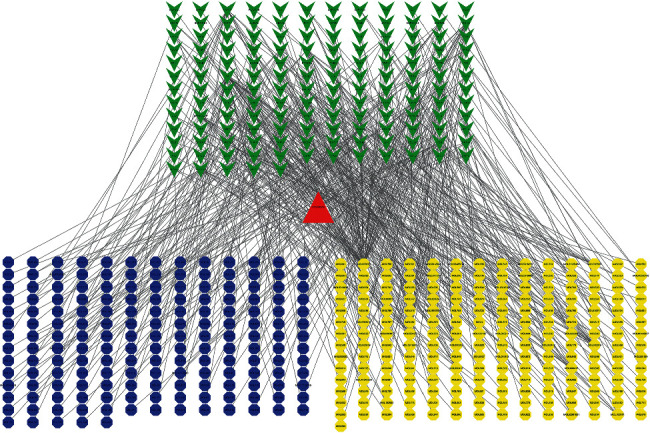
Herb-ingredients-targets (H-I-T) network. Blue node represents AM, yellow nodes represent targets of CPV, red node represents multidrug, and green nodes represent core active compounds of AM-CPV.

**Figure 5:**
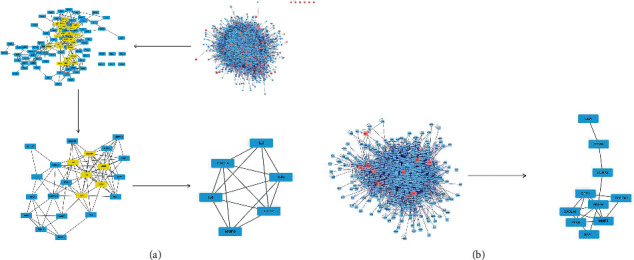
The process of topological screening for the PPI network. (a) Drug is matched with union differential genes. (b) Drugs are matched with overlapping differential genes.

149 AM-CPV drug-pairs and GC compound targets were chosen (Figure 2(c)). The composite target was included in the String database, and the unconnected target was removed to get the PPI network (Supplementary Table 7). BisoGenet showed that the network contained 409 nodes and 93772 edges. The “herbs-components-targets” network of GC was constructed (Figure 4), including 121 nodes and 374 edges. Then, the CytoNCA was used to further analyze the core targets in the network. After twice screening, 8 core targets were obtained (Supplementary Tables 8 and 9). The node transmits information and transmission efficiency as the “key target” of the follow-up research (Figure 5). It can be seen that STAT1, MMP9, IL6, HSP90AA1, JUN, CCL2, IFNG, and CXCL8 are the principal effect genes (Table 2 or Supplementary Tables 1 and 10). In the same method, it was found that CCNA2, MMP9, TYMS, CDA, COL1A1, MMP3, SPP1, CXCL10, STAT1, and PLAU were the main effective genes of AM-CPV drug-pairs and GC intersection DEGs in TCGA. MMP9 and STAT1 are the DEGs of both, suggesting that MMP9 and STAT1 may be the important genes of AM and CPV in the therapy of GC.

### 3.4. GO and KEGG Enrichment Analysis

**Figure 6:**
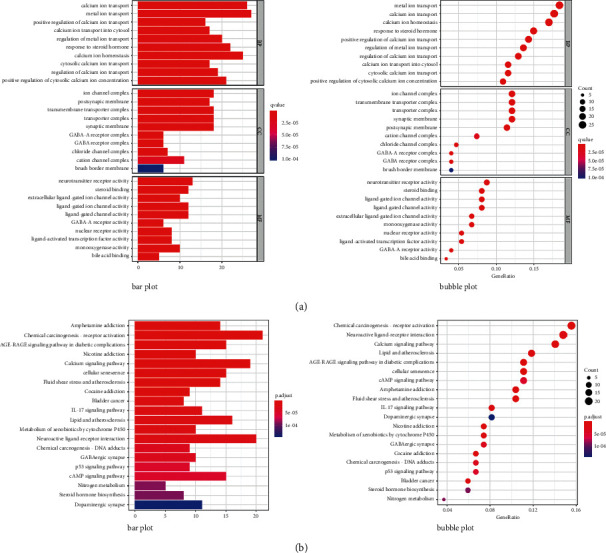
Enrichment analysis. (a) GO. (b) KEGG.

**Figure 7:**
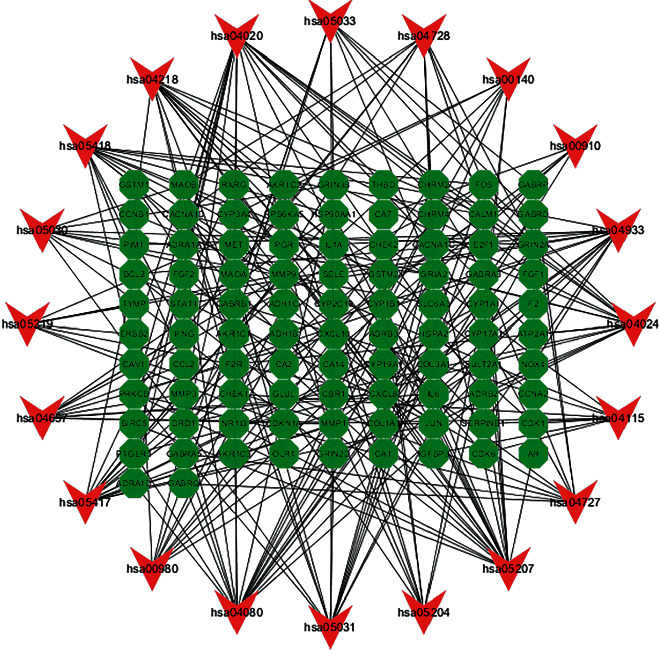
KEGG pathway enrichment analysis of the target-pathway network. The red nodes represent the pathways, whereas the green nodes represent the targets involved in these pathways. The edges represent the interactions between the targets and the pathways, and the node size is proportional to the degree of interaction.

GO enrichment analysis identified 1609 core targets involving molecular function (MF), cellular composition (CC), and biological process (BP). The MF mainly involves the channel activity (GO: 0015267), passive transmembrane transporter activity (GO: 0022803), and ion channel activity (GO: 0005216). The BP mainly involves ion channel complex (GO: 0034702), transmembrane transporter complex (GO: 1902495), and transporter complex (GO: 1990351). The CC mainly involves metal ion transport (GO: 0030001), calcium ion transport (GO: 0006816), and calcium ion homeostasis (GO: 0055074) (Supplementary Tables 11–13). In addition, the main signaling pathways involved in the treatment of GC were identified by KEGG enrichment analysis, including chemical carcinogenesis-receptor activation (hsa05207), neuroactive ligand-receptor interaction (hsa04080), calcium signaling pathway (hsa04020), AGE-RAGE signaling pathway in diabetic complications (hsa04933), cAMP signaling pathway (hsa04024), and PI3K-Akt signaling pathway (hsa04151) (Figures 6(a) and 6(b)). The network diagram of “core targets-signal pathways” was built (Figure 7) (Supplementary Tables 14 and 16).

### 3.5. Components-Targets Docking Analysis

**Figure 8:**
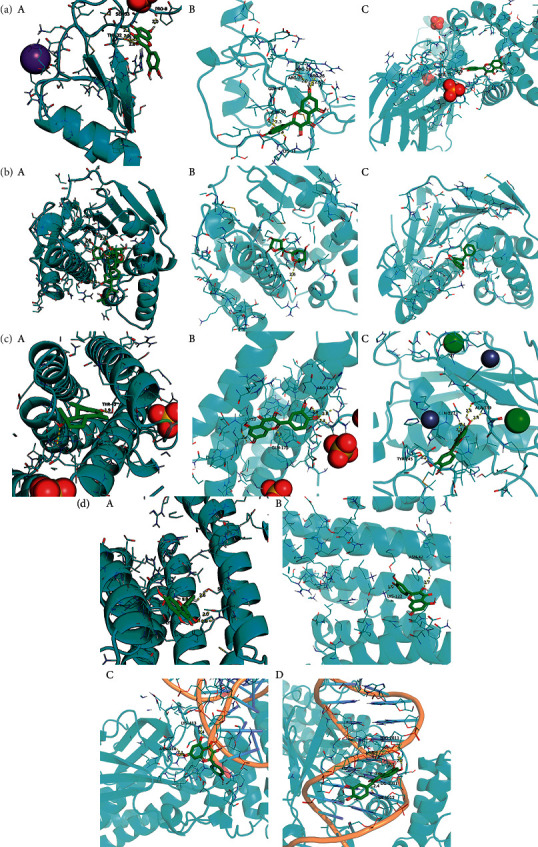
(a) Schematic 3D representation of the molecular docking model, active sites, and binding distances. A: CCL2: quercetin-3ifd; B: CXCL8: quercetin-4xdx; C: IFNG: quercetin-3bes. (b) A: HSP90AA1: isoastragaloside ii-2yk9; B: HSP90AA1: pinocembrin-2yk9; C: HSP90AA1: sucrose-2yk9. (c) A: IL6: procurcumenol-1alu; B: IL6: quercetin-1alu; C: MMP9: quercetin-6esm. (d) A: JUN: kaempferol-6y3v; B: JUN: quercetin-6y3v; C: STAT1: kaempferol-1bf5; D: STAT1: quercetin-1bf5.

The eight core targets were docked out of the PPI network topology. 3ifd (CCL2, containing ligands), 4xdx (CXCL8, containing ligands), 2yk9 (HSP90AA1, containing ligands), 3bes (IFNG, containing ligands), 1alu (IL6, containing ligands), 6y3v (JUN, containing ligands), 6esm (MMP9, containing ligands), and1bf5 (STAT1, containing ligands) were downloaded. Evaluation of the binding strength and activity of active components and targets by SYBYL2.0 software showed that the TotalScore of formononetin was 2.6282 less than 3. So, molecular docking of formononetin was not done. The affinity energies of the molecules were CCL2: quercetin-3ifd were −6.1 kcal/mol, CXCL8: quercetin-4xdx were −7.5 kcal/mol, HSP90AA1: isoastragaloside ii-2yk9 were −8.0 kcal/mol; pinocembrin-2yk9 were −8.0 kcal/mol, IFNG: quercetin-3bes were −7.4 kcal/mol, IL6: quercetin-1alu were −7.0 kcal/mol; procurcumenol-1alu were −6.5 kcal/mol, and JUN: quercetin-6y3v were −6.4 kcal/mol. Kaempferol-6y3v were −6.3 kcal/mol, MMP9: quercetin-6esm were −10.7 kcal/mol, STAT1: kaempferol-1bf5 were −9.8 kcal; quercetin-1bf5 were −9.4 kcal/mol (Supplementary Table 15). Molecular docking results of CCL2 indicated that there were three hydrogen bonds with THR-32 at the distance of 2.2 pm, 2.3 pm and 2.8 pm. There was one hydrogen bond with PRO-8 and SER-33 at the distances of 2.0 pm and 2.1 pm, respectively. The molecular docking result of CXCL8 showed that there were three hydrogen bonds with ARG-26 at the distances of 2.0 pm, 2.2 pm, and 2.5 pm. There was one hydrogen bond with GLU, CYS, and LYS-11 at the distances of 2.3 pm, 2.7 pm, and 2.4 pm, respectively. The molecular docking result of HSP90AA1 showed that there were two hydrogen bonds with LYS-58-isoastragaloside ii at the distances of 2.3 pm and 2.8 pm. There was one hydrogen bond with HIS-154, GLY-97, and GLY-97 at the distances of 2.3 pm, 2.8 pm, and 2.4 pm, respectively. There is no hydrogen bond with pinocembrin. There is a hydrogen bond between the bottom molecule of sucrose and ASP-54 at the distance of 2.6 pm. The molecular docking result of IFNG showed that there was one hydrogen bond with SER-76 at the distances of 1.9 pm. The molecular docking result of IL6 showed that there were four hydrogen bonds with SER-76 at the distances of 2.8 pm, 2.8 pm, 2.8 pm, and 2.3 pm. There was one hydrogen bond with LEU-33 and GLN-175 at the distances of 2.3 pm and 2.4 pm, respectively, and there was one hydrogen bond with the bottom molecule of procurements and THR-43 and GLN-156 at the distances of 1.9 pm and 2.5 pm, respectively. The molecular docking result of JUN shows that there was one hydrogen bond between quercetin ligand of ASN-42 and LYS-122 at distances of 2.7 pm and 1.9 pm, respectively, and two hydrogen bonds with kaempferol ligand-ASN-42 at distances of 2.0 pm and 2.6 pm, respectively. There was one hydrogen bond with SER-45 and LYS-122 at the distances of 2.6 pm and 1.9 pm, respectively. Molecular docking consequences of MMP9 indicated that there was one hydrogen bond with TYR-245 at the distance of 2.2 pm. There was two hydrogen bonds with ALA-189 and CLN-227 at the distances of 2.4 pm and 2.4 pm and 2.1 pm and 2.3 pm, respectively. Molecular docking results of STAT1 indicated that there was one hydrogen bond with kaempferol ligand-LYS-413 at the distances of 2.4 pm. There was two hydrogen bonds with LYS-413 at the distances of 2.1 pm and 2.2 pm. There was perhaps one hydrogen bond with quercetin ligand, DF-2011, DG-2010, DG-1011, and DT-1012 at the distances of 2.2 pm, 2.2 pm, 2.0 pm, and 2.4 pm, respectively (Figure 8) (Table 3 or Supplementary Table 15). It can be seen that quercetin and kaempferol are the main active ingredients of AM-CPV in treating GC.

The higher the absolute value of the lowest binding energy between the compound and the target is, the more stable the combination is. Results of molecular docking showed that the lowest binding energy of small molecule ligands and their corresponding protein receptors was all less than −5.0 kcal·mol/L, indicating that the binding condition was better, and the corresponding ligands and receptors had strong binding and high affinity.

## 4. Discussion

AM, a plant of the genus *Astragalus* in the Leguminosae, recorded in Shén Nóng Bĕn Căo Jīng (《神农本草经》Shen Nong's Classic of the Materia Medica), belongs to top-grade medicine. It can promote qi and secure the exterior, diuresis, suppressing toxin, and purulent muscle and is typically used in the treatment of shortness of breath, palpitation, fatigue, etc. [16]. Modern pharmacological studies have discovered that AM can increase leukocytes in blood and significantly improve the mononuclear macrophage system and the phagocytic function of leukocytes [17]. *Astragalus* polysaccharides may independently induce apoptosis of GC cells and enhance the apoptosis-promoting effect of adriamycin by reducing the expression and inhibitory function of CD4+ and CD25+ [18], which can improve immunologic function of patients with GC and delays the progression of GC. So, it can be utilized in the treatment of lung cancer, nasopharyngeal carcinoma, prostatic carcinoma, etc. [19]. CPV, a Zingiberaceae plant known as turmeric, taro, etc. returns to the liver and spleen meridians and could promote the flow of qi, break blood, eliminate stagnation, and relieve pain [20]. The main active components are sesquiterpenes and diphenyl heptane compounds, which function as antitumor and anodyne [21] mainly through STAT-3, HIF1/ROS, Wnt/*β*-catenin and Sp-1, cysteine, and other protein apoptosis pathways [22] playing an important role in anticancer of cervical cancer, endometrial cancer, lung cancer, and many other cancers [23].

We found most of the targets are quercetin (61 targets) and kaempferol (25 targets), and the literature on the analysis of the mechanism of quercetin and kaempferol acting on GC also confirmed the feasibility of drug therapy taken into account this active ingredient [24]. As we know, the abnormality of cell proliferation and apoptosis is the most basic feature of neoplastic cells. Therefore, the treatment should be focused on regulating the balance of GC cell proliferation and apoptosis and solving the immune and neurological problems caused by the imbalance [25]. Studies have demonstrated that quercetin can inhibit cell proliferation and strengthen apoptosis by activating p38-MAPK and other pathways to regulate cell proliferation rate and telomerase activity in GC patients [26]. It can regulate HIF-1*α* signal and Akt-mTor signal to affect the level of inflammatory factors in tumor microenvironment and initiate the autophagy process of GC cells [27]. It can enhance proapoptotic proteins such as Bad and Bax to induce apoptosis and antiproliferation of human GC cells [28]. Kaempferol exists widely in all kinds of fruit and vegetables. In addition to anti-inflammatory, antioxidant, antibacterial, and antiviral effects, kaempferol also has a certain anticancer effect on a variety of human cancers, including GC [29]. It has been observed that kaempferol is one of the estrogen-related receptor *α* (ERR *α*) reverse agonists, which can inhibit oxidative stress and tumor glycolysis through PI3K-Akt signal pathway and ErbB signal pathway to promote tumor cell apoptosis [30]. Song Haibin [31] showed that kaempferol could significantly inhibit the growth of transplanted GC after comparing the growth difference of GC tissue in vivo and in vitro. Further literature had found that kaempferol can regulate the apoptosis of gastric mucosal epithelial cells by inhibiting cell cycle transition and inducing cell cycle arrest in G1DB S cells [32]. In addition, kaempferol can inhibit the activity of transcription factors and proinflammatory enzymes and the expression of inflammation-related genes to decrease the occurrence of inflammation [29]. Inhibition of p38 signal pathway and MAPK-related extracellular signal-regulated kinase and activation of PI3K/AKT/mTOR signal transduction are also essential ways for kaempferol in anti-inflammation [33]. It can be observed that most of the compounds of AM-CPV drug-pairs take its pharmacological effects by regulating immune-inflammatory reaction, inhibiting cell proliferation and differentiation and promoting apoptosis, blocking, and inhibiting cell cycle, etc.

STAT1, MMP9, IL6, HSP90AA1, JUN, CCL2, IFNG, CXCL8, and other genes screened are the principal effect genes. MMP9 and STAT1 were chosen as core genes by overlapping intersection and union. MMP9, a member of the matrix metalloproteinases (MMPs) family, is one of the keys protease involved in the decomposition of the extracellular matrix in physiology and disease [34]. Numerous studies have found that MMP9 plays an essential role in invasion and metastasis of tumor, affecting tumor microenvironmental factors, with great value as a biomarker of various specific cancers [35]. CXCL8 is a multifunctional proinflammatory chemokine, which is significantly upregulated in tumor and tumor microenvironment as a key regulatory role [36]. CXCL8 can encourage the production and release of MMP9 and mediate the occurrence of GC and related abdominal pain. The expression of MMP9 and CXCL8 in well-differentiated tumors is considerably higher [37]. Studies have shown that MMP9 and CXCL8 can be biomarkers to predict prognosis and indicate a more aggressive phenotype of tumor [38]. As a member of the STAT family, STAT1, as a tumor inhibitor [39], is closely related to cell growth inhibition [40] which can inhibit proliferation and promote apoptosis of the tumor. Abril et al. [41] and other studies had found a quiet low expression level of STAT1 in gastric adenocarcinoma cell line. Yuan et al.'s [42] studies also confirmed that there is a low expression of STAT1 in 70.9% of GC tissue, suggesting that STAT1 can be an important marker for the diagnosis of GC. IL6, a type of cytokine of the chemokine family, which can activate and regulate immune cells, regulate proliferation and differentiation, plays an important role in inflammatory response [43], and also is the fundamental factor of chemotherapy resistance, which proposed that interleukin inhibitors can improve the tumor microenvironment to enhance the response of GC cells to chemotherapy drugs [44]. JUN is associated with the regulation of cell differentiation, proliferation, and apoptosis and can prevent T cell apoptosis by upregulating the antiapoptotic protein B-cell lymphoma or leukemia-3 [45]. C-JUN, a proto-oncogene (its protein is JUN), is a cellular homologue of viral oncoprotein v-JUN [46], which is the major carcinogenic transcription factor found [47], and CCL2 can not only maintain the drug resistance of drug-resistant cancer cells but also cause drug resistance of sensitive cancer cells. CCL2 knockout or autophagy induction successfully reverses the drug resistance of tumor cells, which have potential value in biomarkers and intervention targets of chemotherapy resistance [48]. IFNG is a form II interferon produced by immune cells such as T cells and NK cells. After interaction with its receptor IFNGR1, it can inhibit the growth of various kinds of tumors such as GC by regulating the JAK-STAT pathway [49]. It can be observed that AM-CPV may affect the development of GC in the aspects of intervention of inflammation, apoptosis, and cell metabolism.

Following that, we analyzed the enrichment of core targets by GO and KEGG. GO and KEGG enrichment analysis showed that the core target added value in a biological process, cell composition, and molecular function. In MF, 172 items are enriched mainly involving channel activity, passive transmembrane transporter activity, ion channel activity, etc. In BP, 1363 items are enriched, mainly involving metal ion transport, calcium ion transport and homeostasis, divalent inorganic cation homeostasis, etc. In CC, 39 items are enriched, mainly involving neuronal cell body membrane, ion channel complex, transmembrane transporter complex, transporter complex, synaptic membrane, etc. In addition, we also observed 20 pathways related to GC and constructed a “target-pathway” network with immune regulation, classical inflammation, multiple cancer pathways, retroactive ligand-receptor interaction, cell proliferation, and apoptosis involved. Among the signal pathways screened in this study, the highest correlation with the effective target of AM-CPV drug-pairs is the AGE-RAGE signal pathway, followed by calcium signal pathway, PI3K-Akt signal pathway, immune-inflammatory regulation, and cancer-related signal pathway. The combination of AGEs and its cell surface receptor RAGE can exert its pathophysiological effects in age-related diseases, diabetic complications. and the course of cancer [50]. The combination of AGEs and RAGE can activate NF-KB, mitogen-activated protein kinase, or JAK/STAT pathway to participate in inflammation, proliferation, and movement [51]. Deng et al. [52] found that AGEs-RAGE could activate MEK1/2/ERK pathway to upregulate the expression of Sp1, while MEK1/2 inhibitor U0126 decreased the expression of AGEs-induced Sp1 in GC cells by blocking the activation of ERK. Studies have found that the higher the expression of RAGE in cancer cells, the higher the malignant degree of the tumor, which may be attributed to the effect of RAGE on the adhesion between tumor cells, the decomposition of intracellular matrix, and the velocity of cell movement [53]. Hiroki et al. [54], by detecting the expression of RAGE in 8 kinds of GC cell lines and tissues, found that the expression of RAGE could increase the invasive ability of cell lines and decompose type IV collagen, thus promoting the migration of GC cells. Calcium signaling pathway can participate in the cell cycle. When cells are stimulated by physical and chemical effect, the calcium pathway transforms physical and chemical signals into biological signals and then affects cell differentiation [55]. The change of intracellular calcium concentration can regulate multiple pathways and play an important role in regulating cell life cycle, differentiation, and transfer [56]. Some studies have demonstrated that calcium can enhance the expression and function of calcium-sensitive receptors and induce the proliferation, migration, and invasion of GC cells, thus promoting the occurrence of GC [57].

In addition to the above two most important pathways, the following pathways also play a part in AM-CPV in the treatment of GC. ① PI3K/AKT signal pathway is one of the most frequently activated signal transduction pathways in tumors, which are closely linked to the growth, proliferation, and apoptosis of GC cells [58]. PI3K-Akt signal pathway can activate PI3K by promoting Akt phosphocreatine and regulate cell proliferation to promote tumor growth [59]. In addition, aerobic glycolysis, as a unique metabolic mode of tumor, can offer a large number of carbon sources for tumor tissue for proliferation and inhibit the aerobic oxidation pathway to avoid the production of oxygen radicals and apoptosis. In this process, PI3K/Akt signaling pathway plays an important role [60]. Active components of AM can inhibit the progression of GC by affecting Akt phosphorylation, downregulating Akt kinase, and then regulating PI3K-Akt signal pathway to inhibit epithelial-mesenchymal transformation of GC [61]. ② MAPK signaling pathway: MAPK is a group of threonine protein kinases that can be stimulated by different extracellular cells, regulating a variety of important cellular physiological and pathological processes, such as cell growth, differentiation, environmental stress adaptation, and inflammatory response [62].

Experiments have shown that MAPKl4 and MAPK3/1 pathways have the function of controlling the migration and proliferation of malignant tumors [63]. ③ IL-17 signal pathway: after the combination of IL-17 and the receptor, it can promote the release of proinflammatory cytokines through the MAP kinase pathway and NF-*κ*B pathway to amplify the inflammatory response [64]. ④ P53 signal pathway: P53 is a tumor suppressor protein that inhibits cell cycle progression and regulates the expression of various genes such as apoptosis [65]. P53 signal can prevent the emergence and development of GC by promoting DNA repair, inducing apoptosis, cell cycle control, programmed necrosis, etc. [66]. It has been found that aspergillosis can induce apoptosis of cancer cells through p53 signal pathway activation [67]. ⑤ TNF signaling pathway: TNF pathway participates in the process of cell growth, proliferation, inflammation, and immunity. After activated, TNF pathway can correspondingly induce NF-*κ*B to enter the nucleus and promote the production and release of inflammatory factors such as TNF-*α*, IL-8, and IL-6 [68]. TNF inflammatory cytokines which were abundant in tumor microenvironment can promote tumor growth, cause the imbalance of cell proliferation and apoptosis, and destroy the innate immune response to cancer cells, which is the key factor in the occurrence and development of GC [69].

Immuno-inflammatory signaling pathways such as HIF-1, toll-like receptor signaling pathway, and JAK-STAT signaling pathway are also involved in the treatment of GC with AM-CPV. In addition, chemical carcinogenesis-receptor activation, neuroactive ligand-receptor interaction, lipid and atherosclerosis, and other cancer-related signal pathways are rarely mentioned in the study of GC, which may become a new research direction.

AM-CPV has the following characteristics on the regulation of differentially expressed target genes in GC: ① STAT1, MMP9, IL6, HSP90AA1, JUN, CCL2, IFNG, and CXCL8 are affected by multiple active components at the same time, indicating that the target genes are regulated by multiple active components. ② Active components such as kaempferol and quercetin are targeted and regulated with multiple target genes at the same time, which shows that the active components play different roles in regulating GC lesions through multitarget gene crossover. ③ Quercetin targets 59 target genes at the same time, of which 30 are downregulated and 29 are upregulated. It can be observed that some of the functional components in AM-CPV drug-pairs act on both upregulated and downregulated genes at the same time. Therefore, it can be recognized that the drug may be bidirectional in targeting regulation of GC lesions.

## 5. Conclusions

To summarize, the AM-CPV medication combination governs the therapy of GC through a multitarget, multicomponent, and multisignal route, with a cooperative and bidirectional regulation mechanism. Its active ingredients may treat GC by regulating the expression of STAT1, MMP9, IL6, HSP90AA1, JUN, CCL2, IFNG, CXCL8, and other targets, activating or inhibiting immune-inflammatory and cancer signaling pathways such as the AGE-RAGE signal pathway, calcium signal pathway, and PI3K/AKT signal pathway, and regulating immune-inflammatory reaction, cell proliferation, differentiation, and apoptosis and antioxidant stress response, etc. Despite the fact that this work gives some theoretical foundation and research suggestions, it still has certain limitations. Improvements should be made in this direction: ① we might continue to conduct focused fundamental research on the key components of the AM-CPV drug-pairs, such as serum pharmacology and extraction of active components of the compound comprising pharmacology, based on the network pharmacology research results. ② More clinical trials are needed to see if the medication is suitable for long-term maintenance treatment in GC patients.

## Figures and Tables

**Table 1:** Core components of AM-CPV.

PubChem ID	Name	OB	DL	Source	Structure
5281654	Isorhamnetin	49.6	0.31	TCMSP	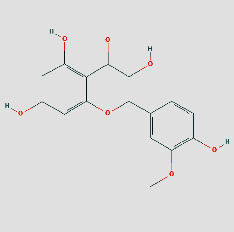
15689652	7-o-Methylisomucronulatol	74.69	0.3	TCMSP	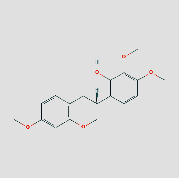
5280378	Formononetin	69.67	0.21	TCMSP	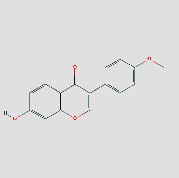
442811	Mucronulatol	NA	NA	ETCM	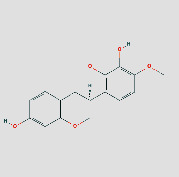
5280448	Calycosin	47.75	0.24	TCMSP	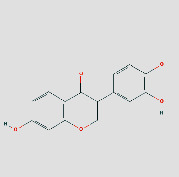
5280863	Kaempferol	41.88	0.24	TCMSP	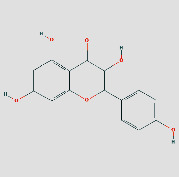
5280343	Quercetin	46.43	0.28	TCMSP	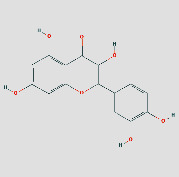
312601999	Borneol	NA	NA	ETCM	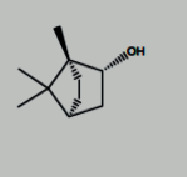
6321405	Isoborneol	NA	NA	TCMID	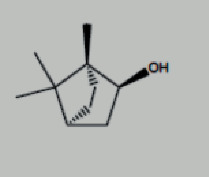
23500184	Bornylacetate	NA	NA	ETCM	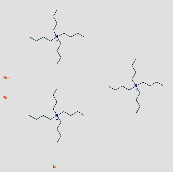
154279	Alpinetin	NA	NA	ETCM	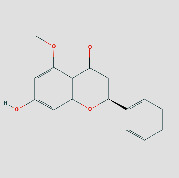
68071	Pinocembrin	NA	NA	ETCM	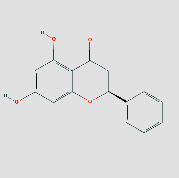
91711266	(R)-3,5,8a-Trimethyl-7,8,8a,9-tetrahydronaphtho[2,3-b]furan-4(6H)-one	NA	NA	TCMID	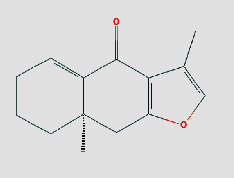
14681481	(5S,8R,9S,10S,13S,14S)-3-Ethyl-3-hydroxy-10	NA	NA	TCMID	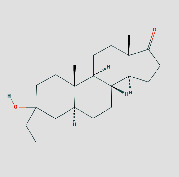
12302222	Tau-Cadinol	NA	NA	ETCM	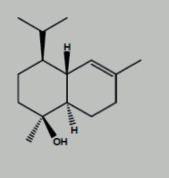
12304220	Cis-Cubenol	NA	NA	ETCM	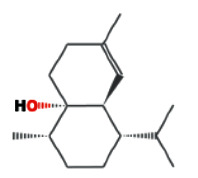
9992974	(4Ar,5R,5As,6Ar)-6A-Hydroxy-3,5A-dimethyl-5-(3-oxobutyl)-4,4A,5,5A,6,6A-hexahydro-2H-cyclopropa[F][1]benzofuran-2-one	NA	NA	ETCM	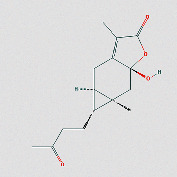

**Table 2:** Information on 8 core targets.

SUID	Gene symbol	Protein name	Betweenness	Closeness	Degree
198221	IL6	Interleukin-6	5.166666667	1	7
198138	JUN	Transcription factor AP-1	5.166666667	1	7
198213	IFNG	Interferon gamma	2.666666667	0.875	6
198196	CCL2	C-C motif chemokine 2	0.5	0.777777778	5
198223	CXCL8	Interleukin-8	0.5	0.777777778	5
198267	STAT1	Signal transducer and activator of transcription 1-alpha/beta	0	0.7	4
198205	MMP9	Matrix metalloproteinase-9	0	0.7	4
198105	HSP90AA1	Heat shock protein HSP 90-alpha	0	0.7	4

**Table 3:** The results of drug and disease molecular docking.

Ingredient	Molecular formula	Molecular weight	Crystal structure	Target	Affinity energy/(kj·mol^−1^)
Quercetin	C_15_H_10_O_7_	302.23 g/mol	3ifd	CCL2	−6.1
Quercetin	C_15_H_10_O_7_	302.23 g/mol	4xdx	CXCL8	−7.5
Isoastragaloside ii	C_43_H_70_O_15_	827.00 g/mol	2yk9	HSP90AA1	−8.0
Pinocembrin	C_15_H_12_O_4_	256.25 g/mol			−8.0
Sucrose	C_12_H_22_O_11_	342.3 g/mol			−5.9
Quercetin	C_15_H_10_O_7_	302.23 g/mol	3bes	IFNG	−7.4
Quercetin	C_15_H_10_O_7_	302.23 g/mol	1alu	IL6	−7.0
Procurcumenol	C_15_H_22_O_2_	234.33 g/mol			−6.5
Kaempferol	C_15_H_10_O_6_	286.24 g/mol	6y3v	JUN	−6.3
Quercetin	C_15_H_10_O_7_	302.23 g/mol			−6.4
Quercetin	C_15_H_10_O_7_	302.23 g/mol	6esm	MMP9	−10.7
Kaempferol	C_15_H_10_O_6_	286.24 g/mol	1bf5	STAT1	−9.8
Quercetin	C_15_H_10_O_7_	302.23 g/mol			−9.4

## Data Availability

All the data can be obtained from the open source website we provide, and the conclusion can be drawn through the analysis of the relevant software. The (diseases) data used to support the findings of this study have been deposited in the (GEO) repository (https://www.ncbi.nlm.nih.gov/geo/) and (TCGA) repository (https://portal.gdc.cancer.gov/). The (drugs) data used to support the findings of this study have been deposited in the [TCMSP] (TCMSP, https://tcmspw.com/tcmsp.php), [TCMID] (TCMID, http://47.100.169.139:8000/tcmid/), and [ETCM] (ETCM, http://www.tcmip.cn/ETCM/index.php/Home/Index/) repository. The (diseases-drugs) data used to support the findings of this study are included within the supplementary information files.

## Abbreviations

AM: *Astragalus mongholicus* (*huáng qí*)
CPV: *Curcuma phaeocaulis* Valeton (*é zhú*)
GC: Gastric carcinoma
GEO: Gene expression omnibus
TCGA: The cancer genome atlas
TCMSP: The TCM system pharmacology database
TCMID: Traditional Chinese medicine integrated database
ETCM: The Encyclopedia of Traditional Chinese Medicine
TCM: Traditional Chinese medicine
GO: Gene ontology
KEGG: Kyoto Encyclopedia of Genes and Genomes
PLGC: Precancerous lesions of gastric cancer
OB: Oral availability
DL: Drug-likeness
SMILES: Small molecular structure
DC: Degree centrality
CC: Closeness centrality
BC: Betweenness centrality.

## Supplementary Materials

Supplementary Materials Supplementary 1: core target gene. Supplementary 2: the active ingredients from TCMSP database. Supplementary 3: the active ingredients from TCMID database and relevant literature. Supplementary 4: the active ingredients from ETCM database and relevant literature. Supplementary 5: core active components. Supplementary 6: differential genes of GC overlapped by TCGA and GEO. Supplementary 7: 121 composite targets of drug pair of AM-CPV and GC. Supplementary 8: filter once after the 30 core target gene of AM-CPV and GC. Supplementary 9: filter twice after the 8 core target genes. Supplementary 10: drug pair of AM-CPV and intersection GC of core target genes. Supplementary 11: molecular function (MF) of GO enrichment analysis. Supplementary 12: cellular composition (CC) of GO enrichment analysis. Supplementary 13: biological process (BP) of GO enrichment analysis. Supplementary 14: core targets-signal pathways of KEGG enrichment analysis. Supplementary 15: the results of drug and disease molecular docking. Supplementary 16: the diagram of core signaling pathway. .
