# Supplementary material for: Mechanism of Herb Pairs Astragalus mongholicus and Curcuma phaeocaulis Valeton in Treating Gastric Carcinoma: A Network Pharmacology Combines with Differential Analysis and Molecular Docking
Source: Evid Based Complement Alternat Med. 2022 Mar 14;2022:8361431. doi: 10.1155/2022/8361431 (PMC8938068; doi:10.1155/2022/8361431)
Supplement: Supplementary Materials — Supplementary 1: core target gene. Supplementary 2: the active ingredients from TCMSP database. Supplementary 3: the active ingredients from TCMID database and relevant literature. Supplementary 4: the active ingredients from ETCM database and relevant literature. Supplementary 5: core active components. Supplementary 6: differential genes of GC overlapped by TCGA and GEO. Supplementary 7: 121 composite targets of drug pair of AM-CPV and GC. Supplementary 8: filter once after the 30 core target gene of AM-CPV and GC. Supplementary 9: filter twice after the 8 core target genes. Supplementary 10: drug pair of AM-CPV and intersection GC of core target genes. Supplementary 11: molecular function (MF) of GO enrichment analysis. Supplementary 12: cellular composition (CC) of GO enrichment analysis. Supplementary 13: biological process (BP) of GO enrichment analysis. Supplementary 14: core targets-signal pathways of KEGG enrichment analysis. Supplementary 15: the results of drug and disease molecular docking. Supplementary 16: the diagram of core signaling pathway. . [file 8361431.f1.doc]

Mechanism of herb-pairs Astragalus mongholicus-Curcuma phaeocaulis Valeton of treating gastric carcinoma: A network pharmacology combines with differential analysis and molecular docking

Zixuan Wu^1^, Xiyang Pan^1^, Chaosheng Deng^1^, Minjie Cai^3^, Kai Yuan^2^, Peidong Huang^2^, Guoqi Shi^1^

**Supplementary to the manuscript**

Contents of supplementary appendix

[Supplementary 1 3](#__RefHeading___Toc4137)

[Core target gene 3](#__RefHeading___Toc1574)

[Supplementary 2 4](#__RefHeading___Toc28737)

[The active ingredients from TCMSP database 4](#__RefHeading___Toc22953)

[Supplementary 3 33](#__RefHeading___Toc17014)

[The active ingredients from TCMID database and relevant literature 33](#__RefHeading___Toc1140)

[Supplementary 4 51](#__RefHeading___Toc18175)

[The active ingredients from ETCM database and relevant literature 51](#__RefHeading___Toc11455)

[Supplementary 5 106](#__RefHeading___Toc24203)

[Core active components 106](#__RefHeading___Toc7792)

[Supplementary 6 110](#__RefHeading___Toc9108)

[Differential genes of GC overlapped by TCGA and GEO 110](#__RefHeading___Toc14237)

[Supplementary 7 113](#__RefHeading___Toc31571)

[121 composite targets of drug pair of AM-CPV and GC 113](#__RefHeading___Toc12844)

[Supplementary 8 117](#__RefHeading___Toc17248)

[Filter once after the 30 core target gene of AM-CPV and GC 117](#__RefHeading___Toc31610)

[Supplementary 9 118](#__RefHeading___Toc10558)

[Filter twice after the 8 core target genes 118](#__RefHeading___Toc4652)

[Supplementary 10 119](#__RefHeading___Toc25446)

[Drug pair of AM-CPV and intersection GC of core target genes 119](#__RefHeading___Toc25496)

[Supplementary 11 120](#__RefHeading___Toc32657)

[Molecular function(MF) of GO enrichment analysis 120](#__RefHeading___Toc11626)

[Supplementary 12 137](#__RefHeading___Toc22597)

[Cellular composition(CC) of GO enrichment analysis 137](#__RefHeading___Toc19820)

[Supplementary 13 144](#__RefHeading___Toc5120)

[Biological process(BP) of GO enrichment analysis 144](#__RefHeading___Toc30877)

[Supplementary 14 203](#__RefHeading___Toc25195)

[Core targets-Signal pathways of KEGG enrichment analysis 203](#__RefHeading___Toc23551)

[Supplementary 15 213](#__RefHeading___Toc4222)

[The results of drug and disease molecular docking 213](#__RefHeading___Toc31931)

[Supplementary 16 214](#__RefHeading___Toc4741)

[The diagram of core signaling pathway 214](#__RefHeading___Toc6804)

# Supplementary 1

**Core target gene**

**Supplementary table 1. Drug pair of AM-LA and GC of core target gene.**

| SUID | Gene symbol | Protein name | Betweenness | Closeness | Degree |
| --- | --- | --- | --- | --- | --- |
| 198221 | IL6 | Interleukin-6 | 5.166666667 | 1 | 7 |
| 198138 | JUN | Transcription factor AP-1 | 5.166666667 | 1 | 7 |
| 198213 | IFNG | Interferon gamma | 2.666666667 | 0.875 | 6 |
| 198196 | CCL2 | C-C motif chemokine 2 | 0.5 | 0.777777778 | 5 |
| 198223 | CXCL8 | Interleukin-8 | 0.5 | 0.777777778 | 5 |
| 198267 | STAT1 | Signal transducer and activator of transcription 1-alpha/beta | 0 | 0.7 | 4 |
| 198205 | MMP9 | Matrix metalloproteinase-9 | 0 | 0.7 | 4 |
| 198105 | HSP90AA1 | Heat shock protein HSP 90-alpha | 0 | 0.7 | 4 |

# Supplementary 2

## **The active ingredients** **from TCMSP database**

**Supplementary table 2. The active ingredients of AM-CPV from TCMSP database.**

| Drug | MolId | MolName | Symbol |
| --- | --- | --- | --- |
| Curcuma phaeocaulis Valeton | MOL000296 | hederagenin | Progesterone receptor |
| Curcuma phaeocaulis Valeton | MOL000296 | hederagenin | Nuclear receptor coactivator 2 |
| Curcuma phaeocaulis Valeton | MOL000296 | hederagenin | Muscarinic acetylcholine receptor M3 |
| Curcuma phaeocaulis Valeton | MOL000296 | hederagenin | Muscarinic acetylcholine receptor M1 |
| Curcuma phaeocaulis Valeton | MOL000296 | hederagenin | Gamma-aminobutyric-acid receptor alpha-2 subunit |
| Curcuma phaeocaulis Valeton | MOL000296 | hederagenin | Gamma-aminobutyric-acid receptor alpha-3 subunit |
| Curcuma phaeocaulis Valeton | MOL000296 | hederagenin | Muscarinic acetylcholine receptor M2 |
| Curcuma phaeocaulis Valeton | MOL000296 | hederagenin | Alpha-1B adrenergic receptor |
| Curcuma phaeocaulis Valeton | MOL000296 | hederagenin | Gamma-aminobutyric acid receptor subunit alpha-1 |
| Curcuma phaeocaulis Valeton | MOL000296 | hederagenin | Glutamate receptor 2 |
| Curcuma phaeocaulis Valeton | MOL000296 | hederagenin | Gamma-aminobutyric-acid receptor subunit alpha-6 |
| Curcuma phaeocaulis Valeton | MOL000296 | hederagenin | Gamma-aminobutyric-acid receptor alpha-5 subunit |
| Curcuma phaeocaulis Valeton | MOL000296 | hederagenin | Ig gamma-1 chain C region |
| Curcuma phaeocaulis Valeton | MOL000296 | hederagenin | Alcohol dehydrogenase 1B |
| Curcuma phaeocaulis Valeton | MOL000296 | hederagenin | Alcohol dehydrogenase 1C |
| Curcuma phaeocaulis Valeton | MOL000296 | hederagenin | Lysozyme |
| Curcuma phaeocaulis Valeton | MOL000296 | hederagenin | Nicotinate-nucleotide--dimethylbenzimidazole phosphoribosyltransferase |
| Curcuma phaeocaulis Valeton | MOL000296 | hederagenin | Prostaglandin G/H synthase 1 |
| Curcuma phaeocaulis Valeton | MOL000296 | hederagenin | Sodium channel protein type 5 subunit alpha |
| Curcuma phaeocaulis Valeton | MOL000296 | hederagenin | Prostaglandin G/H synthase 2 |
| Curcuma phaeocaulis Valeton | MOL000296 | hederagenin | Retinoic acid receptor RXR-alpha |
| Curcuma phaeocaulis Valeton | MOL000296 | hederagenin | CGMP-inhibited 3',5'-cyclic phosphodiesterase A |
| Curcuma phaeocaulis Valeton | MOL000296 | hederagenin | Sodium-dependent noradrenaline transporter |
| Curcuma phaeocaulis Valeton | MOL000296 | hederagenin | Cytochrome P450-cam |
| Astragalus mongholicus | MOL000211 | Mairin | Progesterone receptor |
| Astragalus mongholicus | MOL000239 | Jaranol | Nitric oxide synthase, inducible |
| Astragalus mongholicus | MOL000239 | Jaranol | Prostaglandin G/H synthase 1 |
| Astragalus mongholicus | MOL000239 | Jaranol | Androgen receptor |
| Astragalus mongholicus | MOL000239 | Jaranol | Sodium channel protein type 5 subunit alpha |
| Astragalus mongholicus | MOL000239 | Jaranol | Prostaglandin G/H synthase 2 |
| Astragalus mongholicus | MOL000239 | Jaranol | Estrogen receptor beta |
| Astragalus mongholicus | MOL000239 | Jaranol | Dipeptidyl peptidase IV |
| Astragalus mongholicus | MOL000239 | Jaranol | Heat shock protein HSP 90 |
| Astragalus mongholicus | MOL000239 | Jaranol | Cell division protein kinase 2 |
| Astragalus mongholicus | MOL000239 | Jaranol | Serine/threonine-protein kinase Chk1 |
| Astragalus mongholicus | MOL000239 | Jaranol | Trypsin-1 |
| Astragalus mongholicus | MOL000239 | Jaranol | Nuclear receptor coactivator 2 |
| Astragalus mongholicus | MOL000239 | Jaranol | Calmodulin |
| Astragalus mongholicus | MOL000296 | hederagenin | Progesterone receptor |
| Astragalus mongholicus | MOL000296 | hederagenin | Nuclear receptor coactivator 2 |
| Astragalus mongholicus | MOL000296 | hederagenin | Muscarinic acetylcholine receptor M3 |
| Astragalus mongholicus | MOL000296 | hederagenin | Muscarinic acetylcholine receptor M1 |
| Astragalus mongholicus | MOL000296 | hederagenin | Gamma-aminobutyric-acid receptor alpha-2 subunit |
| Astragalus mongholicus | MOL000296 | hederagenin | Gamma-aminobutyric-acid receptor alpha-3 subunit |
| Astragalus mongholicus | MOL000296 | hederagenin | Muscarinic acetylcholine receptor M2 |
| Astragalus mongholicus | MOL000296 | hederagenin | Alpha-1B adrenergic receptor |
| Astragalus mongholicus | MOL000296 | hederagenin | Gamma-aminobutyric acid receptor subunit alpha-1 |
| Astragalus mongholicus | MOL000296 | hederagenin | Glutamate receptor 2 |
| Astragalus mongholicus | MOL000296 | hederagenin | Gamma-aminobutyric-acid receptor subunit alpha-6 |
| Astragalus mongholicus | MOL000296 | hederagenin | Gamma-aminobutyric-acid receptor alpha-5 subunit |
| Astragalus mongholicus | MOL000296 | hederagenin | Ig gamma-1 chain C region |
| Astragalus mongholicus | MOL000296 | hederagenin | Alcohol dehydrogenase 1B |
| Astragalus mongholicus | MOL000296 | hederagenin | Alcohol dehydrogenase 1C |
| Astragalus mongholicus | MOL000296 | hederagenin | Lysozyme |
| Astragalus mongholicus | MOL000296 | hederagenin | Nicotinate-nucleotide--dimethylbenzimidazole phosphoribosyltransferase |
| Astragalus mongholicus | MOL000296 | hederagenin | Prostaglandin G/H synthase 1 |
| Astragalus mongholicus | MOL000296 | hederagenin | Sodium channel protein type 5 subunit alpha |
| Astragalus mongholicus | MOL000296 | hederagenin | Prostaglandin G/H synthase 2 |
| Astragalus mongholicus | MOL000296 | hederagenin | Retinoic acid receptor RXR-alpha |
| Astragalus mongholicus | MOL000296 | hederagenin | CGMP-inhibited 3',5'-cyclic phosphodiesterase A |
| Astragalus mongholicus | MOL000296 | hederagenin | Sodium-dependent noradrenaline transporter |
| Astragalus mongholicus | MOL000296 | hederagenin | Cytochrome P450-cam |
| Astragalus mongholicus | MOL000033 | (3S,8S,9S,10R,13R,14S,17R)-10,13-dimethyl-17-[(2R,5S)-5-propan-2-yloctan-2-yl]-2,3,4,7,8,9,11,12,14,15,16,17-dodecahydro-1H-cyclopenta[a]phenanthren-3-ol | Progesterone receptor |
| Astragalus mongholicus | MOL000354 | isorhamnetin | Nitric oxide synthase, inducible |
| Astragalus mongholicus | MOL000354 | isorhamnetin | Prostaglandin G/H synthase 1 |
| Astragalus mongholicus | MOL000354 | isorhamnetin | Estrogen receptor |
| Astragalus mongholicus | MOL000354 | isorhamnetin | Androgen receptor |
| Astragalus mongholicus | MOL000354 | isorhamnetin | Peroxisome proliferator activated receptor gamma |
| Astragalus mongholicus | MOL000354 | isorhamnetin | Prostaglandin G/H synthase 2 |
| Astragalus mongholicus | MOL000354 | isorhamnetin | mRNA of Protein-tyrosine phosphatase, non-receptor type 1 |
| Astragalus mongholicus | MOL000354 | isorhamnetin | Estrogen receptor beta |
| Astragalus mongholicus | MOL000354 | isorhamnetin | Dipeptidyl peptidase IV |
| Astragalus mongholicus | MOL000354 | isorhamnetin | Mitogen-activated protein kinase 14 |
| Astragalus mongholicus | MOL000354 | isorhamnetin | Glycogen synthase kinase-3 beta |
| Astragalus mongholicus | MOL000354 | isorhamnetin | Heat shock protein HSP 90 |
| Astragalus mongholicus | MOL000354 | isorhamnetin | Cell division protein kinase 2 |
| Astragalus mongholicus | MOL000354 | isorhamnetin | Phosphatidylinositol-4,5-bisphosphate 3-kinase catalytic subunit, gamma isoform |
| Astragalus mongholicus | MOL000354 | isorhamnetin | mRNA of PKA Catalytic Subunit C-alpha |
| Astragalus mongholicus | MOL000354 | isorhamnetin | Trypsin-1 |
| Astragalus mongholicus | MOL000354 | isorhamnetin | Proto-oncogene serine/threonine-protein kinase Pim-1 |
| Astragalus mongholicus | MOL000354 | isorhamnetin | Cyclin-A2 |
| Astragalus mongholicus | MOL000354 | isorhamnetin | Nuclear receptor coactivator 2 |
| Astragalus mongholicus | MOL000354 | isorhamnetin | Calmodulin |
| Astragalus mongholicus | MOL000354 | isorhamnetin | Glycogen phosphorylase, muscle form |
| Astragalus mongholicus | MOL000354 | isorhamnetin | Peroxisome proliferator activated receptor delta |
| Astragalus mongholicus | MOL000354 | isorhamnetin | Serine/threonine-protein kinase Chk1 |
| Astragalus mongholicus | MOL000354 | isorhamnetin | Aldose reductase |
| Astragalus mongholicus | MOL000354 | isorhamnetin | Nuclear receptor coactivator 1 |
| Astragalus mongholicus | MOL000354 | isorhamnetin | Coagulation factor VII |
| Astragalus mongholicus | MOL000354 | isorhamnetin | Thrombin |
| Astragalus mongholicus | MOL000354 | isorhamnetin | Nitric-oxide synthase, endothelial |
| Astragalus mongholicus | MOL000354 | isorhamnetin | Acetylcholinesterase |
| Astragalus mongholicus | MOL000354 | isorhamnetin | Gamma-aminobutyric acid receptor subunit alpha-1 |
| Astragalus mongholicus | MOL000354 | isorhamnetin | Amine oxidase [flavin-containing] B |
| Astragalus mongholicus | MOL000354 | isorhamnetin | Glutamate receptor 2 |
| Astragalus mongholicus | MOL000354 | isorhamnetin | Cytochrome P450-cam |
| Astragalus mongholicus | MOL000354 | isorhamnetin | Transcription factor p65 |
| Astragalus mongholicus | MOL000354 | isorhamnetin | Xanthine dehydrogenase/oxidase |
| Astragalus mongholicus | MOL000354 | isorhamnetin | Neutrophil cytosol factor 1 |
| Astragalus mongholicus | MOL000354 | isorhamnetin | Oxidized low-density lipoprotein receptor 1 |
| Astragalus mongholicus | MOL000371 | 3,9-di-O-methylnissolin | Nitric oxide synthase, inducible |
| Astragalus mongholicus | MOL000371 | 3,9-di-O-methylnissolin | Prostaglandin G/H synthase 1 |
| Astragalus mongholicus | MOL000371 | 3,9-di-O-methylnissolin | Muscarinic acetylcholine receptor M3 |
| Astragalus mongholicus | MOL000371 | 3,9-di-O-methylnissolin | Thrombin |
| Astragalus mongholicus | MOL000371 | 3,9-di-O-methylnissolin | Muscarinic acetylcholine receptor M1 |
| Astragalus mongholicus | MOL000371 | 3,9-di-O-methylnissolin | Estrogen receptor |
| Astragalus mongholicus | MOL000371 | 3,9-di-O-methylnissolin | Beta-1 adrenergic receptor |
| Astragalus mongholicus | MOL000371 | 3,9-di-O-methylnissolin | Sodium channel protein type 5 subunit alpha |
| Astragalus mongholicus | MOL000371 | 3,9-di-O-methylnissolin | Prostaglandin G/H synthase 2 |
| Astragalus mongholicus | MOL000371 | 3,9-di-O-methylnissolin | Nitric-oxide synthase, endothelial |
| Astragalus mongholicus | MOL000371 | 3,9-di-O-methylnissolin | 5-hydroxytryptamine receptor 3A |
| Astragalus mongholicus | MOL000371 | 3,9-di-O-methylnissolin | Alpha-2C adrenergic receptor |
| Astragalus mongholicus | MOL000371 | 3,9-di-O-methylnissolin | Retinoic acid receptor RXR-alpha |
| Astragalus mongholicus | MOL000371 | 3,9-di-O-methylnissolin | Acetylcholinesterase |
| Astragalus mongholicus | MOL000371 | 3,9-di-O-methylnissolin | CGMP-inhibited 3',5'-cyclic phosphodiesterase A |
| Astragalus mongholicus | MOL000371 | 3,9-di-O-methylnissolin | Alpha-1B adrenergic receptor |
| Astragalus mongholicus | MOL000371 | 3,9-di-O-methylnissolin | Beta-2 adrenergic receptor |
| Astragalus mongholicus | MOL000371 | 3,9-di-O-methylnissolin | Alpha-1D adrenergic receptor |
| Astragalus mongholicus | MOL000371 | 3,9-di-O-methylnissolin | Mu-type opioid receptor |
| Astragalus mongholicus | MOL000371 | 3,9-di-O-methylnissolin | Gamma-aminobutyric acid receptor subunit alpha-1 |
| Astragalus mongholicus | MOL000371 | 3,9-di-O-methylnissolin | Trypsin-1 |
| Astragalus mongholicus | MOL000371 | 3,9-di-O-methylnissolin | Nuclear receptor coactivator 2 |
| Astragalus mongholicus | MOL000371 | 3,9-di-O-methylnissolin | Calmodulin |
| Astragalus mongholicus | MOL000378 | 7-O-methylisomucronulatol | Nitric oxide synthase, inducible |
| Astragalus mongholicus | MOL000378 | 7-O-methylisomucronulatol | Prostaglandin G/H synthase 1 |
| Astragalus mongholicus | MOL000378 | 7-O-methylisomucronulatol | Dopamine D1 receptor |
| Astragalus mongholicus | MOL000378 | 7-O-methylisomucronulatol | Muscarinic acetylcholine receptor M3 |
| Astragalus mongholicus | MOL000378 | 7-O-methylisomucronulatol | Thrombin |
| Astragalus mongholicus | MOL000378 | 7-O-methylisomucronulatol | Potassium voltage-gated channel subfamily H member 2 |
| Astragalus mongholicus | MOL000378 | 7-O-methylisomucronulatol | Muscarinic acetylcholine receptor M1 |
| Astragalus mongholicus | MOL000378 | 7-O-methylisomucronulatol | Estrogen receptor |
| Astragalus mongholicus | MOL000378 | 7-O-methylisomucronulatol | Androgen receptor |
| Astragalus mongholicus | MOL000378 | 7-O-methylisomucronulatol | Beta-1 adrenergic receptor |
| Astragalus mongholicus | MOL000378 | 7-O-methylisomucronulatol | Sodium channel protein type 5 subunit alpha |
| Astragalus mongholicus | MOL000378 | 7-O-methylisomucronulatol | Peroxisome proliferator activated receptor gamma |
| Astragalus mongholicus | MOL000378 | 7-O-methylisomucronulatol | Coagulation factor Xa |
| Astragalus mongholicus | MOL000378 | 7-O-methylisomucronulatol | Muscarinic acetylcholine receptor M5 |
| Astragalus mongholicus | MOL000378 | 7-O-methylisomucronulatol | Prostaglandin G/H synthase 2 |
| Astragalus mongholicus | MOL000378 | 7-O-methylisomucronulatol | Nitric-oxide synthase, endothelial |
| Astragalus mongholicus | MOL000378 | 7-O-methylisomucronulatol | Alpha-2C adrenergic receptor |
| Astragalus mongholicus | MOL000378 | 7-O-methylisomucronulatol | Muscarinic acetylcholine receptor M4 |
| Astragalus mongholicus | MOL000378 | 7-O-methylisomucronulatol | Retinoic acid receptor RXR-alpha |
| Astragalus mongholicus | MOL000378 | 7-O-methylisomucronulatol | Delta-type opioid receptor |
| Astragalus mongholicus | MOL000378 | 7-O-methylisomucronulatol | CGMP-inhibited 3',5'-cyclic phosphodiesterase A |
| Astragalus mongholicus | MOL000378 | 7-O-methylisomucronulatol | 5-hydroxytryptamine 2A receptor |
| Astragalus mongholicus | MOL000378 | 7-O-methylisomucronulatol | Alpha-1A adrenergic receptor |
| Astragalus mongholicus | MOL000378 | 7-O-methylisomucronulatol | Muscarinic acetylcholine receptor M2 |
| Astragalus mongholicus | MOL000378 | 7-O-methylisomucronulatol | Alpha-1B adrenergic receptor |
| Astragalus mongholicus | MOL000378 | 7-O-methylisomucronulatol | Sodium-dependent dopamine transporter |
| Astragalus mongholicus | MOL000378 | 7-O-methylisomucronulatol | Beta-2 adrenergic receptor |
| Astragalus mongholicus | MOL000378 | 7-O-methylisomucronulatol | Alpha-1D adrenergic receptor |
| Astragalus mongholicus | MOL000378 | 7-O-methylisomucronulatol | Sodium-dependent serotonin transporter |
| Astragalus mongholicus | MOL000378 | 7-O-methylisomucronulatol | Estrogen receptor beta |
| Astragalus mongholicus | MOL000378 | 7-O-methylisomucronulatol | Gamma-aminobutyric acid receptor subunit alpha-1 |
| Astragalus mongholicus | MOL000378 | 7-O-methylisomucronulatol | Dipeptidyl peptidase IV |
| Astragalus mongholicus | MOL000378 | 7-O-methylisomucronulatol | Mitogen-activated protein kinase 14 |
| Astragalus mongholicus | MOL000378 | 7-O-methylisomucronulatol | Glycogen synthase kinase-3 beta |
| Astragalus mongholicus | MOL000378 | 7-O-methylisomucronulatol | Heat shock protein HSP 90 |
| Astragalus mongholicus | MOL000378 | 7-O-methylisomucronulatol | Cell division protein kinase 2 |
| Astragalus mongholicus | MOL000378 | 7-O-methylisomucronulatol | Serine/threonine-protein kinase Chk1 |
| Astragalus mongholicus | MOL000378 | 7-O-methylisomucronulatol | mRNA of PKA Catalytic Subunit C-alpha |
| Astragalus mongholicus | MOL000378 | 7-O-methylisomucronulatol | Retinoic acid receptor RXR-beta |
| Astragalus mongholicus | MOL000378 | 7-O-methylisomucronulatol | Trypsin-1 |
| Astragalus mongholicus | MOL000378 | 7-O-methylisomucronulatol | Proto-oncogene serine/threonine-protein kinase Pim-1 |
| Astragalus mongholicus | MOL000378 | 7-O-methylisomucronulatol | Cyclin-A2 |
| Astragalus mongholicus | MOL000378 | 7-O-methylisomucronulatol | Nuclear receptor coactivator 2 |
| Astragalus mongholicus | MOL000378 | 7-O-methylisomucronulatol | Calcium-activated potassium channel subunit alpha 1 |
| Astragalus mongholicus | MOL000378 | 7-O-methylisomucronulatol | Calmodulin |
| Astragalus mongholicus | MOL000379 | 9,10-dimethoxypterocarpan-3-O-β-D-glucoside | Prostaglandin G/H synthase 2 |
| Astragalus mongholicus | MOL000379 | 9,10-dimethoxypterocarpan-3-O-β-D-glucoside | DNA topoisomerase II |
| Astragalus mongholicus | MOL000379 | 9,10-dimethoxypterocarpan-3-O-β-D-glucoside | Nuclear receptor coactivator 2 |
| Astragalus mongholicus | MOL000380 | (6aR,11aR)-9,10-dimethoxy-6a,11a-dihydro-6H-benzofurano[3,2-c]chromen-3-ol | Nitric oxide synthase, inducible |
| Astragalus mongholicus | MOL000380 | (6aR,11aR)-9,10-dimethoxy-6a,11a-dihydro-6H-benzofurano[3,2-c]chromen-3-ol | Prostaglandin G/H synthase 1 |
| Astragalus mongholicus | MOL000380 | (6aR,11aR)-9,10-dimethoxy-6a,11a-dihydro-6H-benzofurano[3,2-c]chromen-3-ol | Muscarinic acetylcholine receptor M3 |
| Astragalus mongholicus | MOL000380 | (6aR,11aR)-9,10-dimethoxy-6a,11a-dihydro-6H-benzofurano[3,2-c]chromen-3-ol | Thrombin |
| Astragalus mongholicus | MOL000380 | (6aR,11aR)-9,10-dimethoxy-6a,11a-dihydro-6H-benzofurano[3,2-c]chromen-3-ol | Muscarinic acetylcholine receptor M1 |
| Astragalus mongholicus | MOL000380 | (6aR,11aR)-9,10-dimethoxy-6a,11a-dihydro-6H-benzofurano[3,2-c]chromen-3-ol | Estrogen receptor |
| Astragalus mongholicus | MOL000380 | (6aR,11aR)-9,10-dimethoxy-6a,11a-dihydro-6H-benzofurano[3,2-c]chromen-3-ol | Sodium channel protein type 5 subunit alpha |
| Astragalus mongholicus | MOL000380 | (6aR,11aR)-9,10-dimethoxy-6a,11a-dihydro-6H-benzofurano[3,2-c]chromen-3-ol | Prostaglandin G/H synthase 2 |
| Astragalus mongholicus | MOL000380 | (6aR,11aR)-9,10-dimethoxy-6a,11a-dihydro-6H-benzofurano[3,2-c]chromen-3-ol | 5-hydroxytryptamine receptor 3A |
| Astragalus mongholicus | MOL000380 | (6aR,11aR)-9,10-dimethoxy-6a,11a-dihydro-6H-benzofurano[3,2-c]chromen-3-ol | Retinoic acid receptor RXR-alpha |
| Astragalus mongholicus | MOL000380 | (6aR,11aR)-9,10-dimethoxy-6a,11a-dihydro-6H-benzofurano[3,2-c]chromen-3-ol | Acetylcholinesterase |
| Astragalus mongholicus | MOL000380 | (6aR,11aR)-9,10-dimethoxy-6a,11a-dihydro-6H-benzofurano[3,2-c]chromen-3-ol | Alpha-1B adrenergic receptor |
| Astragalus mongholicus | MOL000380 | (6aR,11aR)-9,10-dimethoxy-6a,11a-dihydro-6H-benzofurano[3,2-c]chromen-3-ol | Beta-2 adrenergic receptor |
| Astragalus mongholicus | MOL000380 | (6aR,11aR)-9,10-dimethoxy-6a,11a-dihydro-6H-benzofurano[3,2-c]chromen-3-ol | Alpha-1D adrenergic receptor |
| Astragalus mongholicus | MOL000380 | (6aR,11aR)-9,10-dimethoxy-6a,11a-dihydro-6H-benzofurano[3,2-c]chromen-3-ol | Gamma-aminobutyric acid receptor subunit alpha-1 |
| Astragalus mongholicus | MOL000380 | (6aR,11aR)-9,10-dimethoxy-6a,11a-dihydro-6H-benzofurano[3,2-c]chromen-3-ol | Heat shock protein HSP 90 |
| Astragalus mongholicus | MOL000380 | (6aR,11aR)-9,10-dimethoxy-6a,11a-dihydro-6H-benzofurano[3,2-c]chromen-3-ol | Neuronal acetylcholine receptor protein, alpha-7 chain |
| Astragalus mongholicus | MOL000380 | (6aR,11aR)-9,10-dimethoxy-6a,11a-dihydro-6H-benzofurano[3,2-c]chromen-3-ol | Trypsin-1 |
| Astragalus mongholicus | MOL000380 | (6aR,11aR)-9,10-dimethoxy-6a,11a-dihydro-6H-benzofurano[3,2-c]chromen-3-ol | Nuclear receptor coactivator 2 |
| Astragalus mongholicus | MOL000380 | (6aR,11aR)-9,10-dimethoxy-6a,11a-dihydro-6H-benzofurano[3,2-c]chromen-3-ol | Nuclear receptor coactivator 1 |
| Astragalus mongholicus | MOL000380 | (6aR,11aR)-9,10-dimethoxy-6a,11a-dihydro-6H-benzofurano[3,2-c]chromen-3-ol | Calmodulin |
| Astragalus mongholicus | MOL000380 | (6aR,11aR)-9,10-dimethoxy-6a,11a-dihydro-6H-benzofurano[3,2-c]chromen-3-ol | Muscarinic acetylcholine receptor M4 |
| Astragalus mongholicus | MOL000387 | Bifendate | Prostaglandin G/H synthase 2 |
| Astragalus mongholicus | MOL000387 | Bifendate | Vascular endothelial growth factor receptor 2 |
| Astragalus mongholicus | MOL000387 | Bifendate | Hepatocyte growth factor receptor |
| Astragalus mongholicus | MOL000387 | Bifendate | Heat shock protein HSP 90 |
| Astragalus mongholicus | MOL000387 | Bifendate | Calcium-activated potassium channel subunit alpha 1 |
| Astragalus mongholicus | MOL000387 | Bifendate | Prostaglandin G/H synthase 1 |
| Astragalus mongholicus | MOL000387 | Bifendate | DNA topoisomerase II |
| Astragalus mongholicus | MOL000392 | formononetin | Nitric oxide synthase, inducible |
| Astragalus mongholicus | MOL000392 | formononetin | Prostaglandin G/H synthase 1 |
| Astragalus mongholicus | MOL000392 | formononetin | Muscarinic acetylcholine receptor M1 |
| Astragalus mongholicus | MOL000392 | formononetin | Estrogen receptor |
| Astragalus mongholicus | MOL000392 | formononetin | Androgen receptor |
| Astragalus mongholicus | MOL000392 | formononetin | Peroxisome proliferator activated receptor gamma |
| Astragalus mongholicus | MOL000392 | formononetin | Prostaglandin G/H synthase 2 |
| Astragalus mongholicus | MOL000392 | formononetin | Retinoic acid receptor RXR-alpha |
| Astragalus mongholicus | MOL000392 | formononetin | CGMP-inhibited 3',5'-cyclic phosphodiesterase A |
| Astragalus mongholicus | MOL000392 | formononetin | Alpha-1A adrenergic receptor |
| Astragalus mongholicus | MOL000392 | formononetin | Sodium-dependent dopamine transporter |
| Astragalus mongholicus | MOL000392 | formononetin | Beta-2 adrenergic receptor |
| Astragalus mongholicus | MOL000392 | formononetin | Sodium-dependent serotonin transporter |
| Astragalus mongholicus | MOL000392 | formononetin | Estrogen receptor beta |
| Astragalus mongholicus | MOL000392 | formononetin | Dipeptidyl peptidase IV |
| Astragalus mongholicus | MOL000392 | formononetin | Mitogen-activated protein kinase 14 |
| Astragalus mongholicus | MOL000392 | formononetin | Glycogen synthase kinase-3 beta |
| Astragalus mongholicus | MOL000392 | formononetin | Heat shock protein HSP 90 |
| Astragalus mongholicus | MOL000392 | formononetin | Cell division protein kinase 2 |
| Astragalus mongholicus | MOL000392 | formononetin | Amine oxidase [flavin-containing] B |
| Astragalus mongholicus | MOL000392 | formononetin | Serine/threonine-protein kinase Chk1 |
| Astragalus mongholicus | MOL000392 | formononetin | mRNA of PKA Catalytic Subunit C-alpha |
| Astragalus mongholicus | MOL000392 | formononetin | Trypsin-1 |
| Astragalus mongholicus | MOL000392 | formononetin | Proto-oncogene serine/threonine-protein kinase Pim-1 |
| Astragalus mongholicus | MOL000392 | formononetin | Cyclin-A2 |
| Astragalus mongholicus | MOL000392 | formononetin | Calmodulin |
| Astragalus mongholicus | MOL000392 | formononetin | cAMP-dependent protein kinase inhibitor alpha |
| Astragalus mongholicus | MOL000392 | formononetin | Thrombin |
| Astragalus mongholicus | MOL000392 | formononetin | Nitric-oxide synthase, endothelial |
| Astragalus mongholicus | MOL000392 | formononetin | Acetylcholinesterase |
| Astragalus mongholicus | MOL000392 | formononetin | Beta-lactamase |
| Astragalus mongholicus | MOL000392 | formononetin | Transcription factor AP-1 |
| Astragalus mongholicus | MOL000392 | formononetin | Peroxisome proliferator-activated receptor gamma |
| Astragalus mongholicus | MOL000392 | formononetin | Interleukin-4 |
| Astragalus mongholicus | MOL000392 | formononetin | NAD-dependent deacetylase sirtuin-1 |
| Astragalus mongholicus | MOL000392 | formononetin | ATP synthase subunit beta, mitochondrial |
| Astragalus mongholicus | MOL000392 | formononetin | NADH-ubiquinone oxidoreductase chain 6 |
| Astragalus mongholicus | MOL000392 | formononetin | 3 beta-hydroxysteroid dehydrogenase/Delta 5-->4-isomerase type 2 |
| Astragalus mongholicus | MOL000392 | formononetin | 3 beta-hydroxysteroid dehydrogenase/Delta 5-->4-isomerase type 1 |
| Astragalus mongholicus | MOL000412 | Mucronulatol | Nitric oxide synthase, inducible |
| Astragalus mongholicus | MOL000412 | Mucronulatol | Prostaglandin G/H synthase 1 |
| Astragalus mongholicus | MOL000412 | Mucronulatol | Estrogen receptor |
| Astragalus mongholicus | MOL000412 | Mucronulatol | Androgen receptor |
| Astragalus mongholicus | MOL000412 | Mucronulatol | Sodium channel protein type 5 subunit alpha |
| Astragalus mongholicus | MOL000412 | Mucronulatol | Peroxisome proliferator activated receptor gamma |
| Astragalus mongholicus | MOL000412 | Mucronulatol | Prostaglandin G/H synthase 2 |
| Astragalus mongholicus | MOL000412 | Mucronulatol | Retinoic acid receptor RXR-alpha |
| Astragalus mongholicus | MOL000412 | Mucronulatol | CGMP-inhibited 3',5'-cyclic phosphodiesterase A |
| Astragalus mongholicus | MOL000412 | Mucronulatol | Alpha-1B adrenergic receptor |
| Astragalus mongholicus | MOL000412 | Mucronulatol | Sodium-dependent dopamine transporter |
| Astragalus mongholicus | MOL000412 | Mucronulatol | Alpha-1D adrenergic receptor |
| Astragalus mongholicus | MOL000412 | Mucronulatol | Estrogen receptor beta |
| Astragalus mongholicus | MOL000412 | Mucronulatol | Dipeptidyl peptidase IV |
| Astragalus mongholicus | MOL000412 | Mucronulatol | Mitogen-activated protein kinase 14 |
| Astragalus mongholicus | MOL000412 | Mucronulatol | Glycogen synthase kinase-3 beta |
| Astragalus mongholicus | MOL000412 | Mucronulatol | Heat shock protein HSP 90 |
| Astragalus mongholicus | MOL000412 | Mucronulatol | Cell division protein kinase 2 |
| Astragalus mongholicus | MOL000412 | Mucronulatol | Serine/threonine-protein kinase Chk1 |
| Astragalus mongholicus | MOL000412 | Mucronulatol | Trypsin-1 |
| Astragalus mongholicus | MOL000412 | Mucronulatol | Proto-oncogene serine/threonine-protein kinase Pim-1 |
| Astragalus mongholicus | MOL000412 | Mucronulatol | Cyclin-A2 |
| Astragalus mongholicus | MOL000412 | Mucronulatol | Nuclear receptor coactivator 2 |
| Astragalus mongholicus | MOL000412 | Mucronulatol | Calmodulin |
| Astragalus mongholicus | MOL000417 | Calycosin | Nitric oxide synthase, inducible |
| Astragalus mongholicus | MOL000417 | Calycosin | Prostaglandin G/H synthase 1 |
| Astragalus mongholicus | MOL000417 | Calycosin | Estrogen receptor |
| Astragalus mongholicus | MOL000417 | Calycosin | Androgen receptor |
| Astragalus mongholicus | MOL000417 | Calycosin | Peroxisome proliferator activated receptor gamma |
| Astragalus mongholicus | MOL000417 | Calycosin | Prostaglandin G/H synthase 2 |
| Astragalus mongholicus | MOL000417 | Calycosin | Retinoic acid receptor RXR-alpha |
| Astragalus mongholicus | MOL000417 | Calycosin | CGMP-inhibited 3',5'-cyclic phosphodiesterase A |
| Astragalus mongholicus | MOL000417 | Calycosin | Estrogen receptor beta |
| Astragalus mongholicus | MOL000417 | Calycosin | Dipeptidyl peptidase IV |
| Astragalus mongholicus | MOL000417 | Calycosin | Mitogen-activated protein kinase 14 |
| Astragalus mongholicus | MOL000417 | Calycosin | Glycogen synthase kinase-3 beta |
| Astragalus mongholicus | MOL000417 | Calycosin | Heat shock protein HSP 90 |
| Astragalus mongholicus | MOL000417 | Calycosin | Cell division protein kinase 2 |
| Astragalus mongholicus | MOL000417 | Calycosin | Serine/threonine-protein kinase Chk1 |
| Astragalus mongholicus | MOL000417 | Calycosin | mRNA of PKA Catalytic Subunit C-alpha |
| Astragalus mongholicus | MOL000417 | Calycosin | Trypsin-1 |
| Astragalus mongholicus | MOL000417 | Calycosin | Proto-oncogene serine/threonine-protein kinase Pim-1 |
| Astragalus mongholicus | MOL000417 | Calycosin | Cyclin-A2 |
| Astragalus mongholicus | MOL000417 | Calycosin | Nuclear receptor coactivator 2 |
| Astragalus mongholicus | MOL000417 | Calycosin | Calmodulin |
| Astragalus mongholicus | MOL000417 | Calycosin | Beta-2 adrenergic receptor |
| Astragalus mongholicus | MOL000422 | kaempferol | Nitric oxide synthase, inducible |
| Astragalus mongholicus | MOL000422 | kaempferol | Prostaglandin G/H synthase 1 |
| Astragalus mongholicus | MOL000422 | kaempferol | Androgen receptor |
| Astragalus mongholicus | MOL000422 | kaempferol | Peroxisome proliferator activated receptor gamma |
| Astragalus mongholicus | MOL000422 | kaempferol | Prostaglandin G/H synthase 2 |
| Astragalus mongholicus | MOL000422 | kaempferol | Heat shock protein HSP 90 |
| Astragalus mongholicus | MOL000422 | kaempferol | Phosphatidylinositol-4,5-bisphosphate 3-kinase catalytic subunit, gamma isoform |
| Astragalus mongholicus | MOL000422 | kaempferol | mRNA of PKA Catalytic Subunit C-alpha |
| Astragalus mongholicus | MOL000422 | kaempferol | Nuclear receptor coactivator 2 |
| Astragalus mongholicus | MOL000422 | kaempferol | Dipeptidyl peptidase IV |
| Astragalus mongholicus | MOL000422 | kaempferol | Trypsin-1 |
| Astragalus mongholicus | MOL000422 | kaempferol | Progesterone receptor |
| Astragalus mongholicus | MOL000422 | kaempferol | Thrombin |
| Astragalus mongholicus | MOL000422 | kaempferol | Muscarinic acetylcholine receptor M1 |
| Astragalus mongholicus | MOL000422 | kaempferol | Nitric-oxide synthase, endothelial |
| Astragalus mongholicus | MOL000422 | kaempferol | Gamma-aminobutyric-acid receptor alpha-2 subunit |
| Astragalus mongholicus | MOL000422 | kaempferol | Acetylcholinesterase |
| Astragalus mongholicus | MOL000422 | kaempferol | Sodium-dependent noradrenaline transporter |
| Astragalus mongholicus | MOL000422 | kaempferol | Muscarinic acetylcholine receptor M2 |
| Astragalus mongholicus | MOL000422 | kaempferol | Alpha-1B adrenergic receptor |
| Astragalus mongholicus | MOL000422 | kaempferol | Gamma-aminobutyric acid receptor subunit alpha-1 |
| Astragalus mongholicus | MOL000422 | kaempferol | DNA topoisomerase II |
| Astragalus mongholicus | MOL000422 | kaempferol | Coagulation factor VII |
| Astragalus mongholicus | MOL000422 | kaempferol | Calmodulin |
| Astragalus mongholicus | MOL000422 | kaempferol | Transcription factor p65 |
| Astragalus mongholicus | MOL000422 | kaempferol | Inhibitor of nuclear factor kappa-B kinase subunit beta |
| Astragalus mongholicus | MOL000422 | kaempferol | RAC-alpha serine/threonine-protein kinase |
| Astragalus mongholicus | MOL000422 | kaempferol | Apoptosis regulator Bcl-2 |
| Astragalus mongholicus | MOL000422 | kaempferol | Apoptosis regulator BAX |
| Astragalus mongholicus | MOL000422 | kaempferol | Tumor necrosis factor |
| Astragalus mongholicus | MOL000422 | kaempferol | Transcription factor AP-1 |
| Astragalus mongholicus | MOL000422 | kaempferol | Activator of 90 kDa heat shock protein ATPase homolog 1 |
| Astragalus mongholicus | MOL000422 | kaempferol | Caspase-3 |
| Astragalus mongholicus | MOL000422 | kaempferol | Mitogen-activated protein kinase 8 |
| Astragalus mongholicus | MOL000422 | kaempferol | Xanthine dehydrogenase/oxidase |
| Astragalus mongholicus | MOL000422 | kaempferol | Interstitial collagenase |
| Astragalus mongholicus | MOL000422 | kaempferol | Signal transducer and activator of transcription 1-alpha/beta |
| Astragalus mongholicus | MOL000422 | kaempferol | Cell division control protein 2 homolog |
| Astragalus mongholicus | MOL000422 | kaempferol | Peroxisome proliferator-activated receptor gamma |
| Astragalus mongholicus | MOL000422 | kaempferol | Heme oxygenase 1 |
| Astragalus mongholicus | MOL000422 | kaempferol | Cytochrome P450 3A4 |
| Astragalus mongholicus | MOL000422 | kaempferol | Cytochrome P450 1A2 |
| Astragalus mongholicus | MOL000422 | kaempferol | Cytochrome P450 1A1 |
| Astragalus mongholicus | MOL000422 | kaempferol | Intercellular adhesion molecule 1 |
| Astragalus mongholicus | MOL000422 | kaempferol | E-selectin |
| Astragalus mongholicus | MOL000422 | kaempferol | Vascular cell adhesion protein 1 |
| Astragalus mongholicus | MOL000422 | kaempferol | Nuclear receptor subfamily 1 group I member 2 |
| Astragalus mongholicus | MOL000422 | kaempferol | Cytochrome P450 1B1 |
| Astragalus mongholicus | MOL000422 | kaempferol | Arachidonate 5-lipoxygenase |
| Astragalus mongholicus | MOL000422 | kaempferol | Hyaluronan synthase 2 |
| Astragalus mongholicus | MOL000422 | kaempferol | Glutathione S-transferase P |
| Astragalus mongholicus | MOL000422 | kaempferol | Aryl hydrocarbon receptor |
| Astragalus mongholicus | MOL000422 | kaempferol | 26S proteasome non-ATPase regulatory subunit 3 |
| Astragalus mongholicus | MOL000422 | kaempferol | Solute carrier family 2, facilitated glucose transporter member 4 |
| Astragalus mongholicus | MOL000422 | kaempferol | Nuclear receptor subfamily 1 group I member 3 |
| Astragalus mongholicus | MOL000422 | kaempferol | Insulin receptor |
| Astragalus mongholicus | MOL000422 | kaempferol | Type I iodothyronine deiodinase |
| Astragalus mongholicus | MOL000422 | kaempferol | Serine/threonine-protein phosphatase 2B catalytic subunit alpha isoform |
| Astragalus mongholicus | MOL000422 | kaempferol | Peroxidase C1A |
| Astragalus mongholicus | MOL000422 | kaempferol | Glutathione S-transferase Mu 1 |
| Astragalus mongholicus | MOL000422 | kaempferol | Glutathione S-transferase Mu 2 |
| Astragalus mongholicus | MOL000422 | kaempferol | Aldo-keto reductase family 1 member C3 |
| Astragalus mongholicus | MOL000422 | kaempferol | Antileukoproteinase |
| Astragalus mongholicus | MOL000433 | FA | Cell division protein kinase 2 |
| Astragalus mongholicus | MOL000433 | FA | Thrombin |
| Astragalus mongholicus | MOL000433 | FA | Glycogen synthase kinase-3 beta |
| Astragalus mongholicus | MOL000439 | isomucronulatol-7,2'-di-O-glucosiole | DNA topoisomerase II |
| Astragalus mongholicus | MOL000442 | 1,7-Dihydroxy-3,9-dimethoxy pterocarpene | Prostaglandin G/H synthase 2 |
| Astragalus mongholicus | MOL000442 | 1,7-Dihydroxy-3,9-dimethoxy pterocarpene | Retinoic acid receptor RXR-alpha |
| Astragalus mongholicus | MOL000442 | 1,7-Dihydroxy-3,9-dimethoxy pterocarpene | Heat shock protein HSP 90 |
| Astragalus mongholicus | MOL000442 | 1,7-Dihydroxy-3,9-dimethoxy pterocarpene | Trypsin-1 |
| Astragalus mongholicus | MOL000098 | quercetin | Prostaglandin G/H synthase 1 |
| Astragalus mongholicus | MOL000098 | quercetin | Androgen receptor |
| Astragalus mongholicus | MOL000098 | quercetin | Peroxisome proliferator activated receptor gamma |
| Astragalus mongholicus | MOL000098 | quercetin | Prostaglandin G/H synthase 2 |
| Astragalus mongholicus | MOL000098 | quercetin | Heat shock protein HSP 90 |
| Astragalus mongholicus | MOL000098 | quercetin | Phosphatidylinositol-4,5-bisphosphate 3-kinase catalytic subunit, gamma isoform |
| Astragalus mongholicus | MOL000098 | quercetin | Nuclear receptor coactivator 2 |
| Astragalus mongholicus | MOL000098 | quercetin | Dipeptidyl peptidase IV |
| Astragalus mongholicus | MOL000098 | quercetin | Aldose reductase |
| Astragalus mongholicus | MOL000098 | quercetin | Trypsin-1 |
| Astragalus mongholicus | MOL000098 | quercetin | DNA topoisomerase II |
| Astragalus mongholicus | MOL000098 | quercetin | Thrombin |
| Astragalus mongholicus | MOL000098 | quercetin | Potassium voltage-gated channel subfamily H member 2 |
| Astragalus mongholicus | MOL000098 | quercetin | Sodium channel protein type 5 subunit alpha |
| Astragalus mongholicus | MOL000098 | quercetin | Coagulation factor Xa |
| Astragalus mongholicus | MOL000098 | quercetin | Beta-2 adrenergic receptor |
| Astragalus mongholicus | MOL000098 | quercetin | Stromelysin-1 |
| Astragalus mongholicus | MOL000098 | quercetin | mRNA of PKA Catalytic Subunit C-alpha |
| Astragalus mongholicus | MOL000098 | quercetin | Coagulation factor VII |
| Astragalus mongholicus | MOL000098 | quercetin | Nitric-oxide synthase, endothelial |
| Astragalus mongholicus | MOL000098 | quercetin | Retinoic acid receptor RXR-alpha |
| Astragalus mongholicus | MOL000098 | quercetin | Acetylcholinesterase |
| Astragalus mongholicus | MOL000098 | quercetin | Gamma-aminobutyric acid receptor subunit alpha-1 |
| Astragalus mongholicus | MOL000098 | quercetin | Amine oxidase [flavin-containing] B |
| Astragalus mongholicus | MOL000098 | quercetin | Transcription factor p65 |
| Astragalus mongholicus | MOL000098 | quercetin | Epidermal growth factor receptor |
| Astragalus mongholicus | MOL000098 | quercetin | RAC-alpha serine/threonine-protein kinase |
| Astragalus mongholicus | MOL000098 | quercetin | Vascular endothelial growth factor A |
| Astragalus mongholicus | MOL000098 | quercetin | G1/S-specific cyclin-D1 |
| Astragalus mongholicus | MOL000098 | quercetin | Apoptosis regulator Bcl-2 |
| Astragalus mongholicus | MOL000098 | quercetin | Bcl-2-like protein 1 |
| Astragalus mongholicus | MOL000098 | quercetin | Proto-oncogene c-Fos |
| Astragalus mongholicus | MOL000098 | quercetin | Cyclin-dependent kinase inhibitor 1 |
| Astragalus mongholicus | MOL000098 | quercetin | Eukaryotic translation initiation factor 6 |
| Astragalus mongholicus | MOL000098 | quercetin | Apoptosis regulator BAX |
| Astragalus mongholicus | MOL000098 | quercetin | Caspase-9 |
| Astragalus mongholicus | MOL000098 | quercetin | Urokinase-type plasminogen activator |
| Astragalus mongholicus | MOL000098 | quercetin | 72 kDa type IV collagenase |
| Astragalus mongholicus | MOL000098 | quercetin | Matrix metalloproteinase-9 |
| Astragalus mongholicus | MOL000098 | quercetin | Mitogen-activated protein kinase 1 |
| Astragalus mongholicus | MOL000098 | quercetin | Interleukin-10 |
| Astragalus mongholicus | MOL000098 | quercetin | Pro-epidermal growth factor |
| Astragalus mongholicus | MOL000098 | quercetin | Retinoblastoma-associated protein |
| Astragalus mongholicus | MOL000098 | quercetin | Tumor necrosis factor |
| Astragalus mongholicus | MOL000098 | quercetin | Transcription factor AP-1 |
| Astragalus mongholicus | MOL000098 | quercetin | Interleukin-6 |
| Astragalus mongholicus | MOL000098 | quercetin | Cyclin-dependent kinase inhibitor 2A, isoforms 1/2/3 |
| Astragalus mongholicus | MOL000098 | quercetin | Activator of 90 kDa heat shock protein ATPase homolog 1 |
| Astragalus mongholicus | MOL000098 | quercetin | Caspase-3 |
| Astragalus mongholicus | MOL000098 | quercetin | Cellular tumor antigen p53 |
| Astragalus mongholicus | MOL000098 | quercetin | ETS domain-containing protein Elk-1 |
| Astragalus mongholicus | MOL000098 | quercetin | NF-kappa-B inhibitor alpha |
| Astragalus mongholicus | MOL000098 | quercetin | NADPH--cytochrome P450 reductase |
| Astragalus mongholicus | MOL000098 | quercetin | Ornithine decarboxylase |
| Astragalus mongholicus | MOL000098 | quercetin | Xanthine dehydrogenase/oxidase |
| Astragalus mongholicus | MOL000098 | quercetin | Caspase-8 |
| Astragalus mongholicus | MOL000098 | quercetin | DNA topoisomerase 1 |
| Astragalus mongholicus | MOL000098 | quercetin | RAF proto-oncogene serine/threonine-protein kinase |
| Astragalus mongholicus | MOL000098 | quercetin | Superoxide dismutase [Cu-Zn] |
| Astragalus mongholicus | MOL000098 | quercetin | Protein kinase C alpha type |
| Astragalus mongholicus | MOL000098 | quercetin | Interstitial collagenase |
| Astragalus mongholicus | MOL000098 | quercetin | Hypoxia-inducible factor 1-alpha |
| Astragalus mongholicus | MOL000098 | quercetin | Signal transducer and activator of transcription 1-alpha/beta |
| Astragalus mongholicus | MOL000098 | quercetin | Protein CBFA2T1 |
| Astragalus mongholicus | MOL000098 | quercetin | Probable E3 ubiquitin-protein ligase HERC5 |
| Astragalus mongholicus | MOL000098 | quercetin | Cell division control protein 2 homolog |
| Astragalus mongholicus | MOL000098 | quercetin | 78 kDa glucose-regulated protein |
| Astragalus mongholicus | MOL000098 | quercetin | Receptor tyrosine-protein kinase erbB-2 |
| Astragalus mongholicus | MOL000098 | quercetin | Peroxisome proliferator-activated receptor gamma |
| Astragalus mongholicus | MOL000098 | quercetin | Acetyl-CoA carboxylase 1 |
| Astragalus mongholicus | MOL000098 | quercetin | Heme oxygenase 1 |
| Astragalus mongholicus | MOL000098 | quercetin | Cytochrome P450 3A4 |
| Astragalus mongholicus | MOL000098 | quercetin | Cytochrome P450 1A2 |
| Astragalus mongholicus | MOL000098 | quercetin | Caveolin-1 |
| Astragalus mongholicus | MOL000098 | quercetin | Myc proto-oncogene protein |
| Astragalus mongholicus | MOL000098 | quercetin | Tissue factor |
| Astragalus mongholicus | MOL000098 | quercetin | Gap junction alpha-1 protein |
| Astragalus mongholicus | MOL000098 | quercetin | Cytochrome P450 1A1 |
| Astragalus mongholicus | MOL000098 | quercetin | Intercellular adhesion molecule 1 |
| Astragalus mongholicus | MOL000098 | quercetin | Interleukin-1 beta |
| Astragalus mongholicus | MOL000098 | quercetin | C-C motif chemokine 2 |
| Astragalus mongholicus | MOL000098 | quercetin | E-selectin |
| Astragalus mongholicus | MOL000098 | quercetin | Vascular cell adhesion protein 1 |
| Astragalus mongholicus | MOL000098 | quercetin | Prostaglandin E2 receptor EP3 subtype |
| Astragalus mongholicus | MOL000098 | quercetin | Interleukin-8 |
| Astragalus mongholicus | MOL000098 | quercetin | Protein kinase C beta type |
| Astragalus mongholicus | MOL000098 | quercetin | Baculoviral IAP repeat-containing protein 5 |
| Astragalus mongholicus | MOL000098 | quercetin | Dual oxidase 2 |
| Astragalus mongholicus | MOL000098 | quercetin | Nitric oxide synthase, endothelial |
| Astragalus mongholicus | MOL000098 | quercetin | Heat shock protein beta-1 |
| Astragalus mongholicus | MOL000098 | quercetin | Transforming growth factor beta-1 |
| Astragalus mongholicus | MOL000098 | quercetin | Estrogen sulfotransferase |
| Astragalus mongholicus | MOL000098 | quercetin | Maltase-glucoamylase, intestinal |
| Astragalus mongholicus | MOL000098 | quercetin | Interleukin-2 |
| Astragalus mongholicus | MOL000098 | quercetin | Nuclear receptor subfamily 1 group I member 2 |
| Astragalus mongholicus | MOL000098 | quercetin | Cytochrome P450 1B1 |
| Astragalus mongholicus | MOL000098 | quercetin | G2/mitotic-specific cyclin-B1 |
| Astragalus mongholicus | MOL000098 | quercetin | Tissue-type plasminogen activator |
| Astragalus mongholicus | MOL000098 | quercetin | Thrombomodulin |
| Astragalus mongholicus | MOL000098 | quercetin | Plasminogen activator inhibitor 1 |
| Astragalus mongholicus | MOL000098 | quercetin | Collagen alpha-1(I) chain |
| Astragalus mongholicus | MOL000098 | quercetin | Interferon gamma |
| Astragalus mongholicus | MOL000098 | quercetin | Arachidonate 5-lipoxygenase |
| Astragalus mongholicus | MOL000098 | quercetin | Phosphatidylinositol-3,4,5-trisphosphate 3-phosphatase and dual-specificity protein phosphatase PTEN |
| Astragalus mongholicus | MOL000098 | quercetin | Interleukin-1 alpha |
| Astragalus mongholicus | MOL000098 | quercetin | Myeloperoxidase |
| Astragalus mongholicus | MOL000098 | quercetin | DNA topoisomerase 2-alpha |
| Astragalus mongholicus | MOL000098 | quercetin | Neutrophil cytosol factor 1 |
| Astragalus mongholicus | MOL000098 | quercetin | ATP-binding cassette sub-family G member 2 |
| Astragalus mongholicus | MOL000098 | quercetin | Hyaluronan synthase 2 |
| Astragalus mongholicus | MOL000098 | quercetin | Glutathione S-transferase P |
| Astragalus mongholicus | MOL000098 | quercetin | Nuclear factor erythroid 2-related factor 2 |
| Astragalus mongholicus | MOL000098 | quercetin | NAD(P)H dehydrogenase [quinone] 1 |
| Astragalus mongholicus | MOL000098 | quercetin | Poly [ADP-ribose] polymerase 1 |
| Astragalus mongholicus | MOL000098 | quercetin | Aryl hydrocarbon receptor |
| Astragalus mongholicus | MOL000098 | quercetin | 26S proteasome non-ATPase regulatory subunit 3 |
| Astragalus mongholicus | MOL000098 | quercetin | Solute carrier family 2, facilitated glucose transporter member 4 |
| Astragalus mongholicus | MOL000098 | quercetin | Collagen alpha-1(III) chain |
| Astragalus mongholicus | MOL000098 | quercetin | DNA gyrase subunit B |
| Astragalus mongholicus | MOL000098 | quercetin | C-X-C motif chemokine 11 |
| Astragalus mongholicus | MOL000098 | quercetin | C-X-C motif chemokine 2 |
| Astragalus mongholicus | MOL000098 | quercetin | DDB1- and CUL4-associated factor 5 |
| Astragalus mongholicus | MOL000098 | quercetin | Nuclear receptor subfamily 1 group I member 3 |
| Astragalus mongholicus | MOL000098 | quercetin | Serine/threonine-protein kinase Chk2 |
| Astragalus mongholicus | MOL000098 | quercetin | Insulin receptor |
| Astragalus mongholicus | MOL000098 | quercetin | Claudin-4 |
| Astragalus mongholicus | MOL000098 | quercetin | Peroxisome proliferator-activated receptor alpha |
| Astragalus mongholicus | MOL000098 | quercetin | Peroxisome proliferator-activated receptor delta |
| Astragalus mongholicus | MOL000098 | quercetin | Heat shock factor protein 1 |
| Astragalus mongholicus | MOL000098 | quercetin | C-reactive protein |
| Astragalus mongholicus | MOL000098 | quercetin | C-X-C motif chemokine 10 |
| Astragalus mongholicus | MOL000098 | quercetin | Inhibitor of nuclear factor kappa-B kinase subunit alpha |
| Astragalus mongholicus | MOL000098 | quercetin | Osteopontin |
| Astragalus mongholicus | MOL000098 | quercetin | Runt-related transcription factor 2 |
| Astragalus mongholicus | MOL000098 | quercetin | Ras association domain-containing protein 1 |
| Astragalus mongholicus | MOL000098 | quercetin | Transcription factor E2F1 |
| Astragalus mongholicus | MOL000098 | quercetin | Transcription factor E2F2 |
| Astragalus mongholicus | MOL000098 | quercetin | Prostatic acid phosphatase |
| Astragalus mongholicus | MOL000098 | quercetin | Cathepsin D |
| Astragalus mongholicus | MOL000098 | quercetin | Insulin-like growth factor-binding protein 3 |
| Astragalus mongholicus | MOL000098 | quercetin | Insulin-like growth factor II |
| Astragalus mongholicus | MOL000098 | quercetin | CD40 ligand |
| Astragalus mongholicus | MOL000098 | quercetin | Interferon regulatory factor 1 |
| Astragalus mongholicus | MOL000098 | quercetin | Receptor tyrosine-protein kinase erbB-3 |
| Astragalus mongholicus | MOL000098 | quercetin | Serum paraoxonase/arylesterase 1 |
| Astragalus mongholicus | MOL000098 | quercetin | Type I iodothyronine deiodinase |
| Astragalus mongholicus | MOL000098 | quercetin | Procollagen C-endopeptidase enhancer 1 |
| Astragalus mongholicus | MOL000098 | quercetin | Puromycin-sensitive aminopeptidase |
| Astragalus mongholicus | MOL000098 | quercetin | Hexokinase-2 |
| Astragalus mongholicus | MOL000098 | quercetin | Homeobox protein Nkx-3.1 |
| Astragalus mongholicus | MOL000098 | quercetin | Ras GTPase-activating protein 1 |
| Astragalus mongholicus | MOL000098 | quercetin | Peroxidase C1A |
| Astragalus mongholicus | MOL000098 | quercetin | Glutathione S-transferase Mu 1 |
| Astragalus mongholicus | MOL000098 | quercetin | Glutathione S-transferase Mu 2 |

# Supplementary 3

## **The active ingredients from TCMID database and relevant** **literature**

**Supplementary table 3a. AM of the active ingredients from TCMID database and relevant literature.**

| Drug | MolId | MolName | Symbol | Source |
| --- | --- | --- | --- | --- |
| Astragalus mongholicus | MOL162933 | (6ar, 11ar)-10-hydroxy-3, 9-dimethoxypterocarpane | Protein-tyrosine phosphatase 1B | TCMID |
| Astragalus mongholicus | MOL162933 | (6ar, 11ar)-10-hydroxy-3, 9-dimethoxypterocarpane | Estrogen receptor alpha | TCMID |
| Astragalus mongholicus | MOL162933 | (6ar, 11ar)-10-hydroxy-3, 9-dimethoxypterocarpane | Estrogen receptor beta | TCMID |
| Astragalus mongholicus | MOL162933 | (6ar, 11ar)-10-hydroxy-3, 9-dimethoxypterocarpane | Cytochrome P450 19A1 | TCMID |
| Astragalus mongholicus | MOL162933 | (6ar, 11ar)-10-hydroxy-3, 9-dimethoxypterocarpane | Ribosomal protein S6 kinase alpha 5 | TCMID |
| Astragalus mongholicus | MOL162933 | (6ar, 11ar)-10-hydroxy-3, 9-dimethoxypterocarpane | Monoamine oxidase A | TCMID |
| Astragalus mongholicus | MOL162933 | (6ar, 11ar)-10-hydroxy-3, 9-dimethoxypterocarpane | Arachidonate 12-lipoxygenase | TCMID |
| Astragalus mongholicus | MOL162933 | (6ar, 11ar)-10-hydroxy-3, 9-dimethoxypterocarpane | Glycogen synthase kinase-3 beta | TCMID |
| Astragalus mongholicus | MOL162933 | (6ar, 11ar)-10-hydroxy-3, 9-dimethoxypterocarpane | Eukaryotic initiation factor 4A-I | TCMID |
| Astragalus mongholicus | MOL162933 | (6ar, 11ar)-10-hydroxy-3, 9-dimethoxypterocarpane | Tyrosine-protein kinase SRC | TCMID |
| Astragalus mongholicus | MOL5318279 | 2-hydroxy-3-methoxystrychnine | Poly [ADP-ribose] polymerase-1 | TCMID |
| Astragalus mongholicus | MOL5318279 | 2-hydroxy-3-methoxystrychnine | Serine/threonine-protein kinase Nek1 | TCMID |
| Astragalus mongholicus | MOL5318279 | 2-hydroxy-3-methoxystrychnine | Neurokinin 1 receptor | TCMID |
| Astragalus mongholicus | MOL5318279 | 2-hydroxy-3-methoxystrychnine | Epidermal growth factor receptor erbB1 | TCMID |
| Astragalus mongholicus | MOL5318279 | 2-hydroxy-3-methoxystrychnine | Tyrosine-protein kinase JAK3 | TCMID |
| Astragalus mongholicus | MOL5318279 | 2-hydroxy-3-methoxystrychnine | Tyrosine-protein kinase JAK1 | TCMID |
| Astragalus mongholicus | MOL5318279 | 2-hydroxy-3-methoxystrychnine | Tyrosine-protein kinase JAK2 | TCMID |
| Astragalus mongholicus | MOL5318279 | 2-hydroxy-3-methoxystrychnine | Tyrosine-protein kinase TYK2 | TCMID |
| Astragalus mongholicus | MOL5318279 | 2-hydroxy-3-methoxystrychnine | Adrenergic receptor beta | TCMID |
| Astragalus mongholicus | MOL5318279 | 2-hydroxy-3-methoxystrychnine | Beta-3 adrenergic receptor | TCMID |
| Astragalus mongholicus | MOL21125454 | 20(r)-21,24-cyclo-3beta,25-dihydroxyl-dammar-23(24)-en-21-one | Niemann-Pick C1-like protein 1 | TCMID |
| Astragalus mongholicus | MOL21125454 | 20(r)-21,24-cyclo-3beta,25-dihydroxyl-dammar-23(24)-en-21-one | 11-beta-hydroxysteroid dehydrogenase 1 | TCMID |
| Astragalus mongholicus | MOL21125454 | 20(r)-21,24-cyclo-3beta,25-dihydroxyl-dammar-23(24)-en-21-one | Cytochrome P450 19A1 | TCMID |
| Astragalus mongholicus | MOL21125454 | 20(r)-21,24-cyclo-3beta,25-dihydroxyl-dammar-23(24)-en-21-one | Glucocorticoid receptor | TCMID |
| Astragalus mongholicus | MOL21125454 | 20(r)-21,24-cyclo-3beta,25-dihydroxyl-dammar-23(24)-en-21-one | 11-beta-hydroxysteroid dehydrogenase 2 | TCMID |
| Astragalus mongholicus | MOL21125454 | 20(r)-21,24-cyclo-3beta,25-dihydroxyl-dammar-23(24)-en-21-one | Corticosteroid binding globulin | TCMID |
| Astragalus mongholicus | MOL21125454 | 20(r)-21,24-cyclo-3beta,25-dihydroxyl-dammar-23(24)-en-21-one | Testis-specific androgen-binding protein | TCMID |
| Astragalus mongholicus | MOL21125454 | 20(r)-21,24-cyclo-3beta,25-dihydroxyl-dammar-23(24)-en-21-one | Protein-tyrosine phosphatase 1B | TCMID |
| Astragalus mongholicus | MOL21125454 | 20(r)-21,24-cyclo-3beta,25-dihydroxyl-dammar-23(24)-en-21-one | Androgen Receptor | TCMID |
| Astragalus mongholicus | MOL21125454 | 20(r)-21,24-cyclo-3beta,25-dihydroxyl-dammar-23(24)-en-21-one | Progesterone receptor | TCMID |
| Astragalus mongholicus | MOL5318035 | 20-hexadecanoylingenol | Protein kinase C delta | TCMID |
| Astragalus mongholicus | MOL5318035 | 20-hexadecanoylingenol | Phosphodiesterase 4D | TCMID |
| Astragalus mongholicus | MOL5318035 | 20-hexadecanoylingenol | Subtilisin/kexin type 7 | TCMID |
| Astragalus mongholicus | MOL5318035 | 20-hexadecanoylingenol | Protein kinase C alpha | TCMID |
| Astragalus mongholicus | MOL5318035 | 20-hexadecanoylingenol | HMG-CoA reductase (by homology) | TCMID |
| Astragalus mongholicus | MOL5318035 | 20-hexadecanoylingenol | Interleukin-8 receptor A | TCMID |
| Astragalus mongholicus | MOL5318035 | 20-hexadecanoylingenol | Interleukin-1 beta | TCMID |
| Astragalus mongholicus | MOL5318035 | 20-hexadecanoylingenol | Glutathione S-transferase Mu 1 | TCMID |
| Astragalus mongholicus | MOL5318035 | 20-hexadecanoylingenol | Proto-oncogene vav | TCMID |
| Astragalus mongholicus | MOL5318035 | 20-hexadecanoylingenol | Sarcoplasmic/endoplasmic reticulum calcium ATPase 1 | TCMID |
| Astragalus mongholicus | MOL5316874 | 3,5-dimethoxystilbene | Quinone reductase 2 | TCMID |
| Astragalus mongholicus | MOL5316874 | 3,5-dimethoxystilbene | Cyclooxygenase-1 | TCMID |
| Astragalus mongholicus | MOL5316874 | 3,5-dimethoxystilbene | Cytochrome P450 1B1 | TCMID |
| Astragalus mongholicus | MOL5316874 | 3,5-dimethoxystilbene | Cyclooxygenase-2 | TCMID |
| Astragalus mongholicus | MOL5316874 | 3,5-dimethoxystilbene | Tubulin beta-1 chain | TCMID |
| Astragalus mongholicus | MOL5316874 | 3,5-dimethoxystilbene | Estrogen receptor alpha | TCMID |
| Astragalus mongholicus | MOL5316874 | 3,5-dimethoxystilbene | Aryl hydrocarbon receptor | TCMID |
| Astragalus mongholicus | MOL5316874 | 3,5-dimethoxystilbene | P-glycoprotein 1 | TCMID |
| Astragalus mongholicus | MOL5316874 | 3,5-dimethoxystilbene | Cytochrome P450 19A1 | TCMID |
| Astragalus mongholicus | MOL5316874 | 3,5-dimethoxystilbene | Cytochrome P450 1A1 | TCMID |
| Astragalus mongholicus | MOL125142 | 6-dimethoxy-isoflavane | Tyrosinase | TCMID |
| Astragalus mongholicus | MOL125142 | 6-dimethoxy-isoflavane | Sodium/glucose cotransporter 2 | TCMID |
| Astragalus mongholicus | MOL125142 | 6-dimethoxy-isoflavane | Sodium/glucose cotransporter 1 | TCMID |
| Astragalus mongholicus | MOL125142 | 6-dimethoxy-isoflavane | Equilibrative nucleoside transporter 1 | TCMID |
| Astragalus mongholicus | MOL125142 | 6-dimethoxy-isoflavane | Adenosine A1 receptor | TCMID |
| Astragalus mongholicus | MOL125142 | 6-dimethoxy-isoflavane | Adenosine A2a receptor | TCMID |
| Astragalus mongholicus | MOL125142 | 6-dimethoxy-isoflavane | Adenosine A3 receptor | TCMID |
| Astragalus mongholicus | MOL125142 | 6-dimethoxy-isoflavane | Cyclooxygenase-1 | TCMID |
| Astragalus mongholicus | MOL125142 | 6-dimethoxy-isoflavane | Matrix metalloproteinase 8 | TCMID |
| Astragalus mongholicus | MOL125142 | 6-dimethoxy-isoflavane | Glucose transporter (by homology) | TCMID |
| Astragalus mongholicus | MOL29927927 | acetic acid | Carbonic anhydrase II | TCMID |
| Astragalus mongholicus | MOL29927927 | acetic acid | Carbonic anhydrase I | TCMID |
| Astragalus mongholicus | MOL29927927 | acetic acid | Monocarboxylate transporter 1 (by homology) | TCMID |
| Astragalus mongholicus | MOL29927927 | acetic acid | Neuronal acetylcholine receptor protein alpha-7 subunit | TCMID |
| Astragalus mongholicus | MOL29927927 | acetic acid | Aryl hydrocarbon receptor | TCMID |
| Astragalus mongholicus | MOL29927927 | acetic acid | Monoamine oxidase B | TCMID |
| Astragalus mongholicus | MOL29927927 | acetic acid | Carbonic anhydrase VII | TCMID |
| Astragalus mongholicus | MOL29927927 | acetic acid | Carbonic anhydrase XII | TCMID |
| Astragalus mongholicus | MOL29927927 | acetic acid | Carbonic anhydrase XIV | TCMID |
| Astragalus mongholicus | MOL29927927 | acetic acid | Carbonic anhydrase IX | TCMID |
| Astragalus mongholicus | MOL60961 | adeninenucleoside | Dipeptidyl peptidase IV | TCMID |
| Astragalus mongholicus | MOL60961 | adeninenucleoside | Adenosine A1 receptor | TCMID |
| Astragalus mongholicus | MOL60961 | adeninenucleoside | Adenosine A2a receptor | TCMID |
| Astragalus mongholicus | MOL60961 | adeninenucleoside | Adenosine A3 receptor | TCMID |
| Astragalus mongholicus | MOL60961 | adeninenucleoside | Adenosine kinase | TCMID |
| Astragalus mongholicus | MOL60961 | adeninenucleoside | Heat shock cognate 71 kDa protein | TCMID |
| Astragalus mongholicus | MOL60961 | adeninenucleoside | 78 kDa glucose-regulated protein | TCMID |
| Astragalus mongholicus | MOL60961 | adeninenucleoside | Adenosine deaminase | TCMID |
| Astragalus mongholicus | MOL60961 | adeninenucleoside | Glyceraldehyde-3-phosphate dehydrogenase liver | TCMID |
| Astragalus mongholicus | MOL60961 | adeninenucleoside | Adenosylhomocysteinase | TCMID |
| Astragalus mongholicus | MOL53486374 | cycloastragenol | Beta-glucocerebrosidase | TCMID |
| Astragalus mongholicus | MOL53486374 | cycloastragenol | Protein farnesyltransferase | TCMID |
| Astragalus mongholicus | MOL53486374 | cycloastragenol | Gamma-secretase | TCMID |
| Astragalus mongholicus | MOL53486374 | cycloastragenol | Vitamin D-binding protein | TCMID |
| Astragalus mongholicus | MOL53486374 | cycloastragenol | C-C chemokine receptor type 1 | TCMID |
| Astragalus mongholicus | MOL53486374 | cycloastragenol | Anandamide amidohydrolase | TCMID |
| Astragalus mongholicus | MOL53486374 | cycloastragenol | Epoxide hydratase | TCMID |
| Astragalus mongholicus | MOL53486374 | cycloastragenol | Proteinase-activated receptor 1 | TCMID |
| Astragalus mongholicus | MOL53486374 | cycloastragenol | Delta opioid receptor (by homology) | TCMID |
| Astragalus mongholicus | MOL53486374 | cycloastragenol | Carbonic anhydrase II | TCMID |
| Astragalus mongholicus | MOL13943286 | cyclosieversigenin | Beta-glucocerebrosidase | TCMID |
| Astragalus mongholicus | MOL13943286 | cyclosieversigenin | Protein farnesyltransferase | TCMID |
| Astragalus mongholicus | MOL13943286 | cyclosieversigenin | Gamma-secretase | TCMID |
| Astragalus mongholicus | MOL13943286 | cyclosieversigenin | Vitamin D-binding protein | TCMID |
| Astragalus mongholicus | MOL13943286 | cyclosieversigenin | C-C chemokine receptor type 1 | TCMID |
| Astragalus mongholicus | MOL13943286 | cyclosieversigenin | Anandamide amidohydrolase | TCMID |
| Astragalus mongholicus | MOL13943286 | cyclosieversigenin | Epoxide hydratase | TCMID |
| Astragalus mongholicus | MOL13943286 | cyclosieversigenin | Proteinase-activated receptor 1 | TCMID |
| Astragalus mongholicus | MOL13943286 | cyclosieversigenin | Delta opioid receptor (by homology) | TCMID |
| Astragalus mongholicus | MOL13943286 | cyclosieversigenin | Carbonic anhydrase II | TCMID |
| Astragalus mongholicus | MOL6037 | folicacid | Thymidylate synthase | TCMID |
| Astragalus mongholicus | MOL6037 | folicacid | Dihydrofolate reductase | TCMID |
| Astragalus mongholicus | MOL6037 | folicacid | Folate transporter 1 | TCMID |
| Astragalus mongholicus | MOL6037 | folicacid | Folate receptor alpha | TCMID |
| Astragalus mongholicus | MOL6037 | folicacid | Folate receptor beta | TCMID |
| Astragalus mongholicus | MOL6037 | folicacid | Histone deacetylase 6 | TCMID |
| Astragalus mongholicus | MOL6037 | folicacid | Proton-coupled folate transporter | TCMID |
| Astragalus mongholicus | MOL6037 | folicacid | Histone deacetylase 8 | TCMID |
| Astragalus mongholicus | MOL6037 | folicacid | Histone deacetylase 1 | TCMID |
| Astragalus mongholicus | MOL6037 | folicacid | Folylpoly-gamma-glutamate synthetase | TCMID |
| Astragalus mongholicus | MOL636741 | gamma-sitosterol | HMG-CoA reductase | TCMID |
| Astragalus mongholicus | MOL636741 | gamma-sitosterol | Cytochrome P450 51 (by homology) | TCMID |
| Astragalus mongholicus | MOL636741 | gamma-sitosterol | Androgen Receptor | TCMID |
| Astragalus mongholicus | MOL636741 | gamma-sitosterol | Niemann-Pick C1-like protein 1 | TCMID |
| Astragalus mongholicus | MOL636741 | gamma-sitosterol | LXR-alpha | TCMID |
| Astragalus mongholicus | MOL636741 | gamma-sitosterol | Cytochrome P450 17A1 | TCMID |
| Astragalus mongholicus | MOL636741 | gamma-sitosterol | Nuclear receptor ROR-gamma | TCMID |
| Astragalus mongholicus | MOL636741 | gamma-sitosterol | Cytochrome P450 19A1 | TCMID |
| Astragalus mongholicus | MOL636741 | gamma-sitosterol | Estrogen receptor beta | TCMID |
| Astragalus mongholicus | MOL636741 | gamma-sitosterol | Estrogen receptor alpha | TCMID |
| Astragalus mongholicus | MOL520159 | hexadecanoicacid | Carbonic anhydrase II | TCMID |
| Astragalus mongholicus | MOL520159 | hexadecanoicacid | Carbonic anhydrase I | TCMID |
| Astragalus mongholicus | MOL520159 | hexadecanoicacid | Estradiol 17-beta-dehydrogenase 3 | TCMID |
| Astragalus mongholicus | MOL520159 | hexadecanoicacid | Fatty acid binding protein adipocyte | TCMID |
| Astragalus mongholicus | MOL520159 | hexadecanoicacid | Fatty acid binding protein muscle | TCMID |
| Astragalus mongholicus | MOL520159 | hexadecanoicacid | Fatty acid binding protein epidermal | TCMID |
| Astragalus mongholicus | MOL520159 | hexadecanoicacid | Fatty acid binding protein intestinal | TCMID |
| Astragalus mongholicus | MOL520159 | hexadecanoicacid | 11-beta-hydroxysteroid dehydrogenase 1 | TCMID |
| Astragalus mongholicus | MOL520159 | hexadecanoicacid | Free fatty acid receptor 1 | TCMID |
| Astragalus mongholicus | MOL520159 | hexadecanoicacid | Peroxisome proliferator-activated receptor delta | TCMID |
| Astragalus mongholicus | MOL60148697 | isoastragaloside i | Gamma-secretase | TCMID |
| Astragalus mongholicus | MOL60148697 | isoastragaloside i | Vascular endothelial growth factor A | TCMID |
| Astragalus mongholicus | MOL60148697 | isoastragaloside i | Acidic fibroblast growth factor | TCMID |
| Astragalus mongholicus | MOL60148697 | isoastragaloside i | Basic fibroblast growth factor | TCMID |
| Astragalus mongholicus | MOL60148697 | isoastragaloside i | Heparanase | TCMID |
| Astragalus mongholicus | MOL60148697 | isoastragaloside i | Nuclear receptor ROR-gamma | TCMID |
| Astragalus mongholicus | MOL60148697 | isoastragaloside i | Glycine receptor subunit alpha-1 | TCMID |
| Astragalus mongholicus | MOL60148697 | isoastragaloside i | Glycine receptor subunit alpha-2 | TCMID |
| Astragalus mongholicus | MOL60148697 | isoastragaloside i | Serotonin 2b (5-HT2b) receptor | TCMID |
| Astragalus mongholicus | MOL60148697 | isoastragaloside i | Alpha-2a adrenergic receptor | TCMID |
| Astragalus mongholicus | MOL60148655 | isoastragaloside ii | Gamma-secretase | TCMID |
| Astragalus mongholicus | MOL60148655 | isoastragaloside ii | Heat shock protein HSP 90-alpha | TCMID |
| Astragalus mongholicus | MOL60148655 | isoastragaloside ii | Vascular endothelial growth factor A | TCMID |
| Astragalus mongholicus | MOL60148655 | isoastragaloside ii | Acidic fibroblast growth factor | TCMID |
| Astragalus mongholicus | MOL60148655 | isoastragaloside ii | Basic fibroblast growth factor | TCMID |
| Astragalus mongholicus | MOL60148655 | isoastragaloside ii | Heparanase | TCMID |
| Astragalus mongholicus | MOL60148655 | isoastragaloside ii | Serotonin 2b (5-HT2b) receptor | TCMID |
| Astragalus mongholicus | MOL60148655 | isoastragaloside ii | Alpha-2a adrenergic receptor | TCMID |
| Astragalus mongholicus | MOL60148655 | isoastragaloside ii | Adrenergic receptor alpha-2 | TCMID |
| Astragalus mongholicus | MOL60148655 | isoastragaloside ii | Alpha-2b adrenergic receptor | TCMID |
| Astragalus mongholicus | MOL3 | isoastragaloside1, 3 | Lysine-specific demethylase 4D-like | TCMID |
| Astragalus mongholicus | MOL3 | isoastragaloside1, 3 | Lysine-specific demethylase 2A | TCMID |
| Astragalus mongholicus | MOL3 | isoastragaloside1, 3 | Lysine-specific demethylase 6B | TCMID |
| Astragalus mongholicus | MOL3 | isoastragaloside1, 3 | Histone lysine demethylase PHF8 | TCMID |
| Astragalus mongholicus | MOL3 | isoastragaloside1, 3 | Lysine-specific demethylase 5C | TCMID |
| Astragalus mongholicus | MOL3 | isoastragaloside1, 3 | Acetylcholinesterase | TCMID |
| Astragalus mongholicus | MOL3 | isoastragaloside1, 3 | Beta-secretase 1 | TCMID |
| Astragalus mongholicus | MOL3 | isoastragaloside1, 3 | Estrogen receptor beta | TCMID |
| Astragalus mongholicus | MOL3 | isoastragaloside1, 3 | Carbonic anhydrase I | TCMID |
| Astragalus mongholicus | MOL3 | isoastragaloside1, 3 | Carbonic anhydrase IX | TCMID |
| Astragalus mongholicus | MOL5318869 | kumatakenin | Aldose reductase (by homology) | TCMID |
| Astragalus mongholicus | MOL5318869 | kumatakenin | Adenosine A1 receptor (by homology) | TCMID |
| Astragalus mongholicus | MOL5318869 | kumatakenin | Adenosine A2a receptor (by homology) | TCMID |
| Astragalus mongholicus | MOL5318869 | kumatakenin | NADPH oxidase 4 | TCMID |
| Astragalus mongholicus | MOL5318869 | kumatakenin | P-glycoprotein 1 | TCMID |
| Astragalus mongholicus | MOL5318869 | kumatakenin | Induced myeloid leukemia cell differentiation protein Mcl-1 | TCMID |
| Astragalus mongholicus | MOL5318869 | kumatakenin | ATP-binding cassette sub-family G member 2 | TCMID |
| Astragalus mongholicus | MOL5318869 | kumatakenin | Estrogen receptor beta | TCMID |
| Astragalus mongholicus | MOL5318869 | kumatakenin | Solute carrier family 22 member 12 | TCMID |
| Astragalus mongholicus | MOL5318869 | kumatakenin | Delta opioid receptor | TCMID |
| Astragalus mongholicus | MOL5317378 | kumugansine a | Monoamine oxidase A | TCMID |
| Astragalus mongholicus | MOL5317378 | kumugansine a | Cathepsin K | TCMID |
| Astragalus mongholicus | MOL5317378 | kumugansine a | Cathepsin S | TCMID |
| Astragalus mongholicus | MOL5317378 | kumugansine a | Cathepsin L | TCMID |
| Astragalus mongholicus | MOL5317378 | kumugansine a | Glycogen synthase kinase-3 beta | TCMID |
| Astragalus mongholicus | MOL5317378 | kumugansine a | Myeloperoxidase | TCMID |
| Astragalus mongholicus | MOL5317378 | kumugansine a | Serine/threonine-protein kinase PIM3 | TCMID |
| Astragalus mongholicus | MOL5317378 | kumugansine a | Thymidine phosphorylase | TCMID |
| Astragalus mongholicus | MOL5317378 | kumugansine a | CDGSH iron-sulfur domain-containing protein 1 | TCMID |
| Astragalus mongholicus | MOL5317378 | kumugansine a | c-Jun N-terminal kinase 1 | TCMID |
| Astragalus mongholicus | MOL23135 | n-candicine | Neuromedin-U receptor 2 | TCMID |
| Astragalus mongholicus | MOL23135 | n-candicine | Trace amine-associated receptor 1 (by homology) | TCMID |
| Astragalus mongholicus | MOL23135 | n-candicine | Dopamine D2 receptor (by homology) | TCMID |
| Astragalus mongholicus | MOL23135 | n-candicine | Lysine-specific demethylase 4D-like | TCMID |
| Astragalus mongholicus | MOL23135 | n-candicine | Norepinephrine transporter | TCMID |
| Astragalus mongholicus | MOL23135 | n-candicine | Melatonin receptor 1A | TCMID |
| Astragalus mongholicus | MOL23135 | n-candicine | Amine oxidase, copper containing | TCMID |
| Astragalus mongholicus | MOL23135 | n-candicine | Serotonin 3a (5-HT3a) receptor | TCMID |
| Astragalus mongholicus | MOL23135 | n-candicine | Sigma opioid receptor | TCMID |
| Astragalus mongholicus | MOL23135 | n-candicine | Mu opioid receptor | TCMID |
| Astragalus mongholicus | MOL44246636 | soyasapogenol b | Protein-tyrosine phosphatase 1B | TCMID |
| Astragalus mongholicus | MOL44246636 | soyasapogenol b | Androgen Receptor | TCMID |
| Astragalus mongholicus | MOL44246636 | soyasapogenol b | Cytochrome P450 2C19 | TCMID |
| Astragalus mongholicus | MOL44246636 | soyasapogenol b | Cytochrome P450 19A1 | TCMID |
| Astragalus mongholicus | MOL44246636 | soyasapogenol b | Nuclear receptor subfamily 1 group I member 3 (by homology) | TCMID |
| Astragalus mongholicus | MOL44246636 | soyasapogenol b | Estrogen receptor alpha | TCMID |
| Astragalus mongholicus | MOL44246636 | soyasapogenol b | Butyrylcholinesterase | TCMID |
| Astragalus mongholicus | MOL44246636 | soyasapogenol b | LXR-alpha | TCMID |
| Astragalus mongholicus | MOL44246636 | soyasapogenol b | Muscarinic acetylcholine receptor M2 | TCMID |
| Astragalus mongholicus | MOL53301851 | sucrose | Cyclin-dependent kinase 1 | TCMID |
| Astragalus mongholicus | MOL53301851 | sucrose | Heat shock protein HSP 90-alpha | TCMID |
| Astragalus mongholicus | MOL53301851 | sucrose | Vascular endothelial growth factor A | TCMID |
| Astragalus mongholicus | MOL53301851 | sucrose | Gamma-secretase | TCMID |
| Astragalus mongholicus | MOL53301851 | sucrose | Acidic fibroblast growth factor | TCMID |
| Astragalus mongholicus | MOL53301851 | sucrose | Heparanase | TCMID |
| Astragalus mongholicus | MOL53301851 | sucrose | Basic fibroblast growth factor | TCMID |
| Astragalus mongholicus | MOL53301851 | sucrose | Galectin-4 | TCMID |
| Astragalus mongholicus | MOL53301851 | sucrose | Galectin-3 | TCMID |
| Astragalus mongholicus | MOL53301851 | sucrose | Galectin-8 | TCMID |
| Astragalus mongholicus | MOL9986231 | suffruticoside a | Tyrosyl-DNA phosphodiesterase 1 | TCMID |
| Astragalus mongholicus | MOL9986231 | suffruticoside a | Epoxide hydratase | TCMID |
| Astragalus mongholicus | MOL9986231 | suffruticoside a | Cytochrome P450 19A1 | TCMID |
| Astragalus mongholicus | MOL9986231 | suffruticoside a | Low affinity sodium-glucose cotransporter | TCMID |
| Astragalus mongholicus | MOL9986231 | suffruticoside a | Solute carrier family 28 member 3 | TCMID |
| Astragalus mongholicus | MOL9986231 | suffruticoside a | Sodium/glucose cotransporter 2 | TCMID |
| Astragalus mongholicus | MOL9986231 | suffruticoside a | Estrogen receptor alpha | TCMID |
| Astragalus mongholicus | MOL9986231 | suffruticoside a | Plasminogen activator inhibitor-1 | TCMID |
| Astragalus mongholicus | MOL9986231 | suffruticoside a | T-cell protein-tyrosine phosphatase | TCMID |
| Astragalus mongholicus | MOL9986231 | suffruticoside a | Squalene monooxygenase (by homology) | TCMID |
| Astragalus mongholicus | MOL45356795 | uridine | Cytidine deaminase | TCMID |
| Astragalus mongholicus | MOL45356795 | uridine | Adenosine deaminase | TCMID |
| Astragalus mongholicus | MOL45356795 | uridine | Muscle glycogen phosphorylase | TCMID |
| Astragalus mongholicus | MOL45356795 | uridine | Thymidine kinase, cytosolic | TCMID |
| Astragalus mongholicus | MOL45356795 | uridine | Thymidylate synthase (by homology) | TCMID |
| Astragalus mongholicus | MOL45356795 | uridine | Adenosine A1 receptor | TCMID |
| Astragalus mongholicus | MOL45356795 | uridine | Thymidine kinase, mitochondrial (by homology) | TCMID |
| Astragalus mongholicus | MOL45356795 | uridine | Pyrimidinergic receptor P2Y6 | TCMID |
| Astragalus mongholicus | MOL45356795 | uridine | UDP-N-acetylglucosamine--peptide N-acetylglucosaminyltransferase 110 kDa subunit | TCMID |
| Astragalus mongholicus | MOL45356795 | uridine | Carbonic anhydrase XII | TCMID |
| Astragalus mongholicus | MOL222284 | β-sitosterol | HMG-CoA reductase | TCMID |
| Astragalus mongholicus | MOL222284 | β-sitosterol | Cytochrome P450 51 (by homology) | TCMID |
| Astragalus mongholicus | MOL222284 | β-sitosterol | Androgen Receptor | TCMID |
| Astragalus mongholicus | MOL222284 | β-sitosterol | Niemann-Pick C1-like protein 1 | TCMID |
| Astragalus mongholicus | MOL222284 | β-sitosterol | LXR-alpha | TCMID |
| Astragalus mongholicus | MOL222284 | β-sitosterol | Cytochrome P450 17A1 | TCMID |
| Astragalus mongholicus | MOL222284 | β-sitosterol | Nuclear receptor ROR-gamma | TCMID |
| Astragalus mongholicus | MOL222284 | β-sitosterol | Cytochrome P450 19A1 | TCMID |
| Astragalus mongholicus | MOL222284 | β-sitosterol | Estrogen receptor beta | TCMID |
| Astragalus mongholicus | MOL222284 | β-sitosterol | Estrogen receptor alpha | TCMID |

**Supplementary table 3b. CPV of the active ingredients from TCMID database and relevant literature.**

| Drug | MolId | MolName | Symbol | Source |
| --- | --- | --- | --- | --- |
| Curcuma phaeocaulis Valeton | MOL11128245 | curcumenol | Norepinephrine transporter | TCMID |
| Curcuma phaeocaulis Valeton | MOL11128245 | curcumenol | Serotonin transporter | TCMID |
| Curcuma phaeocaulis Valeton | MOL11128245 | curcumenol | Poly [ADP-ribose] polymerase-1 | TCMID |
| Curcuma phaeocaulis Valeton | MOL11128245 | curcumenol | Sigma opioid receptor | TCMID |
| Curcuma phaeocaulis Valeton | MOL11128245 | curcumenol | P2X purinoceptor 7 | TCMID |
| Curcuma phaeocaulis Valeton | MOL11128245 | curcumenol | Transient receptor potential cation channel subfamily V member 3 | TCMID |
| Curcuma phaeocaulis Valeton | MOL11128245 | curcumenol | p53-binding protein Mdm-2 | TCMID |
| Curcuma phaeocaulis Valeton | MOL11128245 | curcumenol | Protein tyrosine kinase 2 beta | TCMID |
| Curcuma phaeocaulis Valeton | MOL11128245 | curcumenol | Fatty acid desaturase 1 | TCMID |
| Curcuma phaeocaulis Valeton | MOL11128245 | curcumenol | Glycogen synthase kinase-3 alpha | TCMID |
| Curcuma phaeocaulis Valeton | MOL44629981 | curcumin | Beta-secretase 1 | TCMID |
| Curcuma phaeocaulis Valeton | MOL44629981 | curcumin | Beta amyloid A4 protein | TCMID |
| Curcuma phaeocaulis Valeton | MOL44629981 | curcumin | Monoamine oxidase A | TCMID |
| Curcuma phaeocaulis Valeton | MOL44629981 | curcumin | Histone acetyltransferase p300 | TCMID |
| Curcuma phaeocaulis Valeton | MOL44629981 | curcumin | Prostaglandin E synthase | TCMID |
| Curcuma phaeocaulis Valeton | MOL44629981 | curcumin | Glyoxalase I | TCMID |
| Curcuma phaeocaulis Valeton | MOL44629981 | curcumin | Toll-like receptor (TLR7/TLR9) | TCMID |
| Curcuma phaeocaulis Valeton | MOL44629981 | curcumin | Inhibitor of NF-kappa-B kinase (IKK) | TCMID |
| Curcuma phaeocaulis Valeton | MOL44629981 | curcumin | Serine/threonine-protein kinase AKT | TCMID |
| Curcuma phaeocaulis Valeton | MOL44629981 | curcumin | DNA topoisomerase II alpha | TCMID |
| Curcuma phaeocaulis Valeton | MOL10466651 | curdione | Cytochrome P450 19A1 | TCMID |
| Curcuma phaeocaulis Valeton | MOL10466651 | curdione | Cathepsin D | TCMID |
| Curcuma phaeocaulis Valeton | MOL10466651 | curdione | Cyclooxygenase-1 | TCMID |
| Curcuma phaeocaulis Valeton | MOL10466651 | curdione | C-C chemokine receptor type 5 | TCMID |
| Curcuma phaeocaulis Valeton | MOL10466651 | curdione | Vanilloid receptor | TCMID |
| Curcuma phaeocaulis Valeton | MOL10466651 | curdione | Anandamide amidohydrolase | TCMID |
| Curcuma phaeocaulis Valeton | MOL10466651 | curdione | Melatonin receptor 1A | TCMID |
| Curcuma phaeocaulis Valeton | MOL10466651 | curdione | Melatonin receptor 1B | TCMID |
| Curcuma phaeocaulis Valeton | MOL10466651 | curdione | Cathepsin K | TCMID |
| Curcuma phaeocaulis Valeton | MOL10466651 | curdione | Cathepsin L | TCMID |
| Curcuma phaeocaulis Valeton | MOL5316217 | curzerene | Dopamine D4 receptor | TCMID |
| Curcuma phaeocaulis Valeton | MOL10376566 | curzerenone | Serotonin transporter (by homology) | literature |
| Curcuma phaeocaulis Valeton | MOL10376566 | curzerenone | Dopamine transporter (by homology) | literature |
| Curcuma phaeocaulis Valeton | MOL10376566 | curzerenone | Cyclooxygenase-2 | literature |
| Curcuma phaeocaulis Valeton | MOL10376566 | curzerenone | Polyadenylate-binding protein 1 | literature |
| Curcuma phaeocaulis Valeton | MOL10376566 | curzerenone | Estradiol 17-beta-dehydrogenase 2 | literature |
| Curcuma phaeocaulis Valeton | MOL10376566 | curzerenone | NACHT, LRR and PYD domains-containing protein 3 | literature |
| Curcuma phaeocaulis Valeton | MOL10376566 | curzerenone | Epoxide hydrolase 1 | literature |
| Curcuma phaeocaulis Valeton | MOL10376566 | curzerenone | Prolyl endopeptidase | literature |
| Curcuma phaeocaulis Valeton | MOL10376566 | curzerenone | Cathepsin K | literature |
| Curcuma phaeocaulis Valeton | MOL10376566 | curzerenone | Cathepsin L | literature |
| Curcuma phaeocaulis Valeton | MOL11128245 | epicurcumenol | Norepinephrine transporter | TCMID |
| Curcuma phaeocaulis Valeton | MOL11128245 | epicurcumenol | Serotonin transporter | TCMID |
| Curcuma phaeocaulis Valeton | MOL11128245 | epicurcumenol | Poly [ADP-ribose] polymerase-1 | TCMID |
| Curcuma phaeocaulis Valeton | MOL11128245 | epicurcumenol | Sigma opioid receptor | TCMID |
| Curcuma phaeocaulis Valeton | MOL11128245 | epicurcumenol | P2X purinoceptor 7 | TCMID |
| Curcuma phaeocaulis Valeton | MOL11128245 | epicurcumenol | Transient receptor potential cation channel subfamily V member 3 | TCMID |
| Curcuma phaeocaulis Valeton | MOL11128245 | epicurcumenol | p53-binding protein Mdm-2 | TCMID |
| Curcuma phaeocaulis Valeton | MOL11128245 | epicurcumenol | Protein tyrosine kinase 2 beta | TCMID |
| Curcuma phaeocaulis Valeton | MOL11128245 | epicurcumenol | Fatty acid desaturase 1 | TCMID |
| Curcuma phaeocaulis Valeton | MOL11128245 | epicurcumenol | Glycogen synthase kinase-3 alpha | TCMID |
| Curcuma phaeocaulis Valeton | MOL5317062 | epicurzerenone | Serotonin transporter (by homology) | TCMID |
| Curcuma phaeocaulis Valeton | MOL5317062 | epicurzerenone | Dopamine transporter (by homology) | TCMID |
| Curcuma phaeocaulis Valeton | MOL5317062 | epicurzerenone | Cyclooxygenase-2 | TCMID |
| Curcuma phaeocaulis Valeton | MOL5317062 | epicurzerenone | Polyadenylate-binding protein 1 | TCMID |
| Curcuma phaeocaulis Valeton | MOL5317062 | epicurzerenone | Estradiol 17-beta-dehydrogenase 2 | TCMID |
| Curcuma phaeocaulis Valeton | MOL5317062 | epicurzerenone | NACHT, LRR and PYD domains-containing protein 3 | TCMID |
| Curcuma phaeocaulis Valeton | MOL5317062 | epicurzerenone | Epoxide hydrolase 1 | TCMID |
| Curcuma phaeocaulis Valeton | MOL5317062 | epicurzerenone | Prolyl endopeptidase | TCMID |
| Curcuma phaeocaulis Valeton | MOL5317062 | epicurzerenone | Cathepsin K | TCMID |
| Curcuma phaeocaulis Valeton | MOL5317062 | epicurzerenone | Cathepsin L | TCMID |
| Curcuma phaeocaulis Valeton | MOL5316216 | neocurdione | Cytochrome P450 19A1 | TCMID |
| Curcuma phaeocaulis Valeton | MOL5316216 | neocurdione | Cathepsin D | TCMID |
| Curcuma phaeocaulis Valeton | MOL5316216 | neocurdione | Cyclooxygenase-1 | TCMID |
| Curcuma phaeocaulis Valeton | MOL5316216 | neocurdione | C-C chemokine receptor type 5 | TCMID |
| Curcuma phaeocaulis Valeton | MOL5316216 | neocurdione | Vanilloid receptor | TCMID |
| Curcuma phaeocaulis Valeton | MOL5316216 | neocurdione | Anandamide amidohydrolase | TCMID |
| Curcuma phaeocaulis Valeton | MOL5316216 | neocurdione | Melatonin receptor 1A | TCMID |
| Curcuma phaeocaulis Valeton | MOL5316216 | neocurdione | Melatonin receptor 1B | TCMID |
| Curcuma phaeocaulis Valeton | MOL5316216 | neocurdione | Cathepsin K | TCMID |
| Curcuma phaeocaulis Valeton | MOL5316216 | neocurdione | Cathepsin L | TCMID |
| Curcuma phaeocaulis Valeton | MOL10263440 | procurcumenol | Glucocorticoid receptor | TCMID |
| Curcuma phaeocaulis Valeton | MOL10263440 | procurcumenol | Androgen Receptor | TCMID |
| Curcuma phaeocaulis Valeton | MOL10263440 | procurcumenol | Mineralocorticoid receptor | TCMID |
| Curcuma phaeocaulis Valeton | MOL10263440 | procurcumenol | Cytochrome P450 19A1 | TCMID |
| Curcuma phaeocaulis Valeton | MOL10263440 | procurcumenol | 11-beta-hydroxysteroid dehydrogenase 2 | TCMID |
| Curcuma phaeocaulis Valeton | MOL10263440 | procurcumenol | 11-beta-hydroxysteroid dehydrogenase 1 | TCMID |
| Curcuma phaeocaulis Valeton | MOL10263440 | procurcumenol | Interleukin-6 | TCMID |
| Curcuma phaeocaulis Valeton | MOL10263440 | procurcumenol | Glutamine synthetase | TCMID |
| Curcuma phaeocaulis Valeton | MOL10263440 | procurcumenol | G-protein coupled bile acid receptor 1 | TCMID |
| Curcuma phaeocaulis Valeton | MOL10263440 | procurcumenol | Fatty acid-binding protein, liver (by homology) | TCMID |

# Supplementary 4

## **The active ingredients from ETCM database and relevant literature**

**Supplementary table 4a. AM of the active ingredients from ETCM database and relevant literature.**

| Drug | MolId | MolName | Symbol |
| --- | --- | --- | --- |
| Astragalus mongholicus | MOL500 | (Trans-Trans)Farnesol,3,7,11-Trimethyldodeca-2,6,10-Trien-1-Ol,Farnesol | MAOB |
| Astragalus mongholicus | MOL501 | (Trans-Trans)Farnesol,3,7,11-Trimethyldodeca-2,6,10-Trien-1-Ol,Farnesol | NR1H4 |
| Astragalus mongholicus | MOL502 | 1,7,7-Trimethylbicyclo[2.2.1]Heptan-2-Ol,Borneol | AKR1D1 |
| Astragalus mongholicus | MOL503 | 1,7,7-Trimethylbicyclo[2.2.1]Heptan-2-Ol,Borneol | AR |
| Astragalus mongholicus | MOL504 | 1,7,7-Trimethylbicyclo[2.2.1]Heptan-2-Ol,Borneol | CACNA1C |
| Astragalus mongholicus | MOL505 | 1,7,7-Trimethylbicyclo[2.2.1]Heptan-2-Ol,Borneol | CACNA1D |
| Astragalus mongholicus | MOL506 | 1,7,7-Trimethylbicyclo[2.2.1]Heptan-2-Ol,Borneol | CACNA1F |
| Astragalus mongholicus | MOL507 | 1,7,7-Trimethylbicyclo[2.2.1]Heptan-2-Ol,Borneol | CACNA1S |
| Astragalus mongholicus | MOL508 | 1,7,7-Trimethylbicyclo[2.2.1]Heptan-2-Ol,Borneol | CACNB1 |
| Astragalus mongholicus | MOL509 | 1,7,7-Trimethylbicyclo[2.2.1]Heptan-2-Ol,Borneol | CACNB2 |
| Astragalus mongholicus | MOL510 | 1,7,7-Trimethylbicyclo[2.2.1]Heptan-2-Ol,Borneol | CACNB3 |
| Astragalus mongholicus | MOL511 | 1,7,7-Trimethylbicyclo[2.2.1]Heptan-2-Ol,Borneol | CACNB4 |
| Astragalus mongholicus | MOL512 | 1,7,7-Trimethylbicyclo[2.2.1]Heptan-2-Ol,Borneol | ESR1 |
| Astragalus mongholicus | MOL513 | 1,7,7-Trimethylbicyclo[2.2.1]Heptan-2-Ol,Borneol | ESR2 |
| Astragalus mongholicus | MOL514 | 1,7,7-Trimethylbicyclo[2.2.1]Heptan-2-Ol,Borneol | GABRA1 |
| Astragalus mongholicus | MOL515 | 1,7,7-Trimethylbicyclo[2.2.1]Heptan-2-Ol,Borneol | GABRA2 |
| Astragalus mongholicus | MOL516 | 1,7,7-Trimethylbicyclo[2.2.1]Heptan-2-Ol,Borneol | GABRA3 |
| Astragalus mongholicus | MOL517 | 1,7,7-Trimethylbicyclo[2.2.1]Heptan-2-Ol,Borneol | GABRA4 |
| Astragalus mongholicus | MOL518 | 1,7,7-Trimethylbicyclo[2.2.1]Heptan-2-Ol,Borneol | GABRA5 |
| Astragalus mongholicus | MOL519 | 1,7,7-Trimethylbicyclo[2.2.1]Heptan-2-Ol,Borneol | GABRA6 |
| Astragalus mongholicus | MOL520 | 1,7,7-Trimethylbicyclo[2.2.1]Heptan-2-Ol,Borneol | GABRB1 |
| Astragalus mongholicus | MOL521 | 1,7,7-Trimethylbicyclo[2.2.1]Heptan-2-Ol,Borneol | GABRB2 |
| Astragalus mongholicus | MOL522 | 1,7,7-Trimethylbicyclo[2.2.1]Heptan-2-Ol,Borneol | GABRB3 |
| Astragalus mongholicus | MOL523 | 1,7,7-Trimethylbicyclo[2.2.1]Heptan-2-Ol,Borneol | GABRD |
| Astragalus mongholicus | MOL524 | 1,7,7-Trimethylbicyclo[2.2.1]Heptan-2-Ol,Borneol | GABRE |
| Astragalus mongholicus | MOL525 | 1,7,7-Trimethylbicyclo[2.2.1]Heptan-2-Ol,Borneol | GABRG1 |
| Astragalus mongholicus | MOL526 | 1,7,7-Trimethylbicyclo[2.2.1]Heptan-2-Ol,Borneol | GABRG2 |
| Astragalus mongholicus | MOL527 | 1,7,7-Trimethylbicyclo[2.2.1]Heptan-2-Ol,Borneol | GABRG3 |
| Astragalus mongholicus | MOL528 | 1,7,7-Trimethylbicyclo[2.2.1]Heptan-2-Ol,Borneol | GABRP |
| Astragalus mongholicus | MOL529 | 1,7,7-Trimethylbicyclo[2.2.1]Heptan-2-Ol,Borneol | GABRQ |
| Astragalus mongholicus | MOL530 | 1,7,7-Trimethylbicyclo[2.2.1]Heptan-2-Ol,Borneol | HSD17B1 |
| Astragalus mongholicus | MOL531 | 1,7,7-Trimethylbicyclo[2.2.1]Heptan-2-Ol,Borneol | HSD17B11 |
| Astragalus mongholicus | MOL532 | 1,7,7-Trimethylbicyclo[2.2.1]Heptan-2-Ol,Borneol | IGHG2 |
| Astragalus mongholicus | MOL533 | 1,7,7-Trimethylbicyclo[2.2.1]Heptan-2-Ol,Borneol | LSS |
| Astragalus mongholicus | MOL534 | 1,7,7-Trimethylbicyclo[2.2.1]Heptan-2-Ol,Borneol | NR1I3 |
| Astragalus mongholicus | MOL535 | 1,7,7-Trimethylbicyclo[2.2.1]Heptan-2-Ol,Borneol | NR3C2 |
| Astragalus mongholicus | MOL536 | 1,7,7-Trimethylbicyclo[2.2.1]Heptan-2-Ol,Borneol | OPRK1 |
| Astragalus mongholicus | MOL537 | 1,7,7-Trimethylbicyclo[2.2.1]Heptan-2-Ol,Borneol | SULT2A1 |
| Astragalus mongholicus | MOL538 | 1,7,7-Trimethylbicyclo[2.2.1]Heptan-2-Ol,Borneol | TRPA1 |
| Astragalus mongholicus | MOL539 | 1,7,7-Trimethylbicyclo[2.2.1]Heptan-2-Ol,Borneol | TRPM8 |
| Astragalus mongholicus | MOL540 | 1,7,7-Trimethylbicyclo[2.2.1]Heptan-2-Ol,Borneol | TRPV3 |
| Astragalus mongholicus | MOL541 | 1,7,7-Trimethylbicyclo[2.2.1]Heptan-2-Ol,Borneol | VDR |
| Astragalus mongholicus | MOL542 | Camphor | AKR1D1 |
| Astragalus mongholicus | MOL543 | Camphor | NCOA1 |
| Astragalus mongholicus | MOL544 | Camphor | NR1I3 |
| Astragalus mongholicus | MOL545 | Camphor | RXRA |
| Astragalus mongholicus | MOL546 | Camphor | TRPA1 |
| Astragalus mongholicus | MOL547 | Camphor | TRPM8 |
| Astragalus mongholicus | MOL548 | Camphor | TRPV1 |
| Astragalus mongholicus | MOL549 | Camphor | TRPV3 |
| Astragalus mongholicus | MOL550 | 1-Isopropyl-4-Methylcyclohex-3-Enol,4-Terpineol | VDR |
| Astragalus mongholicus | MOL551 | (1R,2R,4R)-1,7,7-Trimethylbicyclo[2.2.1]Heptan-2-Ol,Isoborneol,L-Isoborneol | AKR1D1 |
| Astragalus mongholicus | MOL552 | (1R,2R,4R)-1,7,7-Trimethylbicyclo[2.2.1]Heptan-2-Ol,Isoborneol,L-Isoborneol | AR |
| Astragalus mongholicus | MOL553 | (1R,2R,4R)-1,7,7-Trimethylbicyclo[2.2.1]Heptan-2-Ol,Isoborneol,L-Isoborneol | CACNA1C |
| Astragalus mongholicus | MOL554 | (1R,2R,4R)-1,7,7-Trimethylbicyclo[2.2.1]Heptan-2-Ol,Isoborneol,L-Isoborneol | CACNA1D |
| Astragalus mongholicus | MOL555 | (1R,2R,4R)-1,7,7-Trimethylbicyclo[2.2.1]Heptan-2-Ol,Isoborneol,L-Isoborneol | CACNA1F |
| Astragalus mongholicus | MOL556 | (1R,2R,4R)-1,7,7-Trimethylbicyclo[2.2.1]Heptan-2-Ol,Isoborneol,L-Isoborneol | CACNA1S |
| Astragalus mongholicus | MOL557 | (1R,2R,4R)-1,7,7-Trimethylbicyclo[2.2.1]Heptan-2-Ol,Isoborneol,L-Isoborneol | CACNB1 |
| Astragalus mongholicus | MOL558 | (1R,2R,4R)-1,7,7-Trimethylbicyclo[2.2.1]Heptan-2-Ol,Isoborneol,L-Isoborneol | CACNB2 |
| Astragalus mongholicus | MOL559 | (1R,2R,4R)-1,7,7-Trimethylbicyclo[2.2.1]Heptan-2-Ol,Isoborneol,L-Isoborneol | CACNB3 |
| Astragalus mongholicus | MOL560 | (1R,2R,4R)-1,7,7-Trimethylbicyclo[2.2.1]Heptan-2-Ol,Isoborneol,L-Isoborneol | CACNB4 |
| Astragalus mongholicus | MOL561 | (1R,2R,4R)-1,7,7-Trimethylbicyclo[2.2.1]Heptan-2-Ol,Isoborneol,L-Isoborneol | ESR1 |
| Astragalus mongholicus | MOL562 | (1R,2R,4R)-1,7,7-Trimethylbicyclo[2.2.1]Heptan-2-Ol,Isoborneol,L-Isoborneol | ESR2 |
| Astragalus mongholicus | MOL563 | (1R,2R,4R)-1,7,7-Trimethylbicyclo[2.2.1]Heptan-2-Ol,Isoborneol,L-Isoborneol | GABRA1 |
| Astragalus mongholicus | MOL564 | (1R,2R,4R)-1,7,7-Trimethylbicyclo[2.2.1]Heptan-2-Ol,Isoborneol,L-Isoborneol | GABRA2 |
| Astragalus mongholicus | MOL565 | (1R,2R,4R)-1,7,7-Trimethylbicyclo[2.2.1]Heptan-2-Ol,Isoborneol,L-Isoborneol | GABRA3 |
| Astragalus mongholicus | MOL566 | (1R,2R,4R)-1,7,7-Trimethylbicyclo[2.2.1]Heptan-2-Ol,Isoborneol,L-Isoborneol | GABRA4 |
| Astragalus mongholicus | MOL567 | (1R,2R,4R)-1,7,7-Trimethylbicyclo[2.2.1]Heptan-2-Ol,Isoborneol,L-Isoborneol | GABRA5 |
| Astragalus mongholicus | MOL568 | (1R,2R,4R)-1,7,7-Trimethylbicyclo[2.2.1]Heptan-2-Ol,Isoborneol,L-Isoborneol | GABRA6 |
| Astragalus mongholicus | MOL569 | (1R,2R,4R)-1,7,7-Trimethylbicyclo[2.2.1]Heptan-2-Ol,Isoborneol,L-Isoborneol | GABRB1 |
| Astragalus mongholicus | MOL570 | (1R,2R,4R)-1,7,7-Trimethylbicyclo[2.2.1]Heptan-2-Ol,Isoborneol,L-Isoborneol | GABRB2 |
| Astragalus mongholicus | MOL571 | (1R,2R,4R)-1,7,7-Trimethylbicyclo[2.2.1]Heptan-2-Ol,Isoborneol,L-Isoborneol | GABRB3 |
| Astragalus mongholicus | MOL572 | (1R,2R,4R)-1,7,7-Trimethylbicyclo[2.2.1]Heptan-2-Ol,Isoborneol,L-Isoborneol | GABRD |
| Astragalus mongholicus | MOL573 | (1R,2R,4R)-1,7,7-Trimethylbicyclo[2.2.1]Heptan-2-Ol,Isoborneol,L-Isoborneol | GABRE |
| Astragalus mongholicus | MOL574 | (1R,2R,4R)-1,7,7-Trimethylbicyclo[2.2.1]Heptan-2-Ol,Isoborneol,L-Isoborneol | GABRG1 |
| Astragalus mongholicus | MOL575 | (1R,2R,4R)-1,7,7-Trimethylbicyclo[2.2.1]Heptan-2-Ol,Isoborneol,L-Isoborneol | GABRG2 |
| Astragalus mongholicus | MOL576 | (1R,2R,4R)-1,7,7-Trimethylbicyclo[2.2.1]Heptan-2-Ol,Isoborneol,L-Isoborneol | GABRG3 |
| Astragalus mongholicus | MOL577 | (1R,2R,4R)-1,7,7-Trimethylbicyclo[2.2.1]Heptan-2-Ol,Isoborneol,L-Isoborneol | GABRP |
| Astragalus mongholicus | MOL578 | (1R,2R,4R)-1,7,7-Trimethylbicyclo[2.2.1]Heptan-2-Ol,Isoborneol,L-Isoborneol | GABRQ |
| Astragalus mongholicus | MOL579 | (1R,2R,4R)-1,7,7-Trimethylbicyclo[2.2.1]Heptan-2-Ol,Isoborneol,L-Isoborneol | HSD17B1 |
| Astragalus mongholicus | MOL580 | (1R,2R,4R)-1,7,7-Trimethylbicyclo[2.2.1]Heptan-2-Ol,Isoborneol,L-Isoborneol | HSD17B11 |
| Astragalus mongholicus | MOL581 | (1R,2R,4R)-1,7,7-Trimethylbicyclo[2.2.1]Heptan-2-Ol,Isoborneol,L-Isoborneol | IGHG2 |
| Astragalus mongholicus | MOL582 | (1R,2R,4R)-1,7,7-Trimethylbicyclo[2.2.1]Heptan-2-Ol,Isoborneol,L-Isoborneol | LSS |
| Astragalus mongholicus | MOL583 | (1R,2R,4R)-1,7,7-Trimethylbicyclo[2.2.1]Heptan-2-Ol,Isoborneol,L-Isoborneol | NR1I3 |
| Astragalus mongholicus | MOL584 | (1R,2R,4R)-1,7,7-Trimethylbicyclo[2.2.1]Heptan-2-Ol,Isoborneol,L-Isoborneol | NR3C2 |
| Astragalus mongholicus | MOL585 | (1R,2R,4R)-1,7,7-Trimethylbicyclo[2.2.1]Heptan-2-Ol,Isoborneol,L-Isoborneol | OPRK1 |
| Astragalus mongholicus | MOL586 | (1R,2R,4R)-1,7,7-Trimethylbicyclo[2.2.1]Heptan-2-Ol,Isoborneol,L-Isoborneol | SULT2A1 |
| Astragalus mongholicus | MOL587 | (1R,2R,4R)-1,7,7-Trimethylbicyclo[2.2.1]Heptan-2-Ol,Isoborneol,L-Isoborneol | TRPA1 |
| Astragalus mongholicus | MOL588 | (1R,2R,4R)-1,7,7-Trimethylbicyclo[2.2.1]Heptan-2-Ol,Isoborneol,L-Isoborneol | TRPM8 |
| Astragalus mongholicus | MOL589 | (1R,2R,4R)-1,7,7-Trimethylbicyclo[2.2.1]Heptan-2-Ol,Isoborneol,L-Isoborneol | TRPV3 |
| Astragalus mongholicus | MOL590 | (1R,2R,4R)-1,7,7-Trimethylbicyclo[2.2.1]Heptan-2-Ol,Isoborneol,L-Isoborneol | VDR |
| Astragalus mongholicus | MOL591 | 1,7,7-Trimethylbicyclo[2.2.1]Heptan-2-YlAcetate,BornylAcetate | AR |
| Astragalus mongholicus | MOL592 | 1,7,7-Trimethylbicyclo[2.2.1]Heptan-2-YlAcetate,BornylAcetate | ESR1 |
| Astragalus mongholicus | MOL593 | 1,7,7-Trimethylbicyclo[2.2.1]Heptan-2-YlAcetate,BornylAcetate | HSD17B1 |
| Astragalus mongholicus | MOL594 | 1,7,7-Trimethylbicyclo[2.2.1]Heptan-2-YlAcetate,BornylAcetate | NR3C2 |
| Astragalus mongholicus | MOL595 | 1,7,7-Trimethylbicyclo[2.2.1]Heptan-2-YlAcetate,BornylAcetate | TRPA1 |
| Astragalus mongholicus | MOL596 | 1,7,7-Trimethylbicyclo[2.2.1]Heptan-2-YlAcetate,BornylAcetate | TRPM8 |
| Astragalus mongholicus | MOL597 | 1,7,7-Trimethylbicyclo[2.2.1]Heptan-2-YlAcetate,BornylAcetate | TRPV1 |
| Astragalus mongholicus | MOL598 | 1,7,7-Trimethylbicyclo[2.2.1]Heptan-2-YlAcetate,BornylAcetate | TRPV3 |
| Astragalus mongholicus | MOL599 | (S)-5-Hydroxy-7-Methoxy-2-Phenylchroman-4-One,7-Hydroxy-5-Methoxyflavanone | AKR1C1 |
| Astragalus mongholicus | MOL600 | (S)-5-Hydroxy-7-Methoxy-2-Phenylchroman-4-One,7-Hydroxy-5-Methoxyflavanone | CYP19A1 |
| Astragalus mongholicus | MOL601 | (S)-5-Hydroxy-7-Methoxy-2-Phenylchroman-4-One,7-Hydroxy-5-Methoxyflavanone | CYP1B1 |
| Astragalus mongholicus | MOL602 | (S)-5-Hydroxy-7-Methoxy-2-Phenylchroman-4-One,7-Hydroxy-5-Methoxyflavanone | ESR1 |
| Astragalus mongholicus | MOL603 | (S)-5-Hydroxy-7-Methoxy-2-Phenylchroman-4-One,7-Hydroxy-5-Methoxyflavanone | ESR2 |
| Astragalus mongholicus | MOL604 | (S)-5-Hydroxy-7-Methoxy-2-Phenylchroman-4-One,7-Hydroxy-5-Methoxyflavanone | KANSL3 |
| Astragalus mongholicus | MOL605 | (S)-5-Hydroxy-7-Methoxy-2-Phenylchroman-4-One,7-Hydroxy-5-Methoxyflavanone | MTTP |
| Astragalus mongholicus | MOL606 | (S)-5-Hydroxy-7-Methoxy-2-Phenylchroman-4-One,7-Hydroxy-5-Methoxyflavanone | SHBG |
| Astragalus mongholicus | MOL607 | (S)-5-Hydroxy-7-Methoxy-2-Phenylchroman-4-One,7-Hydroxy-5-Methoxyflavanone | SOAT1 |
| Astragalus mongholicus | MOL608 | (S)-5-Hydroxy-7-Methoxy-2-Phenylchroman-4-One,7-Hydroxy-5-Methoxyflavanone | SOAT2 |
| Astragalus mongholicus | MOL609 | (S)-5,7-Dihydroxy-2-Phenylchroman-4-One,Pinocembrin | ACTB |
| Astragalus mongholicus | MOL610 | (S)-5,7-Dihydroxy-2-Phenylchroman-4-One,Pinocembrin | AHR |
| Astragalus mongholicus | MOL611 | (S)-5,7-Dihydroxy-2-Phenylchroman-4-One,Pinocembrin | AKR1C1 |
| Astragalus mongholicus | MOL612 | (S)-5,7-Dihydroxy-2-Phenylchroman-4-One,Pinocembrin | AKT1 |
| Astragalus mongholicus | MOL613 | (S)-5,7-Dihydroxy-2-Phenylchroman-4-One,Pinocembrin | ATP5A1 |
| Astragalus mongholicus | MOL614 | (S)-5,7-Dihydroxy-2-Phenylchroman-4-One,Pinocembrin | ATP5B |
| Astragalus mongholicus | MOL615 | (S)-5,7-Dihydroxy-2-Phenylchroman-4-One,Pinocembrin | ATP5C1 |
| Astragalus mongholicus | MOL616 | (S)-5,7-Dihydroxy-2-Phenylchroman-4-One,Pinocembrin | CBR1 |
| Astragalus mongholicus | MOL617 | (S)-5,7-Dihydroxy-2-Phenylchroman-4-One,Pinocembrin | CDK6 |
| Astragalus mongholicus | MOL618 | (S)-5,7-Dihydroxy-2-Phenylchroman-4-One,Pinocembrin | CEBPB |
| Astragalus mongholicus | MOL619 | (S)-5,7-Dihydroxy-2-Phenylchroman-4-One,Pinocembrin | CSNK2A1 |
| Astragalus mongholicus | MOL620 | (S)-5,7-Dihydroxy-2-Phenylchroman-4-One,Pinocembrin | CSNK2B |
| Astragalus mongholicus | MOL621 | (S)-5,7-Dihydroxy-2-Phenylchroman-4-One,Pinocembrin | CYP19A1 |
| Astragalus mongholicus | MOL622 | (S)-5,7-Dihydroxy-2-Phenylchroman-4-One,Pinocembrin | CYP1B1 |
| Astragalus mongholicus | MOL623 | (S)-5,7-Dihydroxy-2-Phenylchroman-4-One,Pinocembrin | EIF3F |
| Astragalus mongholicus | MOL624 | (S)-5,7-Dihydroxy-2-Phenylchroman-4-One,Pinocembrin | ESR1 |
| Astragalus mongholicus | MOL625 | (S)-5,7-Dihydroxy-2-Phenylchroman-4-One,Pinocembrin | ESR2 |
| Astragalus mongholicus | MOL626 | (S)-5,7-Dihydroxy-2-Phenylchroman-4-One,Pinocembrin | ESRRA |
| Astragalus mongholicus | MOL627 | (S)-5,7-Dihydroxy-2-Phenylchroman-4-One,Pinocembrin | ESRRB |
| Astragalus mongholicus | MOL628 | (S)-5,7-Dihydroxy-2-Phenylchroman-4-One,Pinocembrin | GPER1 |
| Astragalus mongholicus | MOL629 | (S)-5,7-Dihydroxy-2-Phenylchroman-4-One,Pinocembrin | HCK |
| Astragalus mongholicus | MOL630 | (S)-5,7-Dihydroxy-2-Phenylchroman-4-One,Pinocembrin | HIBCH |
| Astragalus mongholicus | MOL631 | (S)-5,7-Dihydroxy-2-Phenylchroman-4-One,Pinocembrin | HSP90AA1 |
| Astragalus mongholicus | MOL632 | (S)-5,7-Dihydroxy-2-Phenylchroman-4-One,Pinocembrin | HSPA2 |
| Astragalus mongholicus | MOL633 | (S)-5,7-Dihydroxy-2-Phenylchroman-4-One,Pinocembrin | IGHG1 |
| Astragalus mongholicus | MOL634 | (S)-5,7-Dihydroxy-2-Phenylchroman-4-One,Pinocembrin | JAK1 |
| Astragalus mongholicus | MOL635 | (S)-5,7-Dihydroxy-2-Phenylchroman-4-One,Pinocembrin | KANSL3 |
| Astragalus mongholicus | MOL636 | (S)-5,7-Dihydroxy-2-Phenylchroman-4-One,Pinocembrin | MTTP |
| Astragalus mongholicus | MOL637 | (S)-5,7-Dihydroxy-2-Phenylchroman-4-One,Pinocembrin | NCOA1 |
| Astragalus mongholicus | MOL638 | (S)-5,7-Dihydroxy-2-Phenylchroman-4-One,Pinocembrin | NCOA2 |
| Astragalus mongholicus | MOL639 | (S)-5,7-Dihydroxy-2-Phenylchroman-4-One,Pinocembrin | NQO2 |
| Astragalus mongholicus | MOL640 | (S)-5,7-Dihydroxy-2-Phenylchroman-4-One,Pinocembrin | NR1I2 |
| Astragalus mongholicus | MOL641 | (S)-5,7-Dihydroxy-2-Phenylchroman-4-One,Pinocembrin | PIK3CG |
| Astragalus mongholicus | MOL642 | (S)-5,7-Dihydroxy-2-Phenylchroman-4-One,Pinocembrin | PIM1 |
| Astragalus mongholicus | MOL643 | (S)-5,7-Dihydroxy-2-Phenylchroman-4-One,Pinocembrin | PTK2B |
| Astragalus mongholicus | MOL644 | (S)-5,7-Dihydroxy-2-Phenylchroman-4-One,Pinocembrin | RUVBL2 |
| Astragalus mongholicus | MOL645 | (S)-5,7-Dihydroxy-2-Phenylchroman-4-One,Pinocembrin | SF3B3 |
| Astragalus mongholicus | MOL646 | (S)-5,7-Dihydroxy-2-Phenylchroman-4-One,Pinocembrin | SHBG |
| Astragalus mongholicus | MOL647 | (S)-5,7-Dihydroxy-2-Phenylchroman-4-One,Pinocembrin | SOAT1 |
| Astragalus mongholicus | MOL648 | (S)-5,7-Dihydroxy-2-Phenylchroman-4-One,Pinocembrin | SOAT2 |
| Astragalus mongholicus | MOL649 | (S)-5,7-Dihydroxy-2-Phenylchroman-4-One,Pinocembrin | STK17B |
| Astragalus mongholicus | MOL650 | (S)-5,7-Dihydroxy-2-Phenylchroman-4-One,Pinocembrin | TOP2A |
| Astragalus mongholicus | MOL651 | (S)-5,7-Dihydroxy-2-Phenylchroman-4-One,Pinocembrin | UBA1 |
| Astragalus mongholicus | MOL652 | (S)-5,7-Dihydroxy-2-Phenylchroman-4-One,Pinocembrin | UGT3A1 |
| Astragalus mongholicus | MOL653 | 1-(2,4-Dihydroxy-6-Methoxyphenyl)-3-Phenylprop-2-En-1-One,Cardamonin | AR |
| Astragalus mongholicus | MOL654 | 1-(2,4-Dihydroxy-6-Methoxyphenyl)-3-Phenylprop-2-En-1-One,Cardamonin | CTRB1 |
| Astragalus mongholicus | MOL655 | 1-(2,4-Dihydroxy-6-Methoxyphenyl)-3-Phenylprop-2-En-1-One,Cardamonin | ESR1 |
| Astragalus mongholicus | MOL656 | 1-(2,4-Dihydroxy-6-Methoxyphenyl)-3-Phenylprop-2-En-1-One,Cardamonin | ESR2 |
| Astragalus mongholicus | MOL657 | 1-(2,4-Dihydroxy-6-Methoxyphenyl)-3-Phenylprop-2-En-1-One,Cardamonin | PGR |
| Astragalus mongholicus | MOL658 | 1-(2,4-Dihydroxy-6-Methoxyphenyl)-3-Phenylprop-2-En-1-One,Cardamonin | SHBG |
| Astragalus mongholicus | MOL659 | 1-(2,4-Dihydroxy-6-Methoxyphenyl)-3-Phenylprop-2-En-1-One,Cardamonin | Octanal |
| Astragalus mongholicus | MOL660 | 1-(2,4-Dihydroxy-6-Methoxyphenyl)-3-Phenylprop-2-En-1-One,Cardamonin | ACOT13 |
| Astragalus mongholicus | MOL661 | 1-(2,4-Dihydroxy-6-Methoxyphenyl)-3-Phenylprop-2-En-1-One,Cardamonin | DBI |
| Astragalus mongholicus | MOL662 | 1-(2,4-Dihydroxy-6-Methoxyphenyl)-3-Phenylprop-2-En-1-One,Cardamonin | RHO |
| Astragalus mongholicus | MOL663 | 1-(2,4-Dihydroxy-6-Methoxyphenyl)-3-Phenylprop-2-En-1-One,Cardamonin | Nonan-2-One |
| Astragalus mongholicus | MOL664 | 1-(2,4-Dihydroxy-6-Methoxyphenyl)-3-Phenylprop-2-En-1-One,Cardamonin | ACOT13 |
| Astragalus mongholicus | MOL665 | 1-(2,4-Dihydroxy-6-Methoxyphenyl)-3-Phenylprop-2-En-1-One,Cardamonin | DBI |
| Astragalus mongholicus | MOL666 | 1-(2,4-Dihydroxy-6-Methoxyphenyl)-3-Phenylprop-2-En-1-One,Cardamonin | RHO |
| Astragalus mongholicus | MOL667 | 1-(2,4-Dihydroxy-6-Methoxyphenyl)-3-Phenylprop-2-En-1-One,Cardamonin | Methyl-N-Nonylketone,Undecan-2-One |
| Astragalus mongholicus | MOL668 | 1-(2,4-Dihydroxy-6-Methoxyphenyl)-3-Phenylprop-2-En-1-One,Cardamonin | ACOT13 |
| Astragalus mongholicus | MOL669 | 1-(2,4-Dihydroxy-6-Methoxyphenyl)-3-Phenylprop-2-En-1-One,Cardamonin | DBI |
| Astragalus mongholicus | MOL670 | 1-(2,4-Dihydroxy-6-Methoxyphenyl)-3-Phenylprop-2-En-1-One,Cardamonin | RHO |
| Astragalus mongholicus | MOL671 | (-)-CaryophylleneOxide,(1R,4R,6R,10S)-4,12,12-Trimethyl-9-Methylidene-5-Oxatricyclo[8.2.0.0~4,6~]Dodecane | LCN9 |
| Astragalus mongholicus | MOL672 | (-)-CaryophylleneOxide,(1R,4R,6R,10S)-4,12,12-Trimethyl-9-Methylidene-5-Oxatricyclo[8.2.0.0~4,6~]Dodecane | Methanol |
| Astragalus mongholicus | MOL673 | (-)-CaryophylleneOxide,(1R,4R,6R,10S)-4,12,12-Trimethyl-9-Methylidene-5-Oxatricyclo[8.2.0.0~4,6~]Dodecane | MB |
| Astragalus mongholicus | MOL674 | (-)-CaryophylleneOxide,(1R,4R,6R,10S)-4,12,12-Trimethyl-9-Methylidene-5-Oxatricyclo[8.2.0.0~4,6~]Dodecane | Ethyl3-(4-Methoxyphenyl)Acrylate |
| Astragalus mongholicus | MOL675 | (-)-CaryophylleneOxide,(1R,4R,6R,10S)-4,12,12-Trimethyl-9-Methylidene-5-Oxatricyclo[8.2.0.0~4,6~]Dodecane | RARA |
| Astragalus mongholicus | MOL676 | (-)-CaryophylleneOxide,(1R,4R,6R,10S)-4,12,12-Trimethyl-9-Methylidene-5-Oxatricyclo[8.2.0.0~4,6~]Dodecane | RARB |
| Astragalus mongholicus | MOL677 | (-)-CaryophylleneOxide,(1R,4R,6R,10S)-4,12,12-Trimethyl-9-Methylidene-5-Oxatricyclo[8.2.0.0~4,6~]Dodecane | RARG |
| Astragalus mongholicus | MOL678 | (-)-CaryophylleneOxide,(1R,4R,6R,10S)-4,12,12-Trimethyl-9-Methylidene-5-Oxatricyclo[8.2.0.0~4,6~]Dodecane | RXRA |
| Astragalus mongholicus | MOL679 | (-)-CaryophylleneOxide,(1R,4R,6R,10S)-4,12,12-Trimethyl-9-Methylidene-5-Oxatricyclo[8.2.0.0~4,6~]Dodecane | RXRB |
| Astragalus mongholicus | MOL680 | (-)-CaryophylleneOxide,(1R,4R,6R,10S)-4,12,12-Trimethyl-9-Methylidene-5-Oxatricyclo[8.2.0.0~4,6~]Dodecane | RXRG |
| Astragalus mongholicus | MOL681 | (-)-CaryophylleneOxide,(1R,4R,6R,10S)-4,12,12-Trimethyl-9-Methylidene-5-Oxatricyclo[8.2.0.0~4,6~]Dodecane | Bicyclo[3.3.1]Nonan-2-Ol |
| Astragalus mongholicus | MOL682 | (-)-CaryophylleneOxide,(1R,4R,6R,10S)-4,12,12-Trimethyl-9-Methylidene-5-Oxatricyclo[8.2.0.0~4,6~]Dodecane | ADH1B |
| Astragalus mongholicus | MOL683 | (S)-2-Methyl-6-(4-Methylcyclohex-3-En-1-Yl)Hepta-2,6-Dien-1-Ol | NR1H4 |
| Astragalus mongholicus | MOL684 | (S)-2-Methyl-6-(4-Methylcyclohex-3-En-1-Yl)Hepta-2,6-Dien-1-Ol | MAOB |
| Astragalus mongholicus | MOL685 | (1R,2S,5S)-5-Isopropyl-2-Methylbicyclo[3.1.0]Hexan-2-Ol | NR1I3 |
| Astragalus mongholicus | MOL686 | (8S,8As)-8-Hydroxy-3,5,8A-Trimethyl-7,8,8A,9-Tetrahydronaphtho[2,3-B]Furan-4(6H)-One | AR |
| Astragalus mongholicus | MOL687 | (8S,8As)-8-Hydroxy-3,5,8A-Trimethyl-7,8,8A,9-Tetrahydronaphtho[2,3-B]Furan-4(6H)-One | ESR1 |
| Astragalus mongholicus | MOL688 | (8S,8As)-8-Hydroxy-3,5,8A-Trimethyl-7,8,8A,9-Tetrahydronaphtho[2,3-B]Furan-4(6H)-One | ESR2 |
| Astragalus mongholicus | MOL689 | (8S,8As)-8-Hydroxy-3,5,8A-Trimethyl-7,8,8A,9-Tetrahydronaphtho[2,3-B]Furan-4(6H)-One | GABRA1 |
| Astragalus mongholicus | MOL690 | (8S,8As)-8-Hydroxy-3,5,8A-Trimethyl-7,8,8A,9-Tetrahydronaphtho[2,3-B]Furan-4(6H)-One | GABRA2 |
| Astragalus mongholicus | MOL691 | (8S,8As)-8-Hydroxy-3,5,8A-Trimethyl-7,8,8A,9-Tetrahydronaphtho[2,3-B]Furan-4(6H)-One | GABRA3 |
| Astragalus mongholicus | MOL692 | (8S,8As)-8-Hydroxy-3,5,8A-Trimethyl-7,8,8A,9-Tetrahydronaphtho[2,3-B]Furan-4(6H)-One | GABRA4 |
| Astragalus mongholicus | MOL693 | (8S,8As)-8-Hydroxy-3,5,8A-Trimethyl-7,8,8A,9-Tetrahydronaphtho[2,3-B]Furan-4(6H)-One | GABRA5 |
| Astragalus mongholicus | MOL694 | (8S,8As)-8-Hydroxy-3,5,8A-Trimethyl-7,8,8A,9-Tetrahydronaphtho[2,3-B]Furan-4(6H)-One | GABRA6 |
| Astragalus mongholicus | MOL695 | (8S,8As)-8-Hydroxy-3,5,8A-Trimethyl-7,8,8A,9-Tetrahydronaphtho[2,3-B]Furan-4(6H)-One | GABRB1 |
| Astragalus mongholicus | MOL696 | (8S,8As)-8-Hydroxy-3,5,8A-Trimethyl-7,8,8A,9-Tetrahydronaphtho[2,3-B]Furan-4(6H)-One | GABRB2 |
| Astragalus mongholicus | MOL697 | (8S,8As)-8-Hydroxy-3,5,8A-Trimethyl-7,8,8A,9-Tetrahydronaphtho[2,3-B]Furan-4(6H)-One | GABRB3 |
| Astragalus mongholicus | MOL698 | (8S,8As)-8-Hydroxy-3,5,8A-Trimethyl-7,8,8A,9-Tetrahydronaphtho[2,3-B]Furan-4(6H)-One | GABRD |
| Astragalus mongholicus | MOL699 | (8S,8As)-8-Hydroxy-3,5,8A-Trimethyl-7,8,8A,9-Tetrahydronaphtho[2,3-B]Furan-4(6H)-One | GABRE |
| Astragalus mongholicus | MOL700 | (8S,8As)-8-Hydroxy-3,5,8A-Trimethyl-7,8,8A,9-Tetrahydronaphtho[2,3-B]Furan-4(6H)-One | GABRG1 |
| Astragalus mongholicus | MOL701 | (8S,8As)-8-Hydroxy-3,5,8A-Trimethyl-7,8,8A,9-Tetrahydronaphtho[2,3-B]Furan-4(6H)-One | GABRG2 |
| Astragalus mongholicus | MOL702 | (8S,8As)-8-Hydroxy-3,5,8A-Trimethyl-7,8,8A,9-Tetrahydronaphtho[2,3-B]Furan-4(6H)-One | GABRG3 |
| Astragalus mongholicus | MOL703 | (8S,8As)-8-Hydroxy-3,5,8A-Trimethyl-7,8,8A,9-Tetrahydronaphtho[2,3-B]Furan-4(6H)-One | GABRP |
| Astragalus mongholicus | MOL704 | (8S,8As)-8-Hydroxy-3,5,8A-Trimethyl-7,8,8A,9-Tetrahydronaphtho[2,3-B]Furan-4(6H)-One | GABRQ |
| Astragalus mongholicus | MOL705 | (8S,8As)-8-Hydroxy-3,5,8A-Trimethyl-7,8,8A,9-Tetrahydronaphtho[2,3-B]Furan-4(6H)-One | GRIN1 |
| Astragalus mongholicus | MOL706 | (8S,8As)-8-Hydroxy-3,5,8A-Trimethyl-7,8,8A,9-Tetrahydronaphtho[2,3-B]Furan-4(6H)-One | GRIN2A |
| Astragalus mongholicus | MOL707 | (8S,8As)-8-Hydroxy-3,5,8A-Trimethyl-7,8,8A,9-Tetrahydronaphtho[2,3-B]Furan-4(6H)-One | GRIN2B |
| Astragalus mongholicus | MOL708 | (8S,8As)-8-Hydroxy-3,5,8A-Trimethyl-7,8,8A,9-Tetrahydronaphtho[2,3-B]Furan-4(6H)-One | GRIN2C |
| Astragalus mongholicus | MOL709 | (8S,8As)-8-Hydroxy-3,5,8A-Trimethyl-7,8,8A,9-Tetrahydronaphtho[2,3-B]Furan-4(6H)-One | GRIN2D |
| Astragalus mongholicus | MOL710 | (8S,8As)-8-Hydroxy-3,5,8A-Trimethyl-7,8,8A,9-Tetrahydronaphtho[2,3-B]Furan-4(6H)-One | GRIN3A |
| Astragalus mongholicus | MOL711 | (8S,8As)-8-Hydroxy-3,5,8A-Trimethyl-7,8,8A,9-Tetrahydronaphtho[2,3-B]Furan-4(6H)-One | GRIN3B |
| Astragalus mongholicus | MOL712 | (8S,8As)-8-Hydroxy-3,5,8A-Trimethyl-7,8,8A,9-Tetrahydronaphtho[2,3-B]Furan-4(6H)-One | HSD11B2 |
| Astragalus mongholicus | MOL713 | (8S,8As)-8-Hydroxy-3,5,8A-Trimethyl-7,8,8A,9-Tetrahydronaphtho[2,3-B]Furan-4(6H)-One | HSD17B1 |
| Astragalus mongholicus | MOL714 | (8S,8As)-8-Hydroxy-3,5,8A-Trimethyl-7,8,8A,9-Tetrahydronaphtho[2,3-B]Furan-4(6H)-One | NPPB |
| Astragalus mongholicus | MOL715 | (8S,8As)-8-Hydroxy-3,5,8A-Trimethyl-7,8,8A,9-Tetrahydronaphtho[2,3-B]Furan-4(6H)-One | NR1I2 |
| Astragalus mongholicus | MOL716 | (8S,8As)-8-Hydroxy-3,5,8A-Trimethyl-7,8,8A,9-Tetrahydronaphtho[2,3-B]Furan-4(6H)-One | NR1I3 |
| Astragalus mongholicus | MOL717 | (8S,8As)-8-Hydroxy-3,5,8A-Trimethyl-7,8,8A,9-Tetrahydronaphtho[2,3-B]Furan-4(6H)-One | PPARA |
| Astragalus mongholicus | MOL718 | (8S,8As)-8-Hydroxy-3,5,8A-Trimethyl-7,8,8A,9-Tetrahydronaphtho[2,3-B]Furan-4(6H)-One | SIGMAR1 |
| Astragalus mongholicus | MOL719 | (8S,8As)-8-Hydroxy-3,5,8A-Trimethyl-7,8,8A,9-Tetrahydronaphtho[2,3-B]Furan-4(6H)-One | SULT2A1 |
| Astragalus mongholicus | MOL720 | (8S,8As)-8-Hydroxy-3,5,8A-Trimethyl-7,8,8A,9-Tetrahydronaphtho[2,3-B]Furan-4(6H)-One | SULT2B1 |
| Astragalus mongholicus | MOL721 | (1R,11R)-1,5,9,9-Tetramethyl-12-Oxabicyclo[9.1.0]Dodeca-4,7-Diene | LCN9 |
| Astragalus mongholicus | MOL722 | (1R,11R)-1,5,5,8-Tetramethyl-12-Oxabicyclo[9.1.0]Dodeca-3,7-Diene | LCN9 |
| Astragalus mongholicus | MOL723 | (3S,3As,8Ar,Z)-3-Hydroxy-3,8-Dimethyl-5-(Propan-2-Ylidene)-1,2,3,3A,4,5-Hexahydroazulen-6(8Ah)-One | AKR1C1 |
| Astragalus mongholicus | MOL724 | (3S,3As,8Ar,Z)-3-Hydroxy-3,8-Dimethyl-5-(Propan-2-Ylidene)-1,2,3,3A,4,5-Hexahydroazulen-6(8Ah)-One | AKR1C2 |
| Astragalus mongholicus | MOL725 | (3S,3As,8Ar,Z)-3-Hydroxy-3,8-Dimethyl-5-(Propan-2-Ylidene)-1,2,3,3A,4,5-Hexahydroazulen-6(8Ah)-One | AR |
| Astragalus mongholicus | MOL726 | (3S,3As,8Ar,Z)-3-Hydroxy-3,8-Dimethyl-5-(Propan-2-Ylidene)-1,2,3,3A,4,5-Hexahydroazulen-6(8Ah)-One | ESR1 |
| Astragalus mongholicus | MOL727 | (3S,3As,8Ar,Z)-3-Hydroxy-3,8-Dimethyl-5-(Propan-2-Ylidene)-1,2,3,3A,4,5-Hexahydroazulen-6(8Ah)-One | ESR2 |
| Astragalus mongholicus | MOL728 | (3S,3As,8Ar,Z)-3-Hydroxy-3,8-Dimethyl-5-(Propan-2-Ylidene)-1,2,3,3A,4,5-Hexahydroazulen-6(8Ah)-One | GABRA1 |
| Astragalus mongholicus | MOL729 | (3S,3As,8Ar,Z)-3-Hydroxy-3,8-Dimethyl-5-(Propan-2-Ylidene)-1,2,3,3A,4,5-Hexahydroazulen-6(8Ah)-One | GABRA2 |
| Astragalus mongholicus | MOL730 | (3S,3As,8Ar,Z)-3-Hydroxy-3,8-Dimethyl-5-(Propan-2-Ylidene)-1,2,3,3A,4,5-Hexahydroazulen-6(8Ah)-One | GABRA3 |
| Astragalus mongholicus | MOL731 | (3S,3As,8Ar,Z)-3-Hydroxy-3,8-Dimethyl-5-(Propan-2-Ylidene)-1,2,3,3A,4,5-Hexahydroazulen-6(8Ah)-One | GABRA4 |
| Astragalus mongholicus | MOL732 | (3S,3As,8Ar,Z)-3-Hydroxy-3,8-Dimethyl-5-(Propan-2-Ylidene)-1,2,3,3A,4,5-Hexahydroazulen-6(8Ah)-One | GABRA5 |
| Astragalus mongholicus | MOL733 | (3S,3As,8Ar,Z)-3-Hydroxy-3,8-Dimethyl-5-(Propan-2-Ylidene)-1,2,3,3A,4,5-Hexahydroazulen-6(8Ah)-One | GABRA6 |
| Astragalus mongholicus | MOL734 | (3S,3As,8Ar,Z)-3-Hydroxy-3,8-Dimethyl-5-(Propan-2-Ylidene)-1,2,3,3A,4,5-Hexahydroazulen-6(8Ah)-One | GABRB1 |
| Astragalus mongholicus | MOL735 | (3S,3As,8Ar,Z)-3-Hydroxy-3,8-Dimethyl-5-(Propan-2-Ylidene)-1,2,3,3A,4,5-Hexahydroazulen-6(8Ah)-One | GABRB2 |
| Astragalus mongholicus | MOL736 | (3S,3As,8Ar,Z)-3-Hydroxy-3,8-Dimethyl-5-(Propan-2-Ylidene)-1,2,3,3A,4,5-Hexahydroazulen-6(8Ah)-One | GABRB3 |
| Astragalus mongholicus | MOL737 | (3S,3As,8Ar,Z)-3-Hydroxy-3,8-Dimethyl-5-(Propan-2-Ylidene)-1,2,3,3A,4,5-Hexahydroazulen-6(8Ah)-One | GABRD |
| Astragalus mongholicus | MOL738 | (3S,3As,8Ar,Z)-3-Hydroxy-3,8-Dimethyl-5-(Propan-2-Ylidene)-1,2,3,3A,4,5-Hexahydroazulen-6(8Ah)-One | GABRE |
| Astragalus mongholicus | MOL739 | (3S,3As,8Ar,Z)-3-Hydroxy-3,8-Dimethyl-5-(Propan-2-Ylidene)-1,2,3,3A,4,5-Hexahydroazulen-6(8Ah)-One | GABRG1 |
| Astragalus mongholicus | MOL740 | (3S,3As,8Ar,Z)-3-Hydroxy-3,8-Dimethyl-5-(Propan-2-Ylidene)-1,2,3,3A,4,5-Hexahydroazulen-6(8Ah)-One | GABRG2 |
| Astragalus mongholicus | MOL741 | (3S,3As,8Ar,Z)-3-Hydroxy-3,8-Dimethyl-5-(Propan-2-Ylidene)-1,2,3,3A,4,5-Hexahydroazulen-6(8Ah)-One | GABRG3 |
| Astragalus mongholicus | MOL742 | (3S,3As,8Ar,Z)-3-Hydroxy-3,8-Dimethyl-5-(Propan-2-Ylidene)-1,2,3,3A,4,5-Hexahydroazulen-6(8Ah)-One | GABRP |
| Astragalus mongholicus | MOL743 | (3S,3As,8Ar,Z)-3-Hydroxy-3,8-Dimethyl-5-(Propan-2-Ylidene)-1,2,3,3A,4,5-Hexahydroazulen-6(8Ah)-One | GABRQ |
| Astragalus mongholicus | MOL744 | (3S,3As,8Ar,Z)-3-Hydroxy-3,8-Dimethyl-5-(Propan-2-Ylidene)-1,2,3,3A,4,5-Hexahydroazulen-6(8Ah)-One | GRIN1 |
| Astragalus mongholicus | MOL745 | (3S,3As,8Ar,Z)-3-Hydroxy-3,8-Dimethyl-5-(Propan-2-Ylidene)-1,2,3,3A,4,5-Hexahydroazulen-6(8Ah)-One | GRIN2A |
| Astragalus mongholicus | MOL746 | (3S,3As,8Ar,Z)-3-Hydroxy-3,8-Dimethyl-5-(Propan-2-Ylidene)-1,2,3,3A,4,5-Hexahydroazulen-6(8Ah)-One | GRIN2B |
| Astragalus mongholicus | MOL747 | (3S,3As,8Ar,Z)-3-Hydroxy-3,8-Dimethyl-5-(Propan-2-Ylidene)-1,2,3,3A,4,5-Hexahydroazulen-6(8Ah)-One | GRIN2C |
| Astragalus mongholicus | MOL748 | (3S,3As,8Ar,Z)-3-Hydroxy-3,8-Dimethyl-5-(Propan-2-Ylidene)-1,2,3,3A,4,5-Hexahydroazulen-6(8Ah)-One | GRIN2D |
| Astragalus mongholicus | MOL749 | (3S,3As,8Ar,Z)-3-Hydroxy-3,8-Dimethyl-5-(Propan-2-Ylidene)-1,2,3,3A,4,5-Hexahydroazulen-6(8Ah)-One | GRIN3A |
| Astragalus mongholicus | MOL750 | (3S,3As,8Ar,Z)-3-Hydroxy-3,8-Dimethyl-5-(Propan-2-Ylidene)-1,2,3,3A,4,5-Hexahydroazulen-6(8Ah)-One | GRIN3B |
| Astragalus mongholicus | MOL751 | (3S,3As,8Ar,Z)-3-Hydroxy-3,8-Dimethyl-5-(Propan-2-Ylidene)-1,2,3,3A,4,5-Hexahydroazulen-6(8Ah)-One | HSD17B1 |
| Astragalus mongholicus | MOL752 | (3S,3As,8Ar,Z)-3-Hydroxy-3,8-Dimethyl-5-(Propan-2-Ylidene)-1,2,3,3A,4,5-Hexahydroazulen-6(8Ah)-One | LSS |
| Astragalus mongholicus | MOL753 | (3S,3As,8Ar,Z)-3-Hydroxy-3,8-Dimethyl-5-(Propan-2-Ylidene)-1,2,3,3A,4,5-Hexahydroazulen-6(8Ah)-One | NCOA2 |
| Astragalus mongholicus | MOL754 | (3S,3As,8Ar,Z)-3-Hydroxy-3,8-Dimethyl-5-(Propan-2-Ylidene)-1,2,3,3A,4,5-Hexahydroazulen-6(8Ah)-One | NPPB |
| Astragalus mongholicus | MOL755 | (3S,3As,8Ar,Z)-3-Hydroxy-3,8-Dimethyl-5-(Propan-2-Ylidene)-1,2,3,3A,4,5-Hexahydroazulen-6(8Ah)-One | NR1I2 |
| Astragalus mongholicus | MOL756 | (3S,3As,8Ar,Z)-3-Hydroxy-3,8-Dimethyl-5-(Propan-2-Ylidene)-1,2,3,3A,4,5-Hexahydroazulen-6(8Ah)-One | NR1I3 |
| Astragalus mongholicus | MOL757 | (3S,3As,8Ar,Z)-3-Hydroxy-3,8-Dimethyl-5-(Propan-2-Ylidene)-1,2,3,3A,4,5-Hexahydroazulen-6(8Ah)-One | NR3C2 |
| Astragalus mongholicus | MOL758 | (3S,3As,8Ar,Z)-3-Hydroxy-3,8-Dimethyl-5-(Propan-2-Ylidene)-1,2,3,3A,4,5-Hexahydroazulen-6(8Ah)-One | PGR |
| Astragalus mongholicus | MOL759 | (3S,3As,8Ar,Z)-3-Hydroxy-3,8-Dimethyl-5-(Propan-2-Ylidene)-1,2,3,3A,4,5-Hexahydroazulen-6(8Ah)-One | PPARA |
| Astragalus mongholicus | MOL760 | (3S,3As,8Ar,Z)-3-Hydroxy-3,8-Dimethyl-5-(Propan-2-Ylidene)-1,2,3,3A,4,5-Hexahydroazulen-6(8Ah)-One | SIGMAR1 |
| Astragalus mongholicus | MOL761 | (3S,3As,8Ar,Z)-3-Hydroxy-3,8-Dimethyl-5-(Propan-2-Ylidene)-1,2,3,3A,4,5-Hexahydroazulen-6(8Ah)-One | SULT2A1 |
| Astragalus mongholicus | MOL762 | (3S,3As,8Ar,Z)-3-Hydroxy-3,8-Dimethyl-5-(Propan-2-Ylidene)-1,2,3,3A,4,5-Hexahydroazulen-6(8Ah)-One | SULT2B1 |
| Astragalus mongholicus | MOL763 | (3S,3As,8Ar,Z)-3-Hydroxy-3,8-Dimethyl-5-(Propan-2-Ylidene)-1,2,3,3A,4,5-Hexahydroazulen-6(8Ah)-One | VDR |
| Astragalus mongholicus | MOL764 | 1,7-Bis(4-Hydroxyphenyl)Hepta-1,6-Diene-3,5-Dione | PYGM |
| Astragalus mongholicus | MOL765 | 2,4-Dimethyloctan-4-Ol | LCN9 |
| Astragalus mongholicus | MOL766 | 2,3,3-Trimethyl-2-(3-Methylbuta-1,3-Dien-1-Yl)-6-Methylenecyclohexanone | AR |
| Astragalus mongholicus | MOL767 | 2,3,3-Trimethyl-2-(3-Methylbuta-1,3-Dien-1-Yl)-6-Methylenecyclohexanone | ESR1 |
| Astragalus mongholicus | MOL768 | 2,3,3-Trimethyl-2-(3-Methylbuta-1,3-Dien-1-Yl)-6-Methylenecyclohexanone | ESR2 |
| Astragalus mongholicus | MOL769 | 2,3,3-Trimethyl-2-(3-Methylbuta-1,3-Dien-1-Yl)-6-Methylenecyclohexanone | PGR |
| Astragalus mongholicus | MOL770 | (3Z,7Z)-3,7,10,10-Tetramethyl-12-Oxa-Bicyclo[9.1.0]Dodeca-3,7-Diene | LCN9 |
| Astragalus mongholicus | MOL771 | (3Z,7Z)-3,7,10,10-Tetramethyl-12-Oxa-Bicyclo[9.1.0]Dodeca-3,7-Diene | Decan-2-One |
| Astragalus mongholicus | MOL772 | (3Z,7Z)-3,7,10,10-Tetramethyl-12-Oxa-Bicyclo[9.1.0]Dodeca-3,7-Diene | ACOT13 |
| Astragalus mongholicus | MOL773 | (3Z,7Z)-3,7,10,10-Tetramethyl-12-Oxa-Bicyclo[9.1.0]Dodeca-3,7-Diene | DBI |
| Astragalus mongholicus | MOL774 | (3Z,7Z)-3,7,10,10-Tetramethyl-12-Oxa-Bicyclo[9.1.0]Dodeca-3,7-Diene | RHO |
| Astragalus mongholicus | MOL775 | (5S,8R,9S,10S,13S,14S)-3-Ethyl-3-Hydroxy-10,13-Dimethyl-Tetradecahydro-2H-Cyclopenta[A]Phenanthren-17(14H)-One | ADH1C |
| Astragalus mongholicus | MOL776 | (5S,8R,9S,10S,13S,14S)-3-Ethyl-3-Hydroxy-10,13-Dimethyl-Tetradecahydro-2H-Cyclopenta[A]Phenanthren-17(14H)-One | AKR1C1 |
| Astragalus mongholicus | MOL777 | (5S,8R,9S,10S,13S,14S)-3-Ethyl-3-Hydroxy-10,13-Dimethyl-Tetradecahydro-2H-Cyclopenta[A]Phenanthren-17(14H)-One | AKR1C2 |
| Astragalus mongholicus | MOL778 | (5S,8R,9S,10S,13S,14S)-3-Ethyl-3-Hydroxy-10,13-Dimethyl-Tetradecahydro-2H-Cyclopenta[A]Phenanthren-17(14H)-One | AKR1D1 |
| Astragalus mongholicus | MOL779 | (5S,8R,9S,10S,13S,14S)-3-Ethyl-3-Hydroxy-10,13-Dimethyl-Tetradecahydro-2H-Cyclopenta[A]Phenanthren-17(14H)-One | AR |
| Astragalus mongholicus | MOL780 | (5S,8R,9S,10S,13S,14S)-3-Ethyl-3-Hydroxy-10,13-Dimethyl-Tetradecahydro-2H-Cyclopenta[A]Phenanthren-17(14H)-One | BCL2 |
| Astragalus mongholicus | MOL781 | (5S,8R,9S,10S,13S,14S)-3-Ethyl-3-Hydroxy-10,13-Dimethyl-Tetradecahydro-2H-Cyclopenta[A]Phenanthren-17(14H)-One | CES1 |
| Astragalus mongholicus | MOL782 | (5S,8R,9S,10S,13S,14S)-3-Ethyl-3-Hydroxy-10,13-Dimethyl-Tetradecahydro-2H-Cyclopenta[A]Phenanthren-17(14H)-One | COX4I1 |
| Astragalus mongholicus | MOL783 | (5S,8R,9S,10S,13S,14S)-3-Ethyl-3-Hydroxy-10,13-Dimethyl-Tetradecahydro-2H-Cyclopenta[A]Phenanthren-17(14H)-One | COX5A |
| Astragalus mongholicus | MOL784 | (5S,8R,9S,10S,13S,14S)-3-Ethyl-3-Hydroxy-10,13-Dimethyl-Tetradecahydro-2H-Cyclopenta[A]Phenanthren-17(14H)-One | COX5B |
| Astragalus mongholicus | MOL785 | (5S,8R,9S,10S,13S,14S)-3-Ethyl-3-Hydroxy-10,13-Dimethyl-Tetradecahydro-2H-Cyclopenta[A]Phenanthren-17(14H)-One | COX6A2 |
| Astragalus mongholicus | MOL786 | (5S,8R,9S,10S,13S,14S)-3-Ethyl-3-Hydroxy-10,13-Dimethyl-Tetradecahydro-2H-Cyclopenta[A]Phenanthren-17(14H)-One | COX6B1 |
| Astragalus mongholicus | MOL787 | (5S,8R,9S,10S,13S,14S)-3-Ethyl-3-Hydroxy-10,13-Dimethyl-Tetradecahydro-2H-Cyclopenta[A]Phenanthren-17(14H)-One | COX6C |
| Astragalus mongholicus | MOL788 | (5S,8R,9S,10S,13S,14S)-3-Ethyl-3-Hydroxy-10,13-Dimethyl-Tetradecahydro-2H-Cyclopenta[A]Phenanthren-17(14H)-One | COX7A1 |
| Astragalus mongholicus | MOL789 | (5S,8R,9S,10S,13S,14S)-3-Ethyl-3-Hydroxy-10,13-Dimethyl-Tetradecahydro-2H-Cyclopenta[A]Phenanthren-17(14H)-One | COX7B |
| Astragalus mongholicus | MOL790 | (5S,8R,9S,10S,13S,14S)-3-Ethyl-3-Hydroxy-10,13-Dimethyl-Tetradecahydro-2H-Cyclopenta[A]Phenanthren-17(14H)-One | COX7C |
| Astragalus mongholicus | MOL791 | (5S,8R,9S,10S,13S,14S)-3-Ethyl-3-Hydroxy-10,13-Dimethyl-Tetradecahydro-2H-Cyclopenta[A]Phenanthren-17(14H)-One | COX8A |
| Astragalus mongholicus | MOL792 | (5S,8R,9S,10S,13S,14S)-3-Ethyl-3-Hydroxy-10,13-Dimethyl-Tetradecahydro-2H-Cyclopenta[A]Phenanthren-17(14H)-One | EFTUD1 |
| Astragalus mongholicus | MOL793 | (5S,8R,9S,10S,13S,14S)-3-Ethyl-3-Hydroxy-10,13-Dimethyl-Tetradecahydro-2H-Cyclopenta[A]Phenanthren-17(14H)-One | ESR1 |
| Astragalus mongholicus | MOL794 | (5S,8R,9S,10S,13S,14S)-3-Ethyl-3-Hydroxy-10,13-Dimethyl-Tetradecahydro-2H-Cyclopenta[A]Phenanthren-17(14H)-One | ESR2 |
| Astragalus mongholicus | MOL795 | (5S,8R,9S,10S,13S,14S)-3-Ethyl-3-Hydroxy-10,13-Dimethyl-Tetradecahydro-2H-Cyclopenta[A]Phenanthren-17(14H)-One | ESRRG |
| Astragalus mongholicus | MOL796 | (5S,8R,9S,10S,13S,14S)-3-Ethyl-3-Hydroxy-10,13-Dimethyl-Tetradecahydro-2H-Cyclopenta[A]Phenanthren-17(14H)-One | FABP6 |
| Astragalus mongholicus | MOL797 | (5S,8R,9S,10S,13S,14S)-3-Ethyl-3-Hydroxy-10,13-Dimethyl-Tetradecahydro-2H-Cyclopenta[A]Phenanthren-17(14H)-One | FECH |
| Astragalus mongholicus | MOL798 | (5S,8R,9S,10S,13S,14S)-3-Ethyl-3-Hydroxy-10,13-Dimethyl-Tetradecahydro-2H-Cyclopenta[A]Phenanthren-17(14H)-One | G6PD |
| Astragalus mongholicus | MOL799 | (5S,8R,9S,10S,13S,14S)-3-Ethyl-3-Hydroxy-10,13-Dimethyl-Tetradecahydro-2H-Cyclopenta[A]Phenanthren-17(14H)-One | GABRA1 |
| Astragalus mongholicus | MOL800 | (5S,8R,9S,10S,13S,14S)-3-Ethyl-3-Hydroxy-10,13-Dimethyl-Tetradecahydro-2H-Cyclopenta[A]Phenanthren-17(14H)-One | GABRA2 |
| Astragalus mongholicus | MOL801 | (5S,8R,9S,10S,13S,14S)-3-Ethyl-3-Hydroxy-10,13-Dimethyl-Tetradecahydro-2H-Cyclopenta[A]Phenanthren-17(14H)-One | GABRA3 |
| Astragalus mongholicus | MOL802 | (5S,8R,9S,10S,13S,14S)-3-Ethyl-3-Hydroxy-10,13-Dimethyl-Tetradecahydro-2H-Cyclopenta[A]Phenanthren-17(14H)-One | GABRA4 |
| Astragalus mongholicus | MOL803 | (5S,8R,9S,10S,13S,14S)-3-Ethyl-3-Hydroxy-10,13-Dimethyl-Tetradecahydro-2H-Cyclopenta[A]Phenanthren-17(14H)-One | GABRA5 |
| Astragalus mongholicus | MOL804 | (5S,8R,9S,10S,13S,14S)-3-Ethyl-3-Hydroxy-10,13-Dimethyl-Tetradecahydro-2H-Cyclopenta[A]Phenanthren-17(14H)-One | GABRA6 |
| Astragalus mongholicus | MOL805 | (5S,8R,9S,10S,13S,14S)-3-Ethyl-3-Hydroxy-10,13-Dimethyl-Tetradecahydro-2H-Cyclopenta[A]Phenanthren-17(14H)-One | GABRB1 |
| Astragalus mongholicus | MOL806 | (5S,8R,9S,10S,13S,14S)-3-Ethyl-3-Hydroxy-10,13-Dimethyl-Tetradecahydro-2H-Cyclopenta[A]Phenanthren-17(14H)-One | GABRB2 |
| Astragalus mongholicus | MOL807 | (5S,8R,9S,10S,13S,14S)-3-Ethyl-3-Hydroxy-10,13-Dimethyl-Tetradecahydro-2H-Cyclopenta[A]Phenanthren-17(14H)-One | GABRB3 |
| Astragalus mongholicus | MOL808 | (5S,8R,9S,10S,13S,14S)-3-Ethyl-3-Hydroxy-10,13-Dimethyl-Tetradecahydro-2H-Cyclopenta[A]Phenanthren-17(14H)-One | GABRD |
| Astragalus mongholicus | MOL809 | (5S,8R,9S,10S,13S,14S)-3-Ethyl-3-Hydroxy-10,13-Dimethyl-Tetradecahydro-2H-Cyclopenta[A]Phenanthren-17(14H)-One | GABRE |
| Astragalus mongholicus | MOL810 | (5S,8R,9S,10S,13S,14S)-3-Ethyl-3-Hydroxy-10,13-Dimethyl-Tetradecahydro-2H-Cyclopenta[A]Phenanthren-17(14H)-One | GABRG1 |
| Astragalus mongholicus | MOL811 | (5S,8R,9S,10S,13S,14S)-3-Ethyl-3-Hydroxy-10,13-Dimethyl-Tetradecahydro-2H-Cyclopenta[A]Phenanthren-17(14H)-One | GABRG2 |
| Astragalus mongholicus | MOL812 | (5S,8R,9S,10S,13S,14S)-3-Ethyl-3-Hydroxy-10,13-Dimethyl-Tetradecahydro-2H-Cyclopenta[A]Phenanthren-17(14H)-One | GABRG3 |
| Astragalus mongholicus | MOL813 | (5S,8R,9S,10S,13S,14S)-3-Ethyl-3-Hydroxy-10,13-Dimethyl-Tetradecahydro-2H-Cyclopenta[A]Phenanthren-17(14H)-One | GABRP |
| Astragalus mongholicus | MOL814 | (5S,8R,9S,10S,13S,14S)-3-Ethyl-3-Hydroxy-10,13-Dimethyl-Tetradecahydro-2H-Cyclopenta[A]Phenanthren-17(14H)-One | GABRQ |
| Astragalus mongholicus | MOL815 | (5S,8R,9S,10S,13S,14S)-3-Ethyl-3-Hydroxy-10,13-Dimethyl-Tetradecahydro-2H-Cyclopenta[A]Phenanthren-17(14H)-One | GPBAR1 |
| Astragalus mongholicus | MOL816 | (5S,8R,9S,10S,13S,14S)-3-Ethyl-3-Hydroxy-10,13-Dimethyl-Tetradecahydro-2H-Cyclopenta[A]Phenanthren-17(14H)-One | GSTP1 |
| Astragalus mongholicus | MOL817 | (5S,8R,9S,10S,13S,14S)-3-Ethyl-3-Hydroxy-10,13-Dimethyl-Tetradecahydro-2H-Cyclopenta[A]Phenanthren-17(14H)-One | HSD17B1 |
| Astragalus mongholicus | MOL818 | (5S,8R,9S,10S,13S,14S)-3-Ethyl-3-Hydroxy-10,13-Dimethyl-Tetradecahydro-2H-Cyclopenta[A]Phenanthren-17(14H)-One | HSD17B11 |
| Astragalus mongholicus | MOL819 | (5S,8R,9S,10S,13S,14S)-3-Ethyl-3-Hydroxy-10,13-Dimethyl-Tetradecahydro-2H-Cyclopenta[A]Phenanthren-17(14H)-One | IGHG2 |
| Astragalus mongholicus | MOL820 | (5S,8R,9S,10S,13S,14S)-3-Ethyl-3-Hydroxy-10,13-Dimethyl-Tetradecahydro-2H-Cyclopenta[A]Phenanthren-17(14H)-One | MT-CO1 |
| Astragalus mongholicus | MOL821 | (5S,8R,9S,10S,13S,14S)-3-Ethyl-3-Hydroxy-10,13-Dimethyl-Tetradecahydro-2H-Cyclopenta[A]Phenanthren-17(14H)-One | MT-CO2 |
| Astragalus mongholicus | MOL822 | (5S,8R,9S,10S,13S,14S)-3-Ethyl-3-Hydroxy-10,13-Dimethyl-Tetradecahydro-2H-Cyclopenta[A]Phenanthren-17(14H)-One | MT-CO3 |
| Astragalus mongholicus | MOL823 | (5S,8R,9S,10S,13S,14S)-3-Ethyl-3-Hydroxy-10,13-Dimethyl-Tetradecahydro-2H-Cyclopenta[A]Phenanthren-17(14H)-One | NCOA2 |
| Astragalus mongholicus | MOL824 | (5S,8R,9S,10S,13S,14S)-3-Ethyl-3-Hydroxy-10,13-Dimethyl-Tetradecahydro-2H-Cyclopenta[A]Phenanthren-17(14H)-One | NR1H4 |
| Astragalus mongholicus | MOL825 | (5S,8R,9S,10S,13S,14S)-3-Ethyl-3-Hydroxy-10,13-Dimethyl-Tetradecahydro-2H-Cyclopenta[A]Phenanthren-17(14H)-One | NR1I2 |
| Astragalus mongholicus | MOL826 | (5S,8R,9S,10S,13S,14S)-3-Ethyl-3-Hydroxy-10,13-Dimethyl-Tetradecahydro-2H-Cyclopenta[A]Phenanthren-17(14H)-One | NR3C1 |
| Astragalus mongholicus | MOL827 | (5S,8R,9S,10S,13S,14S)-3-Ethyl-3-Hydroxy-10,13-Dimethyl-Tetradecahydro-2H-Cyclopenta[A]Phenanthren-17(14H)-One | NR3C2 |
| Astragalus mongholicus | MOL828 | (5S,8R,9S,10S,13S,14S)-3-Ethyl-3-Hydroxy-10,13-Dimethyl-Tetradecahydro-2H-Cyclopenta[A]Phenanthren-17(14H)-One | PGR |
| Astragalus mongholicus | MOL829 | (5S,8R,9S,10S,13S,14S)-3-Ethyl-3-Hydroxy-10,13-Dimethyl-Tetradecahydro-2H-Cyclopenta[A]Phenanthren-17(14H)-One | PLA2G1B |
| Astragalus mongholicus | MOL830 | (5S,8R,9S,10S,13S,14S)-3-Ethyl-3-Hydroxy-10,13-Dimethyl-Tetradecahydro-2H-Cyclopenta[A]Phenanthren-17(14H)-One | SULT2A1 |
| Astragalus mongholicus | MOL831 | (5S,8R,9S,10S,13S,14S)-3-Ethyl-3-Hydroxy-10,13-Dimethyl-Tetradecahydro-2H-Cyclopenta[A]Phenanthren-17(14H)-One | SULT2B1 |
| Astragalus mongholicus | MOL832 | (1S,4S,4Ar,8Ar)-4-Isopropyl-1,6-Dimethyl-1,2,3,4,4A,7,8,8A-Octahydronaphthalen-1-Ol | AKR1C1 |
| Astragalus mongholicus | MOL833 | (1S,4S,4Ar,8Ar)-4-Isopropyl-1,6-Dimethyl-1,2,3,4,4A,7,8,8A-Octahydronaphthalen-1-Ol | AKR1C2 |
| Astragalus mongholicus | MOL834 | (1S,4S,4Ar,8Ar)-4-Isopropyl-1,6-Dimethyl-1,2,3,4,4A,7,8,8A-Octahydronaphthalen-1-Ol | AR |
| Astragalus mongholicus | MOL835 | (1S,4S,4Ar,8Ar)-4-Isopropyl-1,6-Dimethyl-1,2,3,4,4A,7,8,8A-Octahydronaphthalen-1-Ol | CLEC4E |
| Astragalus mongholicus | MOL836 | (1S,4S,4Ar,8Ar)-4-Isopropyl-1,6-Dimethyl-1,2,3,4,4A,7,8,8A-Octahydronaphthalen-1-Ol | ESR1 |
| Astragalus mongholicus | MOL837 | (1S,4S,4Ar,8Ar)-4-Isopropyl-1,6-Dimethyl-1,2,3,4,4A,7,8,8A-Octahydronaphthalen-1-Ol | LSS |
| Astragalus mongholicus | MOL838 | (1S,4S,4Ar,8Ar)-4-Isopropyl-1,6-Dimethyl-1,2,3,4,4A,7,8,8A-Octahydronaphthalen-1-Ol | NCOA2 |
| Astragalus mongholicus | MOL839 | (1S,4S,4Ar,8Ar)-4-Isopropyl-1,6-Dimethyl-1,2,3,4,4A,7,8,8A-Octahydronaphthalen-1-Ol | NR1I2 |
| Astragalus mongholicus | MOL840 | (1S,4S,4Ar,8Ar)-4-Isopropyl-1,6-Dimethyl-1,2,3,4,4A,7,8,8A-Octahydronaphthalen-1-Ol | NR1I3 |
| Astragalus mongholicus | MOL841 | (1S,4S,4Ar,8Ar)-4-Isopropyl-1,6-Dimethyl-1,2,3,4,4A,7,8,8A-Octahydronaphthalen-1-Ol | NR3C2 |
| Astragalus mongholicus | MOL842 | (1S,4S,4Ar,8Ar)-4-Isopropyl-1,6-Dimethyl-1,2,3,4,4A,7,8,8A-Octahydronaphthalen-1-Ol | PGR |
| Astragalus mongholicus | MOL843 | (1S,4S,4Ar,8Ar)-4-Isopropyl-1,6-Dimethyl-1,2,3,4,4A,7,8,8A-Octahydronaphthalen-1-Ol | RORA |
| Astragalus mongholicus | MOL844 | (1S,4S,4Ar,8Ar)-4-Isopropyl-1,6-Dimethyl-1,2,3,4,4A,7,8,8A-Octahydronaphthalen-1-Ol | SULT2B1 |
| Astragalus mongholicus | MOL845 | (1S,4S,4Ar,8Ar)-4-Isopropyl-1,6-Dimethyl-1,2,3,4,4A,7,8,8A-Octahydronaphthalen-1-Ol | VDR |
| Astragalus mongholicus | MOL846 | (1S,4R,4Ar,8Ar)-1-Isopropyl-4,7-Dimethyl-1,2,3,4,4A,5,6,8A-Octahydronaphthalen-4A-Ol | AKR1C1 |
| Astragalus mongholicus | MOL847 | (1S,4R,4Ar,8Ar)-1-Isopropyl-4,7-Dimethyl-1,2,3,4,4A,5,6,8A-Octahydronaphthalen-4A-Ol | AKR1C2 |
| Astragalus mongholicus | MOL848 | (1S,4R,4Ar,8Ar)-1-Isopropyl-4,7-Dimethyl-1,2,3,4,4A,5,6,8A-Octahydronaphthalen-4A-Ol | AR |
| Astragalus mongholicus | MOL849 | (1S,4R,4Ar,8Ar)-1-Isopropyl-4,7-Dimethyl-1,2,3,4,4A,5,6,8A-Octahydronaphthalen-4A-Ol | CLEC4E |
| Astragalus mongholicus | MOL850 | (1S,4R,4Ar,8Ar)-1-Isopropyl-4,7-Dimethyl-1,2,3,4,4A,5,6,8A-Octahydronaphthalen-4A-Ol | ESR1 |
| Astragalus mongholicus | MOL851 | (1S,4R,4Ar,8Ar)-1-Isopropyl-4,7-Dimethyl-1,2,3,4,4A,5,6,8A-Octahydronaphthalen-4A-Ol | ESR2 |
| Astragalus mongholicus | MOL852 | (1S,4R,4Ar,8Ar)-1-Isopropyl-4,7-Dimethyl-1,2,3,4,4A,5,6,8A-Octahydronaphthalen-4A-Ol | GABRA1 |
| Astragalus mongholicus | MOL853 | (1S,4R,4Ar,8Ar)-1-Isopropyl-4,7-Dimethyl-1,2,3,4,4A,5,6,8A-Octahydronaphthalen-4A-Ol | GABRA2 |
| Astragalus mongholicus | MOL854 | (1S,4R,4Ar,8Ar)-1-Isopropyl-4,7-Dimethyl-1,2,3,4,4A,5,6,8A-Octahydronaphthalen-4A-Ol | GABRA3 |
| Astragalus mongholicus | MOL855 | (1S,4R,4Ar,8Ar)-1-Isopropyl-4,7-Dimethyl-1,2,3,4,4A,5,6,8A-Octahydronaphthalen-4A-Ol | GABRA4 |
| Astragalus mongholicus | MOL856 | (1S,4R,4Ar,8Ar)-1-Isopropyl-4,7-Dimethyl-1,2,3,4,4A,5,6,8A-Octahydronaphthalen-4A-Ol | GABRA5 |
| Astragalus mongholicus | MOL857 | (1S,4R,4Ar,8Ar)-1-Isopropyl-4,7-Dimethyl-1,2,3,4,4A,5,6,8A-Octahydronaphthalen-4A-Ol | GABRA6 |
| Astragalus mongholicus | MOL858 | (1S,4R,4Ar,8Ar)-1-Isopropyl-4,7-Dimethyl-1,2,3,4,4A,5,6,8A-Octahydronaphthalen-4A-Ol | GABRB1 |
| Astragalus mongholicus | MOL859 | (1S,4R,4Ar,8Ar)-1-Isopropyl-4,7-Dimethyl-1,2,3,4,4A,5,6,8A-Octahydronaphthalen-4A-Ol | GABRB2 |
| Astragalus mongholicus | MOL860 | (1S,4R,4Ar,8Ar)-1-Isopropyl-4,7-Dimethyl-1,2,3,4,4A,5,6,8A-Octahydronaphthalen-4A-Ol | GABRB3 |
| Astragalus mongholicus | MOL861 | (1S,4R,4Ar,8Ar)-1-Isopropyl-4,7-Dimethyl-1,2,3,4,4A,5,6,8A-Octahydronaphthalen-4A-Ol | GABRD |
| Astragalus mongholicus | MOL862 | (1S,4R,4Ar,8Ar)-1-Isopropyl-4,7-Dimethyl-1,2,3,4,4A,5,6,8A-Octahydronaphthalen-4A-Ol | GABRE |
| Astragalus mongholicus | MOL863 | (1S,4R,4Ar,8Ar)-1-Isopropyl-4,7-Dimethyl-1,2,3,4,4A,5,6,8A-Octahydronaphthalen-4A-Ol | GABRG1 |
| Astragalus mongholicus | MOL864 | (1S,4R,4Ar,8Ar)-1-Isopropyl-4,7-Dimethyl-1,2,3,4,4A,5,6,8A-Octahydronaphthalen-4A-Ol | GABRG2 |
| Astragalus mongholicus | MOL865 | (1S,4R,4Ar,8Ar)-1-Isopropyl-4,7-Dimethyl-1,2,3,4,4A,5,6,8A-Octahydronaphthalen-4A-Ol | GABRG3 |
| Astragalus mongholicus | MOL866 | (1S,4R,4Ar,8Ar)-1-Isopropyl-4,7-Dimethyl-1,2,3,4,4A,5,6,8A-Octahydronaphthalen-4A-Ol | GABRP |
| Astragalus mongholicus | MOL867 | (1S,4R,4Ar,8Ar)-1-Isopropyl-4,7-Dimethyl-1,2,3,4,4A,5,6,8A-Octahydronaphthalen-4A-Ol | GABRQ |
| Astragalus mongholicus | MOL868 | (1S,4R,4Ar,8Ar)-1-Isopropyl-4,7-Dimethyl-1,2,3,4,4A,5,6,8A-Octahydronaphthalen-4A-Ol | GRIN1 |
| Astragalus mongholicus | MOL869 | (1S,4R,4Ar,8Ar)-1-Isopropyl-4,7-Dimethyl-1,2,3,4,4A,5,6,8A-Octahydronaphthalen-4A-Ol | GRIN2A |
| Astragalus mongholicus | MOL870 | (1S,4R,4Ar,8Ar)-1-Isopropyl-4,7-Dimethyl-1,2,3,4,4A,5,6,8A-Octahydronaphthalen-4A-Ol | GRIN2B |
| Astragalus mongholicus | MOL871 | (1S,4R,4Ar,8Ar)-1-Isopropyl-4,7-Dimethyl-1,2,3,4,4A,5,6,8A-Octahydronaphthalen-4A-Ol | GRIN2C |
| Astragalus mongholicus | MOL872 | (1S,4R,4Ar,8Ar)-1-Isopropyl-4,7-Dimethyl-1,2,3,4,4A,5,6,8A-Octahydronaphthalen-4A-Ol | GRIN2D |
| Astragalus mongholicus | MOL873 | (1S,4R,4Ar,8Ar)-1-Isopropyl-4,7-Dimethyl-1,2,3,4,4A,5,6,8A-Octahydronaphthalen-4A-Ol | GRIN3A |
| Astragalus mongholicus | MOL874 | (1S,4R,4Ar,8Ar)-1-Isopropyl-4,7-Dimethyl-1,2,3,4,4A,5,6,8A-Octahydronaphthalen-4A-Ol | GRIN3B |
| Astragalus mongholicus | MOL875 | (1S,4R,4Ar,8Ar)-1-Isopropyl-4,7-Dimethyl-1,2,3,4,4A,5,6,8A-Octahydronaphthalen-4A-Ol | HOXA10 |
| Astragalus mongholicus | MOL876 | (1S,4R,4Ar,8Ar)-1-Isopropyl-4,7-Dimethyl-1,2,3,4,4A,5,6,8A-Octahydronaphthalen-4A-Ol | HSD17B1 |
| Astragalus mongholicus | MOL877 | (1S,4R,4Ar,8Ar)-1-Isopropyl-4,7-Dimethyl-1,2,3,4,4A,5,6,8A-Octahydronaphthalen-4A-Ol | LSS |
| Astragalus mongholicus | MOL878 | (1S,4R,4Ar,8Ar)-1-Isopropyl-4,7-Dimethyl-1,2,3,4,4A,5,6,8A-Octahydronaphthalen-4A-Ol | NCOA2 |
| Astragalus mongholicus | MOL879 | (1S,4R,4Ar,8Ar)-1-Isopropyl-4,7-Dimethyl-1,2,3,4,4A,5,6,8A-Octahydronaphthalen-4A-Ol | NPPB |
| Astragalus mongholicus | MOL880 | (1S,4R,4Ar,8Ar)-1-Isopropyl-4,7-Dimethyl-1,2,3,4,4A,5,6,8A-Octahydronaphthalen-4A-Ol | NR1I2 |
| Astragalus mongholicus | MOL881 | (1S,4R,4Ar,8Ar)-1-Isopropyl-4,7-Dimethyl-1,2,3,4,4A,5,6,8A-Octahydronaphthalen-4A-Ol | NR1I3 |
| Astragalus mongholicus | MOL882 | (1S,4R,4Ar,8Ar)-1-Isopropyl-4,7-Dimethyl-1,2,3,4,4A,5,6,8A-Octahydronaphthalen-4A-Ol | NR3C2 |
| Astragalus mongholicus | MOL883 | (1S,4R,4Ar,8Ar)-1-Isopropyl-4,7-Dimethyl-1,2,3,4,4A,5,6,8A-Octahydronaphthalen-4A-Ol | PGR |
| Astragalus mongholicus | MOL884 | (1S,4R,4Ar,8Ar)-1-Isopropyl-4,7-Dimethyl-1,2,3,4,4A,5,6,8A-Octahydronaphthalen-4A-Ol | PPARA |
| Astragalus mongholicus | MOL885 | (1S,4R,4Ar,8Ar)-1-Isopropyl-4,7-Dimethyl-1,2,3,4,4A,5,6,8A-Octahydronaphthalen-4A-Ol | RORA |
| Astragalus mongholicus | MOL886 | (1S,4R,4Ar,8Ar)-1-Isopropyl-4,7-Dimethyl-1,2,3,4,4A,5,6,8A-Octahydronaphthalen-4A-Ol | SIGMAR1 |
| Astragalus mongholicus | MOL887 | (1S,4R,4Ar,8Ar)-1-Isopropyl-4,7-Dimethyl-1,2,3,4,4A,5,6,8A-Octahydronaphthalen-4A-Ol | SULT2A1 |
| Astragalus mongholicus | MOL888 | (1S,4R,4Ar,8Ar)-1-Isopropyl-4,7-Dimethyl-1,2,3,4,4A,5,6,8A-Octahydronaphthalen-4A-Ol | SULT2B1 |
| Astragalus mongholicus | MOL889 | (1S,4R,4Ar,8Ar)-1-Isopropyl-4,7-Dimethyl-1,2,3,4,4A,5,6,8A-Octahydronaphthalen-4A-Ol | VDR |
| Astragalus mongholicus | MOL890 | (1Ar,7S,7As,7Br,Z)-1,1,4,7-Tetramethyl-1A,2,3,5,6,7,7A,7B-Octahydro-1H-Cyclopropa[E]Azulen-7-Ol | CLEC4E |
| Astragalus mongholicus | MOL891 | (1Ar,7S,7As,7Br,Z)-1,1,4,7-Tetramethyl-1A,2,3,5,6,7,7A,7B-Octahydro-1H-Cyclopropa[E]Azulen-7-Ol | LSS |
| Astragalus mongholicus | MOL892 | (1Ar,7S,7As,7Br,Z)-1,1,4,7-Tetramethyl-1A,2,3,5,6,7,7A,7B-Octahydro-1H-Cyclopropa[E]Azulen-7-Ol | NR1I3 |
| Astragalus mongholicus | MOL893 | (1Ar,7S,7As,7Br,Z)-1,1,4,7-Tetramethyl-1A,2,3,5,6,7,7A,7B-Octahydro-1H-Cyclopropa[E]Azulen-7-Ol | RORA |
| Astragalus mongholicus | MOL894 | (1Ar,7S,7As,7Br,Z)-1,1,4,7-Tetramethyl-1A,2,3,5,6,7,7A,7B-Octahydro-1H-Cyclopropa[E]Azulen-7-Ol | VDR |
| Astragalus mongholicus | MOL895 | (3Z,7Z)-1,5,5,8-Tetramethyl-12-Oxa-Bicyclo[9.1.0]Dodeca-3,7-Diene | LCN9 |
| Astragalus mongholicus | MOL896 | (3S,3As,8R,8Ar)-3,8-Dihydroxy-3,8-Dimethyl-5-(Propan-2-Ylidene)-Hexahydroazulen-6(1H,2H,7H)-One | AR |
| Astragalus mongholicus | MOL897 | (3S,3As,8R,8Ar)-3,8-Dihydroxy-3,8-Dimethyl-5-(Propan-2-Ylidene)-Hexahydroazulen-6(1H,2H,7H)-One | ESR1 |
| Astragalus mongholicus | MOL898 | (3S,3As,8R,8Ar)-3,8-Dihydroxy-3,8-Dimethyl-5-(Propan-2-Ylidene)-Hexahydroazulen-6(1H,2H,7H)-One | HOXA10 |
| Astragalus mongholicus | MOL899 | (3S,3As,8R,8Ar)-3,8-Dihydroxy-3,8-Dimethyl-5-(Propan-2-Ylidene)-Hexahydroazulen-6(1H,2H,7H)-One | HSD11B2 |
| Astragalus mongholicus | MOL900 | (3S,3As,8R,8Ar)-3,8-Dihydroxy-3,8-Dimethyl-5-(Propan-2-Ylidene)-Hexahydroazulen-6(1H,2H,7H)-One | NCOA1 |
| Astragalus mongholicus | MOL901 | (3S,3As,8R,8Ar)-3,8-Dihydroxy-3,8-Dimethyl-5-(Propan-2-Ylidene)-Hexahydroazulen-6(1H,2H,7H)-One | NPPB |
| Astragalus mongholicus | MOL902 | (3S,3As,8R,8Ar)-3,8-Dihydroxy-3,8-Dimethyl-5-(Propan-2-Ylidene)-Hexahydroazulen-6(1H,2H,7H)-One | VDR |
| Astragalus mongholicus | MOL903 | (3S,3Ar,8Ar,Z)-3,8A-Dihydroxy-3,8-Dimethyl-5-(Propan-2-Ylidene)-1,2,3,3A,4,5-Hexahydroazulen-6(8Ah)-One | AR |
| Astragalus mongholicus | MOL904 | (3S,3Ar,8Ar,Z)-3,8A-Dihydroxy-3,8-Dimethyl-5-(Propan-2-Ylidene)-1,2,3,3A,4,5-Hexahydroazulen-6(8Ah)-One | CYP27B1 |
| Astragalus mongholicus | MOL905 | (3S,3Ar,8Ar,Z)-3,8A-Dihydroxy-3,8-Dimethyl-5-(Propan-2-Ylidene)-1,2,3,3A,4,5-Hexahydroazulen-6(8Ah)-One | HOXA10 |
| Astragalus mongholicus | MOL906 | (3S,3Ar,8Ar,Z)-3,8A-Dihydroxy-3,8-Dimethyl-5-(Propan-2-Ylidene)-1,2,3,3A,4,5-Hexahydroazulen-6(8Ah)-One | HSD11B2 |
| Astragalus mongholicus | MOL907 | (3S,3Ar,8Ar,Z)-3,8A-Dihydroxy-3,8-Dimethyl-5-(Propan-2-Ylidene)-1,2,3,3A,4,5-Hexahydroazulen-6(8Ah)-One | NCOA1 |
| Astragalus mongholicus | MOL908 | (3S,3Ar,8Ar,Z)-3,8A-Dihydroxy-3,8-Dimethyl-5-(Propan-2-Ylidene)-1,2,3,3A,4,5-Hexahydroazulen-6(8Ah)-One | NPPB |
| Astragalus mongholicus | MOL909 | (3S,3Ar,8Ar,Z)-3,8A-Dihydroxy-3,8-Dimethyl-5-(Propan-2-Ylidene)-1,2,3,3A,4,5-Hexahydroazulen-6(8Ah)-One | RXRA |
| Astragalus mongholicus | MOL910 | (3S,3Ar,8Ar,Z)-3,8A-Dihydroxy-3,8-Dimethyl-5-(Propan-2-Ylidene)-1,2,3,3A,4,5-Hexahydroazulen-6(8Ah)-One | VDR |
| Astragalus mongholicus | MOL911 | (3S,3As,5S,8As)-3A-Hydroxy-3,3',3',8-Tetramethyl-1,2,3,3A,4,8A-Hexahydro-6H-Spiro[Azulene-5,2'-Oxiran]-6-One | ESR1 |
| Astragalus mongholicus | MOL912 | (3S,3As,5S,8As)-3A-Hydroxy-3,3',3',8-Tetramethyl-1,2,3,3A,4,8A-Hexahydro-6H-Spiro[Azulene-5,2'-Oxiran]-6-One | ESR2 |
| Astragalus mongholicus | MOL913 | (3S,3As,5S,8As)-3A-Hydroxy-3,3',3',8-Tetramethyl-1,2,3,3A,4,8A-Hexahydro-6H-Spiro[Azulene-5,2'-Oxiran]-6-One | HSD3B1 |
| Astragalus mongholicus | MOL914 | (3S,3As,5S,8As)-3A-Hydroxy-3,3',3',8-Tetramethyl-1,2,3,3A,4,8A-Hexahydro-6H-Spiro[Azulene-5,2'-Oxiran]-6-One | HSD3B2 |
| Astragalus mongholicus | MOL915 | (4Ar,5R,5As,6Ar)-6A-Hydroxy-3,5A-Dimethyl-5-(3-Oxobutyl)-4,4A,5,5A,6,6A-Hexahydro-2H-Cyclopropa[F][1]Benzofuran-2-One | AR |
| Astragalus mongholicus | MOL916 | (4Ar,5R,5As,6Ar)-6A-Hydroxy-3,5A-Dimethyl-5-(3-Oxobutyl)-4,4A,5,5A,6,6A-Hexahydro-2H-Cyclopropa[F][1]Benzofuran-2-One | IGHG2 |
| Astragalus mongholicus | MOL917 | (4Ar,5R,5As,6Ar)-6A-Hydroxy-3,5A-Dimethyl-5-(3-Oxobutyl)-4,4A,5,5A,6,6A-Hexahydro-2H-Cyclopropa[F][1]Benzofuran-2-One | NR3C2 |
| Astragalus mongholicus | MOL918 | (4Ar,5R,5As,6Ar)-6A-Hydroxy-3,5A-Dimethyl-5-(3-Oxobutyl)-4,4A,5,5A,6,6A-Hexahydro-2H-Cyclopropa[F][1]Benzofuran-2-One | PGR |
| Astragalus mongholicus | MOL919 | (4Ar,5R,5As,6As)-3,5A-Dimethyl-5-(3-Oxobutyl)-4,4A,5,5A,6,6A-Hexahydro-2H-Cyclopropa[F][1]Benzofuran-2-One | AR |
| Astragalus mongholicus | MOL920 | (4Ar,5R,5As,6As)-3,5A-Dimethyl-5-(3-Oxobutyl)-4,4A,5,5A,6,6A-Hexahydro-2H-Cyclopropa[F][1]Benzofuran-2-One | ESR1 |
| Astragalus mongholicus | MOL921 | (4Ar,5R,5As,6As)-3,5A-Dimethyl-5-(3-Oxobutyl)-4,4A,5,5A,6,6A-Hexahydro-2H-Cyclopropa[F][1]Benzofuran-2-One | NR3C2 |
| Astragalus mongholicus | MOL922 | (4Ar,5R,5As,6As)-3,5A-Dimethyl-5-(3-Oxobutyl)-4,4A,5,5A,6,6A-Hexahydro-2H-Cyclopropa[F][1]Benzofuran-2-One | PGR |

**Supplementary table 4b. CPV of the active ingredients from ETCM database and relevant literature.**

| Drug | MolId | MolName | Symbol |
| --- | --- | --- | --- |
| Curcuma phaeocaulis Valeton | MOL01 | 1-Isopropyl-4-Methylcyclohex-3-Enol,4-Terpineol | VDR |
| Curcuma phaeocaulis Valeton | MOL02 | Ethyl3-(4-Methoxyphenyl)Acrylate | RARA |
| Curcuma phaeocaulis Valeton | MOL03 | Ethyl3-(4-Methoxyphenyl)Acrylate | RARB |
| Curcuma phaeocaulis Valeton | MOL04 | Ethyl3-(4-Methoxyphenyl)Acrylate | RARG |
| Curcuma phaeocaulis Valeton | MOL05 | Ethyl3-(4-Methoxyphenyl)Acrylate | RXRA |
| Curcuma phaeocaulis Valeton | MOL06 | Ethyl3-(4-Methoxyphenyl)Acrylate | RXRB |
| Curcuma phaeocaulis Valeton | MOL07 | Ethyl3-(4-Methoxyphenyl)Acrylate | RXRG |
| Curcuma phaeocaulis Valeton | MOL08 | 1,7-Bis(4-Hydroxyphenyl)Hepta-1,6-Diene-3,5-Dione | PYGM |
| Curcuma phaeocaulis Valeton | MOL09 | (2,6-Dihydroxy-4-Methoxyphenyl)((1R,2S,6R)-3-Methyl-2-(3-Methylbut-2-Enyl)-6-Phenylcyclohex-3-Enyl)Methanone | PLA2G2E |
| Curcuma phaeocaulis Valeton | MOL10 | (2,4-Dihydroxy-6-Methoxyphenyl)(3-Methyl-2-(3-Methylbut-2-Enyl)-6-Phenylcyclohex-3-Enyl)Methanone | PLA2G2E |
| Curcuma phaeocaulis Valeton | MOL11 | (1R,2S,5S)-5-Isopropyl-2-Methylbicyclo[3.1.0]Hexan-2-Ol | NR1I3 |
| Curcuma phaeocaulis Valeton | MOL12 | Ethyl3-Phenylacrylate | NPRS |
| Curcuma phaeocaulis Valeton | MOL13 | Methanol | MB |
| Curcuma phaeocaulis Valeton | MOL14 | (Trans-Trans)Farnesol,3,7,11-Trimethyldodeca-2,6,10-Trien-1-Ol,Farnesol | MAOB |
| Curcuma phaeocaulis Valeton | MOL15 | (Trans-Trans)Farnesol,3,7,11-Trimethyldodeca-2,6,10-Trien-1-Ol,Farnesol | NR1H4 |
| Curcuma phaeocaulis Valeton | MOL16 | (S)-2-Methyl-6-(4-Methylcyclohex-3-En-1-Yl)Hepta-2,6-Dien-1-Ol | MAOB |
| Curcuma phaeocaulis Valeton | MOL17 | (S)-2-Methyl-6-(4-Methylcyclohex-3-En-1-Yl)Hepta-2,6-Dien-1-Ol | NR1H4 |
| Curcuma phaeocaulis Valeton | MOL18 | 2,4-Dimethyloctan-4-Ol | LCN9 |
| Curcuma phaeocaulis Valeton | MOL19 | (-)-CaryophylleneOxide,(1R,4R,6R,10S)-4,12,12-Trimethyl-9-Methylidene-5-Oxatricyclo[8.2.0.0~4,6~]Dodecane | LCN9 |
| Curcuma phaeocaulis Valeton | MOL20 | (1R,11R)-1,5,9,9-Tetramethyl-12-Oxabicyclo[9.1.0]Dodeca-4,7-Diene | LCN9 |
| Curcuma phaeocaulis Valeton | MOL21 | (1R,11R)-1,5,5,8-Tetramethyl-12-Oxabicyclo[9.1.0]Dodeca-3,7-Diene | LCN9 |
| Curcuma phaeocaulis Valeton | MOL22 | (3Z,7Z)-3,7,10,10-Tetramethyl-12-Oxa-Bicyclo[9.1.0]Dodeca-3,7-Diene | LCN9 |
| Curcuma phaeocaulis Valeton | MOL23 | (3Z,7Z)-1,5,5,8-Tetramethyl-12-Oxa-Bicyclo[9.1.0]Dodeca-3,7-Diene | LCN9 |
| Curcuma phaeocaulis Valeton | MOL24 | (3S,3As,5S,8As)-3A-Hydroxy-3,3',3',8-Tetramethyl-1,2,3,3A,4,8A-Hexahydro-6H-Spiro[Azulene-5,2'-Oxiran]-6-One | ESR1 |
| Curcuma phaeocaulis Valeton | MOL25 | (3S,3As,5S,8As)-3A-Hydroxy-3,3',3',8-Tetramethyl-1,2,3,3A,4,8A-Hexahydro-6H-Spiro[Azulene-5,2'-Oxiran]-6-One | ESR2 |
| Curcuma phaeocaulis Valeton | MOL26 | (3S,3As,5S,8As)-3A-Hydroxy-3,3',3',8-Tetramethyl-1,2,3,3A,4,8A-Hexahydro-6H-Spiro[Azulene-5,2'-Oxiran]-6-One | HSD3B1 |
| Curcuma phaeocaulis Valeton | MOL27 | (3S,3As,5S,8As)-3A-Hydroxy-3,3',3',8-Tetramethyl-1,2,3,3A,4,8A-Hexahydro-6H-Spiro[Azulene-5,2'-Oxiran]-6-One | HSD3B2 |
| Curcuma phaeocaulis Valeton | MOL28 | (1Ar,7S,7As,7Br,Z)-1,1,4,7-Tetramethyl-1A,2,3,5,6,7,7A,7B-Octahydro-1H-Cyclopropa[E]Azulen-7-Ol | CLEC4E |
| Curcuma phaeocaulis Valeton | MOL29 | (1Ar,7S,7As,7Br,Z)-1,1,4,7-Tetramethyl-1A,2,3,5,6,7,7A,7B-Octahydro-1H-Cyclopropa[E]Azulen-7-Ol | LSS |
| Curcuma phaeocaulis Valeton | MOL30 | (1Ar,7S,7As,7Br,Z)-1,1,4,7-Tetramethyl-1A,2,3,5,6,7,7A,7B-Octahydro-1H-Cyclopropa[E]Azulen-7-Ol | NR1I3 |
| Curcuma phaeocaulis Valeton | MOL31 | (1Ar,7S,7As,7Br,Z)-1,1,4,7-Tetramethyl-1A,2,3,5,6,7,7A,7B-Octahydro-1H-Cyclopropa[E]Azulen-7-Ol | RORA |
| Curcuma phaeocaulis Valeton | MOL32 | (1Ar,7S,7As,7Br,Z)-1,1,4,7-Tetramethyl-1A,2,3,5,6,7,7A,7B-Octahydro-1H-Cyclopropa[E]Azulen-7-Ol | VDR |
| Curcuma phaeocaulis Valeton | MOL33 | (4Ar,5R,5As,6As)-3,5A-Dimethyl-5-(3-Oxobutyl)-4,4A,5,5A,6,6A-Hexahydro-2H-Cyclopropa[F][1]Benzofuran-2-One | AR |
| Curcuma phaeocaulis Valeton | MOL34 | (4Ar,5R,5As,6As)-3,5A-Dimethyl-5-(3-Oxobutyl)-4,4A,5,5A,6,6A-Hexahydro-2H-Cyclopropa[F][1]Benzofuran-2-One | ESR1 |
| Curcuma phaeocaulis Valeton | MOL35 | (4Ar,5R,5As,6As)-3,5A-Dimethyl-5-(3-Oxobutyl)-4,4A,5,5A,6,6A-Hexahydro-2H-Cyclopropa[F][1]Benzofuran-2-One | NR3C2 |
| Curcuma phaeocaulis Valeton | MOL36 | (4Ar,5R,5As,6As)-3,5A-Dimethyl-5-(3-Oxobutyl)-4,4A,5,5A,6,6A-Hexahydro-2H-Cyclopropa[F][1]Benzofuran-2-One | PGR |
| Curcuma phaeocaulis Valeton | MOL37 | (3S,3As,8R,8Ar)-3,8-Dihydroxy-3,8-Dimethyl-5-(Propan-2-Ylidene)-Hexahydroazulen-6(1H,2H,7H)-One | AR |
| Curcuma phaeocaulis Valeton | MOL38 | (3S,3As,8R,8Ar)-3,8-Dihydroxy-3,8-Dimethyl-5-(Propan-2-Ylidene)-Hexahydroazulen-6(1H,2H,7H)-One | ESR1 |
| Curcuma phaeocaulis Valeton | MOL39 | (3S,3As,8R,8Ar)-3,8-Dihydroxy-3,8-Dimethyl-5-(Propan-2-Ylidene)-Hexahydroazulen-6(1H,2H,7H)-One | HOXA10 |
| Curcuma phaeocaulis Valeton | MOL40 | (3S,3As,8R,8Ar)-3,8-Dihydroxy-3,8-Dimethyl-5-(Propan-2-Ylidene)-Hexahydroazulen-6(1H,2H,7H)-One | HSD11B2 |
| Curcuma phaeocaulis Valeton | MOL41 | (3S,3As,8R,8Ar)-3,8-Dihydroxy-3,8-Dimethyl-5-(Propan-2-Ylidene)-Hexahydroazulen-6(1H,2H,7H)-One | NCOA1 |
| Curcuma phaeocaulis Valeton | MOL42 | (3S,3As,8R,8Ar)-3,8-Dihydroxy-3,8-Dimethyl-5-(Propan-2-Ylidene)-Hexahydroazulen-6(1H,2H,7H)-One | NPPB |
| Curcuma phaeocaulis Valeton | MOL43 | (3S,3As,8R,8Ar)-3,8-Dihydroxy-3,8-Dimethyl-5-(Propan-2-Ylidene)-Hexahydroazulen-6(1H,2H,7H)-One | VDR |
| Curcuma phaeocaulis Valeton | MOL44 | (3S,3Ar,8Ar,Z)-3,8A-Dihydroxy-3,8-Dimethyl-5-(Propan-2-Ylidene)-1,2,3,3A,4,5-Hexahydroazulen-6(8Ah)-One | AR |
| Curcuma phaeocaulis Valeton | MOL45 | (3S,3Ar,8Ar,Z)-3,8A-Dihydroxy-3,8-Dimethyl-5-(Propan-2-Ylidene)-1,2,3,3A,4,5-Hexahydroazulen-6(8Ah)-One | CYP27B1 |
| Curcuma phaeocaulis Valeton | MOL46 | (3S,3Ar,8Ar,Z)-3,8A-Dihydroxy-3,8-Dimethyl-5-(Propan-2-Ylidene)-1,2,3,3A,4,5-Hexahydroazulen-6(8Ah)-One | HOXA10 |
| Curcuma phaeocaulis Valeton | MOL47 | (3S,3Ar,8Ar,Z)-3,8A-Dihydroxy-3,8-Dimethyl-5-(Propan-2-Ylidene)-1,2,3,3A,4,5-Hexahydroazulen-6(8Ah)-One | HSD11B2 |
| Curcuma phaeocaulis Valeton | MOL48 | (3S,3Ar,8Ar,Z)-3,8A-Dihydroxy-3,8-Dimethyl-5-(Propan-2-Ylidene)-1,2,3,3A,4,5-Hexahydroazulen-6(8Ah)-One | NCOA1 |
| Curcuma phaeocaulis Valeton | MOL49 | (3S,3Ar,8Ar,Z)-3,8A-Dihydroxy-3,8-Dimethyl-5-(Propan-2-Ylidene)-1,2,3,3A,4,5-Hexahydroazulen-6(8Ah)-One | NPPB |
| Curcuma phaeocaulis Valeton | MOL50 | (3S,3Ar,8Ar,Z)-3,8A-Dihydroxy-3,8-Dimethyl-5-(Propan-2-Ylidene)-1,2,3,3A,4,5-Hexahydroazulen-6(8Ah)-One | RXRA |
| Curcuma phaeocaulis Valeton | MOL51 | (3S,3Ar,8Ar,Z)-3,8A-Dihydroxy-3,8-Dimethyl-5-(Propan-2-Ylidene)-1,2,3,3A,4,5-Hexahydroazulen-6(8Ah)-One | VDR |
| Curcuma phaeocaulis Valeton | MOL52 | 1,7,7-Trimethylbicyclo[2.2.1]Heptan-2-YlAcetate,BornylAcetate | AR |
| Curcuma phaeocaulis Valeton | MOL53 | 1,7,7-Trimethylbicyclo[2.2.1]Heptan-2-YlAcetate,BornylAcetate | ESR1 |
| Curcuma phaeocaulis Valeton | MOL54 | 1,7,7-Trimethylbicyclo[2.2.1]Heptan-2-YlAcetate,BornylAcetate | HSD17B1 |
| Curcuma phaeocaulis Valeton | MOL55 | 1,7,7-Trimethylbicyclo[2.2.1]Heptan-2-YlAcetate,BornylAcetate | NR3C2 |
| Curcuma phaeocaulis Valeton | MOL56 | 1,7,7-Trimethylbicyclo[2.2.1]Heptan-2-YlAcetate,BornylAcetate | TRPA1 |
| Curcuma phaeocaulis Valeton | MOL57 | 1,7,7-Trimethylbicyclo[2.2.1]Heptan-2-YlAcetate,BornylAcetate | TRPM8 |
| Curcuma phaeocaulis Valeton | MOL58 | 1,7,7-Trimethylbicyclo[2.2.1]Heptan-2-YlAcetate,BornylAcetate | TRPV1 |
| Curcuma phaeocaulis Valeton | MOL59 | 1,7,7-Trimethylbicyclo[2.2.1]Heptan-2-YlAcetate,BornylAcetate | TRPV3 |
| Curcuma phaeocaulis Valeton | MOL60 | 1-(2,4-Dihydroxy-6-Methoxyphenyl)-3-Phenylprop-2-En-1-One,Cardamonin | AR |
| Curcuma phaeocaulis Valeton | MOL61 | 1-(2,4-Dihydroxy-6-Methoxyphenyl)-3-Phenylprop-2-En-1-One,Cardamonin | CTRB1 |
| Curcuma phaeocaulis Valeton | MOL62 | 1-(2,4-Dihydroxy-6-Methoxyphenyl)-3-Phenylprop-2-En-1-One,Cardamonin | ESR1 |
| Curcuma phaeocaulis Valeton | MOL63 | 1-(2,4-Dihydroxy-6-Methoxyphenyl)-3-Phenylprop-2-En-1-One,Cardamonin | ESR2 |
| Curcuma phaeocaulis Valeton | MOL64 | 1-(2,4-Dihydroxy-6-Methoxyphenyl)-3-Phenylprop-2-En-1-One,Cardamonin | PGR |
| Curcuma phaeocaulis Valeton | MOL65 | 1-(2,4-Dihydroxy-6-Methoxyphenyl)-3-Phenylprop-2-En-1-One,Cardamonin | SHBG |
| Curcuma phaeocaulis Valeton | MOL66 | 2,3,3-Trimethyl-2-(3-Methylbuta-1,3-Dien-1-Yl)-6-Methylenecyclohexanone | AR |
| Curcuma phaeocaulis Valeton | MOL67 | 2,3,3-Trimethyl-2-(3-Methylbuta-1,3-Dien-1-Yl)-6-Methylenecyclohexanone | ESR1 |
| Curcuma phaeocaulis Valeton | MOL68 | 2,3,3-Trimethyl-2-(3-Methylbuta-1,3-Dien-1-Yl)-6-Methylenecyclohexanone | ESR2 |
| Curcuma phaeocaulis Valeton | MOL69 | 2,3,3-Trimethyl-2-(3-Methylbuta-1,3-Dien-1-Yl)-6-Methylenecyclohexanone | PGR |
| Curcuma phaeocaulis Valeton | MOL70 | (8S,8As)-8-Hydroxy-3,5,8A-Trimethyl-7,8,8A,9-Tetrahydronaphtho[2,3-B]Furan-4(6H)-One | AR |
| Curcuma phaeocaulis Valeton | MOL71 | (8S,8As)-8-Hydroxy-3,5,8A-Trimethyl-7,8,8A,9-Tetrahydronaphtho[2,3-B]Furan-4(6H)-One | ESR1 |
| Curcuma phaeocaulis Valeton | MOL72 | (8S,8As)-8-Hydroxy-3,5,8A-Trimethyl-7,8,8A,9-Tetrahydronaphtho[2,3-B]Furan-4(6H)-One | ESR2 |
| Curcuma phaeocaulis Valeton | MOL73 | (8S,8As)-8-Hydroxy-3,5,8A-Trimethyl-7,8,8A,9-Tetrahydronaphtho[2,3-B]Furan-4(6H)-One | GABRA1 |
| Curcuma phaeocaulis Valeton | MOL74 | (8S,8As)-8-Hydroxy-3,5,8A-Trimethyl-7,8,8A,9-Tetrahydronaphtho[2,3-B]Furan-4(6H)-One | GABRA2 |
| Curcuma phaeocaulis Valeton | MOL75 | (8S,8As)-8-Hydroxy-3,5,8A-Trimethyl-7,8,8A,9-Tetrahydronaphtho[2,3-B]Furan-4(6H)-One | GABRA3 |
| Curcuma phaeocaulis Valeton | MOL76 | (8S,8As)-8-Hydroxy-3,5,8A-Trimethyl-7,8,8A,9-Tetrahydronaphtho[2,3-B]Furan-4(6H)-One | GABRA4 |
| Curcuma phaeocaulis Valeton | MOL77 | (8S,8As)-8-Hydroxy-3,5,8A-Trimethyl-7,8,8A,9-Tetrahydronaphtho[2,3-B]Furan-4(6H)-One | GABRA5 |
| Curcuma phaeocaulis Valeton | MOL78 | (8S,8As)-8-Hydroxy-3,5,8A-Trimethyl-7,8,8A,9-Tetrahydronaphtho[2,3-B]Furan-4(6H)-One | GABRA6 |
| Curcuma phaeocaulis Valeton | MOL79 | (8S,8As)-8-Hydroxy-3,5,8A-Trimethyl-7,8,8A,9-Tetrahydronaphtho[2,3-B]Furan-4(6H)-One | GABRB1 |
| Curcuma phaeocaulis Valeton | MOL80 | (8S,8As)-8-Hydroxy-3,5,8A-Trimethyl-7,8,8A,9-Tetrahydronaphtho[2,3-B]Furan-4(6H)-One | GABRB2 |
| Curcuma phaeocaulis Valeton | MOL81 | (8S,8As)-8-Hydroxy-3,5,8A-Trimethyl-7,8,8A,9-Tetrahydronaphtho[2,3-B]Furan-4(6H)-One | GABRB3 |
| Curcuma phaeocaulis Valeton | MOL82 | (8S,8As)-8-Hydroxy-3,5,8A-Trimethyl-7,8,8A,9-Tetrahydronaphtho[2,3-B]Furan-4(6H)-One | GABRD |
| Curcuma phaeocaulis Valeton | MOL83 | (8S,8As)-8-Hydroxy-3,5,8A-Trimethyl-7,8,8A,9-Tetrahydronaphtho[2,3-B]Furan-4(6H)-One | GABRE |
| Curcuma phaeocaulis Valeton | MOL84 | (8S,8As)-8-Hydroxy-3,5,8A-Trimethyl-7,8,8A,9-Tetrahydronaphtho[2,3-B]Furan-4(6H)-One | GABRG1 |
| Curcuma phaeocaulis Valeton | MOL85 | (8S,8As)-8-Hydroxy-3,5,8A-Trimethyl-7,8,8A,9-Tetrahydronaphtho[2,3-B]Furan-4(6H)-One | GABRG2 |
| Curcuma phaeocaulis Valeton | MOL86 | (8S,8As)-8-Hydroxy-3,5,8A-Trimethyl-7,8,8A,9-Tetrahydronaphtho[2,3-B]Furan-4(6H)-One | GABRG3 |
| Curcuma phaeocaulis Valeton | MOL87 | (8S,8As)-8-Hydroxy-3,5,8A-Trimethyl-7,8,8A,9-Tetrahydronaphtho[2,3-B]Furan-4(6H)-One | GABRP |
| Curcuma phaeocaulis Valeton | MOL88 | (8S,8As)-8-Hydroxy-3,5,8A-Trimethyl-7,8,8A,9-Tetrahydronaphtho[2,3-B]Furan-4(6H)-One | GABRQ |
| Curcuma phaeocaulis Valeton | MOL89 | (8S,8As)-8-Hydroxy-3,5,8A-Trimethyl-7,8,8A,9-Tetrahydronaphtho[2,3-B]Furan-4(6H)-One | GRIN1 |
| Curcuma phaeocaulis Valeton | MOL90 | (8S,8As)-8-Hydroxy-3,5,8A-Trimethyl-7,8,8A,9-Tetrahydronaphtho[2,3-B]Furan-4(6H)-One | GRIN2A |
| Curcuma phaeocaulis Valeton | MOL91 | (8S,8As)-8-Hydroxy-3,5,8A-Trimethyl-7,8,8A,9-Tetrahydronaphtho[2,3-B]Furan-4(6H)-One | GRIN2B |
| Curcuma phaeocaulis Valeton | MOL92 | (8S,8As)-8-Hydroxy-3,5,8A-Trimethyl-7,8,8A,9-Tetrahydronaphtho[2,3-B]Furan-4(6H)-One | GRIN2C |
| Curcuma phaeocaulis Valeton | MOL93 | (8S,8As)-8-Hydroxy-3,5,8A-Trimethyl-7,8,8A,9-Tetrahydronaphtho[2,3-B]Furan-4(6H)-One | GRIN2D |
| Curcuma phaeocaulis Valeton | MOL94 | (8S,8As)-8-Hydroxy-3,5,8A-Trimethyl-7,8,8A,9-Tetrahydronaphtho[2,3-B]Furan-4(6H)-One | GRIN3A |
| Curcuma phaeocaulis Valeton | MOL95 | (8S,8As)-8-Hydroxy-3,5,8A-Trimethyl-7,8,8A,9-Tetrahydronaphtho[2,3-B]Furan-4(6H)-One | GRIN3B |
| Curcuma phaeocaulis Valeton | MOL96 | (8S,8As)-8-Hydroxy-3,5,8A-Trimethyl-7,8,8A,9-Tetrahydronaphtho[2,3-B]Furan-4(6H)-One | HSD11B2 |
| Curcuma phaeocaulis Valeton | MOL97 | (8S,8As)-8-Hydroxy-3,5,8A-Trimethyl-7,8,8A,9-Tetrahydronaphtho[2,3-B]Furan-4(6H)-One | HSD17B1 |
| Curcuma phaeocaulis Valeton | MOL98 | (8S,8As)-8-Hydroxy-3,5,8A-Trimethyl-7,8,8A,9-Tetrahydronaphtho[2,3-B]Furan-4(6H)-One | NPPB |
| Curcuma phaeocaulis Valeton | MOL99 | (8S,8As)-8-Hydroxy-3,5,8A-Trimethyl-7,8,8A,9-Tetrahydronaphtho[2,3-B]Furan-4(6H)-One | NR1I2 |
| Curcuma phaeocaulis Valeton | MOL100 | (8S,8As)-8-Hydroxy-3,5,8A-Trimethyl-7,8,8A,9-Tetrahydronaphtho[2,3-B]Furan-4(6H)-One | NR1I3 |
| Curcuma phaeocaulis Valeton | MOL101 | (8S,8As)-8-Hydroxy-3,5,8A-Trimethyl-7,8,8A,9-Tetrahydronaphtho[2,3-B]Furan-4(6H)-One | PPARA |
| Curcuma phaeocaulis Valeton | MOL102 | (8S,8As)-8-Hydroxy-3,5,8A-Trimethyl-7,8,8A,9-Tetrahydronaphtho[2,3-B]Furan-4(6H)-One | SIGMAR1 |
| Curcuma phaeocaulis Valeton | MOL103 | (8S,8As)-8-Hydroxy-3,5,8A-Trimethyl-7,8,8A,9-Tetrahydronaphtho[2,3-B]Furan-4(6H)-One | SULT2A1 |
| Curcuma phaeocaulis Valeton | MOL104 | (8S,8As)-8-Hydroxy-3,5,8A-Trimethyl-7,8,8A,9-Tetrahydronaphtho[2,3-B]Furan-4(6H)-One | SULT2B1 |
| Curcuma phaeocaulis Valeton | MOL105 | (4Ar,5R,5As,6Ar)-6A-Hydroxy-3,5A-Dimethyl-5-(3-Oxobutyl)-4,4A,5,5A,6,6A-Hexahydro-2H-Cyclopropa[F][1]Benzofuran-2-One | AR |
| Curcuma phaeocaulis Valeton | MOL106 | (4Ar,5R,5As,6Ar)-6A-Hydroxy-3,5A-Dimethyl-5-(3-Oxobutyl)-4,4A,5,5A,6,6A-Hexahydro-2H-Cyclopropa[F][1]Benzofuran-2-One | IGHG2 |
| Curcuma phaeocaulis Valeton | MOL107 | (4Ar,5R,5As,6Ar)-6A-Hydroxy-3,5A-Dimethyl-5-(3-Oxobutyl)-4,4A,5,5A,6,6A-Hexahydro-2H-Cyclopropa[F][1]Benzofuran-2-One | NR3C2 |
| Curcuma phaeocaulis Valeton | MOL108 | (4Ar,5R,5As,6Ar)-6A-Hydroxy-3,5A-Dimethyl-5-(3-Oxobutyl)-4,4A,5,5A,6,6A-Hexahydro-2H-Cyclopropa[F][1]Benzofuran-2-One | PGR |
| Curcuma phaeocaulis Valeton | MOL109 | (4Ar,5R,5As,6Ar)-6A-Hydroxy-3,5A-Dimethyl-5-(3-Oxobutyl)-4,4A,5,5A,6,6A-Hexahydro-2H-Cyclopropa[F][1]Benzofuran-2-One | Camphor |
| Curcuma phaeocaulis Valeton | MOL110 | (4Ar,5R,5As,6Ar)-6A-Hydroxy-3,5A-Dimethyl-5-(3-Oxobutyl)-4,4A,5,5A,6,6A-Hexahydro-2H-Cyclopropa[F][1]Benzofuran-2-One | AKR1D1 |
| Curcuma phaeocaulis Valeton | MOL111 | (4Ar,5R,5As,6Ar)-6A-Hydroxy-3,5A-Dimethyl-5-(3-Oxobutyl)-4,4A,5,5A,6,6A-Hexahydro-2H-Cyclopropa[F][1]Benzofuran-2-One | NCOA1 |
| Curcuma phaeocaulis Valeton | MOL112 | (4Ar,5R,5As,6Ar)-6A-Hydroxy-3,5A-Dimethyl-5-(3-Oxobutyl)-4,4A,5,5A,6,6A-Hexahydro-2H-Cyclopropa[F][1]Benzofuran-2-One | NR1I3 |
| Curcuma phaeocaulis Valeton | MOL113 | (4Ar,5R,5As,6Ar)-6A-Hydroxy-3,5A-Dimethyl-5-(3-Oxobutyl)-4,4A,5,5A,6,6A-Hexahydro-2H-Cyclopropa[F][1]Benzofuran-2-One | RXRA |
| Curcuma phaeocaulis Valeton | MOL114 | (4Ar,5R,5As,6Ar)-6A-Hydroxy-3,5A-Dimethyl-5-(3-Oxobutyl)-4,4A,5,5A,6,6A-Hexahydro-2H-Cyclopropa[F][1]Benzofuran-2-One | TRPA1 |
| Curcuma phaeocaulis Valeton | MOL115 | (4Ar,5R,5As,6Ar)-6A-Hydroxy-3,5A-Dimethyl-5-(3-Oxobutyl)-4,4A,5,5A,6,6A-Hexahydro-2H-Cyclopropa[F][1]Benzofuran-2-One | TRPM8 |
| Curcuma phaeocaulis Valeton | MOL116 | (4Ar,5R,5As,6Ar)-6A-Hydroxy-3,5A-Dimethyl-5-(3-Oxobutyl)-4,4A,5,5A,6,6A-Hexahydro-2H-Cyclopropa[F][1]Benzofuran-2-One | TRPV1 |
| Curcuma phaeocaulis Valeton | MOL117 | (4Ar,5R,5As,6Ar)-6A-Hydroxy-3,5A-Dimethyl-5-(3-Oxobutyl)-4,4A,5,5A,6,6A-Hexahydro-2H-Cyclopropa[F][1]Benzofuran-2-One | TRPV3 |
| Curcuma phaeocaulis Valeton | MOL118 | 1,7,7-Trimethylbicyclo[2.2.1]Heptan-2-Ol,Borneol | AKR1D1 |
| Curcuma phaeocaulis Valeton | MOL119 | 1,7,7-Trimethylbicyclo[2.2.1]Heptan-2-Ol,Borneol | AR |
| Curcuma phaeocaulis Valeton | MOL120 | 1,7,7-Trimethylbicyclo[2.2.1]Heptan-2-Ol,Borneol | CACNA1C |
| Curcuma phaeocaulis Valeton | MOL121 | 1,7,7-Trimethylbicyclo[2.2.1]Heptan-2-Ol,Borneol | CACNA1D |
| Curcuma phaeocaulis Valeton | MOL122 | 1,7,7-Trimethylbicyclo[2.2.1]Heptan-2-Ol,Borneol | CACNA1F |
| Curcuma phaeocaulis Valeton | MOL123 | 1,7,7-Trimethylbicyclo[2.2.1]Heptan-2-Ol,Borneol | CACNA1S |
| Curcuma phaeocaulis Valeton | MOL124 | 1,7,7-Trimethylbicyclo[2.2.1]Heptan-2-Ol,Borneol | CACNB1 |
| Curcuma phaeocaulis Valeton | MOL125 | 1,7,7-Trimethylbicyclo[2.2.1]Heptan-2-Ol,Borneol | CACNB2 |
| Curcuma phaeocaulis Valeton | MOL126 | 1,7,7-Trimethylbicyclo[2.2.1]Heptan-2-Ol,Borneol | CACNB3 |
| Curcuma phaeocaulis Valeton | MOL127 | 1,7,7-Trimethylbicyclo[2.2.1]Heptan-2-Ol,Borneol | CACNB4 |
| Curcuma phaeocaulis Valeton | MOL128 | 1,7,7-Trimethylbicyclo[2.2.1]Heptan-2-Ol,Borneol | ESR1 |
| Curcuma phaeocaulis Valeton | MOL129 | 1,7,7-Trimethylbicyclo[2.2.1]Heptan-2-Ol,Borneol | ESR2 |
| Curcuma phaeocaulis Valeton | MOL130 | 1,7,7-Trimethylbicyclo[2.2.1]Heptan-2-Ol,Borneol | GABRA1 |
| Curcuma phaeocaulis Valeton | MOL131 | 1,7,7-Trimethylbicyclo[2.2.1]Heptan-2-Ol,Borneol | GABRA2 |
| Curcuma phaeocaulis Valeton | MOL132 | 1,7,7-Trimethylbicyclo[2.2.1]Heptan-2-Ol,Borneol | GABRA3 |
| Curcuma phaeocaulis Valeton | MOL133 | 1,7,7-Trimethylbicyclo[2.2.1]Heptan-2-Ol,Borneol | GABRA4 |
| Curcuma phaeocaulis Valeton | MOL134 | 1,7,7-Trimethylbicyclo[2.2.1]Heptan-2-Ol,Borneol | GABRA5 |
| Curcuma phaeocaulis Valeton | MOL135 | 1,7,7-Trimethylbicyclo[2.2.1]Heptan-2-Ol,Borneol | GABRA6 |
| Curcuma phaeocaulis Valeton | MOL136 | 1,7,7-Trimethylbicyclo[2.2.1]Heptan-2-Ol,Borneol | GABRB1 |
| Curcuma phaeocaulis Valeton | MOL137 | 1,7,7-Trimethylbicyclo[2.2.1]Heptan-2-Ol,Borneol | GABRB2 |
| Curcuma phaeocaulis Valeton | MOL138 | 1,7,7-Trimethylbicyclo[2.2.1]Heptan-2-Ol,Borneol | GABRB3 |
| Curcuma phaeocaulis Valeton | MOL139 | 1,7,7-Trimethylbicyclo[2.2.1]Heptan-2-Ol,Borneol | GABRD |
| Curcuma phaeocaulis Valeton | MOL140 | 1,7,7-Trimethylbicyclo[2.2.1]Heptan-2-Ol,Borneol | GABRE |
| Curcuma phaeocaulis Valeton | MOL141 | 1,7,7-Trimethylbicyclo[2.2.1]Heptan-2-Ol,Borneol | GABRG1 |
| Curcuma phaeocaulis Valeton | MOL142 | 1,7,7-Trimethylbicyclo[2.2.1]Heptan-2-Ol,Borneol | GABRG2 |
| Curcuma phaeocaulis Valeton | MOL143 | 1,7,7-Trimethylbicyclo[2.2.1]Heptan-2-Ol,Borneol | GABRG3 |
| Curcuma phaeocaulis Valeton | MOL144 | 1,7,7-Trimethylbicyclo[2.2.1]Heptan-2-Ol,Borneol | GABRP |
| Curcuma phaeocaulis Valeton | MOL145 | 1,7,7-Trimethylbicyclo[2.2.1]Heptan-2-Ol,Borneol | GABRQ |
| Curcuma phaeocaulis Valeton | MOL146 | 1,7,7-Trimethylbicyclo[2.2.1]Heptan-2-Ol,Borneol | HSD17B1 |
| Curcuma phaeocaulis Valeton | MOL147 | 1,7,7-Trimethylbicyclo[2.2.1]Heptan-2-Ol,Borneol | HSD17B11 |
| Curcuma phaeocaulis Valeton | MOL148 | 1,7,7-Trimethylbicyclo[2.2.1]Heptan-2-Ol,Borneol | IGHG2 |
| Curcuma phaeocaulis Valeton | MOL149 | 1,7,7-Trimethylbicyclo[2.2.1]Heptan-2-Ol,Borneol | LSS |
| Curcuma phaeocaulis Valeton | MOL150 | 1,7,7-Trimethylbicyclo[2.2.1]Heptan-2-Ol,Borneol | NR1I3 |
| Curcuma phaeocaulis Valeton | MOL151 | 1,7,7-Trimethylbicyclo[2.2.1]Heptan-2-Ol,Borneol | NR3C2 |
| Curcuma phaeocaulis Valeton | MOL152 | 1,7,7-Trimethylbicyclo[2.2.1]Heptan-2-Ol,Borneol | OPRK1 |
| Curcuma phaeocaulis Valeton | MOL153 | 1,7,7-Trimethylbicyclo[2.2.1]Heptan-2-Ol,Borneol | SULT2A1 |
| Curcuma phaeocaulis Valeton | MOL154 | 1,7,7-Trimethylbicyclo[2.2.1]Heptan-2-Ol,Borneol | TRPA1 |
| Curcuma phaeocaulis Valeton | MOL155 | 1,7,7-Trimethylbicyclo[2.2.1]Heptan-2-Ol,Borneol | TRPM8 |
| Curcuma phaeocaulis Valeton | MOL156 | 1,7,7-Trimethylbicyclo[2.2.1]Heptan-2-Ol,Borneol | TRPV3 |
| Curcuma phaeocaulis Valeton | MOL157 | 1,7,7-Trimethylbicyclo[2.2.1]Heptan-2-Ol,Borneol | VDR |
| Curcuma phaeocaulis Valeton | MOL158 | (1R,2R,4R)-1,7,7-Trimethylbicyclo[2.2.1]Heptan-2-Ol,Isoborneol,L-Isoborneol | AKR1D1 |
| Curcuma phaeocaulis Valeton | MOL159 | (1R,2R,4R)-1,7,7-Trimethylbicyclo[2.2.1]Heptan-2-Ol,Isoborneol,L-Isoborneol | AR |
| Curcuma phaeocaulis Valeton | MOL160 | (1R,2R,4R)-1,7,7-Trimethylbicyclo[2.2.1]Heptan-2-Ol,Isoborneol,L-Isoborneol | CACNA1C |
| Curcuma phaeocaulis Valeton | MOL161 | (1R,2R,4R)-1,7,7-Trimethylbicyclo[2.2.1]Heptan-2-Ol,Isoborneol,L-Isoborneol | CACNA1D |
| Curcuma phaeocaulis Valeton | MOL162 | (1R,2R,4R)-1,7,7-Trimethylbicyclo[2.2.1]Heptan-2-Ol,Isoborneol,L-Isoborneol | CACNA1F |
| Curcuma phaeocaulis Valeton | MOL163 | (1R,2R,4R)-1,7,7-Trimethylbicyclo[2.2.1]Heptan-2-Ol,Isoborneol,L-Isoborneol | CACNA1S |
| Curcuma phaeocaulis Valeton | MOL164 | (1R,2R,4R)-1,7,7-Trimethylbicyclo[2.2.1]Heptan-2-Ol,Isoborneol,L-Isoborneol | CACNB1 |
| Curcuma phaeocaulis Valeton | MOL165 | (1R,2R,4R)-1,7,7-Trimethylbicyclo[2.2.1]Heptan-2-Ol,Isoborneol,L-Isoborneol | CACNB2 |
| Curcuma phaeocaulis Valeton | MOL166 | (1R,2R,4R)-1,7,7-Trimethylbicyclo[2.2.1]Heptan-2-Ol,Isoborneol,L-Isoborneol | CACNB3 |
| Curcuma phaeocaulis Valeton | MOL167 | (1R,2R,4R)-1,7,7-Trimethylbicyclo[2.2.1]Heptan-2-Ol,Isoborneol,L-Isoborneol | CACNB4 |
| Curcuma phaeocaulis Valeton | MOL168 | (1R,2R,4R)-1,7,7-Trimethylbicyclo[2.2.1]Heptan-2-Ol,Isoborneol,L-Isoborneol | ESR1 |
| Curcuma phaeocaulis Valeton | MOL169 | (1R,2R,4R)-1,7,7-Trimethylbicyclo[2.2.1]Heptan-2-Ol,Isoborneol,L-Isoborneol | ESR2 |
| Curcuma phaeocaulis Valeton | MOL170 | (1R,2R,4R)-1,7,7-Trimethylbicyclo[2.2.1]Heptan-2-Ol,Isoborneol,L-Isoborneol | GABRA1 |
| Curcuma phaeocaulis Valeton | MOL171 | (1R,2R,4R)-1,7,7-Trimethylbicyclo[2.2.1]Heptan-2-Ol,Isoborneol,L-Isoborneol | GABRA2 |
| Curcuma phaeocaulis Valeton | MOL172 | (1R,2R,4R)-1,7,7-Trimethylbicyclo[2.2.1]Heptan-2-Ol,Isoborneol,L-Isoborneol | GABRA3 |
| Curcuma phaeocaulis Valeton | MOL173 | (1R,2R,4R)-1,7,7-Trimethylbicyclo[2.2.1]Heptan-2-Ol,Isoborneol,L-Isoborneol | GABRA4 |
| Curcuma phaeocaulis Valeton | MOL174 | (1R,2R,4R)-1,7,7-Trimethylbicyclo[2.2.1]Heptan-2-Ol,Isoborneol,L-Isoborneol | GABRA5 |
| Curcuma phaeocaulis Valeton | MOL175 | (1R,2R,4R)-1,7,7-Trimethylbicyclo[2.2.1]Heptan-2-Ol,Isoborneol,L-Isoborneol | GABRA6 |
| Curcuma phaeocaulis Valeton | MOL176 | (1R,2R,4R)-1,7,7-Trimethylbicyclo[2.2.1]Heptan-2-Ol,Isoborneol,L-Isoborneol | GABRB1 |
| Curcuma phaeocaulis Valeton | MOL177 | (1R,2R,4R)-1,7,7-Trimethylbicyclo[2.2.1]Heptan-2-Ol,Isoborneol,L-Isoborneol | GABRB2 |
| Curcuma phaeocaulis Valeton | MOL178 | (1R,2R,4R)-1,7,7-Trimethylbicyclo[2.2.1]Heptan-2-Ol,Isoborneol,L-Isoborneol | GABRB3 |
| Curcuma phaeocaulis Valeton | MOL179 | (1R,2R,4R)-1,7,7-Trimethylbicyclo[2.2.1]Heptan-2-Ol,Isoborneol,L-Isoborneol | GABRD |
| Curcuma phaeocaulis Valeton | MOL180 | (1R,2R,4R)-1,7,7-Trimethylbicyclo[2.2.1]Heptan-2-Ol,Isoborneol,L-Isoborneol | GABRE |
| Curcuma phaeocaulis Valeton | MOL181 | (1R,2R,4R)-1,7,7-Trimethylbicyclo[2.2.1]Heptan-2-Ol,Isoborneol,L-Isoborneol | GABRG1 |
| Curcuma phaeocaulis Valeton | MOL182 | (1R,2R,4R)-1,7,7-Trimethylbicyclo[2.2.1]Heptan-2-Ol,Isoborneol,L-Isoborneol | GABRG2 |
| Curcuma phaeocaulis Valeton | MOL183 | (1R,2R,4R)-1,7,7-Trimethylbicyclo[2.2.1]Heptan-2-Ol,Isoborneol,L-Isoborneol | GABRG3 |
| Curcuma phaeocaulis Valeton | MOL184 | (1R,2R,4R)-1,7,7-Trimethylbicyclo[2.2.1]Heptan-2-Ol,Isoborneol,L-Isoborneol | GABRP |
| Curcuma phaeocaulis Valeton | MOL185 | (1R,2R,4R)-1,7,7-Trimethylbicyclo[2.2.1]Heptan-2-Ol,Isoborneol,L-Isoborneol | GABRQ |
| Curcuma phaeocaulis Valeton | MOL186 | (1R,2R,4R)-1,7,7-Trimethylbicyclo[2.2.1]Heptan-2-Ol,Isoborneol,L-Isoborneol | HSD17B1 |
| Curcuma phaeocaulis Valeton | MOL187 | (1R,2R,4R)-1,7,7-Trimethylbicyclo[2.2.1]Heptan-2-Ol,Isoborneol,L-Isoborneol | HSD17B11 |
| Curcuma phaeocaulis Valeton | MOL188 | (1R,2R,4R)-1,7,7-Trimethylbicyclo[2.2.1]Heptan-2-Ol,Isoborneol,L-Isoborneol | IGHG2 |
| Curcuma phaeocaulis Valeton | MOL189 | (1R,2R,4R)-1,7,7-Trimethylbicyclo[2.2.1]Heptan-2-Ol,Isoborneol,L-Isoborneol | LSS |
| Curcuma phaeocaulis Valeton | MOL190 | (1R,2R,4R)-1,7,7-Trimethylbicyclo[2.2.1]Heptan-2-Ol,Isoborneol,L-Isoborneol | NR1I3 |
| Curcuma phaeocaulis Valeton | MOL191 | (1R,2R,4R)-1,7,7-Trimethylbicyclo[2.2.1]Heptan-2-Ol,Isoborneol,L-Isoborneol | NR3C2 |
| Curcuma phaeocaulis Valeton | MOL192 | (1R,2R,4R)-1,7,7-Trimethylbicyclo[2.2.1]Heptan-2-Ol,Isoborneol,L-Isoborneol | OPRK1 |
| Curcuma phaeocaulis Valeton | MOL193 | (1R,2R,4R)-1,7,7-Trimethylbicyclo[2.2.1]Heptan-2-Ol,Isoborneol,L-Isoborneol | SULT2A1 |
| Curcuma phaeocaulis Valeton | MOL194 | (1R,2R,4R)-1,7,7-Trimethylbicyclo[2.2.1]Heptan-2-Ol,Isoborneol,L-Isoborneol | TRPA1 |
| Curcuma phaeocaulis Valeton | MOL195 | (1R,2R,4R)-1,7,7-Trimethylbicyclo[2.2.1]Heptan-2-Ol,Isoborneol,L-Isoborneol | TRPM8 |
| Curcuma phaeocaulis Valeton | MOL196 | (1R,2R,4R)-1,7,7-Trimethylbicyclo[2.2.1]Heptan-2-Ol,Isoborneol,L-Isoborneol | TRPV3 |
| Curcuma phaeocaulis Valeton | MOL197 | (1R,2R,4R)-1,7,7-Trimethylbicyclo[2.2.1]Heptan-2-Ol,Isoborneol,L-Isoborneol | VDR |
| Curcuma phaeocaulis Valeton | MOL198 | (3S,3As,8Ar,Z)-3-Hydroxy-3,8-Dimethyl-5-(Propan-2-Ylidene)-1,2,3,3A,4,5-Hexahydroazulen-6(8Ah)-One | AKR1C1 |
| Curcuma phaeocaulis Valeton | MOL199 | (3S,3As,8Ar,Z)-3-Hydroxy-3,8-Dimethyl-5-(Propan-2-Ylidene)-1,2,3,3A,4,5-Hexahydroazulen-6(8Ah)-One | AKR1C2 |
| Curcuma phaeocaulis Valeton | MOL200 | (3S,3As,8Ar,Z)-3-Hydroxy-3,8-Dimethyl-5-(Propan-2-Ylidene)-1,2,3,3A,4,5-Hexahydroazulen-6(8Ah)-One | AR |
| Curcuma phaeocaulis Valeton | MOL201 | (3S,3As,8Ar,Z)-3-Hydroxy-3,8-Dimethyl-5-(Propan-2-Ylidene)-1,2,3,3A,4,5-Hexahydroazulen-6(8Ah)-One | ESR1 |
| Curcuma phaeocaulis Valeton | MOL202 | (3S,3As,8Ar,Z)-3-Hydroxy-3,8-Dimethyl-5-(Propan-2-Ylidene)-1,2,3,3A,4,5-Hexahydroazulen-6(8Ah)-One | ESR2 |
| Curcuma phaeocaulis Valeton | MOL203 | (3S,3As,8Ar,Z)-3-Hydroxy-3,8-Dimethyl-5-(Propan-2-Ylidene)-1,2,3,3A,4,5-Hexahydroazulen-6(8Ah)-One | GABRA1 |
| Curcuma phaeocaulis Valeton | MOL204 | (3S,3As,8Ar,Z)-3-Hydroxy-3,8-Dimethyl-5-(Propan-2-Ylidene)-1,2,3,3A,4,5-Hexahydroazulen-6(8Ah)-One | GABRA2 |
| Curcuma phaeocaulis Valeton | MOL205 | (3S,3As,8Ar,Z)-3-Hydroxy-3,8-Dimethyl-5-(Propan-2-Ylidene)-1,2,3,3A,4,5-Hexahydroazulen-6(8Ah)-One | GABRA3 |
| Curcuma phaeocaulis Valeton | MOL206 | (3S,3As,8Ar,Z)-3-Hydroxy-3,8-Dimethyl-5-(Propan-2-Ylidene)-1,2,3,3A,4,5-Hexahydroazulen-6(8Ah)-One | GABRA4 |
| Curcuma phaeocaulis Valeton | MOL207 | (3S,3As,8Ar,Z)-3-Hydroxy-3,8-Dimethyl-5-(Propan-2-Ylidene)-1,2,3,3A,4,5-Hexahydroazulen-6(8Ah)-One | GABRA5 |
| Curcuma phaeocaulis Valeton | MOL208 | (3S,3As,8Ar,Z)-3-Hydroxy-3,8-Dimethyl-5-(Propan-2-Ylidene)-1,2,3,3A,4,5-Hexahydroazulen-6(8Ah)-One | GABRA6 |
| Curcuma phaeocaulis Valeton | MOL209 | (3S,3As,8Ar,Z)-3-Hydroxy-3,8-Dimethyl-5-(Propan-2-Ylidene)-1,2,3,3A,4,5-Hexahydroazulen-6(8Ah)-One | GABRB1 |
| Curcuma phaeocaulis Valeton | MOL210 | (3S,3As,8Ar,Z)-3-Hydroxy-3,8-Dimethyl-5-(Propan-2-Ylidene)-1,2,3,3A,4,5-Hexahydroazulen-6(8Ah)-One | GABRB2 |
| Curcuma phaeocaulis Valeton | MOL211 | (3S,3As,8Ar,Z)-3-Hydroxy-3,8-Dimethyl-5-(Propan-2-Ylidene)-1,2,3,3A,4,5-Hexahydroazulen-6(8Ah)-One | GABRB3 |
| Curcuma phaeocaulis Valeton | MOL212 | (3S,3As,8Ar,Z)-3-Hydroxy-3,8-Dimethyl-5-(Propan-2-Ylidene)-1,2,3,3A,4,5-Hexahydroazulen-6(8Ah)-One | GABRD |
| Curcuma phaeocaulis Valeton | MOL213 | (3S,3As,8Ar,Z)-3-Hydroxy-3,8-Dimethyl-5-(Propan-2-Ylidene)-1,2,3,3A,4,5-Hexahydroazulen-6(8Ah)-One | GABRE |
| Curcuma phaeocaulis Valeton | MOL214 | (3S,3As,8Ar,Z)-3-Hydroxy-3,8-Dimethyl-5-(Propan-2-Ylidene)-1,2,3,3A,4,5-Hexahydroazulen-6(8Ah)-One | GABRG1 |
| Curcuma phaeocaulis Valeton | MOL215 | (3S,3As,8Ar,Z)-3-Hydroxy-3,8-Dimethyl-5-(Propan-2-Ylidene)-1,2,3,3A,4,5-Hexahydroazulen-6(8Ah)-One | GABRG2 |
| Curcuma phaeocaulis Valeton | MOL216 | (3S,3As,8Ar,Z)-3-Hydroxy-3,8-Dimethyl-5-(Propan-2-Ylidene)-1,2,3,3A,4,5-Hexahydroazulen-6(8Ah)-One | GABRG3 |
| Curcuma phaeocaulis Valeton | MOL217 | (3S,3As,8Ar,Z)-3-Hydroxy-3,8-Dimethyl-5-(Propan-2-Ylidene)-1,2,3,3A,4,5-Hexahydroazulen-6(8Ah)-One | GABRP |
| Curcuma phaeocaulis Valeton | MOL218 | (3S,3As,8Ar,Z)-3-Hydroxy-3,8-Dimethyl-5-(Propan-2-Ylidene)-1,2,3,3A,4,5-Hexahydroazulen-6(8Ah)-One | GABRQ |
| Curcuma phaeocaulis Valeton | MOL219 | (3S,3As,8Ar,Z)-3-Hydroxy-3,8-Dimethyl-5-(Propan-2-Ylidene)-1,2,3,3A,4,5-Hexahydroazulen-6(8Ah)-One | GRIN1 |
| Curcuma phaeocaulis Valeton | MOL220 | (3S,3As,8Ar,Z)-3-Hydroxy-3,8-Dimethyl-5-(Propan-2-Ylidene)-1,2,3,3A,4,5-Hexahydroazulen-6(8Ah)-One | GRIN2A |
| Curcuma phaeocaulis Valeton | MOL221 | (3S,3As,8Ar,Z)-3-Hydroxy-3,8-Dimethyl-5-(Propan-2-Ylidene)-1,2,3,3A,4,5-Hexahydroazulen-6(8Ah)-One | GRIN2B |
| Curcuma phaeocaulis Valeton | MOL222 | (3S,3As,8Ar,Z)-3-Hydroxy-3,8-Dimethyl-5-(Propan-2-Ylidene)-1,2,3,3A,4,5-Hexahydroazulen-6(8Ah)-One | GRIN2C |
| Curcuma phaeocaulis Valeton | MOL223 | (3S,3As,8Ar,Z)-3-Hydroxy-3,8-Dimethyl-5-(Propan-2-Ylidene)-1,2,3,3A,4,5-Hexahydroazulen-6(8Ah)-One | GRIN2D |
| Curcuma phaeocaulis Valeton | MOL224 | (3S,3As,8Ar,Z)-3-Hydroxy-3,8-Dimethyl-5-(Propan-2-Ylidene)-1,2,3,3A,4,5-Hexahydroazulen-6(8Ah)-One | GRIN3A |
| Curcuma phaeocaulis Valeton | MOL225 | (3S,3As,8Ar,Z)-3-Hydroxy-3,8-Dimethyl-5-(Propan-2-Ylidene)-1,2,3,3A,4,5-Hexahydroazulen-6(8Ah)-One | GRIN3B |
| Curcuma phaeocaulis Valeton | MOL226 | (3S,3As,8Ar,Z)-3-Hydroxy-3,8-Dimethyl-5-(Propan-2-Ylidene)-1,2,3,3A,4,5-Hexahydroazulen-6(8Ah)-One | HSD17B1 |
| Curcuma phaeocaulis Valeton | MOL227 | (3S,3As,8Ar,Z)-3-Hydroxy-3,8-Dimethyl-5-(Propan-2-Ylidene)-1,2,3,3A,4,5-Hexahydroazulen-6(8Ah)-One | LSS |
| Curcuma phaeocaulis Valeton | MOL228 | (3S,3As,8Ar,Z)-3-Hydroxy-3,8-Dimethyl-5-(Propan-2-Ylidene)-1,2,3,3A,4,5-Hexahydroazulen-6(8Ah)-One | NCOA2 |
| Curcuma phaeocaulis Valeton | MOL229 | (3S,3As,8Ar,Z)-3-Hydroxy-3,8-Dimethyl-5-(Propan-2-Ylidene)-1,2,3,3A,4,5-Hexahydroazulen-6(8Ah)-One | NPPB |
| Curcuma phaeocaulis Valeton | MOL230 | (3S,3As,8Ar,Z)-3-Hydroxy-3,8-Dimethyl-5-(Propan-2-Ylidene)-1,2,3,3A,4,5-Hexahydroazulen-6(8Ah)-One | NR1I2 |
| Curcuma phaeocaulis Valeton | MOL231 | (3S,3As,8Ar,Z)-3-Hydroxy-3,8-Dimethyl-5-(Propan-2-Ylidene)-1,2,3,3A,4,5-Hexahydroazulen-6(8Ah)-One | NR1I3 |
| Curcuma phaeocaulis Valeton | MOL232 | (3S,3As,8Ar,Z)-3-Hydroxy-3,8-Dimethyl-5-(Propan-2-Ylidene)-1,2,3,3A,4,5-Hexahydroazulen-6(8Ah)-One | NR3C2 |
| Curcuma phaeocaulis Valeton | MOL233 | (3S,3As,8Ar,Z)-3-Hydroxy-3,8-Dimethyl-5-(Propan-2-Ylidene)-1,2,3,3A,4,5-Hexahydroazulen-6(8Ah)-One | PGR |
| Curcuma phaeocaulis Valeton | MOL234 | (3S,3As,8Ar,Z)-3-Hydroxy-3,8-Dimethyl-5-(Propan-2-Ylidene)-1,2,3,3A,4,5-Hexahydroazulen-6(8Ah)-One | PPARA |
| Curcuma phaeocaulis Valeton | MOL235 | (3S,3As,8Ar,Z)-3-Hydroxy-3,8-Dimethyl-5-(Propan-2-Ylidene)-1,2,3,3A,4,5-Hexahydroazulen-6(8Ah)-One | SIGMAR1 |
| Curcuma phaeocaulis Valeton | MOL236 | (3S,3As,8Ar,Z)-3-Hydroxy-3,8-Dimethyl-5-(Propan-2-Ylidene)-1,2,3,3A,4,5-Hexahydroazulen-6(8Ah)-One | SULT2A1 |
| Curcuma phaeocaulis Valeton | MOL237 | (3S,3As,8Ar,Z)-3-Hydroxy-3,8-Dimethyl-5-(Propan-2-Ylidene)-1,2,3,3A,4,5-Hexahydroazulen-6(8Ah)-One | SULT2B1 |
| Curcuma phaeocaulis Valeton | MOL238 | (3S,3As,8Ar,Z)-3-Hydroxy-3,8-Dimethyl-5-(Propan-2-Ylidene)-1,2,3,3A,4,5-Hexahydroazulen-6(8Ah)-One | VDR |
| Curcuma phaeocaulis Valeton | MOL239 | (S)-5-Hydroxy-7-Methoxy-2-Phenylchroman-4-One,7-Hydroxy-5-Methoxyflavanone | AKR1C1 |
| Curcuma phaeocaulis Valeton | MOL240 | (S)-5-Hydroxy-7-Methoxy-2-Phenylchroman-4-One,7-Hydroxy-5-Methoxyflavanone | CYP19A1 |
| Curcuma phaeocaulis Valeton | MOL241 | (S)-5-Hydroxy-7-Methoxy-2-Phenylchroman-4-One,7-Hydroxy-5-Methoxyflavanone | CYP1B1 |
| Curcuma phaeocaulis Valeton | MOL242 | (S)-5-Hydroxy-7-Methoxy-2-Phenylchroman-4-One,7-Hydroxy-5-Methoxyflavanone | ESR1 |
| Curcuma phaeocaulis Valeton | MOL243 | (S)-5-Hydroxy-7-Methoxy-2-Phenylchroman-4-One,7-Hydroxy-5-Methoxyflavanone | ESR2 |
| Curcuma phaeocaulis Valeton | MOL244 | (S)-5-Hydroxy-7-Methoxy-2-Phenylchroman-4-One,7-Hydroxy-5-Methoxyflavanone | KANSL3 |
| Curcuma phaeocaulis Valeton | MOL245 | (S)-5-Hydroxy-7-Methoxy-2-Phenylchroman-4-One,7-Hydroxy-5-Methoxyflavanone | MTTP |
| Curcuma phaeocaulis Valeton | MOL246 | (S)-5-Hydroxy-7-Methoxy-2-Phenylchroman-4-One,7-Hydroxy-5-Methoxyflavanone | SHBG |
| Curcuma phaeocaulis Valeton | MOL247 | (S)-5-Hydroxy-7-Methoxy-2-Phenylchroman-4-One,7-Hydroxy-5-Methoxyflavanone | SOAT1 |
| Curcuma phaeocaulis Valeton | MOL248 | (S)-5-Hydroxy-7-Methoxy-2-Phenylchroman-4-One,7-Hydroxy-5-Methoxyflavanone | SOAT2 |
| Curcuma phaeocaulis Valeton | MOL249 | (1S,4R,4Ar,8Ar)-1-Isopropyl-4,7-Dimethyl-1,2,3,4,4A,5,6,8A-Octahydronaphthalen-4A-Ol | AKR1C1 |
| Curcuma phaeocaulis Valeton | MOL250 | (1S,4R,4Ar,8Ar)-1-Isopropyl-4,7-Dimethyl-1,2,3,4,4A,5,6,8A-Octahydronaphthalen-4A-Ol | AKR1C2 |
| Curcuma phaeocaulis Valeton | MOL251 | (1S,4R,4Ar,8Ar)-1-Isopropyl-4,7-Dimethyl-1,2,3,4,4A,5,6,8A-Octahydronaphthalen-4A-Ol | AR |
| Curcuma phaeocaulis Valeton | MOL252 | (1S,4R,4Ar,8Ar)-1-Isopropyl-4,7-Dimethyl-1,2,3,4,4A,5,6,8A-Octahydronaphthalen-4A-Ol | CLEC4E |
| Curcuma phaeocaulis Valeton | MOL253 | (1S,4R,4Ar,8Ar)-1-Isopropyl-4,7-Dimethyl-1,2,3,4,4A,5,6,8A-Octahydronaphthalen-4A-Ol | ESR1 |
| Curcuma phaeocaulis Valeton | MOL254 | (1S,4R,4Ar,8Ar)-1-Isopropyl-4,7-Dimethyl-1,2,3,4,4A,5,6,8A-Octahydronaphthalen-4A-Ol | ESR2 |
| Curcuma phaeocaulis Valeton | MOL255 | (1S,4R,4Ar,8Ar)-1-Isopropyl-4,7-Dimethyl-1,2,3,4,4A,5,6,8A-Octahydronaphthalen-4A-Ol | GABRA1 |
| Curcuma phaeocaulis Valeton | MOL256 | (1S,4R,4Ar,8Ar)-1-Isopropyl-4,7-Dimethyl-1,2,3,4,4A,5,6,8A-Octahydronaphthalen-4A-Ol | GABRA2 |
| Curcuma phaeocaulis Valeton | MOL257 | (1S,4R,4Ar,8Ar)-1-Isopropyl-4,7-Dimethyl-1,2,3,4,4A,5,6,8A-Octahydronaphthalen-4A-Ol | GABRA3 |
| Curcuma phaeocaulis Valeton | MOL258 | (1S,4R,4Ar,8Ar)-1-Isopropyl-4,7-Dimethyl-1,2,3,4,4A,5,6,8A-Octahydronaphthalen-4A-Ol | GABRA4 |
| Curcuma phaeocaulis Valeton | MOL259 | (1S,4R,4Ar,8Ar)-1-Isopropyl-4,7-Dimethyl-1,2,3,4,4A,5,6,8A-Octahydronaphthalen-4A-Ol | GABRA5 |
| Curcuma phaeocaulis Valeton | MOL260 | (1S,4R,4Ar,8Ar)-1-Isopropyl-4,7-Dimethyl-1,2,3,4,4A,5,6,8A-Octahydronaphthalen-4A-Ol | GABRA6 |
| Curcuma phaeocaulis Valeton | MOL261 | (1S,4R,4Ar,8Ar)-1-Isopropyl-4,7-Dimethyl-1,2,3,4,4A,5,6,8A-Octahydronaphthalen-4A-Ol | GABRB1 |
| Curcuma phaeocaulis Valeton | MOL262 | (1S,4R,4Ar,8Ar)-1-Isopropyl-4,7-Dimethyl-1,2,3,4,4A,5,6,8A-Octahydronaphthalen-4A-Ol | GABRB2 |
| Curcuma phaeocaulis Valeton | MOL263 | (1S,4R,4Ar,8Ar)-1-Isopropyl-4,7-Dimethyl-1,2,3,4,4A,5,6,8A-Octahydronaphthalen-4A-Ol | GABRB3 |
| Curcuma phaeocaulis Valeton | MOL264 | (1S,4R,4Ar,8Ar)-1-Isopropyl-4,7-Dimethyl-1,2,3,4,4A,5,6,8A-Octahydronaphthalen-4A-Ol | GABRD |
| Curcuma phaeocaulis Valeton | MOL265 | (1S,4R,4Ar,8Ar)-1-Isopropyl-4,7-Dimethyl-1,2,3,4,4A,5,6,8A-Octahydronaphthalen-4A-Ol | GABRE |
| Curcuma phaeocaulis Valeton | MOL266 | (1S,4R,4Ar,8Ar)-1-Isopropyl-4,7-Dimethyl-1,2,3,4,4A,5,6,8A-Octahydronaphthalen-4A-Ol | GABRG1 |
| Curcuma phaeocaulis Valeton | MOL267 | (1S,4R,4Ar,8Ar)-1-Isopropyl-4,7-Dimethyl-1,2,3,4,4A,5,6,8A-Octahydronaphthalen-4A-Ol | GABRG2 |
| Curcuma phaeocaulis Valeton | MOL268 | (1S,4R,4Ar,8Ar)-1-Isopropyl-4,7-Dimethyl-1,2,3,4,4A,5,6,8A-Octahydronaphthalen-4A-Ol | GABRG3 |
| Curcuma phaeocaulis Valeton | MOL269 | (1S,4R,4Ar,8Ar)-1-Isopropyl-4,7-Dimethyl-1,2,3,4,4A,5,6,8A-Octahydronaphthalen-4A-Ol | GABRP |
| Curcuma phaeocaulis Valeton | MOL270 | (1S,4R,4Ar,8Ar)-1-Isopropyl-4,7-Dimethyl-1,2,3,4,4A,5,6,8A-Octahydronaphthalen-4A-Ol | GABRQ |
| Curcuma phaeocaulis Valeton | MOL271 | (1S,4R,4Ar,8Ar)-1-Isopropyl-4,7-Dimethyl-1,2,3,4,4A,5,6,8A-Octahydronaphthalen-4A-Ol | GRIN1 |
| Curcuma phaeocaulis Valeton | MOL272 | (1S,4R,4Ar,8Ar)-1-Isopropyl-4,7-Dimethyl-1,2,3,4,4A,5,6,8A-Octahydronaphthalen-4A-Ol | GRIN2A |
| Curcuma phaeocaulis Valeton | MOL273 | (1S,4R,4Ar,8Ar)-1-Isopropyl-4,7-Dimethyl-1,2,3,4,4A,5,6,8A-Octahydronaphthalen-4A-Ol | GRIN2B |
| Curcuma phaeocaulis Valeton | MOL274 | (1S,4R,4Ar,8Ar)-1-Isopropyl-4,7-Dimethyl-1,2,3,4,4A,5,6,8A-Octahydronaphthalen-4A-Ol | GRIN2C |
| Curcuma phaeocaulis Valeton | MOL275 | (1S,4R,4Ar,8Ar)-1-Isopropyl-4,7-Dimethyl-1,2,3,4,4A,5,6,8A-Octahydronaphthalen-4A-Ol | GRIN2D |
| Curcuma phaeocaulis Valeton | MOL276 | (1S,4R,4Ar,8Ar)-1-Isopropyl-4,7-Dimethyl-1,2,3,4,4A,5,6,8A-Octahydronaphthalen-4A-Ol | GRIN3A |
| Curcuma phaeocaulis Valeton | MOL277 | (1S,4R,4Ar,8Ar)-1-Isopropyl-4,7-Dimethyl-1,2,3,4,4A,5,6,8A-Octahydronaphthalen-4A-Ol | GRIN3B |
| Curcuma phaeocaulis Valeton | MOL278 | (1S,4R,4Ar,8Ar)-1-Isopropyl-4,7-Dimethyl-1,2,3,4,4A,5,6,8A-Octahydronaphthalen-4A-Ol | HOXA10 |
| Curcuma phaeocaulis Valeton | MOL279 | (1S,4R,4Ar,8Ar)-1-Isopropyl-4,7-Dimethyl-1,2,3,4,4A,5,6,8A-Octahydronaphthalen-4A-Ol | HSD17B1 |
| Curcuma phaeocaulis Valeton | MOL280 | (1S,4R,4Ar,8Ar)-1-Isopropyl-4,7-Dimethyl-1,2,3,4,4A,5,6,8A-Octahydronaphthalen-4A-Ol | LSS |
| Curcuma phaeocaulis Valeton | MOL281 | (1S,4R,4Ar,8Ar)-1-Isopropyl-4,7-Dimethyl-1,2,3,4,4A,5,6,8A-Octahydronaphthalen-4A-Ol | NCOA2 |
| Curcuma phaeocaulis Valeton | MOL282 | (1S,4R,4Ar,8Ar)-1-Isopropyl-4,7-Dimethyl-1,2,3,4,4A,5,6,8A-Octahydronaphthalen-4A-Ol | NPPB |
| Curcuma phaeocaulis Valeton | MOL283 | (1S,4R,4Ar,8Ar)-1-Isopropyl-4,7-Dimethyl-1,2,3,4,4A,5,6,8A-Octahydronaphthalen-4A-Ol | NR1I2 |
| Curcuma phaeocaulis Valeton | MOL284 | (1S,4R,4Ar,8Ar)-1-Isopropyl-4,7-Dimethyl-1,2,3,4,4A,5,6,8A-Octahydronaphthalen-4A-Ol | NR1I3 |
| Curcuma phaeocaulis Valeton | MOL285 | (1S,4R,4Ar,8Ar)-1-Isopropyl-4,7-Dimethyl-1,2,3,4,4A,5,6,8A-Octahydronaphthalen-4A-Ol | NR3C2 |
| Curcuma phaeocaulis Valeton | MOL286 | (1S,4R,4Ar,8Ar)-1-Isopropyl-4,7-Dimethyl-1,2,3,4,4A,5,6,8A-Octahydronaphthalen-4A-Ol | PGR |
| Curcuma phaeocaulis Valeton | MOL287 | (1S,4R,4Ar,8Ar)-1-Isopropyl-4,7-Dimethyl-1,2,3,4,4A,5,6,8A-Octahydronaphthalen-4A-Ol | PPARA |
| Curcuma phaeocaulis Valeton | MOL288 | (1S,4R,4Ar,8Ar)-1-Isopropyl-4,7-Dimethyl-1,2,3,4,4A,5,6,8A-Octahydronaphthalen-4A-Ol | RORA |
| Curcuma phaeocaulis Valeton | MOL289 | (1S,4R,4Ar,8Ar)-1-Isopropyl-4,7-Dimethyl-1,2,3,4,4A,5,6,8A-Octahydronaphthalen-4A-Ol | SIGMAR1 |
| Curcuma phaeocaulis Valeton | MOL290 | (1S,4R,4Ar,8Ar)-1-Isopropyl-4,7-Dimethyl-1,2,3,4,4A,5,6,8A-Octahydronaphthalen-4A-Ol | SULT2A1 |
| Curcuma phaeocaulis Valeton | MOL291 | (1S,4R,4Ar,8Ar)-1-Isopropyl-4,7-Dimethyl-1,2,3,4,4A,5,6,8A-Octahydronaphthalen-4A-Ol | SULT2B1 |
| Curcuma phaeocaulis Valeton | MOL292 | (1S,4R,4Ar,8Ar)-1-Isopropyl-4,7-Dimethyl-1,2,3,4,4A,5,6,8A-Octahydronaphthalen-4A-Ol | VDR |
| Curcuma phaeocaulis Valeton | MOL293 | (3S,3As,8Ar)-3-Hydroxy-3-Methyl-8-Methylene-5-(Propan-2-Ylidene)-Hexahydroazulen-6(1H,2H,7H)-One | AKR1C1 |
| Curcuma phaeocaulis Valeton | MOL294 | (3S,3As,8Ar)-3-Hydroxy-3-Methyl-8-Methylene-5-(Propan-2-Ylidene)-Hexahydroazulen-6(1H,2H,7H)-One | AKR1C2 |
| Curcuma phaeocaulis Valeton | MOL295 | (3S,3As,8Ar)-3-Hydroxy-3-Methyl-8-Methylene-5-(Propan-2-Ylidene)-Hexahydroazulen-6(1H,2H,7H)-One | AR |
| Curcuma phaeocaulis Valeton | MOL296 | (3S,3As,8Ar)-3-Hydroxy-3-Methyl-8-Methylene-5-(Propan-2-Ylidene)-Hexahydroazulen-6(1H,2H,7H)-One | ESR1 |
| Curcuma phaeocaulis Valeton | MOL297 | (3S,3As,8Ar)-3-Hydroxy-3-Methyl-8-Methylene-5-(Propan-2-Ylidene)-Hexahydroazulen-6(1H,2H,7H)-One | NCOA2 |
| Curcuma phaeocaulis Valeton | MOL298 | (3S,3As,8Ar)-3-Hydroxy-3-Methyl-8-Methylene-5-(Propan-2-Ylidene)-Hexahydroazulen-6(1H,2H,7H)-One | NPPB |
| Curcuma phaeocaulis Valeton | MOL299 | (3S,3As,8Ar)-3-Hydroxy-3-Methyl-8-Methylene-5-(Propan-2-Ylidene)-Hexahydroazulen-6(1H,2H,7H)-One | NR1I2 |
| Curcuma phaeocaulis Valeton | MOL300 | (3S,3As,8Ar)-3-Hydroxy-3-Methyl-8-Methylene-5-(Propan-2-Ylidene)-Hexahydroazulen-6(1H,2H,7H)-One | NR3C2 |
| Curcuma phaeocaulis Valeton | MOL301 | (3S,3As,8Ar)-3-Hydroxy-3-Methyl-8-Methylene-5-(Propan-2-Ylidene)-Hexahydroazulen-6(1H,2H,7H)-One | PGR |
| Curcuma phaeocaulis Valeton | MOL302 | (3S,3As,8Ar)-3-Hydroxy-3-Methyl-8-Methylene-5-(Propan-2-Ylidene)-Hexahydroazulen-6(1H,2H,7H)-One | SULT2B1 |
| Curcuma phaeocaulis Valeton | MOL303 | (3S,3As,8Ar)-3-Hydroxy-3-Methyl-8-Methylene-5-(Propan-2-Ylidene)-Hexahydroazulen-6(1H,2H,7H)-One | VDR |
| Curcuma phaeocaulis Valeton | MOL304 | (1S,4S,4Ar,8Ar)-4-Isopropyl-1,6-Dimethyl-1,2,3,4,4A,7,8,8A-Octahydronaphthalen-1-Ol | AKR1C1 |
| Curcuma phaeocaulis Valeton | MOL305 | (1S,4S,4Ar,8Ar)-4-Isopropyl-1,6-Dimethyl-1,2,3,4,4A,7,8,8A-Octahydronaphthalen-1-Ol | AKR1C2 |
| Curcuma phaeocaulis Valeton | MOL306 | (1S,4S,4Ar,8Ar)-4-Isopropyl-1,6-Dimethyl-1,2,3,4,4A,7,8,8A-Octahydronaphthalen-1-Ol | AR |
| Curcuma phaeocaulis Valeton | MOL307 | (1S,4S,4Ar,8Ar)-4-Isopropyl-1,6-Dimethyl-1,2,3,4,4A,7,8,8A-Octahydronaphthalen-1-Ol | CLEC4E |
| Curcuma phaeocaulis Valeton | MOL308 | (1S,4S,4Ar,8Ar)-4-Isopropyl-1,6-Dimethyl-1,2,3,4,4A,7,8,8A-Octahydronaphthalen-1-Ol | ESR1 |
| Curcuma phaeocaulis Valeton | MOL309 | (1S,4S,4Ar,8Ar)-4-Isopropyl-1,6-Dimethyl-1,2,3,4,4A,7,8,8A-Octahydronaphthalen-1-Ol | LSS |
| Curcuma phaeocaulis Valeton | MOL310 | (1S,4S,4Ar,8Ar)-4-Isopropyl-1,6-Dimethyl-1,2,3,4,4A,7,8,8A-Octahydronaphthalen-1-Ol | NCOA2 |
| Curcuma phaeocaulis Valeton | MOL311 | (1S,4S,4Ar,8Ar)-4-Isopropyl-1,6-Dimethyl-1,2,3,4,4A,7,8,8A-Octahydronaphthalen-1-Ol | NR1I2 |
| Curcuma phaeocaulis Valeton | MOL312 | (1S,4S,4Ar,8Ar)-4-Isopropyl-1,6-Dimethyl-1,2,3,4,4A,7,8,8A-Octahydronaphthalen-1-Ol | NR1I3 |
| Curcuma phaeocaulis Valeton | MOL313 | (1S,4S,4Ar,8Ar)-4-Isopropyl-1,6-Dimethyl-1,2,3,4,4A,7,8,8A-Octahydronaphthalen-1-Ol | NR3C2 |
| Curcuma phaeocaulis Valeton | MOL314 | (1S,4S,4Ar,8Ar)-4-Isopropyl-1,6-Dimethyl-1,2,3,4,4A,7,8,8A-Octahydronaphthalen-1-Ol | PGR |
| Curcuma phaeocaulis Valeton | MOL315 | (1S,4S,4Ar,8Ar)-4-Isopropyl-1,6-Dimethyl-1,2,3,4,4A,7,8,8A-Octahydronaphthalen-1-Ol | RORA |
| Curcuma phaeocaulis Valeton | MOL316 | (1S,4S,4Ar,8Ar)-4-Isopropyl-1,6-Dimethyl-1,2,3,4,4A,7,8,8A-Octahydronaphthalen-1-Ol | SULT2B1 |
| Curcuma phaeocaulis Valeton | MOL317 | (1S,4S,4Ar,8Ar)-4-Isopropyl-1,6-Dimethyl-1,2,3,4,4A,7,8,8A-Octahydronaphthalen-1-Ol | VDR |
| Curcuma phaeocaulis Valeton | MOL318 | (5S,8R,9S,10S,13S,14S)-3-Ethyl-3-Hydroxy-10,13-Dimethyl-Tetradecahydro-2H-Cyclopenta[A]Phenanthren-17(14H)-One | ADH1C |
| Curcuma phaeocaulis Valeton | MOL319 | (5S,8R,9S,10S,13S,14S)-3-Ethyl-3-Hydroxy-10,13-Dimethyl-Tetradecahydro-2H-Cyclopenta[A]Phenanthren-17(14H)-One | AKR1C1 |
| Curcuma phaeocaulis Valeton | MOL320 | (5S,8R,9S,10S,13S,14S)-3-Ethyl-3-Hydroxy-10,13-Dimethyl-Tetradecahydro-2H-Cyclopenta[A]Phenanthren-17(14H)-One | AKR1C2 |
| Curcuma phaeocaulis Valeton | MOL321 | (5S,8R,9S,10S,13S,14S)-3-Ethyl-3-Hydroxy-10,13-Dimethyl-Tetradecahydro-2H-Cyclopenta[A]Phenanthren-17(14H)-One | AKR1D1 |
| Curcuma phaeocaulis Valeton | MOL322 | (5S,8R,9S,10S,13S,14S)-3-Ethyl-3-Hydroxy-10,13-Dimethyl-Tetradecahydro-2H-Cyclopenta[A]Phenanthren-17(14H)-One | AR |
| Curcuma phaeocaulis Valeton | MOL323 | (5S,8R,9S,10S,13S,14S)-3-Ethyl-3-Hydroxy-10,13-Dimethyl-Tetradecahydro-2H-Cyclopenta[A]Phenanthren-17(14H)-One | BCL2 |
| Curcuma phaeocaulis Valeton | MOL324 | (5S,8R,9S,10S,13S,14S)-3-Ethyl-3-Hydroxy-10,13-Dimethyl-Tetradecahydro-2H-Cyclopenta[A]Phenanthren-17(14H)-One | CES1 |
| Curcuma phaeocaulis Valeton | MOL325 | (5S,8R,9S,10S,13S,14S)-3-Ethyl-3-Hydroxy-10,13-Dimethyl-Tetradecahydro-2H-Cyclopenta[A]Phenanthren-17(14H)-One | COX4I1 |
| Curcuma phaeocaulis Valeton | MOL326 | (5S,8R,9S,10S,13S,14S)-3-Ethyl-3-Hydroxy-10,13-Dimethyl-Tetradecahydro-2H-Cyclopenta[A]Phenanthren-17(14H)-One | COX5A |
| Curcuma phaeocaulis Valeton | MOL327 | (5S,8R,9S,10S,13S,14S)-3-Ethyl-3-Hydroxy-10,13-Dimethyl-Tetradecahydro-2H-Cyclopenta[A]Phenanthren-17(14H)-One | COX5B |
| Curcuma phaeocaulis Valeton | MOL328 | (5S,8R,9S,10S,13S,14S)-3-Ethyl-3-Hydroxy-10,13-Dimethyl-Tetradecahydro-2H-Cyclopenta[A]Phenanthren-17(14H)-One | COX6A2 |
| Curcuma phaeocaulis Valeton | MOL329 | (5S,8R,9S,10S,13S,14S)-3-Ethyl-3-Hydroxy-10,13-Dimethyl-Tetradecahydro-2H-Cyclopenta[A]Phenanthren-17(14H)-One | COX6B1 |
| Curcuma phaeocaulis Valeton | MOL330 | (5S,8R,9S,10S,13S,14S)-3-Ethyl-3-Hydroxy-10,13-Dimethyl-Tetradecahydro-2H-Cyclopenta[A]Phenanthren-17(14H)-One | COX6C |
| Curcuma phaeocaulis Valeton | MOL331 | (5S,8R,9S,10S,13S,14S)-3-Ethyl-3-Hydroxy-10,13-Dimethyl-Tetradecahydro-2H-Cyclopenta[A]Phenanthren-17(14H)-One | COX7A1 |
| Curcuma phaeocaulis Valeton | MOL332 | (5S,8R,9S,10S,13S,14S)-3-Ethyl-3-Hydroxy-10,13-Dimethyl-Tetradecahydro-2H-Cyclopenta[A]Phenanthren-17(14H)-One | COX7B |
| Curcuma phaeocaulis Valeton | MOL333 | (5S,8R,9S,10S,13S,14S)-3-Ethyl-3-Hydroxy-10,13-Dimethyl-Tetradecahydro-2H-Cyclopenta[A]Phenanthren-17(14H)-One | COX7C |
| Curcuma phaeocaulis Valeton | MOL334 | (5S,8R,9S,10S,13S,14S)-3-Ethyl-3-Hydroxy-10,13-Dimethyl-Tetradecahydro-2H-Cyclopenta[A]Phenanthren-17(14H)-One | COX8A |
| Curcuma phaeocaulis Valeton | MOL335 | (5S,8R,9S,10S,13S,14S)-3-Ethyl-3-Hydroxy-10,13-Dimethyl-Tetradecahydro-2H-Cyclopenta[A]Phenanthren-17(14H)-One | EFTUD1 |
| Curcuma phaeocaulis Valeton | MOL336 | (5S,8R,9S,10S,13S,14S)-3-Ethyl-3-Hydroxy-10,13-Dimethyl-Tetradecahydro-2H-Cyclopenta[A]Phenanthren-17(14H)-One | ESR1 |
| Curcuma phaeocaulis Valeton | MOL337 | (5S,8R,9S,10S,13S,14S)-3-Ethyl-3-Hydroxy-10,13-Dimethyl-Tetradecahydro-2H-Cyclopenta[A]Phenanthren-17(14H)-One | ESR2 |
| Curcuma phaeocaulis Valeton | MOL338 | (5S,8R,9S,10S,13S,14S)-3-Ethyl-3-Hydroxy-10,13-Dimethyl-Tetradecahydro-2H-Cyclopenta[A]Phenanthren-17(14H)-One | ESRRG |
| Curcuma phaeocaulis Valeton | MOL339 | (5S,8R,9S,10S,13S,14S)-3-Ethyl-3-Hydroxy-10,13-Dimethyl-Tetradecahydro-2H-Cyclopenta[A]Phenanthren-17(14H)-One | FABP6 |
| Curcuma phaeocaulis Valeton | MOL340 | (5S,8R,9S,10S,13S,14S)-3-Ethyl-3-Hydroxy-10,13-Dimethyl-Tetradecahydro-2H-Cyclopenta[A]Phenanthren-17(14H)-One | FECH |
| Curcuma phaeocaulis Valeton | MOL341 | (5S,8R,9S,10S,13S,14S)-3-Ethyl-3-Hydroxy-10,13-Dimethyl-Tetradecahydro-2H-Cyclopenta[A]Phenanthren-17(14H)-One | G6PD |
| Curcuma phaeocaulis Valeton | MOL342 | (5S,8R,9S,10S,13S,14S)-3-Ethyl-3-Hydroxy-10,13-Dimethyl-Tetradecahydro-2H-Cyclopenta[A]Phenanthren-17(14H)-One | GABRA1 |
| Curcuma phaeocaulis Valeton | MOL343 | (5S,8R,9S,10S,13S,14S)-3-Ethyl-3-Hydroxy-10,13-Dimethyl-Tetradecahydro-2H-Cyclopenta[A]Phenanthren-17(14H)-One | GABRA2 |
| Curcuma phaeocaulis Valeton | MOL344 | (5S,8R,9S,10S,13S,14S)-3-Ethyl-3-Hydroxy-10,13-Dimethyl-Tetradecahydro-2H-Cyclopenta[A]Phenanthren-17(14H)-One | GABRA3 |
| Curcuma phaeocaulis Valeton | MOL345 | (5S,8R,9S,10S,13S,14S)-3-Ethyl-3-Hydroxy-10,13-Dimethyl-Tetradecahydro-2H-Cyclopenta[A]Phenanthren-17(14H)-One | GABRA4 |
| Curcuma phaeocaulis Valeton | MOL346 | (5S,8R,9S,10S,13S,14S)-3-Ethyl-3-Hydroxy-10,13-Dimethyl-Tetradecahydro-2H-Cyclopenta[A]Phenanthren-17(14H)-One | GABRA5 |
| Curcuma phaeocaulis Valeton | MOL347 | (5S,8R,9S,10S,13S,14S)-3-Ethyl-3-Hydroxy-10,13-Dimethyl-Tetradecahydro-2H-Cyclopenta[A]Phenanthren-17(14H)-One | GABRA6 |
| Curcuma phaeocaulis Valeton | MOL348 | (5S,8R,9S,10S,13S,14S)-3-Ethyl-3-Hydroxy-10,13-Dimethyl-Tetradecahydro-2H-Cyclopenta[A]Phenanthren-17(14H)-One | GABRB1 |
| Curcuma phaeocaulis Valeton | MOL349 | (5S,8R,9S,10S,13S,14S)-3-Ethyl-3-Hydroxy-10,13-Dimethyl-Tetradecahydro-2H-Cyclopenta[A]Phenanthren-17(14H)-One | GABRB2 |
| Curcuma phaeocaulis Valeton | MOL350 | (5S,8R,9S,10S,13S,14S)-3-Ethyl-3-Hydroxy-10,13-Dimethyl-Tetradecahydro-2H-Cyclopenta[A]Phenanthren-17(14H)-One | GABRB3 |
| Curcuma phaeocaulis Valeton | MOL351 | (5S,8R,9S,10S,13S,14S)-3-Ethyl-3-Hydroxy-10,13-Dimethyl-Tetradecahydro-2H-Cyclopenta[A]Phenanthren-17(14H)-One | GABRD |
| Curcuma phaeocaulis Valeton | MOL352 | (5S,8R,9S,10S,13S,14S)-3-Ethyl-3-Hydroxy-10,13-Dimethyl-Tetradecahydro-2H-Cyclopenta[A]Phenanthren-17(14H)-One | GABRE |
| Curcuma phaeocaulis Valeton | MOL353 | (5S,8R,9S,10S,13S,14S)-3-Ethyl-3-Hydroxy-10,13-Dimethyl-Tetradecahydro-2H-Cyclopenta[A]Phenanthren-17(14H)-One | GABRG1 |
| Curcuma phaeocaulis Valeton | MOL354 | (5S,8R,9S,10S,13S,14S)-3-Ethyl-3-Hydroxy-10,13-Dimethyl-Tetradecahydro-2H-Cyclopenta[A]Phenanthren-17(14H)-One | GABRG2 |
| Curcuma phaeocaulis Valeton | MOL355 | (5S,8R,9S,10S,13S,14S)-3-Ethyl-3-Hydroxy-10,13-Dimethyl-Tetradecahydro-2H-Cyclopenta[A]Phenanthren-17(14H)-One | GABRG3 |
| Curcuma phaeocaulis Valeton | MOL356 | (5S,8R,9S,10S,13S,14S)-3-Ethyl-3-Hydroxy-10,13-Dimethyl-Tetradecahydro-2H-Cyclopenta[A]Phenanthren-17(14H)-One | GABRP |
| Curcuma phaeocaulis Valeton | MOL357 | (5S,8R,9S,10S,13S,14S)-3-Ethyl-3-Hydroxy-10,13-Dimethyl-Tetradecahydro-2H-Cyclopenta[A]Phenanthren-17(14H)-One | GABRQ |
| Curcuma phaeocaulis Valeton | MOL358 | (5S,8R,9S,10S,13S,14S)-3-Ethyl-3-Hydroxy-10,13-Dimethyl-Tetradecahydro-2H-Cyclopenta[A]Phenanthren-17(14H)-One | GPBAR1 |
| Curcuma phaeocaulis Valeton | MOL359 | (5S,8R,9S,10S,13S,14S)-3-Ethyl-3-Hydroxy-10,13-Dimethyl-Tetradecahydro-2H-Cyclopenta[A]Phenanthren-17(14H)-One | GSTP1 |
| Curcuma phaeocaulis Valeton | MOL360 | (5S,8R,9S,10S,13S,14S)-3-Ethyl-3-Hydroxy-10,13-Dimethyl-Tetradecahydro-2H-Cyclopenta[A]Phenanthren-17(14H)-One | HSD17B1 |
| Curcuma phaeocaulis Valeton | MOL361 | (5S,8R,9S,10S,13S,14S)-3-Ethyl-3-Hydroxy-10,13-Dimethyl-Tetradecahydro-2H-Cyclopenta[A]Phenanthren-17(14H)-One | HSD17B11 |
| Curcuma phaeocaulis Valeton | MOL362 | (5S,8R,9S,10S,13S,14S)-3-Ethyl-3-Hydroxy-10,13-Dimethyl-Tetradecahydro-2H-Cyclopenta[A]Phenanthren-17(14H)-One | IGHG2 |
| Curcuma phaeocaulis Valeton | MOL363 | (5S,8R,9S,10S,13S,14S)-3-Ethyl-3-Hydroxy-10,13-Dimethyl-Tetradecahydro-2H-Cyclopenta[A]Phenanthren-17(14H)-One | MT-CO1 |
| Curcuma phaeocaulis Valeton | MOL364 | (5S,8R,9S,10S,13S,14S)-3-Ethyl-3-Hydroxy-10,13-Dimethyl-Tetradecahydro-2H-Cyclopenta[A]Phenanthren-17(14H)-One | MT-CO2 |
| Curcuma phaeocaulis Valeton | MOL365 | (5S,8R,9S,10S,13S,14S)-3-Ethyl-3-Hydroxy-10,13-Dimethyl-Tetradecahydro-2H-Cyclopenta[A]Phenanthren-17(14H)-One | MT-CO3 |
| Curcuma phaeocaulis Valeton | MOL366 | (5S,8R,9S,10S,13S,14S)-3-Ethyl-3-Hydroxy-10,13-Dimethyl-Tetradecahydro-2H-Cyclopenta[A]Phenanthren-17(14H)-One | NCOA2 |
| Curcuma phaeocaulis Valeton | MOL367 | (5S,8R,9S,10S,13S,14S)-3-Ethyl-3-Hydroxy-10,13-Dimethyl-Tetradecahydro-2H-Cyclopenta[A]Phenanthren-17(14H)-One | NR1H4 |
| Curcuma phaeocaulis Valeton | MOL368 | (5S,8R,9S,10S,13S,14S)-3-Ethyl-3-Hydroxy-10,13-Dimethyl-Tetradecahydro-2H-Cyclopenta[A]Phenanthren-17(14H)-One | NR1I2 |
| Curcuma phaeocaulis Valeton | MOL369 | (5S,8R,9S,10S,13S,14S)-3-Ethyl-3-Hydroxy-10,13-Dimethyl-Tetradecahydro-2H-Cyclopenta[A]Phenanthren-17(14H)-One | NR3C1 |
| Curcuma phaeocaulis Valeton | MOL370 | (5S,8R,9S,10S,13S,14S)-3-Ethyl-3-Hydroxy-10,13-Dimethyl-Tetradecahydro-2H-Cyclopenta[A]Phenanthren-17(14H)-One | NR3C2 |
| Curcuma phaeocaulis Valeton | MOL371 | (5S,8R,9S,10S,13S,14S)-3-Ethyl-3-Hydroxy-10,13-Dimethyl-Tetradecahydro-2H-Cyclopenta[A]Phenanthren-17(14H)-One | PGR |
| Curcuma phaeocaulis Valeton | MOL372 | (5S,8R,9S,10S,13S,14S)-3-Ethyl-3-Hydroxy-10,13-Dimethyl-Tetradecahydro-2H-Cyclopenta[A]Phenanthren-17(14H)-One | PLA2G1B |
| Curcuma phaeocaulis Valeton | MOL373 | (5S,8R,9S,10S,13S,14S)-3-Ethyl-3-Hydroxy-10,13-Dimethyl-Tetradecahydro-2H-Cyclopenta[A]Phenanthren-17(14H)-One | SULT2A1 |
| Curcuma phaeocaulis Valeton | MOL374 | (5S,8R,9S,10S,13S,14S)-3-Ethyl-3-Hydroxy-10,13-Dimethyl-Tetradecahydro-2H-Cyclopenta[A]Phenanthren-17(14H)-One | SULT2B1 |
| Curcuma phaeocaulis Valeton | MOL375 | (5S,8R,9S,10S,13S,14S)-3-Ethyl-3-Hydroxy-10,13-Dimethyl-Tetradecahydro-2H-Cyclopenta[A]Phenanthren-17(14H)-One | Bicyclo[3.3.1]Nonan-2-Ol |
| Curcuma phaeocaulis Valeton | MOL376 | (5S,8R,9S,10S,13S,14S)-3-Ethyl-3-Hydroxy-10,13-Dimethyl-Tetradecahydro-2H-Cyclopenta[A]Phenanthren-17(14H)-One | ADH1B |
| Curcuma phaeocaulis Valeton | MOL377 | (S)-5,7-Dihydroxy-2-Phenylchroman-4-One,Pinocembrin | ACTB |
| Curcuma phaeocaulis Valeton | MOL378 | (S)-5,7-Dihydroxy-2-Phenylchroman-4-One,Pinocembrin | AHR |
| Curcuma phaeocaulis Valeton | MOL379 | (S)-5,7-Dihydroxy-2-Phenylchroman-4-One,Pinocembrin | AKR1C1 |
| Curcuma phaeocaulis Valeton | MOL380 | (S)-5,7-Dihydroxy-2-Phenylchroman-4-One,Pinocembrin | AKT1 |
| Curcuma phaeocaulis Valeton | MOL381 | (S)-5,7-Dihydroxy-2-Phenylchroman-4-One,Pinocembrin | ATP5A1 |
| Curcuma phaeocaulis Valeton | MOL382 | (S)-5,7-Dihydroxy-2-Phenylchroman-4-One,Pinocembrin | ATP5B |
| Curcuma phaeocaulis Valeton | MOL383 | (S)-5,7-Dihydroxy-2-Phenylchroman-4-One,Pinocembrin | ATP5C1 |
| Curcuma phaeocaulis Valeton | MOL384 | (S)-5,7-Dihydroxy-2-Phenylchroman-4-One,Pinocembrin | CBR1 |
| Curcuma phaeocaulis Valeton | MOL385 | (S)-5,7-Dihydroxy-2-Phenylchroman-4-One,Pinocembrin | CDK6 |
| Curcuma phaeocaulis Valeton | MOL386 | (S)-5,7-Dihydroxy-2-Phenylchroman-4-One,Pinocembrin | CEBPB |
| Curcuma phaeocaulis Valeton | MOL387 | (S)-5,7-Dihydroxy-2-Phenylchroman-4-One,Pinocembrin | CSNK2A1 |
| Curcuma phaeocaulis Valeton | MOL388 | (S)-5,7-Dihydroxy-2-Phenylchroman-4-One,Pinocembrin | CSNK2B |
| Curcuma phaeocaulis Valeton | MOL389 | (S)-5,7-Dihydroxy-2-Phenylchroman-4-One,Pinocembrin | CYP19A1 |
| Curcuma phaeocaulis Valeton | MOL390 | (S)-5,7-Dihydroxy-2-Phenylchroman-4-One,Pinocembrin | CYP1B1 |
| Curcuma phaeocaulis Valeton | MOL391 | (S)-5,7-Dihydroxy-2-Phenylchroman-4-One,Pinocembrin | EIF3F |
| Curcuma phaeocaulis Valeton | MOL392 | (S)-5,7-Dihydroxy-2-Phenylchroman-4-One,Pinocembrin | ESR1 |
| Curcuma phaeocaulis Valeton | MOL393 | (S)-5,7-Dihydroxy-2-Phenylchroman-4-One,Pinocembrin | ESR2 |
| Curcuma phaeocaulis Valeton | MOL394 | (S)-5,7-Dihydroxy-2-Phenylchroman-4-One,Pinocembrin | ESRRA |
| Curcuma phaeocaulis Valeton | MOL395 | (S)-5,7-Dihydroxy-2-Phenylchroman-4-One,Pinocembrin | ESRRB |
| Curcuma phaeocaulis Valeton | MOL396 | (S)-5,7-Dihydroxy-2-Phenylchroman-4-One,Pinocembrin | GPER1 |
| Curcuma phaeocaulis Valeton | MOL397 | (S)-5,7-Dihydroxy-2-Phenylchroman-4-One,Pinocembrin | HCK |
| Curcuma phaeocaulis Valeton | MOL398 | (S)-5,7-Dihydroxy-2-Phenylchroman-4-One,Pinocembrin | HIBCH |
| Curcuma phaeocaulis Valeton | MOL399 | (S)-5,7-Dihydroxy-2-Phenylchroman-4-One,Pinocembrin | HSP90AA1 |
| Curcuma phaeocaulis Valeton | MOL400 | (S)-5,7-Dihydroxy-2-Phenylchroman-4-One,Pinocembrin | HSPA2 |
| Curcuma phaeocaulis Valeton | MOL401 | (S)-5,7-Dihydroxy-2-Phenylchroman-4-One,Pinocembrin | IGHG1 |
| Curcuma phaeocaulis Valeton | MOL402 | (S)-5,7-Dihydroxy-2-Phenylchroman-4-One,Pinocembrin | JAK1 |
| Curcuma phaeocaulis Valeton | MOL403 | (S)-5,7-Dihydroxy-2-Phenylchroman-4-One,Pinocembrin | KANSL3 |
| Curcuma phaeocaulis Valeton | MOL404 | (S)-5,7-Dihydroxy-2-Phenylchroman-4-One,Pinocembrin | MTTP |
| Curcuma phaeocaulis Valeton | MOL405 | (S)-5,7-Dihydroxy-2-Phenylchroman-4-One,Pinocembrin | NCOA1 |
| Curcuma phaeocaulis Valeton | MOL406 | (S)-5,7-Dihydroxy-2-Phenylchroman-4-One,Pinocembrin | NCOA2 |
| Curcuma phaeocaulis Valeton | MOL407 | (S)-5,7-Dihydroxy-2-Phenylchroman-4-One,Pinocembrin | NQO2 |
| Curcuma phaeocaulis Valeton | MOL408 | (S)-5,7-Dihydroxy-2-Phenylchroman-4-One,Pinocembrin | NR1I2 |
| Curcuma phaeocaulis Valeton | MOL409 | (S)-5,7-Dihydroxy-2-Phenylchroman-4-One,Pinocembrin | PIK3CG |
| Curcuma phaeocaulis Valeton | MOL410 | (S)-5,7-Dihydroxy-2-Phenylchroman-4-One,Pinocembrin | PIM1 |
| Curcuma phaeocaulis Valeton | MOL411 | (S)-5,7-Dihydroxy-2-Phenylchroman-4-One,Pinocembrin | PTK2B |
| Curcuma phaeocaulis Valeton | MOL412 | (S)-5,7-Dihydroxy-2-Phenylchroman-4-One,Pinocembrin | RUVBL2 |
| Curcuma phaeocaulis Valeton | MOL413 | (S)-5,7-Dihydroxy-2-Phenylchroman-4-One,Pinocembrin | SF3B3 |
| Curcuma phaeocaulis Valeton | MOL414 | (S)-5,7-Dihydroxy-2-Phenylchroman-4-One,Pinocembrin | SHBG |
| Curcuma phaeocaulis Valeton | MOL415 | (S)-5,7-Dihydroxy-2-Phenylchroman-4-One,Pinocembrin | SOAT1 |
| Curcuma phaeocaulis Valeton | MOL416 | (S)-5,7-Dihydroxy-2-Phenylchroman-4-One,Pinocembrin | SOAT2 |
| Curcuma phaeocaulis Valeton | MOL417 | (S)-5,7-Dihydroxy-2-Phenylchroman-4-One,Pinocembrin | STK17B |
| Curcuma phaeocaulis Valeton | MOL418 | (S)-5,7-Dihydroxy-2-Phenylchroman-4-One,Pinocembrin | TOP2A |
| Curcuma phaeocaulis Valeton | MOL419 | (S)-5,7-Dihydroxy-2-Phenylchroman-4-One,Pinocembrin | UBA1 |
| Curcuma phaeocaulis Valeton | MOL420 | (S)-5,7-Dihydroxy-2-Phenylchroman-4-One,Pinocembrin | UGT3A1 |
| Curcuma phaeocaulis Valeton | MOL421 | (S)-5,7-Dihydroxy-2-Phenylchroman-4-One,Pinocembrin | Nonan-2-One |
| Curcuma phaeocaulis Valeton | MOL422 | (S)-5,7-Dihydroxy-2-Phenylchroman-4-One,Pinocembrin | ACOT13 |
| Curcuma phaeocaulis Valeton | MOL423 | (S)-5,7-Dihydroxy-2-Phenylchroman-4-One,Pinocembrin | DBI |
| Curcuma phaeocaulis Valeton | MOL424 | (S)-5,7-Dihydroxy-2-Phenylchroman-4-One,Pinocembrin | RHO |
| Curcuma phaeocaulis Valeton | MOL425 | Methyl-N-Nonylketone,Undecan-2-One | ACOT13 |
| Curcuma phaeocaulis Valeton | MOL426 | Methyl-N-Nonylketone,Undecan-2-One | DBI |
| Curcuma phaeocaulis Valeton | MOL427 | Methyl-N-Nonylketone,Undecan-2-One | RHO |
| Curcuma phaeocaulis Valeton | MOL428 | Methyl-N-Nonylketone,Undecan-2-One | Decan-2-One |
| Curcuma phaeocaulis Valeton | MOL429 | Methyl-N-Nonylketone,Undecan-2-One | ACOT13 |
| Curcuma phaeocaulis Valeton | MOL430 | Methyl-N-Nonylketone,Undecan-2-One | DBI |
| Curcuma phaeocaulis Valeton | MOL431 | Methyl-N-Nonylketone,Undecan-2-One | RHO |
| Curcuma phaeocaulis Valeton | MOL432 | Methyl-N-Nonylketone,Undecan-2-One | Octanal |
| Curcuma phaeocaulis Valeton | MOL433 | Methyl-N-Nonylketone,Undecan-2-One | ACOT13 |
| Curcuma phaeocaulis Valeton | MOL434 | Methyl-N-Nonylketone,Undecan-2-One | DBI |
| Curcuma phaeocaulis Valeton | MOL435 | Methyl-N-Nonylketone,Undecan-2-One | RHO |
| Curcuma phaeocaulis Valeton | MOL436 | 1,7-Bis(4-Hydroxy-3-Methoxyphenyl)Hepta-1,6-Diene-3,5-Dione | ABCC5 |
| Curcuma phaeocaulis Valeton | MOL437 | 1,7-Bis(4-Hydroxy-3-Methoxyphenyl)Hepta-1,6-Diene-3,5-Dione | AR |
| Curcuma phaeocaulis Valeton | MOL438 | 1,7-Bis(4-Hydroxy-3-Methoxyphenyl)Hepta-1,6-Diene-3,5-Dione | CBR1 |
| Curcuma phaeocaulis Valeton | MOL439 | 1,7-Bis(4-Hydroxy-3-Methoxyphenyl)Hepta-1,6-Diene-3,5-Dione | GSTP1 |
| Curcuma phaeocaulis Valeton | MOL440 | 1,7-Bis(4-Hydroxy-3-Methoxyphenyl)Hepta-1,6-Diene-3,5-Dione | PPARG |
| Curcuma phaeocaulis Valeton | MOL441 | 1,7-Bis(4-Hydroxy-3-Methoxyphenyl)Hepta-1,6-Diene-3,5-Dione | TUBB |
| Curcuma phaeocaulis Valeton | MOL442 | 1,7-Bis(4-Hydroxy-3-Methoxyphenyl)Hepta-1,6-Diene-3,5-Dione | VDR |
| Curcuma phaeocaulis Valeton | MOL443 | 1-(4-Hydroxy-3-Methoxyphenyl)-7-(4-Hydroxyphenyl)Hepta-1,6-Diene-3,5-Dione | ABCC5 |
| Curcuma phaeocaulis Valeton | MOL444 | 1-(4-Hydroxy-3-Methoxyphenyl)-7-(4-Hydroxyphenyl)Hepta-1,6-Diene-3,5-Dione | CBR1 |
| Curcuma phaeocaulis Valeton | MOL445 | 1-(4-Hydroxy-3-Methoxyphenyl)-7-(4-Hydroxyphenyl)Hepta-1,6-Diene-3,5-Dione | GSTP1 |
| Curcuma phaeocaulis Valeton | MOL446 | 1-(4-Hydroxy-3-Methoxyphenyl)-7-(4-Hydroxyphenyl)Hepta-1,6-Diene-3,5-Dione | PPARG |
| Curcuma phaeocaulis Valeton | MOL447 | 1-(4-Hydroxy-3-Methoxyphenyl)-7-(4-Hydroxyphenyl)Hepta-1,6-Diene-3,5-Dione | SHBG |
| Curcuma phaeocaulis Valeton | MOL448 | 1-(4-Hydroxy-3-Methoxyphenyl)-7-(4-Hydroxyphenyl)Hepta-1,6-Diene-3,5-Dione | VDR |

# Supplementary 5

**Core active components**

**Supplementary table 5. Drug pair of AM-CPV and GC of core active components**

| PubChem ID | NAME | OB | DL | Source | Structure |
| --- | --- | --- | --- | --- | --- |
| 5281654 | isorhamnetin | 49.6 | 0.31 | TCMSP | 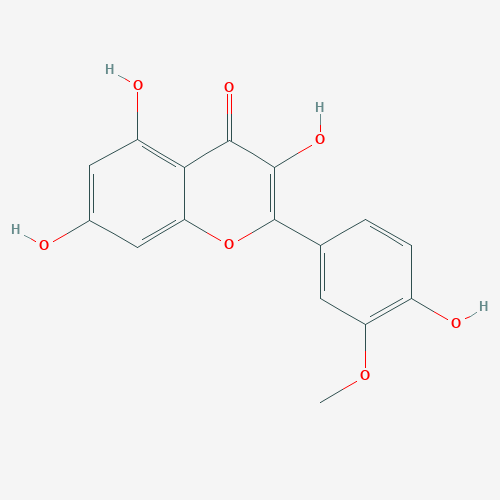 |
| 15689652 | 7-O-methylisomucronulatol | 74.69 | 0.3 | TCMSP | 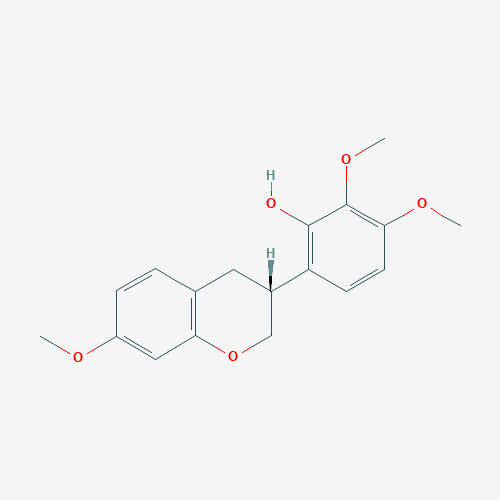 |
| 5280378 | formononetin | 69.67 | 0.21 | TCMSP | 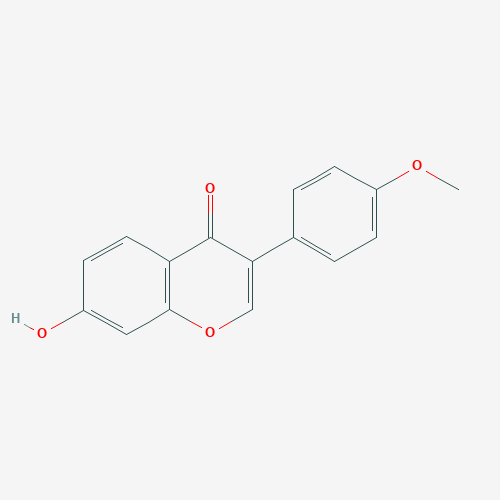 |
| 442811 | Mucronulatol | NA | NA | ETCM | 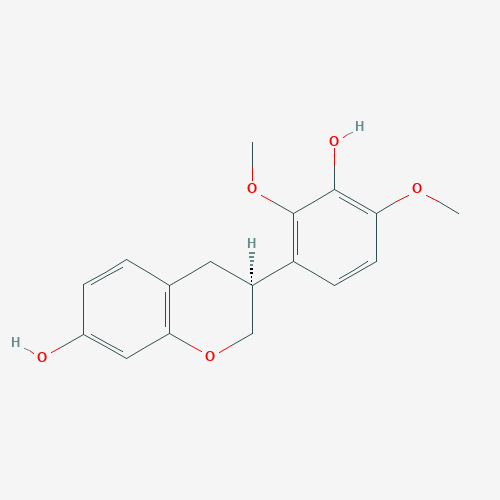 |
| 5280448 | Calycosin | 47.75 | 0.24 | TCMSP | 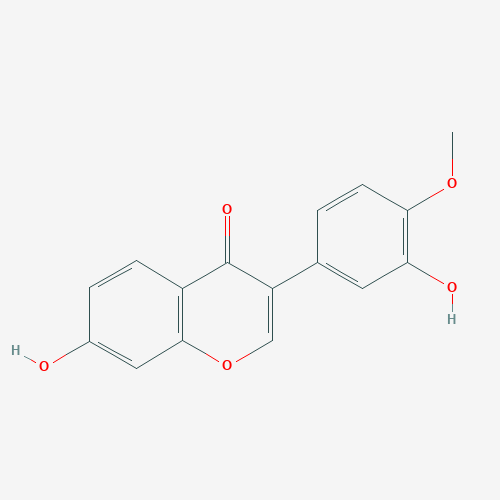 |
| 5280863 | kaempferol | 41.88 | 0.24 | TCMSP | 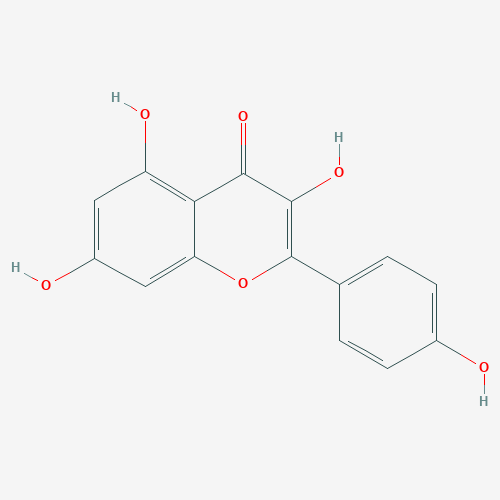 |
| 5280343 | quercetin | 46.43 | 0.28 | TCMSP | 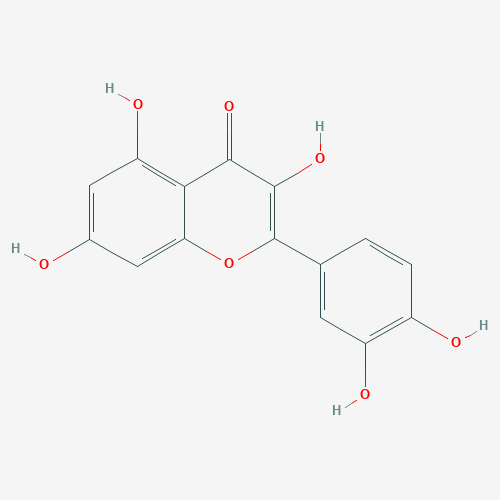 |
| 312601999 | Borneol | NA | NA | ETCM | 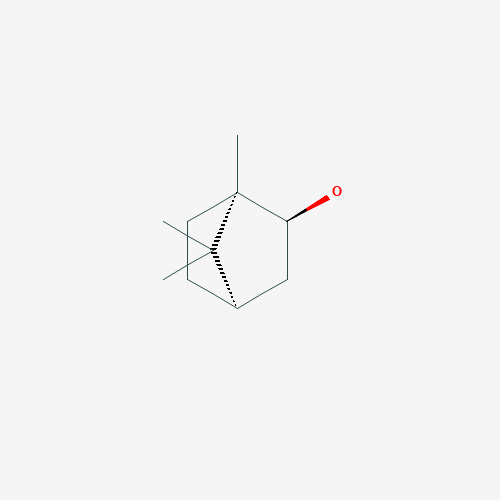 |
| 6321405 | Isoborneol | NA | NA | TCMID | 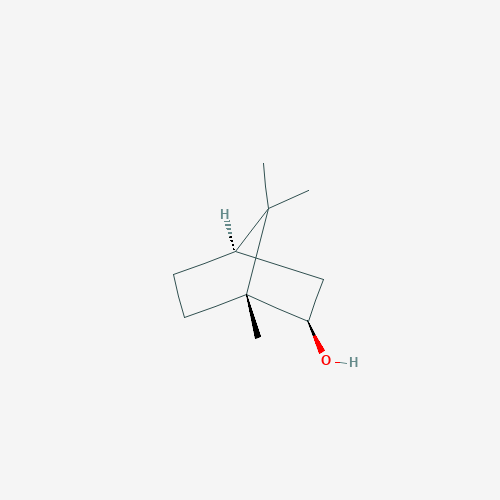 |
| 23500184 | BornylAcetate | NA | NA | ETCM | 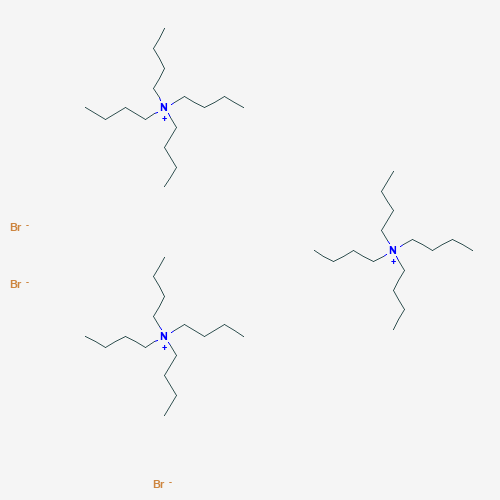 |
| 154279 | Alpinetin | NA | NA | ETCM | 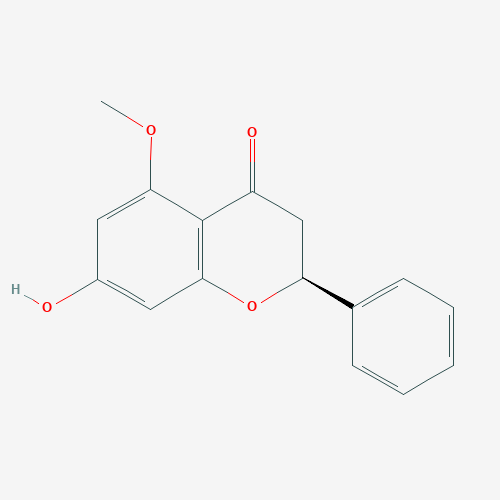 |
| 68071 | Pinocembrin | NA | NA | ETCM | 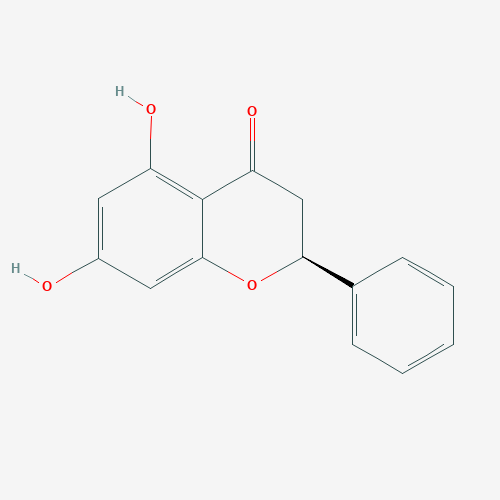 |
| 91711266 | (R)-3,5,8a-Trimethyl-7,8,8a,9-tetrahydronaphtho[2,3-b]furan-4(6H)-one | NA | NA | TCMID | 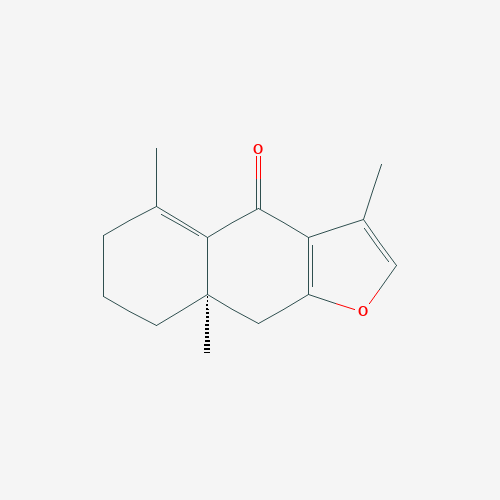 |
| 14681481 | (5S,8R,9S,10S,13S,14S)-3-Ethyl-3-Hydroxy-10 | NA | NA | TCMID | 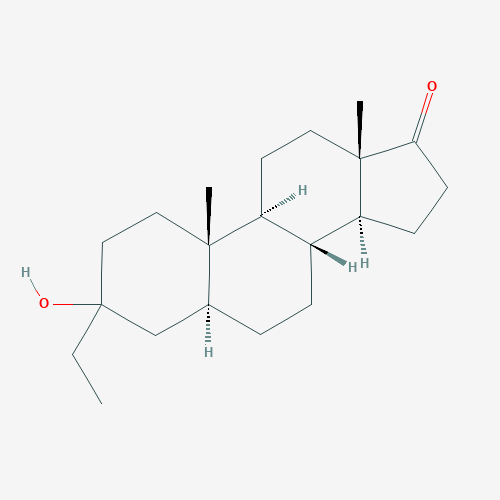 |
| 12302222 | tau-Cadinol | NA | NA | ETCM | 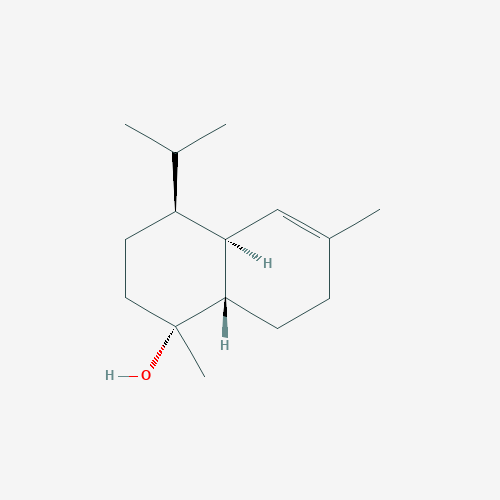 |
| 12304220 | cis-Cubenol | NA | NA | ETCM | 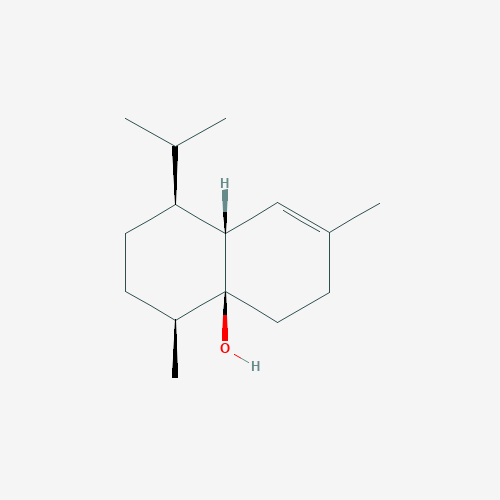 |
| 9992974 | (4Ar,5R,5As,6Ar)-6A-Hydroxy-3,5A-Dimethyl-5-(3-Oxobutyl)-4,4A,5,5A,6,6A-Hexahydro-2H-Cyclopropa[F][1]Benzofuran-2-One | NA | NA | ETCM | 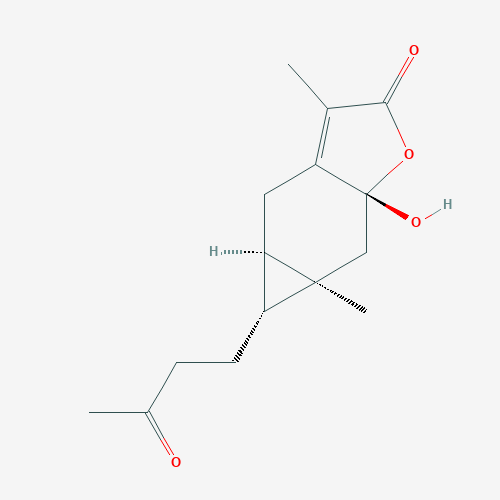 |

# Supplementary 6

**Differential genes of GC overlapped by TCGA and GEO**

**Supplementary table 6. Differential genes of GC overlapped by TCGA and GEO**

| Gene Symbol | Protein name |
| --- | --- |
| ATP4A | Potassium-transporting ATPase alpha chain 1 |
| CLDN1 | Claudin-1 |
| CKM | Creatine kinase M-type |
| CSPG4 | Chondroitin sulfate proteoglycan 4 |
| ETV4 | ETS translocation variant 4 |
| BGN | Biglycan |
| COL1A1 | Collagen alpha-1(I) chain |
| CPA1 | Carboxypeptidase A1 |
| KCNJ13 | Inward rectifier potassium channel 13 |
| FGB | Fibrinogen beta chain |
| LOX | Protein-lysine 6-oxidase |
| CPA2 | Carboxypeptidase A2 |
| PRRX1 | Paired mesoderm homeobox protein 1 |
| ATP4B | Potassium-transporting ATPase subunit beta |
| COL11A1 | Collagen alpha-1(XI) chain |
| KCNE2 | Potassium voltage-gated channel subfamily E member 2 |
| ATP1A2 | Sodium/potassium-transporting ATPase subunit alpha-2 |
| OSMR | Oncostatin-M-specific receptor subunit beta |
| MMP3 | Stromelysin-1 |
| MTHFD1L | Monofunctional C1-tetrahydrofolate synthase, mitochondrial |
| CDA | Cytidine deaminase |
| ECT2 | Protein ECT2 |
| LIPF | Gastric triacylglycerol lipase |
| CST1 | Cystatin-SN |
| AQP4 | Aquaporin-4 |
| MYO1B | Unconventional myosin-Ib |
| SULF1 | Extracellular sulfatase Sulf-1 |
| COL1A2 | Collagen alpha-2(I) chain |
| FAR2 | Fatty acyl-CoA reductase 2 |
| TIMP1 | Metalloproteinase inhibitor 1 |
| DEPDC1 | DEP domain-containing protein 1A |
| MMP10 | Stromelysin-2 |
| THBS2 | Thrombospondin-2 |
| MEST | Mesoderm-specific transcript homolog protein |
| KIF2C | Kinesin-like protein KIF2C |
| ITGA2 | Integrin alpha-2 |
| CCNA2 | Cyclin-A2 |
| CDC20 | Cell division cycle protein 20 homolog |
| SUV39H2 | Histone-lysine N-methyltransferase SUV39H2 |
| PRC1 | Protein regulator of cytokinesis 1 |
| GPRC5A | Retinoic acid-induced protein 3 |
| MFAP2 | Microfibrillar-associated protein 2 |
| ESRRG | Estrogen-related receptor gamma |
| FAP | Prolyl endopeptidase FAP |
| COL4A1 | Collagen alpha-1(IV) chain |
| TYMS | Thymidylate synthase |
| GKN2 | Gastrokine-2 |
| CXCL9 | C-X-C motif chemokine 9 |
| THY1 | Thy-1 membrane glycoprotein |
| TPX2 | Targeting protein for Xklp2 |
| LAMC2 | Laminin subunit gamma-2 |
| CDH11 | Cadherin-11 |
| SPP1 | Osteopontin |
| SERPINH1 | Serpin H1 |
| GKN1 | Gastrokine-1 |
| INHBA | Inhibin beta A chain |
| PLAUR | Urokinase plasminogen activator surface receptor |
| PMEPA1 | Protein TMEPAI |
| LY6E | Lymphocyte antigen 6E |
| MMP12 | Macrophage metalloelastase |
| PLAU | Urokinase-type plasminogen activator |
| CEACAM6 | Carcinoembryonic antigen-related cell adhesion molecule 6 |
| PLA2G7 | Platelet-activating factor acetylhydrolase |
| COL10A1 | Collagen alpha-1(X) chain |
| SFRP4 | Secreted frizzled-related protein 4 |
| MMP9 | Matrix metalloproteinase-9 |
| CXCL10 | C-X-C motif chemokine 10 |
| CTHRC1 | Collagen triple helix repeat-containing protein 1 |
| STAT1 | Signal transducer and activator of transcription 1-alpha/beta |
| COL9A3 | Collagen alpha-3(IX) chain |
| ANXA1 | Annexin A1 |
| MXRA5 | Matrix-remodeling-associated protein 5 |
| SPARC | SPARC |
| PSCA | Annexin A1 |
| XPO5 | Exportin-5 |
| MT1H | Metallothionein-1H |
| CLDN12 | Claudin-12 |
| MMP7 | Matrilysin |
| COL12A1 | Creatine kinase B-type |
| CKB | Creatine kinase B-type |
| CEACAM5 | Carcinoembryonic antigen-related cell adhesion molecule 5 |
| CKS2 | Cyclin-dependent kinases regulatory subunit 2 |
| PODXL | Podocalyxin |

# Supplementary 7

**121 composite targets of drug pair of AM-CPV and GC**

**Supplementary table 7. 121 composite targets of drug pair of AM-CPV and GC.**

| SUID | Gene symbol | Betweenness | Closeness | Degree |
| --- | --- | --- | --- | --- |
| 198105 | HSP90AA1 | 2876.135594 | 0.061728395 | 22 |
| 198221 | IL6 | 764.5919562 | 0.06097561 | 19 |
| 198223 | CXCL8 | 399.3942079 | 0.060331825 | 17 |
| 198196 | CCL2 | 261.2754548 | 0.05988024 | 15 |
| 198072 | CYP3A4 | 1485.803004 | 0.05923001 | 14 |
| 198098 | ERBB2 | 1370.656283 | 0.061193269 | 14 |
| 198138 | JUN | 582.4054604 | 0.060698027 | 14 |
| 198141 | CDK1 | 1455.03604 | 0.059970015 | 13 |
| 198205 | MMP9 | 680.4949606 | 0.06006006 | 13 |
| 198209 | CRP | 925.5958833 | 0.059701493 | 13 |
| 198122 | AR | 1500.700277 | 0.061130922 | 11 |
| 198150 | CCNB1 | 31.747704 | 0.058195926 | 11 |
| 198152 | CCNA2 | 31.747704 | 0.058195926 | 11 |
| 198211 | IL2 | 909.576217 | 0.060483871 | 11 |
| 198213 | IFNG | 151.6009186 | 0.060210738 | 11 |
| 198236 | CDKN1A | 276.3235193 | 0.059671805 | 11 |
| 198320 | FGF2 | 93.35561045 | 0.059464817 | 11 |
| 198111 | CYP1A1 | 187.7400661 | 0.058365759 | 10 |
| 198154 | CHEK1 | 150.3906673 | 0.059376546 | 10 |
| 198199 | MMP1 | 99.90157687 | 0.0591133 | 10 |
| 198267 | STAT1 | 233.0508302 | 0.06027122 | 10 |
| 198102 | F2 | 361.016482 | 0.058737151 | 9 |
| 198113 | AKR1C3 | 582.9348947 | 0.059376546 | 9 |
| 198127 | CYP19A1 | 930.1365412 | 0.060150376 | 9 |
| 198133 | CAV1 | 1016.695645 | 0.060090135 | 9 |
| 198149 | BIRC5 | 301.7678051 | 0.058823529 | 9 |
| 198156 | TYMS | 97.70243914 | 0.057999033 | 9 |
| 198162 | TK1 | 264.9876551 | 0.058337385 | 9 |
| 198197 | SERPINE1 | 100.4844642 | 0.058852379 | 9 |
| 198217 | IL1A | 28.16827069 | 0.059200789 | 9 |
| 198219 | CXCL10 | 217.7786427 | 0.058939096 | 9 |
| 198233 | E2F1 | 30.17105377 | 0.057692308 | 9 |
| 198082 | GSTM1 | 521.8242322 | 0.057859209 | 8 |
| 198129 | CDK6 | 178.1208503 | 0.059850374 | 8 |
| 198160 | FOS | 590.6654214 | 0.05973121 | 8 |
| 198164 | TOP2A | 297.0362067 | 0.059405941 | 8 |
| 198207 | MMP3 | 84.12761107 | 0.05902607 | 8 |
| 198078 | GSTM2 | 85.0665697 | 0.057007126 | 7 |
| 198086 | MAOA | 397.8738399 | 0.057197331 | 7 |
| 198096 | CACNA1C | 100.3218004 | 0.057831325 | 7 |
| 198109 | CYP17A1 | 229.8379988 | 0.059317845 | 7 |
| 198075 | DPP4 | 614.2824154 | 0.058139535 | 6 |
| 198080 | MAOB | 170.402672 | 0.057170081 | 6 |
| 198188 | F2R | 300.5478023 | 0.058881256 | 6 |
| 198227 | CHEK2 | 1.285714286 | 0.057061341 | 6 |
| 198351 | CYP2C19 | 160.8279471 | 0.057361377 | 6 |
| 198388 | IGF2 | 52.50628218 | 0.058910162 | 6 |
| 198077 | ADH1B | 30.72782828 | 0.055736182 | 5 |
| 198084 | ADH1C | 30.72782828 | 0.055736182 | 5 |
| 198107 | AKR1C1 | 25.11382302 | 0.05730659 | 5 |
| 198125 | CYP1B1 | 32.16916103 | 0.057224607 | 5 |
| 198169 | HSPA2 | 206.851698 | 0.058939096 | 5 |
| 198172 | CACNA1D | 32.63124098 | 0.057416268 | 5 |
| 198201 | CXCL11 | 69.54646575 | 0.058508045 | 5 |
| 198203 | SELE | 41.09097774 | 0.058337385 | 5 |
| 198269 | PARP1 | 45.90327503 | 0.05899705 | 5 |
| 198285 | SPP1 | 486.6996989 | 0.058565154 | 5 |
| 198405 | MET | 54.19053806 | 0.059464817 | 5 |
| 198095 | ADRB2 | 297.9414243 | 0.058910162 | 4 |
| 198143 | SHBG | 351.7330021 | 0.05958292 | 4 |
| 198174 | CALM1 | 269.469352 | 0.058939096 | 4 |
| 198191 | IGFBP3 | 33.04099874 | 0.058451047 | 4 |
| 198330 | SULT2A1 | 19.99843048 | 0.057471264 | 4 |
| 198336 | PGR | 81.19696417 | 0.059850374 | 4 |
| 198375 | SLC6A3 | 446.7391782 | 0.057142857 | 4 |
| 198377 | GRIN2A | 55.63504274 | 0.054078414 | 4 |
| 198400 | THBD | 9.241327967 | 0.057115659 | 4 |
| 198419 | PRKCB | 1098.003326 | 0.058708415 | 4 |
| 198435 | GRIA2 | 514.6418803 | 0.056048575 | 4 |
| 198118 | CBR1 | 0 | 0.057251908 | 3 |
| 198167 | KCNH2 | 19.61713946 | 0.05902607 | 3 |
| 198176 | CACNB2 | 0 | 0.056550424 | 3 |
| 198247 | CDA | 110.4623731 | 0.056630486 | 3 |
| 198251 | TYMP | 4.682417582 | 0.055970149 | 3 |
| 198284 | COL1A1 | 210 | 0.057609217 | 3 |
| 198361 | NR1I3 | 552.151065 | 0.059820538 | 3 |
| 198370 | MGAM | 16.92380952 | 0.055452865 | 3 |
| 198372 | DRD1 | 117.2943592 | 0.054644809 | 3 |
| 198385 | FGF1 | 2.105627706 | 0.058280719 | 3 |
| 198397 | PLAU | 0.835099624 | 0.057388809 | 3 |
| 198425 | GABRA3 | 4 | 0.008474576 | 3 |
| 198436 | GRIN3B | 0 | 0.05352364 | 3 |
| 198438 | GRIN2D | 0 | 0.05352364 | 3 |
| 198115 | AKR1C2 | 0 | 0.056470588 | 2 |
| 198135 | NR3C2 | 0 | 0.058910162 | 2 |
| 198145 | BCL2 | 816 | 0.057197331 | 2 |
| 198146 | RXRG | 618 | 0.054619936 | 2 |
| 198158 | PIM1 | 0 | 0.057279236 | 2 |
| 198183 | PYGM | 32.31439275 | 0.056311591 | 2 |
| 198249 | CES1 | 130.5821422 | 0.057034221 | 2 |
| 198293 | PLA2G1B | 142.1671616 | 0.05733397 | 2 |
| 198356 | PTGS1 | 58.73501662 | 0.055970149 | 2 |
| 198366 | GPBAR1 | 210 | 0.049979175 | 2 |
| 198368 | SLC5A2 | 0 | 0.055401662 | 2 |
| 198373 | SLC6A4 | 48.74074074 | 0.054694622 | 2 |
| 198426 | GABRB1 | 0 | 0.008473978 | 2 |
| 198428 | GABRA5 | 0 | 0.008473978 | 2 |
| 198433 | NR1H4 | 416 | 0.052219321 | 2 |
| 198071 | ABCG2 | 0 | 0.056311591 | 1 |
| 198074 | ADA | 0 | 0.055325035 | 1 |
| 198092 | ADRA1A | 0 | 0.008333333 | 1 |
| 198093 | ADRA1D | 0 | 0.008333333 | 1 |
| 198100 | ADRB3 | 0 | 0.056022409 | 1 |
| 198104 | AHSA1 | 0 | 0.058565154 | 1 |
| 198282 | CHRM2 | 0 | 0.055996267 | 1 |
| 198288 | COL3A1 | 0 | 0.054844607 | 1 |
| 198297 | NPPB | 0 | 0.056737589 | 1 |
| 198303 | OLR1 | 0 | 0.056737589 | 1 |
| 198341 | GPER1 | 0 | 0.057142857 | 1 |
| 198365 | DIO1 | 0 | 0.047885076 | 1 |
| 198415 | FOLR2 | 0 | 0.008333333 | 1 |
| 198416 | SLC46A1 | 0 | 0.008333333 | 1 |
| 198430 | GABRQ | 0 | 0.008473379 | 1 |
| 198447 | HK2 | 0 | 0.008333333 | 1 |
| 198448 | SLC2A4 | 0 | 0.008333333 | 1 |
| 198455 | RUNX1T1 | 0 | 0.058565154 | 1 |
| 198506 | MTTP | 0 | 0.008333333 | 1 |
| 198507 | NPC1L1 | 0 | 0.008333333 | 1 |
| 198515 | SERPINA6 | 0 | 0.056630486 | 1 |
| 198522 | TRPM8 | 0 | 0.008333333 | 1 |
| 198523 | TRPV3 | 0 | 0.008333333 | 1 |

# Supplementary 8

**Filter once after the 30 core target gene of AM-CPV and GC**

**Supplementary table 8. Filter once after the 30 core target gene of AM-CPV and GC.**

| SUID | Gene symbol | Betweenness | Closeness | Degree |
| --- | --- | --- | --- | --- |
| 198221 | IL6 | 103.09778 | 0.630434783 | 16 |
| 198223 | CXCL8 | 35.55154568 | 0.547169811 | 13 |
| 198138 | JUN | 107.3696859 | 0.604166667 | 13 |
| 198105 | HSP90AA1 | 156.2719447 | 0.630434783 | 12 |
| 198196 | CCL2 | 28.45301643 | 0.527272727 | 12 |
| 198213 | IFNG | 20.22017427 | 0.527272727 | 10 |
| 198205 | MMP9 | 21.33378843 | 0.517857143 | 10 |
| 198098 | ERBB2 | 127.0713758 | 0.591836735 | 9 |
| 198211 | IL2 | 13.76360861 | 0.517857143 | 9 |
| 198267 | STAT1 | 25.04070929 | 0.547169811 | 9 |
| 198141 | CDK1 | 71.1282634 | 0.5 | 9 |
| 198122 | AR | 112.3199578 | 0.537037037 | 8 |
| 198207 | MMP3 | 1.233333333 | 0.426470588 | 7 |
| 198209 | CRP | 1.733333333 | 0.426470588 | 7 |
| 198199 | MMP1 | 2.642135642 | 0.46031746 | 7 |
| 198320 | FGF2 | 6.711904762 | 0.475409836 | 7 |
| 198236 | CDKN1A | 11.91180486 | 0.491525424 | 6 |
| 198164 | TOP2A | 37.11829004 | 0.453125 | 6 |
| 198219 | CXCL10 | 0.666666667 | 0.426470588 | 6 |
| 198154 | CHEK1 | 15.771004 | 0.453125 | 6 |
| 198149 | BIRC5 | 23.41650849 | 0.439393939 | 6 |
| 198197 | SERPINE1 | 0.4 | 0.420289855 | 6 |
| 198160 | FOS | 37.91650849 | 0.50877193 | 5 |
| 198156 | TYMS | 0.5 | 0.381578947 | 5 |
| 198129 | CDK6 | 4.572893773 | 0.5 | 5 |
| 198162 | TK1 | 0 | 0.376623377 | 4 |
| 198127 | CYP19A1 | 13.78376623 | 0.420289855 | 4 |
| 198113 | AKR1C3 | 0 | 0.3625 | 3 |
| 198109 | CYP17A1 | 0 | 0.3625 | 3 |
| 198169 | HSPA2 | 0 | 0.391891892 | 1 |

# Supplementary 9

**Filter twice after the 8 core target genes**

**Supplementary table 9. Filter twice after the 8 core target genes.**

| SUID | Gene symbol | Protein name | Betweenness | Closeness | Degree |
| --- | --- | --- | --- | --- | --- |
| 198221 | IL6 | Interleukin-6 | 5.166666667 | 1 | 7 |
| 198138 | JUN | Transcription factor AP-1 | 5.166666667 | 1 | 7 |
| 198213 | IFNG | Interferon gamma | 2.666666667 | 0.875 | 6 |
| 198196 | CCL2 | C-C motif chemokine 2 | 0.5 | 0.777777778 | 5 |
| 198223 | CXCL8 | Interleukin-8 | 0.5 | 0.777777778 | 5 |
| 198267 | STAT1 | Signal transducer and activator of transcription 1-alpha/beta | 0 | 0.7 | 4 |
| 198205 | MMP9 | Matrix metalloproteinase-9 | 0 | 0.7 | 4 |
| 198105 | HSP90AA1 | Heat shock protein HSP 90-alpha | 0 | 0.7 | 4 |

# Supplementary 10

**Drug pair of AM-CPV and intersection GC of core target genes**

**Supplementary table 10. Drug pair of AM-CPV and intersection GC of core target genes.**

| SUID | Gene symbol | Betweenness | Closeness | Degree |
| --- | --- | --- | --- | --- |
| 73 | MMP9 | 44 | 0.75 | 7 |
| 80 | MMP3 | 2 | 0.5625 | 5 |
| 82 | SPP1 | 2 | 0.5625 | 5 |
| 85 | CXCL10 | 2 | 0.529411765 | 4 |
| 79 | COL1A1 | 0 | 0.5 | 3 |
| 91 | PLAU | 0 | 0.5 | 3 |
| 72 | CCNA2 | 28 | 0.5625 | 2 |
| 75 | TYMS | 16 | 0.409090909 | 2 |
| 89 | STAT1 | 0 | 0.473684211 | 2 |
| 77 | CDA | 0 | 0.3 | 1 |

# Supplementary 11

**Molecular function(MF) of GO enrichment analysis**

**Supplementary table 11. Molecular function(MF) of GO enrichment analysis.**

| ID | Description | GeneRatio | pvalue | qvalue | geneID | Count |
| --- | --- | --- | --- | --- | --- | --- |
| GO:0030594 | neurotransmitter receptor activity | 13/149 | 6.01E-12 | 1.79E-09 | CHRM2/GRIA2/DRD1/CHRM4/GABRA3/GABRA5/GABRB1/GABRD/GABRP/GABRQ/GRIN2A/GRIN2D/GRIN3B | 13 |
| GO:0005496 | steroid binding | 12/149 | 3.01E-11 | 4.49E-09 | PGR/AR/CYP3A4/CAV1/NR3C2/SERPINA6/SHBG/NR1H4/SOAT2/ESRRB/GPER1/ESRRG | 12 |
| GO:0005230 | extracellular ligand-gated ion channel activity | 10/149 | 3.71E-10 | 3.69E-08 | GRIA2/GABRA3/GABRA5/GABRB1/GABRD/GABRP/GABRQ/GRIN2A/GRIN2D/GRIN3B | 10 |
| GO:0015276 | ligand-gated ion channel activity | 12/149 | 1.46E-09 | 8.69E-08 | GRIA2/KCNH2/GABRA3/GABRA5/GABRB1/GABRD/GABRP/GABRQ/TRPM8/GRIN2A/GRIN2D/GRIN3B | 12 |
| GO:0022834 | ligand-gated channel activity | 12/149 | 1.46E-09 | 8.69E-08 | GRIA2/KCNH2/GABRA3/GABRA5/GABRB1/GABRD/GABRP/GABRQ/TRPM8/GRIN2A/GRIN2D/GRIN3B | 12 |
| GO:0004890 | GABA-A receptor activity | 6/149 | 6.47E-09 | 3.13E-07 | GABRA3/GABRA5/GABRB1/GABRD/GABRP/GABRQ | 6 |
| GO:0004879 | nuclear receptor activity | 8/149 | 8.75E-09 | 3.13E-07 | PGR/AR/NR1I3/NR3C2/NR1H4/ESRRB/RXRG/ESRRG | 8 |
| GO:0098531 | ligand-activated transcription factor activity | 8/149 | 8.75E-09 | 3.13E-07 | PGR/AR/NR1I3/NR3C2/NR1H4/ESRRB/RXRG/ESRRG | 8 |
| GO:0004497 | monooxygenase activity | 10/149 | 9.46E-09 | 3.13E-07 | CYP3A4/CYP1A1/CYP1B1/AKR1C3/CYP19A1/CYP17A1/CYP2C19/AKR1C1/AKR1C2/CYP27B1 | 10 |
| GO:0032052 | bile acid binding | 5/149 | 1.47E-08 | 4.38E-07 | AKR1C3/NR1H4/AKR1C1/AKR1C2/PLA2G1B | 5 |
| GO:0016917 | GABA receptor activity | 6/149 | 1.74E-08 | 4.72E-07 | GABRA3/GABRA5/GABRB1/GABRD/GABRP/GABRQ | 6 |
| GO:0019825 | oxygen binding | 7/149 | 2.51E-08 | 6.23E-07 | CYP3A4/CYP1A1/CYP1B1/CYP19A1/CYP17A1/NOX4/CYP2C19 | 7 |
| GO:0003707 | steroid hormone receptor activity | 6/149 | 5.24E-08 | 1.20E-06 | PGR/NR3C2/ESRRB/GPER1/RXRG/ESRRG | 6 |
| GO:0015267 | channel activity | 18/149 | 6.57E-08 | 1.35E-06 | GRIA2/KCNH2/BCL2/CLDN4/TRPV3/CACNA1C/CACNA1D/CACNB2/GABRA3/GABRA5/GABRB1/GABRD/GABRP/GABRQ/TRPM8/GRIN2A/GRIN2D/GRIN3B | 18 |
| GO:0022803 | passive transmembrane transporter activity | 18/149 | 6.78E-08 | 1.35E-06 | GRIA2/KCNH2/BCL2/CLDN4/TRPV3/CACNA1C/CACNA1D/CACNB2/GABRA3/GABRA5/GABRB1/GABRD/GABRP/GABRQ/TRPM8/GRIN2A/GRIN2D/GRIN3B | 18 |
| GO:0016705 | oxidoreductase activity, acting on paired donors, with incorporation or reduction of molecular oxygen | 11/149 | 7.74E-08 | 1.36E-06 | PTGS1/CYP3A4/CYP1A1/CYP1B1/AKR1C3/CYP19A1/CYP17A1/CYP2C19/AKR1C1/AKR1C2/CYP27B1 | 11 |
| GO:0005216 | ion channel activity | 17/149 | 7.79E-08 | 1.36E-06 | GRIA2/KCNH2/CLDN4/TRPV3/CACNA1C/CACNA1D/CACNB2/GABRA3/GABRA5/GABRB1/GABRD/GABRP/GABRQ/TRPM8/GRIN2A/GRIN2D/GRIN3B | 17 |
| GO:0022836 | gated channel activity | 15/149 | 1.07E-07 | 1.77E-06 | GRIA2/KCNH2/CACNA1C/CACNA1D/CACNB2/GABRA3/GABRA5/GABRB1/GABRD/GABRP/GABRQ/TRPM8/GRIN2A/GRIN2D/GRIN3B | 15 |
| GO:0016709 | oxidoreductase activity, acting on paired donors, with incorporation or reduction of molecular oxygen, NAD(P)H as one donor, and incorporation of one atom of oxygen | 7/149 | 1.13E-07 | 1.77E-06 | CYP3A4/CYP1A1/AKR1C3/CYP2C19/AKR1C1/AKR1C2/CYP27B1 | 7 |
| GO:0015108 | chloride transmembrane transporter activity | 9/149 | 1.34E-07 | 2.00E-06 | SLC6A3/SLC6A4/CLDN4/GABRA3/GABRA5/GABRB1/GABRD/GABRP/GABRQ | 9 |
| GO:0031406 | carboxylic acid binding | 11/149 | 3.20E-07 | 4.53E-06 | SELE/GSTM2/AKR1C3/TYMS/FOLR2/SLC46A1/NR1H4/AKR1C1/GRIN3B/AKR1C2/PLA2G1B | 11 |
| GO:0022824 | transmitter-gated ion channel activity | 7/149 | 4.86E-07 | 6.30E-06 | GRIA2/GABRA3/GABRA5/GABRB1/GRIN2A/GRIN2D/GRIN3B | 7 |
| GO:0022835 | transmitter-gated channel activity | 7/149 | 4.86E-07 | 6.30E-06 | GRIA2/GABRA3/GABRA5/GABRB1/GRIN2A/GRIN2D/GRIN3B | 7 |
| GO:0008395 | steroid hydroxylase activity | 6/149 | 5.80E-07 | 7.19E-06 | CYP3A4/CYP1A1/CYP1B1/CYP19A1/CYP17A1/CYP2C19 | 6 |
| GO:0015085 | calcium ion transmembrane transporter activity | 9/149 | 1.49E-06 | 1.77E-05 | TRPV3/ATP2A1/CACNA1C/CACNA1D/CACNB2/TRPM8/GRIN2A/GRIN2D/GRIN3B | 9 |
| GO:0070330 | aromatase activity | 5/149 | 1.54E-06 | 1.77E-05 | CYP3A4/CYP1A1/CYP1B1/CYP19A1/CYP2C19 | 5 |
| GO:0046873 | metal ion transmembrane transporter activity | 15/149 | 2.02E-06 | 2.22E-05 | KCNH2/SLC6A3/SLC6A4/TRPV3/ATP2A1/SLC5A2/SLC5A1/SLC28A3/CACNA1C/CACNA1D/CACNB2/TRPM8/GRIN2A/GRIN2D/GRIN3B | 15 |
| GO:0020037 | heme binding | 9/149 | 2.15E-06 | 2.29E-05 | PTGS1/CYP3A4/CYP1A1/CYP1B1/CYP19A1/CYP17A1/NOX4/CYP2C19/CYP27B1 | 9 |
| GO:0048018 | receptor ligand activity | 16/149 | 2.23E-06 | 2.29E-05 | DPP4/F2/IL6/CCL2/CXCL8/IL2/IFNG/IL1A/CXCL11/CXCL10/SPP1/IGF2/FGF1/FGF2/TYMP/NPPB | 16 |
| GO:0005254 | chloride channel activity | 7/149 | 2.32E-06 | 2.30E-05 | CLDN4/GABRA3/GABRA5/GABRB1/GABRD/GABRP/GABRQ | 7 |
| GO:0030546 | signaling receptor activator activity | 16/149 | 2.61E-06 | 2.51E-05 | DPP4/F2/IL6/CCL2/CXCL8/IL2/IFNG/IL1A/CXCL11/CXCL10/SPP1/IGF2/FGF1/FGF2/TYMP/NPPB | 16 |
| GO:1901338 | catecholamine binding | 4/149 | 2.83E-06 | 2.59E-05 | ADRB2/DRD1/SLC6A3/ADRB3 | 4 |
| GO:0015103 | inorganic anion transmembrane transporter activity | 9/149 | 2.87E-06 | 2.59E-05 | SLC6A3/SLC6A4/CLDN4/GABRA3/GABRA5/GABRB1/GABRD/GABRP/GABRQ | 9 |
| GO:0008227 | G protein-coupled amine receptor activity | 6/149 | 3.47E-06 | 3.04E-05 | CHRM2/ADRB2/ADRA1D/CHRM4/ADRA1A/ADRB3 | 6 |
| GO:0046906 | tetrapyrrole binding | 9/149 | 3.80E-06 | 3.23E-05 | PTGS1/CYP3A4/CYP1A1/CYP1B1/CYP19A1/CYP17A1/NOX4/CYP2C19/CYP27B1 | 9 |
| GO:0004089 | carbonate dehydratase activity | 4/149 | 3.93E-06 | 3.26E-05 | CA2/CA1/CA7/CA14 | 4 |
| GO:0008509 | anion transmembrane transporter activity | 15/149 | 5.15E-06 | 4.14E-05 | SLC6A3/SLC6A4/SLC2A4/ABCG2/CLDN4/SLC5A2/SLC5A1/SLC46A1/SLC28A3/GABRA3/GABRA5/GABRB1/GABRD/GABRP/GABRQ | 15 |
| GO:0005262 | calcium channel activity | 8/149 | 5.42E-06 | 4.25E-05 | TRPV3/CACNA1C/CACNA1D/CACNB2/TRPM8/GRIN2A/GRIN2D/GRIN3B | 8 |
| GO:0005253 | anion channel activity | 7/149 | 6.38E-06 | 4.87E-05 | CLDN4/GABRA3/GABRA5/GABRB1/GABRD/GABRP/GABRQ | 7 |
| GO:0016712 | oxidoreductase activity, acting on paired donors, with incorporation or reduction of molecular oxygen, reduced flavin or flavoprotein as one donor, and incorporation of one atom of oxygen | 5/149 | 8.83E-06 | 6.58E-05 | CYP3A4/CYP1A1/CYP1B1/CYP19A1/CYP2C19 | 5 |
| GO:0072341 | modified amino acid binding | 7/149 | 1.07E-05 | 7.80E-05 | DPEP1/GSTM1/GSTM2/TYMS/FOLR2/SLC46A1/NOX4 | 7 |
| GO:0098960 | postsynaptic neurotransmitter receptor activity | 6/149 | 1.10E-05 | 7.80E-05 | CHRM2/DRD1/CHRM4/GABRA3/GABRA5/GABRB1 | 6 |
| GO:0016836 | hydro-lyase activity | 6/149 | 1.32E-05 | 9.17E-05 | CYP1A1/CYP1B1/CA2/CA1/CA7/CA14 | 6 |
| GO:0004970 | ionotropic glutamate receptor activity | 4/149 | 1.48E-05 | 9.99E-05 | GRIA2/GRIN2A/GRIN2D/GRIN3B | 4 |
| GO:0005125 | cytokine activity | 10/149 | 2.28E-05 | 0.000150656 | IL6/CCL2/CXCL8/IL2/IFNG/IL1A/CXCL11/CXCL10/SPP1/FGF2 | 10 |
| GO:0033293 | monocarboxylic acid binding | 6/149 | 2.41E-05 | 0.000156221 | GSTM2/AKR1C3/NR1H4/AKR1C1/AKR1C2/PLA2G1B | 6 |
| GO:0016835 | carbon-oxygen lyase activity | 6/149 | 4.44E-05 | 0.000281343 | CYP1A1/CYP1B1/CA2/CA1/CA7/CA14 | 6 |
| GO:0008331 | high voltage-gated calcium channel activity | 3/149 | 6.05E-05 | 0.000375546 | CACNA1C/CACNA1D/CACNB2 | 3 |
| GO:0008066 | glutamate receptor activity | 4/149 | 6.35E-05 | 0.000386228 | GRIA2/GRIN2A/GRIN2D/GRIN3B | 4 |
| GO:0004032 | alditol:NADP+ 1-oxidoreductase activity | 3/149 | 0.000109621 | 0.000640304 | AKR1C3/AKR1C1/AKR1C2 | 3 |
| GO:0005542 | folic acid binding | 3/149 | 0.000109621 | 0.000640304 | TYMS/FOLR2/SLC46A1 | 3 |
| GO:0008144 | drug binding | 5/149 | 0.000136603 | 0.000781471 | SLC6A3/SLC6A4/NPC1L1/TYMS/FOLR2 | 5 |
| GO:0022843 | voltage-gated cation channel activity | 7/149 | 0.000141642 | 0.000781471 | KCNH2/CACNA1C/CACNA1D/CACNB2/GRIN2A/GRIN2D/GRIN3B | 7 |
| GO:0022851 | GABA-gated chloride ion channel activity | 3/149 | 0.000141659 | 0.000781471 | GABRA3/GABRA5/GABRB1 | 3 |
| GO:0050998 | nitric-oxide synthase binding | 3/149 | 0.00017922 | 0.000970704 | CALM1/SLC6A4/CAV1 | 3 |
| GO:0005506 | iron ion binding | 7/149 | 0.000226732 | 0.001206113 | CYP3A4/CYP1A1/CYP1B1/CYP19A1/CYP17A1/CYP2C19/CYP27B1 | 7 |
| GO:0099094 | ligand-gated cation channel activity | 6/149 | 0.000264902 | 0.001384439 | GRIA2/KCNH2/TRPM8/GRIN2A/GRIN2D/GRIN3B | 6 |
| GO:0035173 | histone kinase activity | 3/149 | 0.000272453 | 0.001399351 | CHEK1/CDK1/PRKCB | 3 |
| GO:1901681 | sulfur compound binding | 9/149 | 0.000280194 | 0.001414716 | F2/GSTM1/GSTM2/CXCL11/CXCL10/FGF1/FGF2/SULT2A1/SOAT2 | 9 |
| GO:0015370 | solute:sodium symporter activity | 5/149 | 0.000299079 | 0.0014849 | SLC6A3/SLC6A4/SLC5A2/SLC5A1/SLC28A3 | 5 |
| GO:0008083 | growth factor activity | 7/149 | 0.000362281 | 0.001769205 | F2/IL6/IL2/IGF2/FGF1/FGF2/TYMP | 7 |
| GO:0005126 | cytokine receptor binding | 9/149 | 0.000369437 | 0.001775052 | STAT1/IL6/CCL2/CXCL8/IL2/IFNG/IL1A/CXCL11/CXCL10 | 9 |
| GO:0004303 | estradiol 17-beta-dehydrogenase activity | 3/149 | 0.000392298 | 0.0017979 | AKR1C3/AKR1C1/AKR1C2 | 3 |
| GO:0045236 | CXCR chemokine receptor binding | 3/149 | 0.000392298 | 0.0017979 | CXCL8/CXCL11/CXCL10 | 3 |
| GO:0099095 | ligand-gated anion channel activity | 3/149 | 0.000392298 | 0.0017979 | GABRA3/GABRA5/GABRB1 | 3 |
| GO:0005261 | cation channel activity | 10/149 | 0.000400838 | 0.001809206 | GRIA2/KCNH2/TRPV3/CACNA1C/CACNA1D/CACNB2/TRPM8/GRIN2A/GRIN2D/GRIN3B | 10 |
| GO:0019207 | kinase regulator activity | 8/149 | 0.000442348 | 0.001945989 | CALM1/CCNA2/PKIA/CDKN1A/IL2/CCNB1/CXCL10/IGF2 | 8 |
| GO:0016616 | oxidoreductase activity, acting on the CH-OH group of donors, NAD or NADP as acceptor | 6/149 | 0.000444208 | 0.001945989 | ADH1B/ADH1C/AKR1C3/AKR1C1/CBR1/AKR1C2 | 6 |
| GO:0004252 | serine-type endopeptidase activity | 7/149 | 0.000450973 | 0.00194699 | DPP4/F2/MMP1/MMP3/PLAU/MMP9/MMP8 | 7 |
| GO:0005326 | neurotransmitter transmembrane transporter activity | 3/149 | 0.000463087 | 0.00197073 | SLC6A3/SLC6A4/GABRQ | 3 |
| GO:0004745 | retinol dehydrogenase activity | 3/149 | 0.000541573 | 0.002272277 | ADH1B/ADH1C/AKR1C3 | 3 |
| GO:0005355 | glucose transmembrane transporter activity | 3/149 | 0.000628085 | 0.00252842 | SLC2A4/SLC5A2/SLC5A1 | 3 |
| GO:0008106 | alcohol dehydrogenase (NADP+) activity | 3/149 | 0.000628085 | 0.00252842 | AKR1C3/AKR1C1/AKR1C2 | 3 |
| GO:0015149 | hexose transmembrane transporter activity | 3/149 | 0.000628085 | 0.00252842 | SLC2A4/SLC5A2/SLC5A1 | 3 |
| GO:0008009 | chemokine activity | 4/149 | 0.000667724 | 0.002652153 | CCL2/CXCL8/CXCL11/CXCL10 | 4 |
| GO:0016614 | oxidoreductase activity, acting on CH-OH group of donors | 6/149 | 0.000678719 | 0.002660352 | ADH1B/ADH1C/AKR1C3/AKR1C1/CBR1/AKR1C2 | 6 |
| GO:0002020 | protease binding | 6/149 | 0.000706604 | 0.002733682 | DPP4/SLC6A3/BCL2/SERPINE1/COL1A1/COL3A1 | 6 |
| GO:0070412 | R-SMAD binding | 3/149 | 0.000722941 | 0.002761028 | JUN/FOS/PARP1 | 3 |
| GO:0008236 | serine-type peptidase activity | 7/149 | 0.000825008 | 0.003073999 | DPP4/F2/MMP1/MMP3/PLAU/MMP9/MMP8 | 7 |
| GO:0015145 | monosaccharide transmembrane transporter activity | 3/149 | 0.00082645 | 0.003073999 | SLC2A4/SLC5A2/SLC5A1 | 3 |
| GO:0019209 | kinase activator activity | 5/149 | 0.000835845 | 0.003073999 | CALM1/CDKN1A/IL2/CCNB1/IGF2 | 5 |
| GO:0017171 | serine hydrolase activity | 7/149 | 0.000878371 | 0.003191 | DPP4/F2/MMP1/MMP3/PLAU/MMP9/MMP8 | 7 |
| GO:0019887 | protein kinase regulator activity | 7/149 | 0.000934423 | 0.00335373 | CALM1/CCNA2/PKIA/CDKN1A/CCNB1/CXCL10/IGF2 | 7 |
| GO:0016829 | lyase activity | 7/149 | 0.001054973 | 0.003717112 | CYP1A1/CYP1B1/CA2/CA1/CA7/CA14/CYP17A1 | 7 |
| GO:0051119 | sugar transmembrane transporter activity | 3/149 | 0.001060625 | 0.003717112 | SLC2A4/SLC5A2/SLC5A1 | 3 |
| GO:0005244 | voltage-gated ion channel activity | 7/149 | 0.001153159 | 0.003944998 | KCNH2/CACNA1C/CACNA1D/CACNB2/GRIN2A/GRIN2D/GRIN3B | 7 |
| GO:0022832 | voltage-gated channel activity | 7/149 | 0.001153159 | 0.003944998 | KCNH2/CACNA1C/CACNA1D/CACNB2/GRIN2A/GRIN2D/GRIN3B | 7 |
| GO:0016628 | oxidoreductase activity, acting on the CH-CH group of donors, NAD or NADP as acceptor | 3/149 | 0.001191863 | 0.003944998 | AKR1C3/AKR1C1/AKR1C2 | 3 |
| GO:0051861 | glycolipid binding | 3/149 | 0.001191863 | 0.003944998 | DPEP1/IL2/HSPA2 | 3 |
| GO:0099604 | ligand-gated calcium channel activity | 3/149 | 0.001191863 | 0.003944998 | TRPM8/GRIN2A/GRIN2D | 3 |
| GO:0015294 | solute:cation symporter activity | 5/149 | 0.001283234 | 0.004200754 | SLC6A3/SLC6A4/SLC5A2/SLC5A1/SLC28A3 | 5 |
| GO:0004033 | aldo-keto reductase (NADP) activity | 3/149 | 0.001332905 | 0.004315929 | AKR1C3/AKR1C1/AKR1C2 | 3 |
| GO:0016655 | oxidoreductase activity, acting on NAD(P)H, quinone or similar compound as acceptor | 4/149 | 0.001434291 | 0.004594275 | AKR1C3/AKR1C1/CBR1/AKR1C2 | 4 |
| GO:0016651 | oxidoreductase activity, acting on NAD(P)H | 5/149 | 0.001465653 | 0.004644791 | AKR1C3/NOX4/AKR1C1/CBR1/AKR1C2 | 5 |
| GO:0030332 | cyclin binding | 3/149 | 0.001817464 | 0.00563972 | CDK1/CDKN1A/CDK6 | 3 |
| GO:0033764 | steroid dehydrogenase activity, acting on the CH-OH group of donors, NAD or NADP as acceptor | 3/149 | 0.001817464 | 0.00563972 | AKR1C3/AKR1C1/AKR1C2 | 3 |
| GO:0043177 | organic acid binding | 5/149 | 0.002390514 | 0.007306034 | TYMS/FOLR2/SLC46A1/NR1H4/GRIN3B | 5 |
| GO:0042379 | chemokine receptor binding | 4/149 | 0.002403505 | 0.007306034 | CCL2/CXCL8/CXCL11/CXCL10 | 4 |
| GO:0016229 | steroid dehydrogenase activity | 3/149 | 0.002615976 | 0.007792855 | AKR1C3/AKR1C1/AKR1C2 | 3 |
| GO:0097718 | disordered domain specific binding | 3/149 | 0.002615976 | 0.007792855 | CALM1/HSP90AA1/HSPA2 | 3 |
| GO:0008603 | cAMP-dependent protein kinase regulator activity | 2/149 | 0.002827801 | 0.008099875 | PKIA/CXCL10 | 2 |
| GO:0018455 | alcohol dehydrogenase [NAD(P)+] activity | 2/149 | 0.002827801 | 0.008099875 | ADH1B/ADH1C | 2 |
| GO:0030283 | testosterone dehydrogenase [NAD(P)] activity | 2/149 | 0.002827801 | 0.008099875 | AKR1C3/AKR1C1 | 2 |
| GO:0099528 | G protein-coupled neurotransmitter receptor activity | 2/149 | 0.002827801 | 0.008099875 | CHRM2/CHRM4 | 2 |
| GO:0015144 | carbohydrate transmembrane transporter activity | 3/149 | 0.003336954 | 0.009467248 | SLC2A4/SLC5A2/SLC5A1 | 3 |
| GO:0008503 | benzodiazepine receptor activity | 2/149 | 0.003437821 | 0.00948249 | GABRA3/GABRA5 | 2 |
| GO:0015378 | sodium:chloride symporter activity | 2/149 | 0.003437821 | 0.00948249 | SLC6A3/SLC6A4 | 2 |
| GO:0048407 | platelet-derived growth factor binding | 2/149 | 0.003437821 | 0.00948249 | COL1A1/COL3A1 | 2 |
| GO:0001216 | DNA-binding transcription activator activity | 10/149 | 0.003608356 | 0.009861561 | PGR/AR/JUN/NR1I3/FOS/E2F1/NR1H4/ESRRB/ESRRG/HOXA10 | 10 |
| GO:0016248 | channel inhibitor activity | 3/149 | 0.003878741 | 0.010409519 | CALM1/BCL2/CAV1 | 3 |
| GO:0043539 | protein serine/threonine kinase activator activity | 3/149 | 0.003878741 | 0.010409519 | CALM1/CCNB1/IGF2 | 3 |
| GO:0044389 | ubiquitin-like protein ligase binding | 8/149 | 0.004035416 | 0.010629562 | KCNH2/JUN/BCL2/STAT1/CDKN1A/CCNB1/CHEK2/HSP90AA1 | 8 |
| GO:0008179 | adenylate cyclase binding | 2/149 | 0.004103462 | 0.010629562 | CALM1/ADRB2 | 2 |
| GO:0008353 | RNA polymerase II CTD heptapeptide repeat kinase activity | 2/149 | 0.004103462 | 0.010629562 | CDK1/CDK6 | 2 |
| GO:0043295 | glutathione binding | 2/149 | 0.004103462 | 0.010629562 | GSTM1/GSTM2 | 2 |
| GO:0033218 | amide binding | 9/149 | 0.00473019 | 0.012147402 | GRIA2/ADRB2/GSTM1/GSTM2/TYMS/FOLR2/SLC46A1/SOAT2/GRIN2A | 9 |
| GO:0072542 | protein phosphatase activator activity | 2/149 | 0.004823789 | 0.012177809 | CALM1/IGFBP3 | 2 |
| GO:1900750 | oligopeptide binding | 2/149 | 0.004823789 | 0.012177809 | GSTM1/GSTM2 | 2 |
| GO:0030295 | protein kinase activator activity | 4/149 | 0.004885138 | 0.012229049 | CALM1/CDKN1A/CCNB1/IGF2 | 4 |
| GO:0005245 | voltage-gated calcium channel activity | 3/149 | 0.005457402 | 0.013435797 | CACNA1C/CACNA1D/CACNB2 | 3 |
| GO:1904315 | transmitter-gated ion channel activity involved in regulation of postsynaptic membrane potential | 3/149 | 0.005457402 | 0.013435797 | GABRA3/GABRA5/GABRB1 | 3 |
| GO:0015373 | anion:sodium symporter activity | 2/149 | 0.005597884 | 0.01366869 | SLC6A3/SLC6A4 | 2 |
| GO:0070851 | growth factor receptor binding | 5/149 | 0.005916099 | 0.014328249 | IL6/IL2/IL1A/FGF1/FGF2 | 5 |
| GO:0005178 | integrin binding | 5/149 | 0.006092761 | 0.014637108 | COL3A1/SPP1/IGF2/FGF1/FGF2 | 5 |
| GO:0015293 | symporter activity | 5/149 | 0.006273105 | 0.01483115 | SLC6A3/SLC6A4/SLC5A2/SLC5A1/SLC28A3 | 5 |
| GO:0052689 | carboxylic ester hydrolase activity | 5/149 | 0.006273105 | 0.01483115 | PON1/CA2/CA1/CES1/PLA2G1B | 5 |
| GO:0005237 | inhibitory extracellular ligand-gated ion channel activity | 2/149 | 0.006424837 | 0.015070276 | GABRA3/GABRA5 | 2 |
| GO:0099529 | neurotransmitter receptor activity involved in regulation of postsynaptic membrane potential | 3/149 | 0.006564483 | 0.015277538 | GABRA3/GABRA5/GABRB1 | 3 |
| GO:0016247 | channel regulator activity | 5/149 | 0.006644996 | 0.015345034 | CALM1/ADRB2/BCL2/CAV1/PRKCB | 5 |
| GO:0015081 | sodium ion transmembrane transporter activity | 5/149 | 0.006836622 | 0.015666105 | SLC6A3/SLC6A4/SLC5A2/SLC5A1/SLC28A3 | 5 |
| GO:0005402 | carbohydrate:cation symporter activity | 2/149 | 0.007303749 | 0.01635901 | SLC5A2/SLC5A1 | 2 |
| GO:0016641 | oxidoreductase activity, acting on the CH-NH2 group of donors, oxygen as acceptor | 2/149 | 0.007303749 | 0.01635901 | MAOB/MAOA | 2 |
| GO:0019211 | phosphatase activator activity | 2/149 | 0.007303749 | 0.01635901 | CALM1/IGFBP3 | 2 |
| GO:1901618 | organic hydroxy compound transmembrane transporter activity | 3/149 | 0.007371405 | 0.016387335 | SLC6A3/SLC6A4/SLC5A1 | 3 |
| GO:0016538 | cyclin-dependent protein serine/threonine kinase regulator activity | 3/149 | 0.007795884 | 0.017202613 | CCNA2/CDKN1A/CCNB1 | 3 |
| GO:0015377 | cation:chloride symporter activity | 2/149 | 0.008233733 | 0.017903545 | SLC6A3/SLC6A4 | 2 |
| GO:0050811 | GABA receptor binding | 2/149 | 0.008233733 | 0.017903545 | GABRA5/GABRB1 | 2 |
| GO:1901505 | carbohydrate derivative transmembrane transporter activity | 3/149 | 0.008687412 | 0.018753148 | SLC5A2/SLC5A1/SLC28A3 | 3 |
| GO:0000979 | RNA polymerase II core promoter sequence-specific DNA binding | 2/149 | 0.009213912 | 0.019746589 | STAT1/FOS | 2 |
| GO:0001228 | DNA-binding transcription activator activity, RNA polymerase II-specific | 9/149 | 0.010340767 | 0.022003285 | PGR/AR/JUN/NR1I3/FOS/NR1H4/ESRRB/ESRRG/HOXA10 | 9 |
| GO:0008201 | heparin binding | 5/149 | 0.010978757 | 0.023195135 | F2/CXCL11/CXCL10/FGF1/FGF2 | 5 |
| GO:0008391 | arachidonic acid monooxygenase activity | 2/149 | 0.011321404 | 0.023432339 | CYP1A1/CYP2C19 | 2 |
| GO:0016638 | oxidoreductase activity, acting on the CH-NH2 group of donors | 2/149 | 0.011321404 | 0.023432339 | MAOB/MAOA | 2 |
| GO:0004222 | metalloendopeptidase activity | 4/149 | 0.011327011 | 0.023432339 | MMP1/MMP3/MMP9/MMP8 | 4 |
| GO:0140297 | DNA-binding transcription factor binding | 8/149 | 0.011857226 | 0.024360036 | AR/JUN/BCL2/STAT1/FOS/PRKCB/PARP1/NR1H4 | 8 |
| GO:0016627 | oxidoreductase activity, acting on the CH-CH group of donors | 3/149 | 0.012264353 | 0.025023879 | AKR1C3/AKR1C1/AKR1C2 | 3 |
| GO:0051427 | hormone receptor binding | 5/149 | 0.013583043 | 0.026868574 | STAT1/PRKCB/PARP1/NR1H4/NPPB | 5 |
| GO:0042826 | histone deacetylase binding | 4/149 | 0.013617574 | 0.026868574 | TOP2A/PARP1/HSP90AA1/HOXA10 | 4 |
| GO:0015296 | anion:cation symporter activity | 2/149 | 0.013619424 | 0.026868574 | SLC6A3/SLC6A4 | 2 |
| GO:0015464 | acetylcholine receptor activity | 2/149 | 0.013619424 | 0.026868574 | CHRM2/CHRM4 | 2 |
| GO:0043027 | cysteine-type endopeptidase inhibitor activity involved in apoptotic process | 2/149 | 0.013619424 | 0.026868574 | DPEP1/BIRC5 | 2 |
| GO:0099106 | ion channel regulator activity | 4/149 | 0.015296968 | 0.029979515 | CALM1/ADRB2/CAV1/PRKCB | 4 |
| GO:0001224 | RNA polymerase II transcription coregulator binding | 2/149 | 0.016101338 | 0.031349698 | PGR/AR | 2 |
| GO:0008237 | metallopeptidase activity | 5/149 | 0.017295166 | 0.033455447 | DPEP1/MMP1/MMP3/MMP9/MMP8 | 5 |
| GO:0005104 | fibroblast growth factor receptor binding | 2/149 | 0.017409226 | 0.033458818 | FGF1/FGF2 | 2 |
| GO:0031072 | heat shock protein binding | 4/149 | 0.018051759 | 0.034471308 | AHSA1/CYP1A1/FGF1/HSPA2 | 4 |
| GO:0001223 | transcription coactivator binding | 2/149 | 0.018760673 | 0.035371555 | PGR/AR | 2 |
| GO:0004364 | glutathione transferase activity | 2/149 | 0.018760673 | 0.035371555 | GSTM1/GSTM2 | 2 |
| GO:0051393 | alpha-actinin binding | 2/149 | 0.020154894 | 0.037761238 | CACNA1C/CACNA1D | 2 |
| GO:0004693 | cyclin-dependent protein serine/threonine kinase activity | 2/149 | 0.023068572 | 0.042419791 | CDK1/CDK6 | 2 |
| GO:0090482 | vitamin transmembrane transporter activity | 2/149 | 0.023068572 | 0.042419791 | ABCG2/SLC46A1 | 2 |
| GO:0097472 | cyclin-dependent protein kinase activity | 2/149 | 0.023068572 | 0.042419791 | CDK1/CDK6 | 2 |
| GO:0061629 | RNA polymerase II-specific DNA-binding transcription factor binding | 6/149 | 0.023489719 | 0.042929224 | JUN/STAT1/FOS/PRKCB/PARP1/NR1H4 | 6 |
| GO:0042910 | xenobiotic transmembrane transporter activity | 2/149 | 0.024586508 | 0.044659704 | ABCG2/SLC46A1 | 2 |
| GO:0046332 | SMAD binding | 3/149 | 0.024840945 | 0.044848405 | JUN/FOS/PARP1 | 3 |
| GO:0019838 | growth factor binding | 4/149 | 0.025620834 | 0.045977781 | ERBB2/COL1A1/COL3A1/IGFBP3 | 4 |
| GO:0051721 | protein phosphatase 2A binding | 2/149 | 0.026144178 | 0.046500319 | SLC6A3/BCL2 | 2 |
| GO:0009055 | electron transfer activity | 4/149 | 0.026224208 | 0.046500319 | MAOB/CYP19A1/NOX4/COX7A1 | 4 |
| GO:0022853 | active ion transmembrane transporter activity | 5/149 | 0.026608802 | 0.04690309 | SLC6A3/SLC6A4/SLC5A2/SLC5A1/SLC28A3 | 5 |
| GO:0035257 | nuclear hormone receptor binding | 4/149 | 0.027456458 | 0.04804565 | STAT1/PRKCB/PARP1/NR1H4 | 4 |
| GO:0019239 | deaminase activity | 2/149 | 0.027740845 | 0.04804565 | ADA/CDA | 2 |

# Supplementary 12

**Cellular composition(CC) of GO enrichment analysis**

**Supplementary table 12. Cellular composition(CC) of GO enrichment analysis.**

| ID | Description | GeneRatio | pvalue | qvalue | geneID | Count |
| --- | --- | --- | --- | --- | --- | --- |
| GO:0034702 | ion channel complex | 18/149 | 1.67E-11 | 1.85E-09 | GRIA2/CALM1/KCNH2/CLDN4/ATP2A1/CACNA1C/CACNA1D/CACNB2/GABRA3/GABRA5/GABRB1/GABRD/GABRP/GABRQ/HSPA2/GRIN2A/GRIN2D/GRIN3B | 18 |
| GO:0045211 | postsynaptic membrane | 17/149 | 2.09E-11 | 1.85E-09 | CHRM2/GRIA2/CHRM4/ADRA1A/SLC6A3/SLC6A4/F2R/CACNA1C/GABRA3/GABRA5/GABRB1/GABRD/GABRP/GABRQ/GRIN2A/GRIN2D/GRIN3B | 17 |
| GO:1902495 | transmembrane transporter complex | 18/149 | 5.34E-11 | 3.15E-09 | GRIA2/CALM1/KCNH2/CLDN4/ATP2A1/CACNA1C/CACNA1D/CACNB2/GABRA3/GABRA5/GABRB1/GABRD/GABRP/GABRQ/HSPA2/GRIN2A/GRIN2D/GRIN3B | 18 |
| GO:1990351 | transporter complex | 18/149 | 1.12E-10 | 4.96E-09 | GRIA2/CALM1/KCNH2/CLDN4/ATP2A1/CACNA1C/CACNA1D/CACNB2/GABRA3/GABRA5/GABRB1/GABRD/GABRP/GABRQ/HSPA2/GRIN2A/GRIN2D/GRIN3B | 18 |
| GO:0097060 | synaptic membrane | 18/149 | 4.44E-10 | 1.57E-08 | CHRM2/GRIA2/CHRM4/ADRA1A/SLC6A3/SLC6A4/F2R/CACNA1C/GABRA3/GABRA5/GABRB1/GABRD/GABRP/GABRQ/GPER1/GRIN2A/GRIN2D/GRIN3B | 18 |
| GO:1902711 | GABA-A receptor complex | 6/149 | 4.47E-09 | 1.32E-07 | GABRA3/GABRA5/GABRB1/GABRD/GABRP/GABRQ | 6 |
| GO:1902710 | GABA receptor complex | 6/149 | 6.34E-09 | 1.60E-07 | GABRA3/GABRA5/GABRB1/GABRD/GABRP/GABRQ | 6 |
| GO:0034707 | chloride channel complex | 7/149 | 9.95E-08 | 2.20E-06 | CLDN4/GABRA3/GABRA5/GABRB1/GABRD/GABRP/GABRQ | 7 |
| GO:0034703 | cation channel complex | 11/149 | 1.12E-06 | 2.21E-05 | GRIA2/CALM1/KCNH2/ATP2A1/CACNA1C/CACNA1D/CACNB2/HSPA2/GRIN2A/GRIN2D/GRIN3B | 11 |
| GO:0031526 | brush border membrane | 6/149 | 5.77E-06 | 0.000102085 | ABCG2/NPC1L1/SLC5A1/SLC46A1/SLC28A3/MTTP | 6 |
| GO:0031253 | cell projection membrane | 12/149 | 1.10E-05 | 0.000177498 | DPP4/DRD1/DPEP1/ABCG2/NPC1L1/SLC5A1/SLC46A1/SLC28A3/GABRA3/GABRA5/MTTP/GPER1 | 12 |
| GO:0005903 | brush border | 7/149 | 1.24E-05 | 0.000180294 | ABCG2/NPC1L1/SLC5A1/SLC46A1/SLC28A3/MTTP/SOAT2 | 7 |
| GO:0034704 | calcium channel complex | 6/149 | 1.33E-05 | 0.000180294 | CALM1/ATP2A1/CACNA1C/CACNA1D/CACNB2/HSPA2 | 6 |
| GO:0000307 | cyclin-dependent protein kinase holoenzyme complex | 5/149 | 1.85E-05 | 0.000233172 | CCNA2/CDK1/CDKN1A/CCNB1/CDK6 | 5 |
| GO:0045121 | membrane raft | 11/149 | 3.90E-05 | 0.000431111 | DPP4/OLR1/ADRA1A/SLC6A3/SLC6A4/SELE/SLC2A4/CAV1/ABCG2/F2R/TRPM8 | 11 |
| GO:0098857 | membrane microdomain | 11/149 | 3.90E-05 | 0.000431111 | DPP4/OLR1/ADRA1A/SLC6A3/SLC6A4/SELE/SLC2A4/CAV1/ABCG2/F2R/TRPM8 | 11 |
| GO:0030315 | T-tubule | 5/149 | 4.29E-05 | 0.000446167 | ADRA1A/SLC2A4/CACNA1C/CACNA1D/CACNB2 | 5 |
| GO:0017146 | NMDA selective glutamate receptor complex | 3/149 | 5.03E-05 | 0.000494083 | GRIN2A/GRIN2D/GRIN3B | 3 |
| GO:0045177 | apical part of cell | 12/149 | 8.20E-05 | 0.000727665 | DPP4/ADRB2/DPEP1/ERBB2/MGAM/ABCG2/CLDN4/NPC1L1/SLC5A1/CA2/SLC46A1/NOX4 | 12 |
| GO:0016324 | apical plasma membrane | 11/149 | 8.23E-05 | 0.000727665 | DPP4/ADRB2/DPEP1/ERBB2/MGAM/ABCG2/CLDN4/NPC1L1/SLC5A1/SLC46A1/NOX4 | 11 |
| GO:1990454 | L-type voltage-gated calcium channel complex | 3/149 | 9.12E-05 | 0.000767751 | CACNA1C/CACNA1D/CACNB2 | 3 |
| GO:0099055 | integral component of postsynaptic membrane | 6/149 | 0.000118163 | 0.000949823 | ADRA1A/SLC6A3/SLC6A4/GABRA3/GABRA5/GRIN2A | 6 |
| GO:0098936 | intrinsic component of postsynaptic membrane | 6/149 | 0.000154624 | 0.001188872 | ADRA1A/SLC6A3/SLC6A4/GABRA3/GABRA5/GRIN2A | 6 |
| GO:0099634 | postsynaptic specialization membrane | 6/149 | 0.000189766 | 0.001398277 | GRIA2/CACNA1C/GABRA3/GABRA5/GRIN2A/GRIN2D | 6 |
| GO:0098862 | cluster of actin-based cell projections | 7/149 | 0.000197705 | 0.0013985 | ABCG2/NPC1L1/SLC5A1/SLC46A1/SLC28A3/MTTP/SOAT2 | 7 |
| GO:0043209 | myelin sheath | 4/149 | 0.000291259 | 0.001981031 | CALM1/BCL2/ERBB2/HSP90AA1 | 4 |
| GO:0005891 | voltage-gated calcium channel complex | 4/149 | 0.000319238 | 0.002090917 | CACNA1C/CACNA1D/CACNB2/HSPA2 | 4 |
| GO:0005901 | caveola | 5/149 | 0.000367599 | 0.002321681 | ADRA1A/SLC6A3/SELE/CAV1/F2R | 5 |
| GO:0099699 | integral component of synaptic membrane | 6/149 | 0.000451495 | 0.00275322 | ADRA1A/SLC6A3/SLC6A4/GABRA3/GABRA5/GRIN2A | 6 |
| GO:0008328 | ionotropic glutamate receptor complex | 4/149 | 0.000488688 | 0.002880684 | GRIA2/GRIN2A/GRIN2D/GRIN3B | 4 |
| GO:0098878 | neurotransmitter receptor complex | 4/149 | 0.000571608 | 0.003181165 | GRIA2/GRIN2A/GRIN2D/GRIN3B | 4 |
| GO:0042383 | sarcolemma | 6/149 | 0.000575639 | 0.003181165 | ADRA1A/SLC2A4/CAV1/CACNA1C/CACNA1D/CACNB2 | 6 |
| GO:1902554 | serine/threonine protein kinase complex | 5/149 | 0.000600804 | 0.00321962 | CCNA2/CDK1/CDKN1A/CCNB1/CDK6 | 5 |
| GO:0042734 | presynaptic membrane | 6/149 | 0.000646897 | 0.003364666 | ADRA1A/SLC6A3/SLC6A4/GABRA5/GPER1/GRIN2A | 6 |
| GO:0099240 | intrinsic component of synaptic membrane | 6/149 | 0.000724812 | 0.003662209 | ADRA1A/SLC6A3/SLC6A4/GABRA3/GABRA5/GRIN2A | 6 |
| GO:0099056 | integral component of presynaptic membrane | 4/149 | 0.000879917 | 0.004322401 | ADRA1A/SLC6A3/SLC6A4/GABRA5 | 4 |
| GO:1902911 | protein kinase complex | 5/149 | 0.001214969 | 0.005806966 | CCNA2/CDK1/CDKN1A/CCNB1/CDK6 | 5 |
| GO:0098793 | presynapse | 11/149 | 0.001307584 | 0.006085155 | CALM1/ADRA1A/SLC6A3/SLC6A4/SLC2A4/PRKCB/CACNA1D/CACNB2/GABRA5/GPER1/GRIN2A | 11 |
| GO:0098889 | intrinsic component of presynaptic membrane | 4/149 | 0.001452115 | 0.006419875 | ADRA1A/SLC6A3/SLC6A4/GABRA5 | 4 |
| GO:0098982 | GABA-ergic synapse | 4/149 | 0.001452115 | 0.006419875 | ADRA1A/GABRA3/GABRA5/GABRB1 | 4 |
| GO:0044853 | plasma membrane raft | 5/149 | 0.001622753 | 0.006904426 | ADRA1A/SLC6A3/SELE/CAV1/F2R | 5 |
| GO:0070820 | tertiary granule | 6/149 | 0.001639801 | 0.006904426 | OLR1/PLAU/MMP9/MGAM/MMP8/CDA | 6 |
| GO:0016529 | sarcoplasmic reticulum | 4/149 | 0.002022868 | 0.008319263 | SLC2A4/GSTM2/HK2/ATP2A1 | 4 |
| GO:0005788 | endoplasmic reticulum lumen | 8/149 | 0.002460979 | 0.009891016 | F2/IL6/COL1A1/COL3A1/SPP1/IGFBP3/MTTP/CES1 | 8 |
| GO:0031094 | platelet dense tubular network | 2/149 | 0.003042604 | 0.011956899 | ATP2A1/F2R | 2 |
| GO:0016528 | sarcoplasm | 4/149 | 0.003144273 | 0.012087823 | SLC2A4/GSTM2/HK2/ATP2A1 | 4 |
| GO:0005583 | fibrillar collagen trimer | 2/149 | 0.00363289 | 0.013384333 | COL1A1/COL3A1 | 2 |
| GO:0098643 | banded collagen fibril | 2/149 | 0.00363289 | 0.013384333 | COL1A1/COL3A1 | 2 |
| GO:0009897 | external side of plasma membrane | 9/149 | 0.003785285 | 0.013661179 | GRIA2/F2/SLC2A4/THBD/ABCG2/CXCL10/ADA/FOLR2/TRPM8 | 9 |
| GO:0005741 | mitochondrial outer membrane | 6/149 | 0.003882221 | 0.013730803 | PGR/MAOB/BCL2/HK2/MAOA/CYP27B1 | 6 |
| GO:0098839 | postsynaptic density membrane | 4/149 | 0.004265392 | 0.014629872 | GRIA2/CACNA1C/GRIN2A/GRIN2D | 4 |
| GO:0032590 | dendrite membrane | 3/149 | 0.004301879 | 0.014629872 | GABRA3/GABRA5/GPER1 | 3 |
| GO:0000793 | condensed chromosome | 6/149 | 0.006488748 | 0.021650638 | CHEK1/BIRC5/CCNB1/TOP2A/ESRRB/HSPA2 | 6 |
| GO:0031968 | organelle outer membrane | 6/149 | 0.00692363 | 0.022673877 | PGR/MAOB/BCL2/HK2/MAOA/CYP27B1 | 6 |
| GO:0019867 | outer membrane | 6/149 | 0.007225058 | 0.023230808 | PGR/MAOB/BCL2/HK2/MAOA/CYP27B1 | 6 |
| GO:1904724 | tertiary granule lumen | 3/149 | 0.008557209 | 0.027022766 | MMP9/MMP8/CDA | 3 |
| GO:0032589 | neuron projection membrane | 3/149 | 0.010372039 | 0.031647585 | GABRA3/GABRA5/GPER1 | 3 |
| GO:0009925 | basal plasma membrane | 6/149 | 0.010379655 | 0.031647585 | MET/ERBB2/CLDN4/CA2/SLC46A1/MTTP | 6 |
| GO:0016327 | apicolateral plasma membrane | 2/149 | 0.011051414 | 0.032572587 | THBD/CLDN4 | 2 |
| GO:0098644 | complex of collagen trimers | 2/149 | 0.011051414 | 0.032572587 | COL1A1/COL3A1 | 2 |
| GO:0000228 | nuclear chromosome | 6/149 | 0.012503122 | 0.036247187 | CHEK1/JUN/BIRC5/CCNB1/TOP2A/HSPA2 | 6 |
| GO:0060205 | cytoplasmic vesicle lumen | 7/149 | 0.012716976 | 0.036272529 | PSMD3/SERPINE1/IGF2/MMP8/ADA/HSP90AA1/CDA | 7 |
| GO:0031983 | vesicle lumen | 7/149 | 0.013114309 | 0.036812096 | PSMD3/SERPINE1/IGF2/MMP8/ADA/HSP90AA1/CDA | 7 |
| GO:0101002 | ficolin-1-rich granule | 5/149 | 0.013854332 | 0.037904931 | PSMD3/MMP9/MGAM/HSP90AA1/CDA | 5 |
| GO:0099060 | integral component of postsynaptic specialization membrane | 3/149 | 0.014047453 | 0.037904931 | GABRA3/GABRA5/GRIN2A | 3 |
| GO:0099572 | postsynaptic specialization | 7/149 | 0.014146662 | 0.037904931 | GRIA2/CACNA1C/GABRA3/GABRA5/GPER1/GRIN2A/GRIN2D | 7 |
| GO:0045178 | basal part of cell | 6/149 | 0.014408952 | 0.038031485 | MET/ERBB2/CLDN4/CA2/SLC46A1/MTTP | 6 |
| GO:1904813 | ficolin-1-rich granule lumen | 4/149 | 0.015101134 | 0.039272298 | PSMD3/MMP9/HSP90AA1/CDA | 4 |
| GO:0098948 | intrinsic component of postsynaptic specialization membrane | 3/149 | 0.015820804 | 0.040547598 | GABRA3/GABRA5/GRIN2A | 3 |
| GO:0031528 | microvillus membrane | 2/149 | 0.01668359 | 0.041925915 | DPEP1/MTTP | 2 |
| GO:0098687 | chromosomal region | 7/149 | 0.016859977 | 0.041925915 | CHEK1/CDK1/BIRC5/CCNB1/TOP2A/PARP1/CHEK2 | 7 |
| GO:0005876 | spindle microtubule | 3/149 | 0.017069837 | 0.041925915 | CALM1/CDK1/BIRC5 | 3 |
| GO:0070821 | tertiary granule membrane | 3/149 | 0.018372548 | 0.044507399 | OLR1/PLAU/MGAM | 3 |

# Supplementary 13

**Biological process(BP) of GO enrichment analysis**

**Supplementary table 13. Biological process(BP) of GO enrichment analysis.**

| ID | Description | GeneRatio | pvalue | qvalue | geneID | Count |
| --- | --- | --- | --- | --- | --- | --- |
| GO:0006816 | calcium ion transport | 26/147 | 1.20E-16 | 2.54E-13 | CALM1/F2/DRD1/ADRA1A/BCL2/GSTM2/CAV1/CCL2/PRKCB/CXCL11/CXCL10/TRPV3/ATP2A1/F2R/FGF2/CACNA1C/CACNA1D/CACNB2/TRPM8/GPER1/HSPA2/GRIN2A/GRIN2D/GRIN3B/PLA2G1B/CYP27B1 | 26 |
| GO:0030001 | metal ion transport | 27/147 | 8.34E-16 | 6.74E-13 | CALM1/F2/DRD1/KCNH2/ADRA1A/BCL2/GSTM2/CAV1/CCL2/PRKCB/CXCL11/CXCL10/TRPV3/ATP2A1/F2R/FGF2/CACNA1C/CACNA1D/CACNB2/TRPM8/GPER1/HSPA2/GRIN2A/GRIN2D/GRIN3B/PLA2G1B/CYP27B1 | 27 |
| GO:0051928 | positive regulation of calcium ion transport | 16/147 | 9.52E-16 | 6.74E-13 | CALM1/F2/DRD1/GSTM2/CAV1/CCL2/CXCL11/CXCL10/TRPV3/ATP2A1/F2R/CACNA1D/CACNB2/GPER1/HSPA2/PLA2G1B | 16 |
| GO:0060402 | calcium ion transport into cytosol | 17/147 | 3.15E-15 | 1.67E-12 | CALM1/F2/DRD1/ADRA1A/BCL2/GSTM2/CAV1/CXCL11/CXCL10/TRPV3/F2R/FGF2/CACNA1C/GPER1/GRIN2A/GRIN2D/PLA2G1B | 17 |
| GO:0010959 | regulation of metal ion transport | 20/147 | 5.47E-15 | 2.32E-12 | CALM1/F2/DRD1/KCNH2/BCL2/GSTM2/CAV1/CCL2/CXCL11/CXCL10/TRPV3/ATP2A1/F2R/CACNA1C/CACNA1D/CACNB2/GPER1/HSPA2/GRIN3B/PLA2G1B | 20 |
| GO:0048545 | response to steroid hormone | 22/147 | 1.24E-14 | 4.08E-12 | PGR/AR/CALM1/MAOB/BCL2/AKR1C3/FOS/CDKN1A/IL6/CAV1/COL1A1/PARP1/CLDN4/SPP1/NR3C2/CA2/TYMS/GABRB1/ESRRB/GPER1/RXRG/ESRRG | 22 |
| GO:0055074 | calcium ion homeostasis | 25/147 | 1.35E-14 | 4.08E-12 | CALM1/F2/ADRA1D/DRD1/ADRA1A/BCL2/GSTM2/CAV1/PTGER3/PRKCB/IL2/CXCL11/CXCL10/TRPV3/ATP2A1/F2R/FGF2/CACNA1C/CACNB2/TRPM8/GPER1/GRIN2A/GRIN2D/PLA2G1B/CYP27B1 | 25 |
| GO:0060401 | cytosolic calcium ion transport | 17/147 | 2.78E-14 | 7.37E-12 | CALM1/F2/DRD1/ADRA1A/BCL2/GSTM2/CAV1/CXCL11/CXCL10/TRPV3/F2R/FGF2/CACNA1C/GPER1/GRIN2A/GRIN2D/PLA2G1B | 17 |
| GO:0051924 | regulation of calcium ion transport | 19/147 | 3.55E-14 | 8.09E-12 | CALM1/F2/DRD1/BCL2/GSTM2/CAV1/CCL2/CXCL11/CXCL10/TRPV3/ATP2A1/F2R/CACNA1C/CACNA1D/CACNB2/GPER1/HSPA2/GRIN3B/PLA2G1B | 19 |
| GO:0007204 | positive regulation of cytosolic calcium ion concentration | 21/147 | 3.81E-14 | 8.09E-12 | CALM1/F2/ADRA1D/DRD1/ADRA1A/BCL2/GSTM2/CAV1/PTGER3/IL2/CXCL11/CXCL10/TRPV3/F2R/FGF2/CACNA1C/CACNB2/GPER1/GRIN2A/GRIN2D/PLA2G1B | 21 |
| GO:0006874 | cellular calcium ion homeostasis | 24/147 | 6.54E-14 | 1.23E-11 | CALM1/F2/ADRA1D/DRD1/ADRA1A/BCL2/GSTM2/CAV1/PTGER3/PRKCB/IL2/CXCL11/CXCL10/TRPV3/ATP2A1/F2R/FGF2/CACNA1C/CACNB2/TRPM8/GPER1/GRIN2A/GRIN2D/PLA2G1B | 24 |
| GO:0042493 | response to drug | 22/147 | 6.97E-14 | 1.23E-11 | MAOB/DRD1/KCNH2/ADRA1A/SLC6A3/SLC6A4/DPEP1/JUN/BCL2/CYP3A4/CYP1A1/FOS/CDKN1A/CCNB1/COL1A1/CHEK2/NPC1L1/ADA/TYMS/SLC46A1/CYP2C19/GRIN2A | 22 |
| GO:0072507 | divalent inorganic cation homeostasis | 25/147 | 1.14E-13 | 1.86E-11 | CALM1/F2/ADRA1D/DRD1/ADRA1A/BCL2/GSTM2/CAV1/PTGER3/PRKCB/IL2/CXCL11/CXCL10/TRPV3/ATP2A1/F2R/FGF2/CACNA1C/CACNB2/TRPM8/GPER1/GRIN2A/GRIN2D/PLA2G1B/CYP27B1 | 25 |
| GO:1904062 | regulation of cation transmembrane transport | 21/147 | 2.32E-13 | 3.52E-11 | GRIA2/CALM1/F2/ADRB2/DRD1/KCNH2/GSTM2/MMP9/CAV1/CCL2/IFNG/CXCL11/CXCL10/ATP2A1/F2R/CACNA1C/CACNA1D/CACNB2/GPER1/HSPA2/GRIN2A | 21 |
| GO:0051480 | regulation of cytosolic calcium ion concentration | 21/147 | 2.92E-13 | 4.03E-11 | CALM1/F2/ADRA1D/DRD1/ADRA1A/BCL2/GSTM2/CAV1/PTGER3/IL2/CXCL11/CXCL10/TRPV3/F2R/FGF2/CACNA1C/CACNB2/GPER1/GRIN2A/GRIN2D/PLA2G1B | 21 |
| GO:1904064 | positive regulation of cation transmembrane transport | 15/147 | 3.04E-13 | 4.03E-11 | CALM1/F2/ADRB2/DRD1/KCNH2/GSTM2/CCL2/IFNG/CXCL11/CXCL10/ATP2A1/F2R/CACNB2/GPER1/HSPA2 | 15 |
| GO:0072503 | cellular divalent inorganic cation homeostasis | 24/147 | 3.90E-13 | 4.87E-11 | CALM1/F2/ADRA1D/DRD1/ADRA1A/BCL2/GSTM2/CAV1/PTGER3/PRKCB/IL2/CXCL11/CXCL10/TRPV3/ATP2A1/F2R/FGF2/CACNA1C/CACNB2/TRPM8/GPER1/GRIN2A/GRIN2D/PLA2G1B | 24 |
| GO:0008202 | steroid metabolic process | 20/147 | 1.21E-12 | 1.43E-10 | CYP3A4/CYP1A1/CYP1B1/AKR1C3/IFNG/SPP1/PON1/CYP19A1/NPC1L1/SERPINA6/CYP17A1/FGF1/CYP2C19/NR1H4/SULT2A1/AKR1C1/SOAT2/AKR1C2/CES1/CYP27B1 | 20 |
| GO:0070588 | calcium ion transmembrane transport | 19/147 | 2.84E-12 | 3.18E-10 | CALM1/F2/DRD1/GSTM2/CXCL11/CXCL10/TRPV3/ATP2A1/F2R/FGF2/CACNA1C/CACNA1D/CACNB2/TRPM8/GPER1/HSPA2/GRIN2A/GRIN2D/GRIN3B | 19 |
| GO:0009636 | response to toxic substance | 17/147 | 5.51E-12 | 5.85E-10 | PTGS1/MAOB/SLC6A4/BCL2/CYP1A1/CYP1B1/GSTM1/GSTM2/FOS/CDKN1A/CCNB1/ABCG2/PON1/TYMS/GABRB1/PIM1/CES1 | 17 |
| GO:1904427 | positive regulation of calcium ion transmembrane transport | 11/147 | 6.44E-12 | 6.29E-10 | CALM1/F2/DRD1/GSTM2/CXCL11/CXCL10/ATP2A1/F2R/CACNB2/GPER1/HSPA2 | 11 |
| GO:0034764 | positive regulation of transmembrane transport | 16/147 | 6.82E-12 | 6.29E-10 | CALM1/F2/ADRB2/DRD1/KCNH2/GSTM2/CCL2/IFNG/CXCL11/CXCL10/ATP2A1/CA2/F2R/CACNB2/GPER1/HSPA2 | 16 |
| GO:0034767 | positive regulation of ion transmembrane transport | 16/147 | 6.82E-12 | 6.29E-10 | CALM1/F2/ADRB2/DRD1/KCNH2/GSTM2/CCL2/IFNG/CXCL11/CXCL10/ATP2A1/CA2/F2R/CACNB2/GPER1/HSPA2 | 16 |
| GO:0010524 | positive regulation of calcium ion transport into cytosol | 10/147 | 8.89E-12 | 7.86E-10 | CALM1/F2/DRD1/GSTM2/CAV1/CXCL11/CXCL10/F2R/GPER1/PLA2G1B | 10 |
| GO:0010522 | regulation of calcium ion transport into cytosol | 12/147 | 1.00E-11 | 8.50E-10 | CALM1/F2/DRD1/BCL2/GSTM2/CAV1/CXCL11/CXCL10/F2R/CACNA1C/GPER1/PLA2G1B | 12 |
| GO:0097553 | calcium ion transmembrane import into cytosol | 13/147 | 4.03E-11 | 3.29E-09 | CALM1/F2/DRD1/GSTM2/CXCL11/CXCL10/TRPV3/F2R/FGF2/CACNA1C/GPER1/GRIN2A/GRIN2D | 13 |
| GO:0006066 | alcohol metabolic process | 19/147 | 9.83E-11 | 7.73E-09 | ADH1B/ADH1C/CALM1/CYP3A4/CYP1A1/CYP1B1/AKR1C3/PON1/NPC1L1/FGF1/FGF2/NR1H4/SULT2A1/AKR1C1/SOAT2/GPER1/AKR1C2/CES1/CYP27B1 | 19 |
| GO:0042391 | regulation of membrane potential | 20/147 | 1.59E-10 | 1.21E-08 | CALM1/ADRB2/KCNH2/ADRA1A/JUN/BCL2/CAV1/PARP1/CACNA1C/CACNA1D/CACNB2/GABRA3/GABRA5/GABRB1/GABRD/GABRP/GABRQ/GPER1/GRIN2A/GRIN2D | 20 |
| GO:0042445 | hormone metabolic process | 15/147 | 1.67E-10 | 1.22E-08 | ADH1B/ADH1C/CYP3A4/CYP1A1/CYP1B1/DIO1/AKR1C3/SPP1/CYP19A1/CYP17A1/SULT2A1/AKR1C1/AKR1C2/CES1/CYP27B1 | 15 |
| GO:0006690 | icosanoid metabolic process | 12/147 | 2.19E-10 | 1.55E-08 | PTGS1/DPEP1/CYP1A1/CYP1B1/GSTM1/AKR1C3/PON1/CYP2C19/AKR1C1/CBR1/AKR1C2/PLA2G1B | 12 |
| GO:0071466 | cellular response to xenobiotic stimulus | 12/147 | 2.64E-10 | 1.81E-08 | PTGS1/DPEP1/CYP3A4/CYP1A1/CYP1B1/GSTM1/GSTM2/E2F1/CYP2C19/SULT2A1/AKR1C1/CES1 | 12 |
| GO:0009410 | response to xenobiotic stimulus | 12/147 | 4.58E-10 | 3.03E-08 | PTGS1/DPEP1/CYP3A4/CYP1A1/CYP1B1/GSTM1/GSTM2/E2F1/CYP2C19/SULT2A1/AKR1C1/CES1 | 12 |
| GO:0051281 | positive regulation of release of sequestered calcium ion into cytosol | 8/147 | 7.00E-10 | 4.50E-08 | CALM1/F2/DRD1/GSTM2/CXCL11/CXCL10/F2R/GPER1 | 8 |
| GO:0032409 | regulation of transporter activity | 16/147 | 7.23E-10 | 4.52E-08 | GRIA2/CALM1/ADRB2/BCL2/GSTM2/MMP9/CAV1/CCL2/IFNG/PON1/ATP2A1/CACNA1D/CACNB2/HSPA2/PIM1/GRIN2A | 16 |
| GO:1903169 | regulation of calcium ion transmembrane transport | 12/147 | 1.48E-09 | 9.00E-08 | CALM1/F2/DRD1/GSTM2/CXCL11/CXCL10/ATP2A1/F2R/CACNA1C/CACNB2/GPER1/HSPA2 | 12 |
| GO:0006805 | xenobiotic metabolic process | 11/147 | 2.48E-09 | 1.46E-07 | PTGS1/DPEP1/CYP3A4/CYP1A1/CYP1B1/GSTM1/GSTM2/CYP2C19/SULT2A1/AKR1C1/CES1 | 11 |
| GO:0022898 | regulation of transmembrane transporter activity | 15/147 | 2.91E-09 | 1.67E-07 | GRIA2/CALM1/ADRB2/BCL2/GSTM2/MMP9/CAV1/CCL2/IFNG/ATP2A1/CACNA1D/CACNB2/HSPA2/PIM1/GRIN2A | 15 |
| GO:0015850 | organic hydroxy compound transport | 15/147 | 3.39E-09 | 1.89E-07 | MAOB/DRD1/SLC6A3/SLC6A4/CAV1/SPP1/PON1/CYP19A1/NPC1L1/SLC5A1/NR1H4/AKR1C1/MTTP/SOAT2/CES1 | 15 |
| GO:2000377 | regulation of reactive oxygen species metabolic process | 13/147 | 3.55E-09 | 1.93E-07 | F2/BCL2/CYP1B1/AKR1C3/MMP3/CDKN1A/CAV1/IFNG/CRP/HK2/MMP8/HSP90AA1/NOX4 | 13 |
| GO:1901654 | response to ketone | 13/147 | 3.78E-09 | 2.00E-07 | AR/CALM1/MAOB/AKR1C3/FOS/CDKN1A/CAV1/PARP1/CLDN4/SPP1/TYMS/GABRB1/AKR1C2 | 13 |
| GO:0051235 | maintenance of location | 16/147 | 4.07E-09 | 2.11E-07 | CALM1/F2/DRD1/GSTM2/IL6/CAV1/CXCL11/CRP/CXCL10/HK2/ATP2A1/F2R/FGF2/CACNA1C/GPER1/CES1 | 16 |
| GO:0035296 | regulation of tube diameter | 11/147 | 4.54E-09 | 2.24E-07 | ADRB2/ADRA1D/DRD1/ADRA1A/SLC6A4/CAV1/CRP/ADRB3/F2R/GPER1/NPPB | 11 |
| GO:0097746 | blood vessel diameter maintenance | 11/147 | 4.54E-09 | 2.24E-07 | ADRB2/ADRA1D/DRD1/ADRA1A/SLC6A4/CAV1/CRP/ADRB3/F2R/GPER1/NPPB | 11 |
| GO:0035150 | regulation of tube size | 11/147 | 4.93E-09 | 2.38E-07 | ADRB2/ADRA1D/DRD1/ADRA1A/SLC6A4/CAV1/CRP/ADRB3/F2R/GPER1/NPPB | 11 |
| GO:0072593 | reactive oxygen species metabolic process | 15/147 | 5.53E-09 | 2.56E-07 | F2/MAOB/BCL2/CYP1A1/CYP1B1/AKR1C3/MMP3/CDKN1A/CAV1/IFNG/CRP/HK2/MMP8/HSP90AA1/NOX4 | 15 |
| GO:0032496 | response to lipopolysaccharide | 16/147 | 5.55E-09 | 2.56E-07 | MAOB/JUN/CYP1A1/SELE/FOS/IL6/CCL2/CXCL8/THBD/SERPINE1/IL1A/CXCL11/CXCL10/F2R/NR1H4/CYP27B1 | 16 |
| GO:0007584 | response to nutrient | 12/147 | 6.49E-09 | 2.93E-07 | SLC6A4/CYP1A1/AKR1C3/COL1A1/CXCL10/SPP1/ADA/TYMS/FOLR2/NR1H4/PIM1/CYP27B1 | 12 |
| GO:0051279 | regulation of release of sequestered calcium ion into cytosol | 9/147 | 7.58E-09 | 3.35E-07 | CALM1/F2/DRD1/GSTM2/CXCL11/CXCL10/F2R/CACNA1C/GPER1 | 9 |
| GO:0003018 | vascular process in circulatory system | 14/147 | 7.73E-09 | 3.35E-07 | ADRB2/ADRA1D/DRD1/ADRA1A/SLC6A4/SLC2A4/CAV1/ABCG2/CRP/ADRB3/SLC5A1/F2R/GPER1/NPPB | 14 |
| GO:0034754 | cellular hormone metabolic process | 11/147 | 9.35E-09 | 3.97E-07 | ADH1B/ADH1C/CYP3A4/CYP1A1/CYP1B1/AKR1C3/SPP1/CYP19A1/CYP17A1/AKR1C1/AKR1C2 | 11 |
| GO:0051651 | maintenance of location in cell | 13/147 | 9.88E-09 | 4.11E-07 | CALM1/F2/DRD1/GSTM2/CAV1/CXCL11/CXCL10/HK2/ATP2A1/F2R/FGF2/CACNA1C/GPER1 | 13 |
| GO:0002237 | response to molecule of bacterial origin | 16/147 | 1.29E-08 | 5.26E-07 | MAOB/JUN/CYP1A1/SELE/FOS/IL6/CCL2/CXCL8/THBD/SERPINE1/IL1A/CXCL11/CXCL10/F2R/NR1H4/CYP27B1 | 16 |
| GO:0010038 | response to metal ion | 16/147 | 1.64E-08 | 6.56E-07 | CALM1/MAOB/SLC6A3/DPEP1/JUN/BCL2/CYP1A1/AKR1C3/FOS/MMP9/CAV1/CCNB1/IL1A/PARP1/CA2/MTTP | 16 |
| GO:0051209 | release of sequestered calcium ion into cytosol | 10/147 | 1.74E-08 | 6.86E-07 | CALM1/F2/DRD1/GSTM2/CXCL11/CXCL10/F2R/FGF2/CACNA1C/GPER1 | 10 |
| GO:0051283 | negative regulation of sequestering of calcium ion | 10/147 | 1.90E-08 | 7.34E-07 | CALM1/F2/DRD1/GSTM2/CXCL11/CXCL10/F2R/FGF2/CACNA1C/GPER1 | 10 |
| GO:0033559 | unsaturated fatty acid metabolic process | 10/147 | 2.25E-08 | 8.27E-07 | PTGS1/CYP1A1/CYP1B1/GSTM1/GSTM2/AKR1C3/CYP2C19/AKR1C1/CBR1/AKR1C2 | 10 |
| GO:0051282 | regulation of sequestering of calcium ion | 10/147 | 2.25E-08 | 8.27E-07 | CALM1/F2/DRD1/GSTM2/CXCL11/CXCL10/F2R/FGF2/CACNA1C/GPER1 | 10 |
| GO:0000302 | response to reactive oxygen species | 13/147 | 2.26E-08 | 8.27E-07 | CCNA2/MET/DPEP1/JUN/BCL2/CYP1B1/AKR1C3/MMP3/FOS/MMP9/IL6/COL1A1/ADA | 13 |
| GO:0031960 | response to corticosteroid | 11/147 | 2.99E-08 | 1.07E-06 | CALM1/MAOB/BCL2/AKR1C3/FOS/CDKN1A/IL6/COL1A1/PARP1/TYMS/GPER1 | 11 |
| GO:0033273 | response to vitamin | 9/147 | 3.10E-08 | 1.07E-06 | CYP1A1/COL1A1/CXCL10/SPP1/ADA/TYMS/FOLR2/PIM1/CYP27B1 | 9 |
| GO:0051208 | sequestering of calcium ion | 10/147 | 3.13E-08 | 1.07E-06 | CALM1/F2/DRD1/GSTM2/CXCL11/CXCL10/F2R/FGF2/CACNA1C/GPER1 | 10 |
| GO:0120254 | olefinic compound metabolic process | 10/147 | 3.13E-08 | 1.07E-06 | PTGS1/CYP1A1/CYP1B1/GSTM2/AKR1C3/CYP17A1/CYP2C19/AKR1C1/CBR1/AKR1C2 | 10 |
| GO:0009991 | response to extracellular stimulus | 18/147 | 3.50E-08 | 1.18E-06 | ADRB2/SLC6A4/JUN/BCL2/CYP1A1/AKR1C3/FOS/CDKN1A/COL1A1/CXCL10/SPP1/PON1/ADA/TYMS/FOLR2/NR1H4/PIM1/CYP27B1 | 18 |
| GO:0097305 | response to alcohol | 13/147 | 3.60E-08 | 1.19E-06 | CALM1/MAOB/SLC6A3/SLC2A4/AKR1C3/FOS/CDKN1A/IL2/PARP1/TYMS/GRIN2A/AKR1C2/CES1 | 13 |
| GO:0034308 | primary alcohol metabolic process | 9/147 | 5.02E-08 | 1.64E-06 | ADH1B/ADH1C/CYP3A4/CYP1A1/CYP1B1/AKR1C3/SULT2A1/AKR1C1/AKR1C2 | 9 |
| GO:0006937 | regulation of muscle contraction | 11/147 | 5.42E-08 | 1.74E-06 | CHRM2/CALM1/ADRB2/ADRA1A/GSTM2/CAV1/ATP2A1/ADA/F2R/CACNA1C/GPER1 | 11 |
| GO:0070482 | response to oxygen levels | 16/147 | 5.70E-08 | 1.80E-06 | DPP4/CCNA2/SLC6A4/BCL2/CYP1A1/PSMD3/SLC2A4/CDKN1A/PLAU/CAV1/CCNB1/COL1A1/E2F1/HK2/ADA/NOX4 | 16 |
| GO:0003015 | heart process | 14/147 | 6.18E-08 | 1.93E-06 | CHRM2/CALM1/ADRA1D/KCNH2/ADRA1A/GSTM2/CAV1/IL2/ATP2A1/ADA/NOX4/CACNA1C/CACNA1D/CACNB2 | 14 |
| GO:1903522 | regulation of blood circulation | 14/147 | 6.45E-08 | 1.97E-06 | CHRM2/CALM1/ADRA1D/KCNH2/ADRA1A/GSTM2/CAV1/IL2/ATP2A1/ADA/F2R/CACNA1C/CACNA1D/CACNB2 | 14 |
| GO:0008016 | regulation of heart contraction | 13/147 | 6.50E-08 | 1.97E-06 | CHRM2/CALM1/ADRA1D/KCNH2/ADRA1A/GSTM2/CAV1/IL2/ATP2A1/ADA/CACNA1C/CACNA1D/CACNB2 | 13 |
| GO:0002027 | regulation of heart rate | 9/147 | 6.62E-08 | 1.98E-06 | CALM1/ADRA1D/KCNH2/ADRA1A/CAV1/ADA/CACNA1C/CACNA1D/CACNB2 | 9 |
| GO:0046683 | response to organophosphorus | 10/147 | 7.29E-08 | 2.15E-06 | SLC6A3/SLC6A4/JUN/STAT1/FOS/THBD/COL1A1/TYMS/NOX4/AKR1C1 | 10 |
| GO:0009314 | response to radiation | 17/147 | 7.71E-08 | 2.24E-06 | CHEK1/CALM1/DRD1/JUN/BCL2/MMP1/MMP3/FOS/CDKN1A/MMP9/THBD/PARP1/COL3A1/CHEK2/CXCL10/NOX4/GRIN2A | 17 |
| GO:0031667 | response to nutrient levels | 17/147 | 8.76E-08 | 2.51E-06 | ADRB2/SLC6A4/JUN/BCL2/CYP1A1/AKR1C3/CDKN1A/COL1A1/CXCL10/SPP1/PON1/ADA/TYMS/FOLR2/NR1H4/PIM1/CYP27B1 | 17 |
| GO:0062197 | cellular response to chemical stress | 15/147 | 9.11E-08 | 2.58E-06 | CCNA2/MET/DPEP1/JUN/BCL2/CYP1B1/SLC2A4/AKR1C3/MMP3/FOS/MMP9/IL6/CAV1/PARP1/NOX4 | 15 |
| GO:0006936 | muscle contraction | 15/147 | 1.10E-07 | 3.06E-06 | CHRM2/CALM1/ADRB2/KCNH2/ADRA1A/GSTM2/CAV1/PTGER3/ATP2A1/ADA/F2R/CACNA1C/CACNA1D/CACNB2/GPER1 | 15 |
| GO:0032412 | regulation of ion transmembrane transporter activity | 13/147 | 1.13E-07 | 3.13E-06 | GRIA2/CALM1/ADRB2/GSTM2/MMP9/CAV1/CCL2/IFNG/ATP2A1/CACNA1D/CACNB2/HSPA2/GRIN2A | 13 |
| GO:0007568 | aging | 14/147 | 1.15E-07 | 3.14E-06 | CHEK1/ADRA1A/SLC6A3/JUN/BCL2/CYP1A1/FOS/CDKN1A/SERPINE1/CHEK2/ADA/TYMS/NOX4/CDK6 | 14 |
| GO:0051385 | response to mineralocorticoid | 6/147 | 1.55E-07 | 4.17E-06 | CALM1/MAOB/FOS/CDKN1A/PARP1/GPER1 | 6 |
| GO:0032411 | positive regulation of transporter activity | 9/147 | 2.30E-07 | 6.11E-06 | CALM1/ADRB2/GSTM2/CCL2/IFNG/PON1/ATP2A1/CACNB2/HSPA2 | 9 |
| GO:0030522 | intracellular receptor signaling pathway | 13/147 | 2.38E-07 | 6.23E-06 | PGR/AR/NR1I3/AKR1C3/PARP1/NR3C2/NR1H4/ESRRB/GPER1/PIM1/RXRG/ESRRG/CYP27B1 | 13 |
| GO:0006367 | transcription initiation from RNA polymerase II promoter | 11/147 | 2.49E-07 | 6.43E-06 | PGR/AR/CDK1/NR1I3/CDKN1A/CCNB1/NR3C2/NR1H4/ESRRB/RXRG/ESRRG | 11 |
| GO:0007188 | adenylate cyclase-modulating G protein-coupled receptor signaling pathway | 12/147 | 2.55E-07 | 6.53E-06 | CHRM2/ADRB2/ADRA1D/DRD1/CHRM4/ADRA1A/PTGER3/CXCL11/CXCL10/ADRB3/CACNA1D/GPER1 | 12 |
| GO:0006694 | steroid biosynthetic process | 11/147 | 2.92E-07 | 7.30E-06 | CYP3A4/CYP1A1/AKR1C3/IFNG/CYP19A1/NPC1L1/CYP17A1/FGF1/NR1H4/CES1/CYP27B1 | 11 |
| GO:0060047 | heart contraction | 13/147 | 2.92E-07 | 7.30E-06 | CHRM2/CALM1/ADRA1D/KCNH2/ADRA1A/GSTM2/CAV1/IL2/ATP2A1/ADA/CACNA1C/CACNA1D/CACNB2 | 13 |
| GO:0006940 | regulation of smooth muscle contraction | 7/147 | 3.68E-07 | 9.08E-06 | CHRM2/ADRB2/ADRA1A/CAV1/ADA/F2R/GPER1 | 7 |
| GO:0045765 | regulation of angiogenesis | 14/147 | 3.76E-07 | 9.18E-06 | STAT1/CYP1B1/IL6/ERBB2/CXCL8/PRKCB/SERPINE1/IL1A/CXCL10/HK2/GLUL/FGF1/FGF2/NPPB | 14 |
| GO:0051899 | membrane depolarization | 8/147 | 3.91E-07 | 9.38E-06 | KCNH2/JUN/BCL2/CAV1/PARP1/CACNA1C/CACNA1D/CACNB2 | 8 |
| GO:0006979 | response to oxidative stress | 16/147 | 3.94E-07 | 9.38E-06 | PTGS1/CCNA2/MET/DPEP1/JUN/BCL2/CYP1B1/AKR1C3/MMP3/FOS/MMP9/IL6/COL1A1/PARP1/ADA/NOX4 | 16 |
| GO:0001676 | long-chain fatty acid metabolic process | 9/147 | 4.16E-07 | 9.81E-06 | PTGS1/CYP3A4/CYP1A1/CYP1B1/GSTM1/GSTM2/AKR1C3/CYP2C19/CBR1 | 9 |
| GO:0006631 | fatty acid metabolic process | 15/147 | 4.36E-07 | 1.02E-05 | PTGS1/CYP3A4/CYP1A1/CYP1B1/GSTM1/GSTM2/AKR1C3/CAV1/PON1/CYP2C19/AKR1C1/CBR1/AKR1C2/CES1/PLA2G1B | 15 |
| GO:0071398 | cellular response to fatty acid | 6/147 | 4.55E-07 | 1.04E-05 | AKR1C3/CCNB1/E2F1/NR1H4/AKR1C1/AKR1C2 | 6 |
| GO:0090257 | regulation of muscle system process | 12/147 | 4.60E-07 | 1.04E-05 | CHRM2/CALM1/ADRB2/ADRA1A/GSTM2/CAV1/PARP1/ATP2A1/ADA/F2R/CACNA1C/GPER1 | 12 |
| GO:0070542 | response to fatty acid | 7/147 | 4.64E-07 | 1.04E-05 | AKR1C3/CCNB1/E2F1/PON1/NR1H4/AKR1C1/AKR1C2 | 7 |
| GO:1901342 | regulation of vasculature development | 14/147 | 4.66E-07 | 1.04E-05 | STAT1/CYP1B1/IL6/ERBB2/CXCL8/PRKCB/SERPINE1/IL1A/CXCL10/HK2/GLUL/FGF1/FGF2/NPPB | 14 |
| GO:0034614 | cellular response to reactive oxygen species | 10/147 | 4.81E-07 | 1.06E-05 | CCNA2/MET/DPEP1/JUN/CYP1B1/AKR1C3/MMP3/FOS/MMP9/IL6 | 10 |
| GO:0003073 | regulation of systemic arterial blood pressure | 8/147 | 5.08E-07 | 1.11E-05 | AR/ADRB2/ADRA1D/ADRA1A/ADRB3/F2R/NPPB/CES1 | 8 |
| GO:0003012 | muscle system process | 16/147 | 5.14E-07 | 1.11E-05 | CHRM2/CALM1/ADRB2/KCNH2/ADRA1A/GSTM2/CAV1/PTGER3/PARP1/ATP2A1/ADA/F2R/CACNA1C/CACNA1D/CACNB2/GPER1 | 16 |
| GO:0016572 | histone phosphorylation | 6/147 | 5.34E-07 | 1.14E-05 | CHEK1/CCNA2/CDK1/PRKCB/CCNB1/RPS6KA5 | 6 |
| GO:0044706 | multi-multicellular organism process | 11/147 | 5.93E-07 | 1.24E-05 | PGR/AR/SLC6A4/BCL2/CYP1A1/FOS/MMP9/THBD/CLDN4/SPP1/CYP27B1 | 11 |
| GO:0001666 | response to hypoxia | 14/147 | 5.95E-07 | 1.24E-05 | DPP4/CCNA2/SLC6A4/BCL2/CYP1A1/PSMD3/SLC2A4/PLAU/CAV1/CCNB1/E2F1/HK2/ADA/NOX4 | 14 |
| GO:0006352 | DNA-templated transcription, initiation | 12/147 | 5.97E-07 | 1.24E-05 | PGR/AR/JUN/CDK1/NR1I3/CDKN1A/CCNB1/NR3C2/NR1H4/ESRRB/RXRG/ESRRG | 12 |
| GO:0034599 | cellular response to oxidative stress | 13/147 | 6.42E-07 | 1.32E-05 | CCNA2/MET/DPEP1/JUN/BCL2/CYP1B1/AKR1C3/MMP3/FOS/MMP9/IL6/PARP1/NOX4 | 13 |
| GO:0016125 | sterol metabolic process | 10/147 | 6.77E-07 | 1.38E-05 | CYP3A4/CYP1B1/PON1/CYP19A1/NPC1L1/FGF1/NR1H4/SULT2A1/SOAT2/CES1 | 10 |
| GO:0001505 | regulation of neurotransmitter levels | 11/147 | 8.29E-07 | 1.67E-05 | CALM1/MAOB/DRD1/ADRA1A/SLC6A3/SLC6A4/PRKCB/MAOA/GLUL/CACNB2/GPER1 | 11 |
| GO:0000086 | G2/M transition of mitotic cell cycle | 12/147 | 8.69E-07 | 1.74E-05 | CHEK1/CALM1/CCNA2/PKIA/CDK1/PSMD3/CDKN1A/CCNB1/CHEK2/HSP90AA1/ESRRB/HSPA2 | 12 |
| GO:0036293 | response to decreased oxygen levels | 14/147 | 8.91E-07 | 1.77E-05 | DPP4/CCNA2/SLC6A4/BCL2/CYP1A1/PSMD3/SLC2A4/PLAU/CAV1/CCNB1/E2F1/HK2/ADA/NOX4 | 14 |
| GO:0023061 | signal release | 16/147 | 9.61E-07 | 1.89E-05 | DPP4/CALM1/MAOB/ADRA1A/SLC6A4/IL6/PRKCB/IFNG/SPP1/CYP19A1/F2R/NR1H4/CACNA1C/CACNA1D/CACNB2/GPER1 | 16 |
| GO:0060048 | cardiac muscle contraction | 9/147 | 1.13E-06 | 2.19E-05 | CALM1/KCNH2/ADRA1A/GSTM2/CAV1/ATP2A1/CACNA1C/CACNA1D/CACNB2 | 9 |
| GO:0006939 | smooth muscle contraction | 8/147 | 1.14E-06 | 2.20E-05 | CHRM2/ADRB2/ADRA1A/CAV1/PTGER3/ADA/F2R/GPER1 | 8 |
| GO:2000379 | positive regulation of reactive oxygen species metabolic process | 8/147 | 1.23E-06 | 2.36E-05 | F2/AKR1C3/CDKN1A/IFNG/CRP/MMP8/HSP90AA1/NOX4 | 8 |
| GO:2001257 | regulation of cation channel activity | 10/147 | 1.29E-06 | 2.44E-05 | GRIA2/CALM1/ADRB2/GSTM2/MMP9/CAV1/CCL2/IFNG/CACNB2/GRIN2A | 10 |
| GO:0007586 | digestion | 9/147 | 1.35E-06 | 2.54E-05 | PTGER3/MGAM/NPC1L1/SLC5A1/SLC46A1/TYMP/AKR1C1/SOAT2/AKR1C2 | 9 |
| GO:0033002 | muscle cell proliferation | 11/147 | 1.37E-06 | 2.54E-05 | JUN/STAT1/CDKN1A/MMP9/IL6/CCNB1/IFNG/IGFBP3/FGF2/GPER1/PIM1 | 11 |
| GO:0032414 | positive regulation of ion transmembrane transporter activity | 8/147 | 1.43E-06 | 2.64E-05 | CALM1/ADRB2/GSTM2/CCL2/IFNG/ATP2A1/CACNB2/HSPA2 | 8 |
| GO:0003044 | regulation of systemic arterial blood pressure mediated by a chemical signal | 6/147 | 1.47E-06 | 2.66E-05 | ADRB2/ADRA1D/ADRA1A/ADRB3/F2R/CES1 | 6 |
| GO:0048146 | positive regulation of fibroblast proliferation | 6/147 | 1.47E-06 | 2.66E-05 | CCNA2/CDKN1A/CCNB1/E2F1/CDK6/PLA2G1B | 6 |
| GO:0071880 | adenylate cyclase-activating adrenergic receptor signaling pathway | 5/147 | 1.55E-06 | 2.78E-05 | ADRB2/ADRA1D/DRD1/ADRA1A/ADRB3 | 5 |
| GO:0009409 | response to cold | 6/147 | 1.67E-06 | 2.95E-05 | ADRB2/FOS/CXCL10/HSP90AA1/TRPM8/HSPA2 | 6 |
| GO:0042572 | retinol metabolic process | 6/147 | 1.67E-06 | 2.95E-05 | ADH1B/ADH1C/CYP3A4/CYP1A1/CYP1B1/AKR1C3 | 6 |
| GO:0044839 | cell cycle G2/M phase transition | 12/147 | 1.76E-06 | 3.09E-05 | CHEK1/CALM1/CCNA2/PKIA/CDK1/PSMD3/CDKN1A/CCNB1/CHEK2/HSP90AA1/ESRRB/HSPA2 | 12 |
| GO:0018958 | phenol-containing compound metabolic process | 8/147 | 1.78E-06 | 3.10E-05 | MAOB/DRD1/SLC6A3/BCL2/DIO1/MAOA/SULT2A1/GRIN2A | 8 |
| GO:0007189 | adenylate cyclase-activating G protein-coupled receptor signaling pathway | 9/147 | 1.82E-06 | 3.14E-05 | ADRB2/ADRA1D/DRD1/ADRA1A/PTGER3/CXCL11/CXCL10/ADRB3/GPER1 | 9 |
| GO:0006692 | prostanoid metabolic process | 6/147 | 1.90E-06 | 3.22E-05 | PTGS1/GSTM1/AKR1C3/AKR1C1/CBR1/AKR1C2 | 6 |
| GO:0006693 | prostaglandin metabolic process | 6/147 | 1.90E-06 | 3.22E-05 | PTGS1/GSTM1/AKR1C3/AKR1C1/CBR1/AKR1C2 | 6 |
| GO:0098657 | import into cell | 11/147 | 1.93E-06 | 3.23E-05 | DRD1/KCNH2/SLC6A3/SLC6A4/IFNG/TRPV3/GLUL/SLC5A2/SLC5A1/FOLR2/SLC46A1 | 11 |
| GO:0014074 | response to purine-containing compound | 9/147 | 1.93E-06 | 3.23E-05 | SLC6A3/SLC6A4/JUN/STAT1/GSTM2/FOS/THBD/COL1A1/NOX4 | 9 |
| GO:0006821 | chloride transport | 8/147 | 2.20E-06 | 3.62E-05 | CLDN4/CA7/GABRA3/GABRA5/GABRB1/GABRD/GABRP/GABRQ | 8 |
| GO:0032963 | collagen metabolic process | 8/147 | 2.20E-06 | 3.62E-05 | F2/MMP1/MMP3/MMP9/IL6/COL1A1/MMP8/F2R | 8 |
| GO:0032102 | negative regulation of response to external stimulus | 14/147 | 2.57E-06 | 4.20E-05 | DPP4/F2/DRD1/SLC6A3/PLAU/CCL2/IL2/THBD/SERPINE1/SPP1/ADA/FGF2/NR1H4/GPER1 | 14 |
| GO:0010332 | response to gamma radiation | 6/147 | 3.43E-06 | 5.56E-05 | BCL2/CDKN1A/PARP1/CHEK2/CXCL10/NOX4 | 6 |
| GO:0071875 | adrenergic receptor signaling pathway | 5/147 | 3.88E-06 | 6.21E-05 | ADRB2/ADRA1D/DRD1/ADRA1A/ADRB3 | 5 |
| GO:0044843 | cell cycle G1/S phase transition | 12/147 | 3.89E-06 | 6.21E-05 | CCNA2/BCL2/CDK1/CYP1A1/CDKN1A/CCL2/CCNB1/CHEK2/E2F1/TYMS/CDK6/ESRRB | 12 |
| GO:1902652 | secondary alcohol metabolic process | 9/147 | 4.15E-06 | 6.58E-05 | CYP3A4/PON1/NPC1L1/FGF1/NR1H4/SULT2A1/SOAT2/CES1/CYP27B1 | 9 |
| GO:2000134 | negative regulation of G1/S transition of mitotic cell cycle | 8/147 | 4.25E-06 | 6.68E-05 | BCL2/CDK1/CDKN1A/CCL2/CCNB1/CHEK2/E2F1/CDK6 | 8 |
| GO:0035637 | multicellular organismal signaling | 10/147 | 4.41E-06 | 6.89E-05 | CALM1/DRD1/KCNH2/CAV1/ATP2A1/TYMP/CACNA1C/CACNA1D/CACNB2/GPER1 | 10 |
| GO:1901617 | organic hydroxy compound biosynthetic process | 11/147 | 4.48E-06 | 6.94E-05 | SLC6A3/CYP3A4/AKR1C3/IFNG/NPC1L1/FGF1/FGF2/NR1H4/GPER1/CES1/CYP27B1 | 11 |
| GO:0042448 | progesterone metabolic process | 4/147 | 4.52E-06 | 6.95E-05 | AKR1C3/CYP17A1/AKR1C1/AKR1C2 | 4 |
| GO:0048660 | regulation of smooth muscle cell proliferation | 9/147 | 4.60E-06 | 7.03E-05 | JUN/STAT1/CDKN1A/MMP9/IL6/IFNG/IGFBP3/FGF2/GPER1 | 9 |
| GO:0019748 | secondary metabolic process | 6/147 | 4.76E-06 | 7.22E-05 | BCL2/CYP1A1/CYP1B1/AKR1C3/AKR1C1/AKR1C2 | 6 |
| GO:1903793 | positive regulation of anion transport | 15/147 | 5.07E-06 | 7.57E-05 | SLC6A4/BCL2/ERBB2/CAV1/IFNG/IL1A/SPP1/E2F1/PON1/CYP19A1/CA2/NR1H4/GPER1/CES1/PLA2G1B | 15 |
| GO:0048659 | smooth muscle cell proliferation | 9/147 | 5.09E-06 | 7.57E-05 | JUN/STAT1/CDKN1A/MMP9/IL6/IFNG/IGFBP3/FGF2/GPER1 | 9 |
| GO:0060326 | cell chemotaxis | 12/147 | 5.10E-06 | 7.57E-05 | DPP4/MET/DPEP1/IL6/CCL2/CXCL8/SERPINE1/CXCL11/CXCL10/FGF1/FGF2/PLA2G1B | 12 |
| GO:0042573 | retinoic acid metabolic process | 5/147 | 5.35E-06 | 7.88E-05 | ADH1B/ADH1C/CYP3A4/CYP1A1/AKR1C3 | 5 |
| GO:1903409 | reactive oxygen species biosynthetic process | 8/147 | 5.44E-06 | 7.96E-05 | MAOB/CYP1A1/CYP1B1/CAV1/IFNG/MMP8/HSP90AA1/NOX4 | 8 |
| GO:1903034 | regulation of response to wounding | 9/147 | 5.63E-06 | 8.18E-05 | F2/PLAU/CAV1/THBD/SERPINE1/CLDN4/SPP1/F2R/FGF2 | 9 |
| GO:0006836 | neurotransmitter transport | 10/147 | 5.71E-06 | 8.23E-05 | CALM1/DRD1/ADRA1A/SLC6A3/SLC6A4/PRKCB/GLUL/CACNB2/GABRQ/GPER1 | 10 |
| GO:0006721 | terpenoid metabolic process | 8/147 | 5.78E-06 | 8.23E-05 | ADH1B/ADH1C/CYP3A4/CYP1A1/CYP1B1/AKR1C3/CYP2C19/AKR1C1 | 8 |
| GO:1902807 | negative regulation of cell cycle G1/S phase transition | 8/147 | 5.78E-06 | 8.23E-05 | BCL2/CDK1/CDKN1A/CCL2/CCNB1/CHEK2/E2F1/CDK6 | 8 |
| GO:0007259 | receptor signaling pathway via JAK-STAT | 9/147 | 6.22E-06 | 8.79E-05 | F2/STAT1/CYP1B1/IL6/CAV1/CCL2/IL2/IFNG/F2R | 9 |
| GO:0019369 | arachidonic acid metabolic process | 6/147 | 6.49E-06 | 9.11E-05 | PTGS1/CYP1A1/CYP1B1/AKR1C3/CYP2C19/CBR1 | 6 |
| GO:0071900 | regulation of protein serine/threonine kinase activity | 15/147 | 7.17E-06 | 0.000100096 | CALM1/CCNA2/ADRB2/PKIA/CDK1/CDKN1A/ERBB2/CAV1/CCNB1/IFNG/CXCL10/IGF2/FGF1/FGF2/PLA2G1B | 15 |
| GO:0071241 | cellular response to inorganic substance | 10/147 | 7.64E-06 | 0.000105949 | CCNA2/DPEP1/JUN/CYP1A1/AKR1C3/MMP3/FOS/MMP9/CCNB1/PARP1 | 10 |
| GO:0003085 | negative regulation of systemic arterial blood pressure | 4/147 | 7.79E-06 | 0.000105949 | ADRB2/ADRA1A/ADRB3/NPPB | 4 |
| GO:0009162 | deoxyribonucleoside monophosphate metabolic process | 4/147 | 7.79E-06 | 0.000105949 | ADA/TYMS/TYMP/TK1 | 4 |
| GO:0090399 | replicative senescence | 4/147 | 7.79E-06 | 0.000105949 | CHEK1/CDKN1A/SERPINE1/CHEK2 | 4 |
| GO:0051591 | response to cAMP | 7/147 | 8.19E-06 | 0.000110033 | SLC6A3/JUN/STAT1/FOS/THBD/COL1A1/NOX4 | 7 |
| GO:0006766 | vitamin metabolic process | 8/147 | 8.19E-06 | 0.000110033 | CYP3A4/CYP1A1/AKR1C3/IFNG/FOLR2/SLC46A1/CBR1/CYP27B1 | 8 |
| GO:0062012 | regulation of small molecule metabolic process | 14/147 | 8.43E-06 | 0.000112491 | PSMD3/AKR1C3/CAV1/IFNG/PARP1/IGFBP3/IGF2/FGF1/CDA/NR1H4/ESRRB/GPER1/CES1/CYP27B1 | 14 |
| GO:0061041 | regulation of wound healing | 8/147 | 8.67E-06 | 0.00011497 | F2/PLAU/CAV1/THBD/SERPINE1/CLDN4/F2R/FGF2 | 8 |
| GO:1901990 | regulation of mitotic cell cycle phase transition | 14/147 | 8.87E-06 | 0.000116977 | CHEK1/PKIA/BCL2/CDK1/CYP1A1/PSMD3/CDKN1A/CCL2/CCNB1/CHEK2/E2F1/HSP90AA1/CDK6/HSPA2 | 14 |
| GO:2000045 | regulation of G1/S transition of mitotic cell cycle | 9/147 | 9.10E-06 | 0.000119201 | BCL2/CDK1/CYP1A1/CDKN1A/CCL2/CCNB1/CHEK2/E2F1/CDK6 | 9 |
| GO:0097696 | receptor signaling pathway via STAT | 9/147 | 9.53E-06 | 0.00012389 | F2/STAT1/CYP1B1/IL6/CAV1/CCL2/IL2/IFNG/F2R | 9 |
| GO:1901021 | positive regulation of calcium ion transmembrane transporter activity | 5/147 | 9.57E-06 | 0.00012389 | CALM1/GSTM2/ATP2A1/CACNB2/HSPA2 | 5 |
| GO:0006941 | striated muscle contraction | 9/147 | 9.98E-06 | 0.000128328 | CALM1/KCNH2/ADRA1A/GSTM2/CAV1/ATP2A1/CACNA1C/CACNA1D/CACNB2 | 9 |
| GO:1902476 | chloride transmembrane transport | 7/147 | 1.01E-05 | 0.000129198 | CLDN4/GABRA3/GABRA5/GABRB1/GABRD/GABRP/GABRQ | 7 |
| GO:0043401 | steroid hormone mediated signaling pathway | 8/147 | 1.02E-05 | 0.000129331 | PGR/AR/PARP1/NR3C2/ESRRB/GPER1/RXRG/ESRRG | 8 |
| GO:0055123 | digestive system development | 8/147 | 1.02E-05 | 0.000129331 | BCL2/CYP1A1/CDKN1A/CXCL8/CCNB1/IGF2/ADA/TYMS | 8 |
| GO:0007565 | female pregnancy | 9/147 | 1.04E-05 | 0.000130354 | PGR/AR/BCL2/FOS/MMP9/THBD/CLDN4/SPP1/CYP27B1 | 9 |
| GO:0008217 | regulation of blood pressure | 9/147 | 1.04E-05 | 0.000130354 | PTGS1/AR/ADRB2/ADRA1D/ADRA1A/ADRB3/F2R/NPPB/CES1 | 9 |
| GO:0072330 | monocarboxylic acid biosynthetic process | 10/147 | 1.05E-05 | 0.000130354 | PTGS1/CYP3A4/CYP1A1/GSTM1/GSTM2/AKR1C3/NR1H4/CBR1/CES1/PLA2G1B | 10 |
| GO:0000082 | G1/S transition of mitotic cell cycle | 11/147 | 1.06E-05 | 0.000131224 | BCL2/CDK1/CYP1A1/CDKN1A/CCL2/CCNB1/CHEK2/E2F1/TYMS/CDK6/ESRRB | 11 |
| GO:0001508 | action potential | 8/147 | 1.08E-05 | 0.000132634 | CALM1/KCNH2/ADRA1A/CAV1/CACNA1C/CACNA1D/CACNB2/GPER1 | 8 |
| GO:0042417 | dopamine metabolic process | 5/147 | 1.10E-05 | 0.000133623 | MAOB/DRD1/SLC6A3/MAOA/GRIN2A | 5 |
| GO:0032768 | regulation of monooxygenase activity | 6/147 | 1.14E-05 | 0.000138748 | CALM1/CAV1/IFNG/IL1A/HSP90AA1/CYP27B1 | 6 |
| GO:0009266 | response to temperature stimulus | 10/147 | 1.23E-05 | 0.000146623 | ADRB2/FOS/CDKN1A/IL1A/CXCL10/TRPV3/FGF1/HSP90AA1/TRPM8/HSPA2 | 10 |
| GO:0022600 | digestive system process | 7/147 | 1.24E-05 | 0.000146623 | PTGER3/NPC1L1/SLC5A1/SLC46A1/TYMP/AKR1C1/SOAT2 | 7 |
| GO:0045933 | positive regulation of muscle contraction | 5/147 | 1.25E-05 | 0.000146623 | ADRA1A/ATP2A1/ADA/F2R/GPER1 | 5 |
| GO:0050892 | intestinal absorption | 5/147 | 1.25E-05 | 0.000146623 | NPC1L1/SLC5A1/SLC46A1/AKR1C1/SOAT2 | 5 |
| GO:0086091 | regulation of heart rate by cardiac conduction | 5/147 | 1.25E-05 | 0.000146623 | KCNH2/CAV1/CACNA1C/CACNA1D/CACNB2 | 5 |
| GO:0030193 | regulation of blood coagulation | 6/147 | 1.25E-05 | 0.000146623 | F2/PLAU/CAV1/THBD/SERPINE1/F2R | 6 |
| GO:0030198 | extracellular matrix organization | 13/147 | 1.28E-05 | 0.000149307 | DPP4/MMP1/CYP1B1/MMP3/MMP9/IL6/CAV1/SERPINE1/COL1A1/COL3A1/SPP1/MMP8/FGF2 | 13 |
| GO:0043062 | extracellular structure organization | 13/147 | 1.32E-05 | 0.000152541 | DPP4/MMP1/CYP1B1/MMP3/MMP9/IL6/CAV1/SERPINE1/COL1A1/COL3A1/SPP1/MMP8/FGF2 | 13 |
| GO:1900046 | regulation of hemostasis | 6/147 | 1.36E-05 | 0.000157409 | F2/PLAU/CAV1/THBD/SERPINE1/F2R | 6 |
| GO:0045229 | external encapsulating structure organization | 13/147 | 1.39E-05 | 0.000159193 | DPP4/MMP1/CYP1B1/MMP3/MMP9/IL6/CAV1/SERPINE1/COL1A1/COL3A1/SPP1/MMP8/FGF2 | 13 |
| GO:0006730 | one-carbon metabolic process | 5/147 | 1.42E-05 | 0.000160962 | CA2/CA1/CA7/CA14/TYMS | 5 |
| GO:1904037 | positive regulation of epithelial cell apoptotic process | 5/147 | 1.42E-05 | 0.000160962 | AKR1C3/IL6/CCL2/GPER1/PLA2G1B | 5 |
| GO:1903039 | positive regulation of leukocyte cell-cell adhesion | 10/147 | 1.54E-05 | 0.000173292 | DPP4/SELE/IL6/CAV1/CCL2/IL2/IFNG/IL1A/IGF2/ADA | 10 |
| GO:0051412 | response to corticosterone | 4/147 | 1.56E-05 | 0.000174812 | CALM1/MAOB/FOS/CDKN1A | 4 |
| GO:0031668 | cellular response to extracellular stimulus | 10/147 | 1.59E-05 | 0.00017745 | JUN/BCL2/AKR1C3/FOS/CDKN1A/COL1A1/FOLR2/NR1H4/PIM1/CYP27B1 | 10 |
| GO:0007200 | phospholipase C-activating G protein-coupled receptor signaling pathway | 7/147 | 1.61E-05 | 0.00017745 | CHRM2/F2/ADRA1D/DRD1/ADRA1A/PTGER3/F2R | 7 |
| GO:0030593 | neutrophil chemotaxis | 7/147 | 1.61E-05 | 0.00017745 | DPP4/DPEP1/CCL2/CXCL8/CXCL11/CXCL10/PLA2G1B | 7 |
| GO:0006720 | isoprenoid metabolic process | 8/147 | 1.65E-05 | 0.000180997 | ADH1B/ADH1C/CYP3A4/CYP1A1/CYP1B1/AKR1C3/CYP2C19/AKR1C1 | 8 |
| GO:0050806 | positive regulation of synaptic transmission | 8/147 | 1.73E-05 | 0.000189414 | DRD1/ADRA1A/CCL2/CA7/CACNB2/GPER1/GRIN2A/GRIN2D | 8 |
| GO:0031670 | cellular response to nutrient | 5/147 | 1.81E-05 | 0.000197069 | COL1A1/FOLR2/NR1H4/PIM1/CYP27B1 | 5 |
| GO:0050818 | regulation of coagulation | 6/147 | 1.91E-05 | 0.000205667 | F2/PLAU/CAV1/THBD/SERPINE1/F2R | 6 |
| GO:2001259 | positive regulation of cation channel activity | 6/147 | 1.91E-05 | 0.000205667 | CALM1/ADRB2/GSTM2/CCL2/IFNG/CACNB2 | 6 |
| GO:0071478 | cellular response to radiation | 9/147 | 1.92E-05 | 0.000205667 | CHEK1/CALM1/MMP1/MMP3/CDKN1A/MMP9/PARP1/CHEK2/NOX4 | 9 |
| GO:0048511 | rhythmic process | 11/147 | 1.98E-05 | 0.000211315 | PGR/SLC6A4/JUN/CDK1/SERPINE1/TOP2A/CLDN4/ADA/TYMS/GABRB1/MTTP | 11 |
| GO:0086003 | cardiac muscle cell contraction | 6/147 | 2.07E-05 | 0.000219591 | KCNH2/CAV1/ATP2A1/CACNA1C/CACNA1D/CACNB2 | 6 |
| GO:0048732 | gland development | 13/147 | 2.16E-05 | 0.000227903 | PGR/AR/SLC6A3/MET/BCL2/CYP1A1/IL6/CAV1/IGF2/HK2/ADA/TYMS/FGF1 | 13 |
| GO:0001523 | retinoid metabolic process | 7/147 | 2.19E-05 | 0.000229796 | ADH1B/ADH1C/CYP3A4/CYP1A1/CYP1B1/AKR1C3/AKR1C1 | 7 |
| GO:0008203 | cholesterol metabolic process | 8/147 | 2.22E-05 | 0.000231766 | CYP3A4/PON1/NPC1L1/FGF1/NR1H4/SULT2A1/SOAT2/CES1 | 8 |
| GO:0072401 | signal transduction involved in DNA integrity checkpoint | 6/147 | 2.24E-05 | 0.000231934 | CHEK1/CDK1/CDKN1A/CCNB1/CHEK2/E2F1 | 6 |
| GO:0072422 | signal transduction involved in DNA damage checkpoint | 6/147 | 2.24E-05 | 0.000231934 | CHEK1/CDK1/CDKN1A/CCNB1/CHEK2/E2F1 | 6 |
| GO:1902806 | regulation of cell cycle G1/S phase transition | 9/147 | 2.26E-05 | 0.000232958 | BCL2/CDK1/CYP1A1/CDKN1A/CCL2/CCNB1/CHEK2/E2F1/CDK6 | 9 |
| GO:0150076 | neuroinflammatory response | 5/147 | 2.28E-05 | 0.000234021 | MMP3/MMP9/IL6/IFNG/MMP8 | 5 |
| GO:1901987 | regulation of cell cycle phase transition | 14/147 | 2.29E-05 | 0.000234021 | CHEK1/PKIA/BCL2/CDK1/CYP1A1/PSMD3/CDKN1A/CCL2/CCNB1/CHEK2/E2F1/HSP90AA1/CDK6/HSPA2 | 14 |
| GO:0042359 | vitamin D metabolic process | 4/147 | 2.32E-05 | 0.000234676 | CYP3A4/CYP1A1/IFNG/CYP27B1 | 4 |
| GO:0055093 | response to hyperoxia | 4/147 | 2.32E-05 | 0.000234676 | CYP1A1/CDKN1A/CAV1/COL1A1 | 4 |
| GO:0051098 | regulation of binding | 12/147 | 2.39E-05 | 0.000240093 | CALM1/ADRB2/MET/JUN/CDKN1A/MMP9/CAV1/IFNG/PARP1/E2F1/PON1/MMP8 | 12 |
| GO:0006575 | cellular modified amino acid metabolic process | 9/147 | 2.55E-05 | 0.000250765 | DPEP1/DIO1/GSTM1/GSTM2/TYMS/FOLR2/SLC46A1/SULT2A1/PLA2G1B | 9 |
| GO:0019722 | calcium-mediated signaling | 9/147 | 2.55E-05 | 0.000250765 | CALM1/SELE/GSTM2/CXCL8/ADA/CACNA1C/TRPM8/GRIN2A/GRIN2D | 9 |
| GO:0006775 | fat-soluble vitamin metabolic process | 5/147 | 2.55E-05 | 0.000250765 | CYP3A4/CYP1A1/IFNG/CBR1/CYP27B1 | 5 |
| GO:0017144 | drug metabolic process | 5/147 | 2.55E-05 | 0.000250765 | DPEP1/CYP3A4/CYP1A1/CYP2C19/CBR1 | 5 |
| GO:0032570 | response to progesterone | 5/147 | 2.55E-05 | 0.000250765 | FOS/CAV1/CLDN4/TYMS/GABRB1 | 5 |
| GO:0071496 | cellular response to external stimulus | 11/147 | 2.62E-05 | 0.000254711 | CHEK1/JUN/BCL2/AKR1C3/FOS/CDKN1A/COL1A1/FOLR2/NR1H4/PIM1/CYP27B1 | 11 |
| GO:0055117 | regulation of cardiac muscle contraction | 6/147 | 2.62E-05 | 0.000254711 | CALM1/ADRA1A/GSTM2/CAV1/ATP2A1/CACNA1C | 6 |
| GO:1901991 | negative regulation of mitotic cell cycle phase transition | 10/147 | 2.71E-05 | 0.000262617 | CHEK1/BCL2/CDK1/PSMD3/CDKN1A/CCL2/CCNB1/CHEK2/E2F1/CDK6 | 10 |
| GO:0001964 | startle response | 4/147 | 2.79E-05 | 0.000267102 | DRD1/SLC6A3/GRIN2A/GRIN2D | 4 |
| GO:0030194 | positive regulation of blood coagulation | 4/147 | 2.79E-05 | 0.000267102 | F2/THBD/SERPINE1/F2R | 4 |
| GO:1900048 | positive regulation of hemostasis | 4/147 | 2.79E-05 | 0.000267102 | F2/THBD/SERPINE1/F2R | 4 |
| GO:0072395 | signal transduction involved in cell cycle checkpoint | 6/147 | 2.82E-05 | 0.000267422 | CHEK1/CDK1/CDKN1A/CCNB1/CHEK2/E2F1 | 6 |
| GO:0086001 | cardiac muscle cell action potential | 6/147 | 2.82E-05 | 0.000267422 | CALM1/KCNH2/CAV1/CACNA1C/CACNA1D/CACNB2 | 6 |
| GO:0006953 | acute-phase response | 5/147 | 2.85E-05 | 0.000268407 | F2/IL6/PTGER3/IL1A/CRP | 5 |
| GO:0071383 | cellular response to steroid hormone stimulus | 9/147 | 2.87E-05 | 0.000269661 | PGR/AR/AKR1C3/PARP1/NR3C2/ESRRB/GPER1/RXRG/ESRRG | 9 |
| GO:1901616 | organic hydroxy compound catabolic process | 6/147 | 3.04E-05 | 0.000282264 | MAOB/SLC6A3/AKR1C3/MAOA/SULT2A1/CYP27B1 | 6 |
| GO:0007159 | leukocyte cell-cell adhesion | 12/147 | 3.05E-05 | 0.000282264 | DPP4/OLR1/SELE/IL6/ERBB2/CAV1/CCL2/IL2/IFNG/IL1A/IGF2/ADA | 12 |
| GO:0050727 | regulation of inflammatory response | 12/147 | 3.05E-05 | 0.000282264 | SELE/MMP3/MMP9/IL6/PTGER3/IL2/SERPINE1/IFNG/MMP8/ADA/NR1H4/GPER1 | 12 |
| GO:0016101 | diterpenoid metabolic process | 7/147 | 3.10E-05 | 0.000286476 | ADH1B/ADH1C/CYP3A4/CYP1A1/CYP1B1/AKR1C3/AKR1C1 | 7 |
| GO:0050673 | epithelial cell proliferation | 13/147 | 3.13E-05 | 0.000287343 | PGR/AR/STAT1/IL6/ERBB2/CAV1/CCL2/IGFBP3/IGF2/GPBAR1/FGF1/FGF2/CDK6 | 13 |
| GO:0007569 | cell aging | 7/147 | 3.28E-05 | 0.000300463 | CHEK1/BCL2/CDKN1A/SERPINE1/CHEK2/NOX4/CDK6 | 7 |
| GO:0050820 | positive regulation of coagulation | 4/147 | 3.33E-05 | 0.000303545 | F2/THBD/SERPINE1/F2R | 4 |
| GO:1904019 | epithelial cell apoptotic process | 7/147 | 3.47E-05 | 0.00031498 | AKR1C3/IL6/CCL2/SERPINE1/E2F1/GPER1/PLA2G1B | 7 |
| GO:0033628 | regulation of cell adhesion mediated by integrin | 5/147 | 3.51E-05 | 0.000315264 | DPP4/CYP1B1/PLAU/SERPINE1/ADA | 5 |
| GO:0042310 | vasoconstriction | 6/147 | 3.52E-05 | 0.000315264 | ADRA1D/ADRA1A/SLC6A4/CAV1/CRP/F2R | 6 |
| GO:0048145 | regulation of fibroblast proliferation | 6/147 | 3.52E-05 | 0.000315264 | CCNA2/CDKN1A/CCNB1/E2F1/CDK6/PLA2G1B | 6 |
| GO:0048144 | fibroblast proliferation | 6/147 | 3.78E-05 | 0.000337233 | CCNA2/CDKN1A/CCNB1/E2F1/CDK6/PLA2G1B | 6 |
| GO:0008637 | apoptotic mitochondrial changes | 7/147 | 3.88E-05 | 0.000342783 | JUN/BCL2/MMP9/E2F1/HK2/ATP2A1/GPER1 | 7 |
| GO:0051101 | regulation of DNA binding | 7/147 | 3.88E-05 | 0.000342783 | CALM1/JUN/MMP9/IFNG/PARP1/E2F1/MMP8 | 7 |
| GO:0042730 | fibrinolysis | 4/147 | 3.94E-05 | 0.000347106 | F2/PLAU/THBD/SERPINE1 | 4 |
| GO:0015711 | organic anion transport | 12/147 | 3.96E-05 | 0.000347106 | ABCG2/CA2/CA1/CA7/CA14/FOLR2/SLC46A1/NR1H4/AKR1C1/MTTP/CES1/PLA2G1B | 12 |
| GO:0031669 | cellular response to nutrient levels | 9/147 | 4.05E-05 | 0.000352995 | JUN/BCL2/AKR1C3/CDKN1A/COL1A1/FOLR2/NR1H4/PIM1/CYP27B1 | 9 |
| GO:0022617 | extracellular matrix disassembly | 6/147 | 4.06E-05 | 0.000352995 | DPP4/MMP1/MMP3/MMP9/IL6/MMP8 | 6 |
| GO:0009416 | response to light stimulus | 11/147 | 4.18E-05 | 0.000359386 | CHEK1/CALM1/DRD1/BCL2/MMP1/MMP3/FOS/CDKN1A/MMP9/PARP1/GRIN2A | 11 |
| GO:0043271 | negative regulation of ion transport | 11/147 | 4.18E-05 | 0.000359386 | CALM1/MAOB/KCNH2/PKIA/BCL2/GSTM2/MMP9/CAV1/PRKCB/ADA/F2R | 11 |
| GO:0048638 | regulation of developmental growth | 11/147 | 4.18E-05 | 0.000359386 | AR/ADRB2/SLC6A3/SLC6A4/BCL2/CDKN1A/CCNB1/SPP1/IGF2/FGF2/PIM1 | 11 |
| GO:0009308 | amine metabolic process | 8/147 | 4.22E-05 | 0.000361098 | MAOB/DRD1/SLC6A3/CYP1A1/PSMD3/MAOA/NR1H4/GRIN2A | 8 |
| GO:0098661 | inorganic anion transmembrane transport | 7/147 | 4.32E-05 | 0.000367995 | CLDN4/GABRA3/GABRA5/GABRB1/GABRD/GABRP/GABRQ | 7 |
| GO:0007623 | circadian rhythm | 9/147 | 4.36E-05 | 0.000368346 | SLC6A4/JUN/CDK1/SERPINE1/TOP2A/CLDN4/ADA/TYMS/MTTP | 9 |
| GO:0050870 | positive regulation of T cell activation | 9/147 | 4.36E-05 | 0.000368346 | DPP4/IL6/CAV1/CCL2/IL2/IFNG/IL1A/IGF2/ADA | 9 |
| GO:0006706 | steroid catabolic process | 4/147 | 4.63E-05 | 0.000390111 | CYP3A4/SPP1/CYP19A1/CYP27B1 | 4 |
| GO:0070509 | calcium ion import | 6/147 | 4.66E-05 | 0.000390914 | CCL2/TRPV3/ATP2A1/CACNA1C/CACNA1D/CACNB2 | 6 |
| GO:0015918 | sterol transport | 7/147 | 4.80E-05 | 0.000399745 | CAV1/PON1/NPC1L1/AKR1C1/MTTP/SOAT2/CES1 | 7 |
| GO:0035265 | organ growth | 8/147 | 4.80E-05 | 0.000399745 | AR/ADRA1A/SLC6A4/BCL2/CCNB1/IGF2/FGF2/PIM1 | 8 |
| GO:1901988 | negative regulation of cell cycle phase transition | 10/147 | 4.88E-05 | 0.000404799 | CHEK1/BCL2/CDK1/PSMD3/CDKN1A/CCL2/CCNB1/CHEK2/E2F1/CDK6 | 10 |
| GO:1990266 | neutrophil migration | 7/147 | 5.06E-05 | 0.000417512 | DPP4/DPEP1/CCL2/CXCL8/CXCL11/CXCL10/PLA2G1B | 7 |
| GO:0006584 | catecholamine metabolic process | 5/147 | 5.20E-05 | 0.000423366 | MAOB/DRD1/SLC6A3/MAOA/GRIN2A | 5 |
| GO:0009712 | catechol-containing compound metabolic process | 5/147 | 5.20E-05 | 0.000423366 | MAOB/DRD1/SLC6A3/MAOA/GRIN2A | 5 |
| GO:0006633 | fatty acid biosynthetic process | 8/147 | 5.23E-05 | 0.000423366 | PTGS1/CYP3A4/CYP1A1/GSTM1/GSTM2/AKR1C3/CBR1/PLA2G1B | 8 |
| GO:0046394 | carboxylic acid biosynthetic process | 11/147 | 5.23E-05 | 0.000423366 | PTGS1/CYP3A4/CYP1A1/GSTM1/GSTM2/AKR1C3/GLUL/NR1H4/CBR1/CES1/PLA2G1B | 11 |
| GO:0071621 | granulocyte chemotaxis | 7/147 | 5.32E-05 | 0.000423366 | DPP4/DPEP1/CCL2/CXCL8/CXCL11/CXCL10/PLA2G1B | 7 |
| GO:0019614 | catechol-containing compound catabolic process | 3/147 | 5.35E-05 | 0.000423366 | MAOB/SLC6A3/MAOA | 3 |
| GO:0030647 | aminoglycoside antibiotic metabolic process | 3/147 | 5.35E-05 | 0.000423366 | AKR1C3/AKR1C1/AKR1C2 | 3 |
| GO:0030656 | regulation of vitamin metabolic process | 3/147 | 5.35E-05 | 0.000423366 | AKR1C3/IFNG/CYP27B1 | 3 |
| GO:0042368 | vitamin D biosynthetic process | 3/147 | 5.35E-05 | 0.000423366 | CYP3A4/IFNG/CYP27B1 | 3 |
| GO:0042424 | catecholamine catabolic process | 3/147 | 5.35E-05 | 0.000423366 | MAOB/SLC6A3/MAOA | 3 |
| GO:0051918 | negative regulation of fibrinolysis | 3/147 | 5.35E-05 | 0.000423366 | F2/THBD/SERPINE1 | 3 |
| GO:0035235 | ionotropic glutamate receptor signaling pathway | 4/147 | 5.41E-05 | 0.000423649 | GRIA2/GRIN2A/GRIN2D/GRIN3B | 4 |
| GO:0045987 | positive regulation of smooth muscle contraction | 4/147 | 5.41E-05 | 0.000423649 | ADRA1A/ADA/F2R/GPER1 | 4 |
| GO:0097529 | myeloid leukocyte migration | 9/147 | 5.41E-05 | 0.000423649 | DPP4/DPEP1/IL6/CCL2/CXCL8/SERPINE1/CXCL11/CXCL10/PLA2G1B | 9 |
| GO:0048565 | digestive tract development | 7/147 | 5.60E-05 | 0.000436829 | BCL2/CYP1A1/CDKN1A/CXCL8/CCNB1/ADA/TYMS | 7 |
| GO:0071214 | cellular response to abiotic stimulus | 11/147 | 5.68E-05 | 0.000436829 | CHEK1/CALM1/MMP1/SLC2A4/MMP3/CDKN1A/MMP9/COL1A1/PARP1/CHEK2/NOX4 | 11 |
| GO:0104004 | cellular response to environmental stimulus | 11/147 | 5.68E-05 | 0.000436829 | CHEK1/CALM1/MMP1/SLC2A4/MMP3/CDKN1A/MMP9/COL1A1/PARP1/CHEK2/NOX4 | 11 |
| GO:1903037 | regulation of leukocyte cell-cell adhesion | 11/147 | 5.68E-05 | 0.000436829 | DPP4/SELE/IL6/ERBB2/CAV1/CCL2/IL2/IFNG/IL1A/IGF2/ADA | 11 |
| GO:0051099 | positive regulation of binding | 8/147 | 5.68E-05 | 0.000436829 | CALM1/MET/MMP9/CAV1/IFNG/PARP1/PON1/MMP8 | 8 |
| GO:0043491 | protein kinase B signaling | 10/147 | 5.70E-05 | 0.000436829 | MET/AKR1C3/ERBB2/CCL2/IGF2/FGF1/FGF2/HSP90AA1/GPER1/AKR1C2 | 10 |
| GO:0015698 | inorganic anion transport | 8/147 | 5.93E-05 | 0.000452286 | CLDN4/CA7/GABRA3/GABRA5/GABRB1/GABRD/GABRP/GABRQ | 8 |
| GO:0018209 | peptidyl-serine modification | 11/147 | 6.16E-05 | 0.000468596 | BCL2/CDK1/IL6/CAV1/PRKCB/CCNB1/IFNG/PARP1/CHEK2/RPS6KA5/HSP90AA1 | 11 |
| GO:0086002 | cardiac muscle cell action potential involved in contraction | 5/147 | 6.25E-05 | 0.000471696 | KCNH2/CAV1/CACNA1C/CACNA1D/CACNB2 | 5 |
| GO:0022409 | positive regulation of cell-cell adhesion | 10/147 | 6.25E-05 | 0.000471696 | DPP4/SELE/IL6/CAV1/CCL2/IL2/IFNG/IL1A/IGF2/ADA | 10 |
| GO:0010039 | response to iron ion | 4/147 | 6.27E-05 | 0.000471696 | SLC6A3/BCL2/CYP1A1/CCNB1 | 4 |
| GO:0045787 | positive regulation of cell cycle | 12/147 | 6.36E-05 | 0.000476857 | CHEK1/SLC6A4/CDK1/CYP1A1/CDKN1A/CCNB1/IL1A/CHEK2/E2F1/IGF2/GPER1/HSPA2 | 12 |
| GO:0032944 | regulation of mononuclear cell proliferation | 9/147 | 6.45E-05 | 0.000481588 | BCL2/CDKN1A/IL6/ERBB2/IL2/IL1A/CRP/IGF2/ADA | 9 |
| GO:0034644 | cellular response to UV | 6/147 | 6.48E-05 | 0.000482314 | CHEK1/MMP1/MMP3/CDKN1A/MMP9/PARP1 | 6 |
| GO:0016053 | organic acid biosynthetic process | 11/147 | 6.50E-05 | 0.000482314 | PTGS1/CYP3A4/CYP1A1/GSTM1/GSTM2/AKR1C3/GLUL/NR1H4/CBR1/CES1/PLA2G1B | 11 |
| GO:0050920 | regulation of chemotaxis | 9/147 | 6.67E-05 | 0.000493252 | DPP4/MET/IL6/CCL2/CXCL8/SERPINE1/CXCL10/FGF1/FGF2 | 9 |
| GO:0050708 | regulation of protein secretion | 10/147 | 6.84E-05 | 0.000502592 | DPP4/IL6/IFNG/IL1A/F2R/NR1H4/CACNA1C/CACNA1D/GPER1/PLA2G1B | 10 |
| GO:1901657 | glycosyl compound metabolic process | 7/147 | 6.84E-05 | 0.000502592 | AKR1C3/ADA/TYMP/CDA/TK1/AKR1C1/AKR1C2 | 7 |
| GO:0045766 | positive regulation of angiogenesis | 8/147 | 6.97E-05 | 0.000504965 | CYP1B1/CXCL8/PRKCB/SERPINE1/IL1A/HK2/FGF1/FGF2 | 8 |
| GO:0048771 | tissue remodeling | 8/147 | 6.97E-05 | 0.000504965 | ADRB2/IL6/CAV1/IL2/IL1A/SPP1/CA2/F2R | 8 |
| GO:0051897 | positive regulation of protein kinase B signaling | 8/147 | 6.97E-05 | 0.000504965 | MET/AKR1C3/ERBB2/IGF2/FGF1/FGF2/HSP90AA1/AKR1C2 | 8 |
| GO:1904018 | positive regulation of vasculature development | 8/147 | 6.97E-05 | 0.000504965 | CYP1B1/CXCL8/PRKCB/SERPINE1/IL1A/HK2/FGF1/FGF2 | 8 |
| GO:0030595 | leukocyte chemotaxis | 9/147 | 7.14E-05 | 0.000515554 | DPP4/DPEP1/IL6/CCL2/CXCL8/SERPINE1/CXCL11/CXCL10/PLA2G1B | 9 |
| GO:0036296 | response to increased oxygen levels | 4/147 | 7.23E-05 | 0.000516607 | CYP1A1/CDKN1A/CAV1/COL1A1 | 4 |
| GO:0042133 | neurotransmitter metabolic process | 4/147 | 7.23E-05 | 0.000516607 | MAOB/SLC6A3/SLC6A4/MAOA | 4 |
| GO:0008655 | pyrimidine-containing compound salvage | 3/147 | 7.31E-05 | 0.000516607 | TYMP/CDA/TK1 | 3 |
| GO:0019371 | cyclooxygenase pathway | 3/147 | 7.31E-05 | 0.000516607 | PTGS1/AKR1C3/CBR1 | 3 |
| GO:0043097 | pyrimidine nucleoside salvage | 3/147 | 7.31E-05 | 0.000516607 | TYMP/CDA/TK1 | 3 |
| GO:0071492 | cellular response to UV-A | 3/147 | 7.31E-05 | 0.000516607 | MMP1/MMP3/MMP9 | 3 |
| GO:0002696 | positive regulation of leukocyte activation | 12/147 | 7.34E-05 | 0.000516607 | DPP4/BCL2/CDKN1A/IL6/CAV1/CCL2/IL2/IFNG/IL1A/IGF2/MMP8/ADA | 12 |
| GO:1903035 | negative regulation of response to wounding | 6/147 | 7.35E-05 | 0.000516607 | F2/PLAU/THBD/SERPINE1/SPP1/FGF2 | 6 |
| GO:0006977 | DNA damage response, signal transduction by p53 class mediator resulting in cell cycle arrest | 5/147 | 7.45E-05 | 0.000520022 | CDK1/CDKN1A/CCNB1/CHEK2/E2F1 | 5 |
| GO:0043388 | positive regulation of DNA binding | 5/147 | 7.45E-05 | 0.000520022 | CALM1/MMP9/IFNG/PARP1/MMP8 | 5 |
| GO:0019216 | regulation of lipid metabolic process | 12/147 | 7.52E-05 | 0.000523047 | F2/CYP1A1/AKR1C3/CAV1/IFNG/FGF1/FGF2/NR1H4/SULT2A1/GPER1/CES1/CYP27B1 | 12 |
| GO:0006942 | regulation of striated muscle contraction | 6/147 | 7.82E-05 | 0.000540606 | CALM1/ADRA1A/GSTM2/CAV1/ATP2A1/CACNA1C | 6 |
| GO:0050810 | regulation of steroid biosynthetic process | 6/147 | 7.82E-05 | 0.000540606 | AKR1C3/IFNG/FGF1/NR1H4/CES1/CYP27B1 | 6 |
| GO:0050804 | modulation of chemical synaptic transmission | 12/147 | 8.07E-05 | 0.000553723 | CALM1/ADRB2/DRD1/ADRA1A/SLC6A4/CCL2/PRKCB/CA7/CACNB2/GPER1/GRIN2A/GRIN2D | 12 |
| GO:0072431 | signal transduction involved in mitotic G1 DNA damage checkpoint | 5/147 | 8.11E-05 | 0.000553723 | CDK1/CDKN1A/CCNB1/CHEK2/E2F1 | 5 |
| GO:0086065 | cell communication involved in cardiac conduction | 5/147 | 8.11E-05 | 0.000553723 | CALM1/CAV1/CACNA1C/CACNA1D/CACNB2 | 5 |
| GO:1902400 | intracellular signal transduction involved in G1 DNA damage checkpoint | 5/147 | 8.11E-05 | 0.000553723 | CDK1/CDKN1A/CCNB1/CHEK2/E2F1 | 5 |
| GO:0099177 | regulation of trans-synaptic signaling | 12/147 | 8.26E-05 | 0.000560184 | CALM1/ADRB2/DRD1/ADRA1A/SLC6A4/CCL2/PRKCB/CA7/CACNB2/GPER1/GRIN2A/GRIN2D | 12 |
| GO:0008209 | androgen metabolic process | 4/147 | 8.29E-05 | 0.000560184 | CYP3A4/SPP1/CYP19A1/CYP17A1 | 4 |
| GO:0071295 | cellular response to vitamin | 4/147 | 8.29E-05 | 0.000560184 | COL1A1/FOLR2/PIM1/CYP27B1 | 4 |
| GO:0071482 | cellular response to light stimulus | 7/147 | 8.70E-05 | 0.000586271 | CHEK1/CALM1/MMP1/MMP3/CDKN1A/MMP9/PARP1 | 7 |
| GO:0042542 | response to hydrogen peroxide | 7/147 | 9.12E-05 | 0.000608553 | MET/JUN/BCL2/CYP1B1/IL6/COL1A1/ADA | 7 |
| GO:0050671 | positive regulation of lymphocyte proliferation | 7/147 | 9.12E-05 | 0.000608553 | BCL2/CDKN1A/IL6/IL2/IL1A/IGF2/ADA | 7 |
| GO:0051384 | response to glucocorticoid | 7/147 | 9.12E-05 | 0.000608553 | CALM1/MAOB/BCL2/FOS/CDKN1A/IL6/TYMS | 7 |
| GO:0042632 | cholesterol homeostasis | 6/147 | 9.37E-05 | 0.000621527 | CAV1/NR1H4/AKR1C1/MTTP/SOAT2/CES1 | 6 |
| GO:1904035 | regulation of epithelial cell apoptotic process | 6/147 | 9.37E-05 | 0.000621527 | AKR1C3/IL6/CCL2/SERPINE1/GPER1/PLA2G1B | 6 |
| GO:0045907 | positive regulation of vasoconstriction | 4/147 | 9.46E-05 | 0.000623762 | ADRA1D/ADRA1A/CAV1/F2R | 4 |
| GO:0050867 | positive regulation of cell activation | 12/147 | 9.49E-05 | 0.000623762 | DPP4/BCL2/CDKN1A/IL6/CAV1/CCL2/IL2/IFNG/IL1A/IGF2/MMP8/ADA | 12 |
| GO:0032946 | positive regulation of mononuclear cell proliferation | 7/147 | 9.55E-05 | 0.000623762 | BCL2/CDKN1A/IL6/IL2/IL1A/IGF2/ADA | 7 |
| GO:0090303 | positive regulation of wound healing | 5/147 | 9.58E-05 | 0.000623762 | F2/THBD/SERPINE1/CLDN4/F2R | 5 |
| GO:1902402 | signal transduction involved in mitotic DNA damage checkpoint | 5/147 | 9.58E-05 | 0.000623762 | CDK1/CDKN1A/CCNB1/CHEK2/E2F1 | 5 |
| GO:1902403 | signal transduction involved in mitotic DNA integrity checkpoint | 5/147 | 9.58E-05 | 0.000623762 | CDK1/CDKN1A/CCNB1/CHEK2/E2F1 | 5 |
| GO:0007050 | cell cycle arrest | 9/147 | 9.63E-05 | 0.000624818 | CDK1/CDKN1A/CXCL8/CCNB1/IFNG/CHEK2/E2F1/CDK6/GPER1 | 9 |
| GO:0007077 | mitotic nuclear envelope disassembly | 3/147 | 9.69E-05 | 0.000625058 | CDK1/PRKCB/CCNB1 | 3 |
| GO:0042178 | xenobiotic catabolic process | 3/147 | 9.69E-05 | 0.000625058 | CYP1A1/GSTM1/GSTM2 | 3 |
| GO:0055092 | sterol homeostasis | 6/147 | 9.94E-05 | 0.000639235 | CAV1/NR1H4/AKR1C1/MTTP/SOAT2/CES1 | 6 |
| GO:0051047 | positive regulation of secretion | 10/147 | 0.000108401 | 0.000694977 | SLC6A4/IFNG/IL1A/SPP1/CYP19A1/NR1H4/CACNB2/GPER1/NPPB/PLA2G1B | 10 |
| GO:0051251 | positive regulation of lymphocyte activation | 11/147 | 0.000111508 | 0.000712747 | DPP4/BCL2/CDKN1A/IL6/CAV1/CCL2/IL2/IFNG/IL1A/IGF2/ADA | 11 |
| GO:0072413 | signal transduction involved in mitotic cell cycle checkpoint | 5/147 | 0.000112454 | 0.000716634 | CDK1/CDKN1A/CCNB1/CHEK2/E2F1 | 5 |
| GO:0070663 | regulation of leukocyte proliferation | 9/147 | 0.000116591 | 0.000740773 | BCL2/CDKN1A/IL6/ERBB2/IL2/IL1A/CRP/IGF2/ADA | 9 |
| GO:0071248 | cellular response to metal ion | 8/147 | 0.000119228 | 0.000755268 | DPEP1/JUN/CYP1A1/AKR1C3/FOS/MMP9/CCNB1/PARP1 | 8 |
| GO:0062013 | positive regulation of small molecule metabolic process | 7/147 | 0.000119753 | 0.00075593 | IFNG/IGF2/FGF1/NR1H4/ESRRB/GPER1/CES1 | 7 |
| GO:0010948 | negative regulation of cell cycle process | 11/147 | 0.000120045 | 0.00075593 | CHEK1/BCL2/CDK1/PSMD3/CDKN1A/CCL2/CCNB1/CHEK2/E2F1/CDK6/GPER1 | 11 |
| GO:0042759 | long-chain fatty acid biosynthetic process | 4/147 | 0.000121539 | 0.000760933 | CYP3A4/CYP1A1/GSTM1/GSTM2 | 4 |
| GO:0031571 | mitotic G1 DNA damage checkpoint | 5/147 | 0.000121557 | 0.000760933 | CDK1/CDKN1A/CCNB1/CHEK2/E2F1 | 5 |
| GO:0051588 | regulation of neurotransmitter transport | 6/147 | 0.000124935 | 0.00077496 | CALM1/DRD1/ADRA1A/PRKCB/CACNB2/GPER1 | 6 |
| GO:0010212 | response to ionizing radiation | 7/147 | 0.000125152 | 0.00077496 | BCL2/CDKN1A/THBD/PARP1/CHEK2/CXCL10/NOX4 | 7 |
| GO:0019336 | phenol-containing compound catabolic process | 3/147 | 0.000125258 | 0.00077496 | MAOB/SLC6A3/MAOA | 3 |
| GO:0042362 | fat-soluble vitamin biosynthetic process | 3/147 | 0.000125258 | 0.00077496 | CYP3A4/IFNG/CYP27B1 | 3 |
| GO:0045785 | positive regulation of cell adhesion | 12/147 | 0.000126968 | 0.000783257 | DPP4/SELE/IL6/ERBB2/CAV1/CCL2/IL2/IFNG/IL1A/IGF2/ADA/CDK6 | 12 |
| GO:0044783 | G1 DNA damage checkpoint | 5/147 | 0.000131214 | 0.000804769 | CDK1/CDKN1A/CCNB1/CHEK2/E2F1 | 5 |
| GO:0044819 | mitotic G1/S transition checkpoint | 5/147 | 0.000131214 | 0.000804769 | CDK1/CDKN1A/CCNB1/CHEK2/E2F1 | 5 |
| GO:0008277 | regulation of G protein-coupled receptor signaling pathway | 7/147 | 0.000136542 | 0.000827768 | CALM1/F2/ADRB2/MET/CXCL8/ADA/GPER1 | 7 |
| GO:0009112 | nucleobase metabolic process | 4/147 | 0.000136914 | 0.000827768 | ADA/TYMS/TYMP/CDA | 4 |
| GO:0033280 | response to vitamin D | 4/147 | 0.000136914 | 0.000827768 | CXCL10/SPP1/PIM1/CYP27B1 | 4 |
| GO:0043094 | cellular metabolic compound salvage | 4/147 | 0.000136914 | 0.000827768 | ADA/TYMP/CDA/TK1 | 4 |
| GO:0043276 | anoikis | 4/147 | 0.000136914 | 0.000827768 | BCL2/CAV1/CHEK2/E2F1 | 4 |
| GO:0009755 | hormone-mediated signaling pathway | 8/147 | 0.00013778 | 0.000830633 | PGR/AR/PARP1/NR3C2/ESRRB/GPER1/RXRG/ESRRG | 8 |
| GO:0046425 | regulation of receptor signaling pathway via JAK-STAT | 6/147 | 0.000139515 | 0.000838713 | F2/CYP1B1/IL6/CAV1/IFNG/F2R | 6 |
| GO:0051896 | regulation of protein kinase B signaling | 9/147 | 0.000140376 | 0.000841503 | MET/AKR1C3/ERBB2/IGF2/FGF1/FGF2/HSP90AA1/GPER1/AKR1C2 | 9 |
| GO:0009306 | protein secretion | 11/147 | 0.000145586 | 0.000870279 | DPP4/IL6/IFNG/IL1A/F2R/NR1H4/CACNA1C/CACNA1D/MTTP/GPER1/PLA2G1B | 11 |
| GO:0009411 | response to UV | 7/147 | 0.000148759 | 0.000886185 | CHEK1/BCL2/MMP1/MMP3/CDKN1A/MMP9/PARP1 | 7 |
| GO:0035592 | establishment of protein localization to extracellular region | 11/147 | 0.000149082 | 0.000886185 | DPP4/IL6/IFNG/IL1A/F2R/NR1H4/CACNA1C/CACNA1D/MTTP/GPER1/PLA2G1B | 11 |
| GO:0019932 | second-messenger-mediated signaling | 10/147 | 0.00015013 | 0.000889921 | CALM1/SELE/GSTM2/CXCL8/ADA/CACNA1C/TRPM8/GRIN2A/GRIN2D/NPPB | 10 |
| GO:0002791 | regulation of peptide secretion | 10/147 | 0.000154148 | 0.000911194 | DPP4/IL6/IFNG/IL1A/F2R/NR1H4/CACNA1C/CACNA1D/GPER1/PLA2G1B | 10 |
| GO:0043434 | response to peptide hormone | 12/147 | 0.000157642 | 0.00092748 | CCNA2/STAT1/SLC2A4/CAV1/PRKCB/COL1A1/PARP1/IGF2/CA2/NR1H4/GPER1/PLA2G1B | 12 |
| GO:0051917 | regulation of fibrinolysis | 3/147 | 0.00015851 | 0.00092748 | F2/THBD/SERPINE1 | 3 |
| GO:0070141 | response to UV-A | 3/147 | 0.00015851 | 0.00092748 | MMP1/MMP3/MMP9 | 3 |
| GO:0071222 | cellular response to lipopolysaccharide | 8/147 | 0.000158652 | 0.00092748 | IL6/CCL2/CXCL8/SERPINE1/IL1A/CXCL11/CXCL10/NR1H4 | 8 |
| GO:0070665 | positive regulation of leukocyte proliferation | 7/147 | 0.000161847 | 0.000940979 | BCL2/CDKN1A/IL6/IL2/IL1A/IGF2/ADA | 7 |
| GO:0097530 | granulocyte migration | 7/147 | 0.000161847 | 0.000940979 | DPP4/DPEP1/CCL2/CXCL8/CXCL11/CXCL10/PLA2G1B | 7 |
| GO:0018105 | peptidyl-serine phosphorylation | 10/147 | 0.000162457 | 0.000941942 | BCL2/CDK1/IL6/CAV1/PRKCB/CCNB1/IFNG/CHEK2/RPS6KA5/HSP90AA1 | 10 |
| GO:0071902 | positive regulation of protein serine/threonine kinase activity | 10/147 | 0.000166751 | 0.000964206 | CALM1/ADRB2/CDK1/ERBB2/CCNB1/IFNG/IGF2/FGF1/FGF2/PLA2G1B | 10 |
| GO:0051051 | negative regulation of transport | 12/147 | 0.000168 | 0.000966673 | CALM1/MAOB/KCNH2/PKIA/BCL2/GSTM2/MMP9/CAV1/PTGER3/PRKCB/ADA/F2R | 12 |
| GO:0045927 | positive regulation of growth | 9/147 | 0.000168089 | 0.000966673 | F2/SLC6A3/BCL2/ERBB2/IL2/CCNB1/IGF2/FGF2/PIM1 | 9 |
| GO:0061337 | cardiac conduction | 7/147 | 0.000168733 | 0.000967754 | CALM1/KCNH2/CAV1/ATP2A1/CACNA1C/CACNA1D/CACNB2 | 7 |
| GO:0050678 | regulation of epithelial cell proliferation | 11/147 | 0.000171596 | 0.000974884 | PGR/AR/STAT1/ERBB2/CAV1/CCL2/IGF2/GPBAR1/FGF1/FGF2/CDK6 | 11 |
| GO:0042311 | vasodilation | 4/147 | 0.000171814 | 0.000974884 | ADRB2/DRD1/ADRB3/GPER1 | 4 |
| GO:0086010 | membrane depolarization during action potential | 4/147 | 0.000171814 | 0.000974884 | KCNH2/CACNA1C/CACNA1D/CACNB2 | 4 |
| GO:2000310 | regulation of NMDA receptor activity | 4/147 | 0.000171814 | 0.000974884 | GRIA2/CCL2/IFNG/GRIN2A | 4 |
| GO:0071692 | protein localization to extracellular region | 11/147 | 0.000175616 | 0.00099244 | DPP4/IL6/IFNG/IL1A/F2R/NR1H4/CACNA1C/CACNA1D/MTTP/GPER1/PLA2G1B | 11 |
| GO:0007187 | G protein-coupled receptor signaling pathway, coupled to cyclic nucleotide second messenger | 5/147 | 0.000175843 | 0.00099244 | CHRM2/DRD1/CHRM4/CCL2/ADRB3 | 5 |
| GO:0010389 | regulation of G2/M transition of mitotic cell cycle | 8/147 | 0.000194792 | 0.001091565 | CHEK1/PKIA/CDK1/PSMD3/CDKN1A/CCNB1/HSP90AA1/HSPA2 | 8 |
| GO:0002070 | epithelial cell maturation | 3/147 | 0.000197007 | 0.001091565 | PGR/CDKN1A/TYMS | 3 |
| GO:0030397 | membrane disassembly | 3/147 | 0.000197007 | 0.001091565 | CDK1/PRKCB/CCNB1 | 3 |
| GO:0035635 | entry of bacterium into host cell | 3/147 | 0.000197007 | 0.001091565 | MET/CAV1/CXCL8 | 3 |
| GO:0042574 | retinal metabolic process | 3/147 | 0.000197007 | 0.001091565 | CYP1B1/AKR1C3/AKR1C1 | 3 |
| GO:0051081 | nuclear envelope disassembly | 3/147 | 0.000197007 | 0.001091565 | CDK1/PRKCB/CCNB1 | 3 |
| GO:0071732 | cellular response to nitric oxide | 3/147 | 0.000197007 | 0.001091565 | CCNA2/DPEP1/MMP3 | 3 |
| GO:0071156 | regulation of cell cycle arrest | 6/147 | 0.000201469 | 0.001113381 | CDK1/CDKN1A/CCNB1/CHEK2/E2F1/GPER1 | 6 |
| GO:0046883 | regulation of hormone secretion | 9/147 | 0.000206049 | 0.001135734 | DPP4/IL6/IFNG/SPP1/CYP19A1/NR1H4/CACNA1C/CACNA1D/GPER1 | 9 |
| GO:0002685 | regulation of leukocyte migration | 8/147 | 0.000208245 | 0.001144862 | DPP4/SELE/IL6/CCL2/CXCL8/SERPINE1/CXCL10/ADA | 8 |
| GO:0030301 | cholesterol transport | 6/147 | 0.000211852 | 0.00115155 | CAV1/PON1/NPC1L1/AKR1C1/SOAT2/CES1 | 6 |
| GO:1904659 | glucose transmembrane transport | 6/147 | 0.000211852 | 0.00115155 | SLC2A4/PRKCB/HK2/SLC5A2/SLC5A1/PLA2G1B | 6 |
| GO:0001504 | neurotransmitter uptake | 4/147 | 0.000212717 | 0.00115155 | DRD1/SLC6A3/SLC6A4/GLUL | 4 |
| GO:0008210 | estrogen metabolic process | 4/147 | 0.000212717 | 0.00115155 | CYP3A4/CYP1A1/CYP1B1/CYP19A1 | 4 |
| GO:0009163 | nucleoside biosynthetic process | 4/147 | 0.000212717 | 0.00115155 | ADA/TYMP/CDA/TK1 | 4 |
| GO:0071276 | cellular response to cadmium ion | 4/147 | 0.000212717 | 0.00115155 | JUN/AKR1C3/FOS/MMP9 | 4 |
| GO:0045930 | negative regulation of mitotic cell cycle | 10/147 | 0.000215219 | 0.001162133 | CHEK1/BCL2/CDK1/PSMD3/CDKN1A/CCL2/CCNB1/CHEK2/E2F1/CDK6 | 10 |
| GO:0051341 | regulation of oxidoreductase activity | 6/147 | 0.000222653 | 0.001196184 | CALM1/CAV1/IFNG/IL1A/HSP90AA1/CYP27B1 | 6 |
| GO:1904892 | regulation of receptor signaling pathway via STAT | 6/147 | 0.000222653 | 0.001196184 | F2/CYP1B1/IL6/CAV1/IFNG/F2R | 6 |
| GO:0030856 | regulation of epithelial cell differentiation | 7/147 | 0.000223859 | 0.001196603 | STAT1/MMP9/CAV1/SERPINE1/IFNG/FGF2/CYP27B1 | 7 |
| GO:0046165 | alcohol biosynthetic process | 7/147 | 0.000223859 | 0.001196603 | CYP3A4/NPC1L1/FGF1/FGF2/GPER1/CES1/CYP27B1 | 7 |
| GO:0030168 | platelet activation | 7/147 | 0.000232798 | 0.001241263 | F2/IL6/PRKCB/THBD/COL1A1/COL3A1/F2R | 7 |
| GO:0022612 | gland morphogenesis | 6/147 | 0.000233883 | 0.001243918 | PGR/AR/BCL2/IL6/CAV1/FGF1 | 6 |
| GO:0007190 | activation of adenylate cyclase activity | 4/147 | 0.0002356 | 0.001249921 | CALM1/ADRB2/DRD1/ADRB3 | 4 |
| GO:0071219 | cellular response to molecule of bacterial origin | 8/147 | 0.000237443 | 0.001254642 | IL6/CCL2/CXCL8/SERPINE1/IL1A/CXCL11/CXCL10/NR1H4 | 8 |
| GO:0071375 | cellular response to peptide hormone stimulus | 10/147 | 0.000237673 | 0.001254642 | CCNA2/SLC2A4/CAV1/PRKCB/PARP1/IGF2/CA2/NR1H4/GPER1/PLA2G1B | 10 |
| GO:0006206 | pyrimidine nucleobase metabolic process | 3/147 | 0.000241087 | 0.001260129 | TYMS/TYMP/CDA | 3 |
| GO:0031649 | heat generation | 3/147 | 0.000241087 | 0.001260129 | ADRB2/PTGER3/IL1A | 3 |
| GO:0043174 | nucleoside salvage | 3/147 | 0.000241087 | 0.001260129 | TYMP/CDA/TK1 | 3 |
| GO:0046134 | pyrimidine nucleoside biosynthetic process | 3/147 | 0.000241087 | 0.001260129 | TYMP/CDA/TK1 | 3 |
| GO:0044106 | cellular amine metabolic process | 7/147 | 0.000242024 | 0.001261917 | MAOB/DRD1/SLC6A3/PSMD3/MAOA/NR1H4/GRIN2A | 7 |
| GO:0008645 | hexose transmembrane transport | 6/147 | 0.000245554 | 0.001277183 | SLC2A4/PRKCB/HK2/SLC5A2/SLC5A1/PLA2G1B | 6 |
| GO:0033627 | cell adhesion mediated by integrin | 5/147 | 0.000246931 | 0.001278083 | DPP4/CYP1B1/PLAU/SERPINE1/ADA | 5 |
| GO:1903036 | positive regulation of response to wounding | 5/147 | 0.000246931 | 0.001278083 | F2/THBD/SERPINE1/CLDN4/F2R | 5 |
| GO:0050863 | regulation of T cell activation | 10/147 | 0.000249617 | 0.001288839 | DPP4/IL6/ERBB2/CAV1/CCL2/IL2/IFNG/IL1A/IGF2/ADA | 10 |
| GO:1901653 | cellular response to peptide | 11/147 | 0.000251684 | 0.001296359 | CCNA2/ADRB2/SLC2A4/CAV1/PRKCB/PARP1/IGF2/CA2/NR1H4/GPER1/PLA2G1B | 11 |
| GO:0008207 | C21-steroid hormone metabolic process | 4/147 | 0.000260202 | 0.001320995 | AKR1C3/CYP17A1/AKR1C1/AKR1C2 | 4 |
| GO:0030890 | positive regulation of B cell proliferation | 4/147 | 0.000260202 | 0.001320995 | BCL2/CDKN1A/IL2/ADA | 4 |
| GO:0045776 | negative regulation of blood pressure | 4/147 | 0.000260202 | 0.001320995 | ADRB2/ADRA1A/ADRB3/NPPB | 4 |
| GO:0070296 | sarcoplasmic reticulum calcium ion transport | 4/147 | 0.000260202 | 0.001320995 | CALM1/GSTM2/ATP2A1/CACNA1C | 4 |
| GO:0072528 | pyrimidine-containing compound biosynthetic process | 4/147 | 0.000260202 | 0.001320995 | TYMS/TYMP/CDA/TK1 | 4 |
| GO:0150077 | regulation of neuroinflammatory response | 4/147 | 0.000260202 | 0.001320995 | MMP3/MMP9/IL6/MMP8 | 4 |
| GO:0006809 | nitric oxide biosynthetic process | 5/147 | 0.000263441 | 0.001334245 | CYP1B1/CAV1/IFNG/MMP8/HSP90AA1 | 5 |
| GO:0030336 | negative regulation of cell migration | 10/147 | 0.000268475 | 0.001356503 | DPP4/DPEP1/BCL2/CYP1B1/CCL2/SERPINE1/COL3A1/IGFBP3/ADA/FGF2 | 10 |
| GO:0015749 | monosaccharide transmembrane transport | 6/147 | 0.000270269 | 0.00136197 | SLC2A4/PRKCB/HK2/SLC5A2/SLC5A1/PLA2G1B | 6 |
| GO:0009408 | response to heat | 7/147 | 0.000271482 | 0.00136197 | CDKN1A/IL1A/CXCL10/TRPV3/FGF1/HSP90AA1/HSPA2 | 7 |
| GO:0098739 | import across plasma membrane | 7/147 | 0.000271482 | 0.00136197 | KCNH2/IFNG/TRPV3/SLC5A2/SLC5A1/FOLR2/SLC46A1 | 7 |
| GO:1903532 | positive regulation of secretion by cell | 9/147 | 0.000279961 | 0.001398671 | SLC6A4/IFNG/IL1A/SPP1/CYP19A1/NR1H4/CACNB2/GPER1/PLA2G1B | 9 |
| GO:0046323 | glucose import | 5/147 | 0.000280775 | 0.001398671 | SLC2A4/HK2/SLC5A2/SLC5A1/PLA2G1B | 5 |
| GO:0061045 | negative regulation of wound healing | 5/147 | 0.000280775 | 0.001398671 | F2/PLAU/THBD/SERPINE1/FGF2 | 5 |
| GO:0010523 | negative regulation of calcium ion transport into cytosol | 3/147 | 0.00029108 | 0.001439865 | CALM1/BCL2/GSTM2 | 3 |
| GO:0030299 | intestinal cholesterol absorption | 3/147 | 0.00029108 | 0.001439865 | NPC1L1/AKR1C1/SOAT2 | 3 |
| GO:1902170 | cellular response to reactive nitrogen species | 3/147 | 0.00029108 | 0.001439865 | CCNA2/DPEP1/MMP3 | 3 |
| GO:0034219 | carbohydrate transmembrane transport | 6/147 | 0.000296898 | 0.00146523 | SLC2A4/PRKCB/HK2/SLC5A2/SLC5A1/PLA2G1B | 6 |
| GO:0006073 | cellular glucan metabolic process | 5/147 | 0.000298961 | 0.001468581 | CALM1/PYGM/MGAM/IGF2/ESRRB | 5 |
| GO:0044042 | glucan metabolic process | 5/147 | 0.000298961 | 0.001468581 | CALM1/PYGM/MGAM/IGF2/ESRRB | 5 |
| GO:0097191 | extrinsic apoptotic signaling pathway | 8/147 | 0.000305941 | 0.001499397 | AR/BCL2/CAV1/IL2/SERPINE1/IFNG/IL1A/GPER1 | 8 |
| GO:0002790 | peptide secretion | 11/147 | 0.000312126 | 0.001526186 | DPP4/IL6/IFNG/IL1A/F2R/NR1H4/CACNA1C/CACNA1D/MTTP/GPER1/PLA2G1B | 11 |
| GO:1901659 | glycosyl compound biosynthetic process | 4/147 | 0.000314858 | 0.001536005 | ADA/TYMP/CDA/TK1 | 4 |
| GO:0016049 | cell growth | 12/147 | 0.000320021 | 0.001557612 | F2/ADRA1A/BCL2/CDKN1A/ERBB2/IL2/SPP1/IGFBP3/HSP90AA1/CDA/NPPB/CYP27B1 | 12 |
| GO:1902749 | regulation of cell cycle G2/M phase transition | 8/147 | 0.000325373 | 0.00157726 | CHEK1/PKIA/CDK1/PSMD3/CDKN1A/CCNB1/HSP90AA1/HSPA2 | 8 |
| GO:0003014 | renal system process | 6/147 | 0.000325545 | 0.00157726 | ADRA1A/BCL2/AKR1C3/CLDN4/F2R/NPPB | 6 |
| GO:0046209 | nitric oxide metabolic process | 5/147 | 0.000338 | 0.001633875 | CYP1B1/CAV1/IFNG/MMP8/HSP90AA1 | 5 |
| GO:0002688 | regulation of leukocyte chemotaxis | 6/147 | 0.000340657 | 0.001639252 | DPP4/IL6/CCL2/CXCL8/SERPINE1/CXCL10 | 6 |
| GO:0034766 | negative regulation of ion transmembrane transport | 6/147 | 0.000340657 | 0.001639252 | CALM1/KCNH2/GSTM2/MMP9/CAV1/PRKCB | 6 |
| GO:0015701 | bicarbonate transport | 4/147 | 0.000345063 | 0.001652765 | CA2/CA1/CA7/CA14 | 4 |
| GO:0032620 | interleukin-17 production | 4/147 | 0.000345063 | 0.001652765 | IL6/IL2/IFNG/NR1H4 | 4 |
| GO:0050670 | regulation of lymphocyte proliferation | 8/147 | 0.000345802 | 0.001652765 | BCL2/CDKN1A/IL6/ERBB2/IL2/IL1A/IGF2/ADA | 8 |
| GO:0007596 | blood coagulation | 10/147 | 0.000356292 | 0.001691578 | F2/PLAU/IL6/CAV1/PRKCB/THBD/SERPINE1/COL1A1/COL3A1/F2R | 10 |
| GO:0034763 | negative regulation of transmembrane transport | 6/147 | 0.000356314 | 0.001691578 | CALM1/KCNH2/GSTM2/MMP9/CAV1/PRKCB | 6 |
| GO:0070252 | actin-mediated cell contraction | 6/147 | 0.000356314 | 0.001691578 | KCNH2/CAV1/ATP2A1/CACNA1C/CACNA1D/CACNB2 | 6 |
| GO:0061844 | antimicrobial humoral immune response mediated by antimicrobial peptide | 5/147 | 0.000358908 | 0.001696303 | F2/CXCL8/CXCL11/CXCL10/PLA2G1B | 5 |
| GO:2001057 | reactive nitrogen species metabolic process | 5/147 | 0.000358908 | 0.001696303 | CYP1B1/CAV1/IFNG/MMP8/HSP90AA1 | 5 |
| GO:0046427 | positive regulation of receptor signaling pathway via JAK-STAT | 4/147 | 0.000377286 | 0.001779201 | F2/CYP1B1/IL6/F2R | 4 |
| GO:2000146 | negative regulation of cell motility | 10/147 | 0.000381646 | 0.001795772 | DPP4/DPEP1/BCL2/CYP1B1/CCL2/SERPINE1/COL3A1/IGFBP3/ADA/FGF2 | 10 |
| GO:0097193 | intrinsic apoptotic signaling pathway | 9/147 | 0.000384403 | 0.001804744 | BCL2/CYP1B1/CDKN1A/MMP9/CAV1/PARP1/CHEK2/E2F1/ATP2A1 | 9 |
| GO:0019218 | regulation of steroid metabolic process | 6/147 | 0.000389315 | 0.001823767 | AKR1C3/IFNG/FGF1/NR1H4/CES1/CYP27B1 | 6 |
| GO:0007599 | hemostasis | 10/147 | 0.000390424 | 0.001824274 | F2/PLAU/IL6/CAV1/PRKCB/THBD/SERPINE1/COL1A1/COL3A1/F2R | 10 |
| GO:0001659 | temperature homeostasis | 7/147 | 0.000391142 | 0.001824274 | ADRB2/CAV1/PTGER3/IL1A/ADRB3/TRPM8/ESRRG | 7 |
| GO:0050817 | coagulation | 10/147 | 0.000399371 | 0.001858567 | F2/PLAU/IL6/CAV1/PRKCB/THBD/SERPINE1/COL1A1/COL3A1/F2R | 10 |
| GO:0032943 | mononuclear cell proliferation | 9/147 | 0.000404591 | 0.001878743 | BCL2/CDKN1A/IL6/ERBB2/IL2/IL1A/CRP/IGF2/ADA | 9 |
| GO:0009110 | vitamin biosynthetic process | 3/147 | 0.000410075 | 0.001891789 | CYP3A4/IFNG/CYP27B1 | 3 |
| GO:0071731 | response to nitric oxide | 3/147 | 0.000410075 | 0.001891789 | CCNA2/DPEP1/MMP3 | 3 |
| GO:0098856 | intestinal lipid absorption | 3/147 | 0.000410075 | 0.001891789 | NPC1L1/AKR1C1/SOAT2 | 3 |
| GO:0014910 | regulation of smooth muscle cell migration | 5/147 | 0.000427528 | 0.001963766 | BCL2/PLAU/SERPINE1/IGFBP3/NOX4 | 5 |
| GO:0099601 | regulation of neurotransmitter receptor activity | 5/147 | 0.000427528 | 0.001963766 | GRIA2/ADRB2/CCL2/IFNG/GRIN2A | 5 |
| GO:0051271 | negative regulation of cellular component movement | 10/147 | 0.000446707 | 0.002044953 | DPP4/DPEP1/BCL2/CYP1B1/CCL2/SERPINE1/COL3A1/IGFBP3/ADA/FGF2 | 10 |
| GO:0051932 | synaptic transmission, GABAergic | 4/147 | 0.000448094 | 0.002044953 | ADRA1A/CA7/GABRA3/GABRA5 | 4 |
| GO:2001258 | negative regulation of cation channel activity | 4/147 | 0.000448094 | 0.002044953 | CALM1/GSTM2/MMP9/CAV1 | 4 |
| GO:0071158 | positive regulation of cell cycle arrest | 5/147 | 0.000452464 | 0.002060466 | CDK1/CDKN1A/CCNB1/CHEK2/E2F1 | 5 |
| GO:0045471 | response to ethanol | 6/147 | 0.000462459 | 0.002101469 | MAOB/SLC6A3/SLC2A4/IL2/TYMS/GRIN2A | 6 |
| GO:1901019 | regulation of calcium ion transmembrane transporter activity | 5/147 | 0.000478481 | 0.002152138 | CALM1/GSTM2/ATP2A1/CACNB2/HSPA2 | 5 |
| GO:1904705 | regulation of vascular associated smooth muscle cell proliferation | 5/147 | 0.000478481 | 0.002152138 | JUN/CDKN1A/MMP9/FGF2/GPER1 | 5 |
| GO:1990874 | vascular associated smooth muscle cell proliferation | 5/147 | 0.000478481 | 0.002152138 | JUN/CDKN1A/MMP9/FGF2/GPER1 | 5 |
| GO:0019373 | epoxygenase P450 pathway | 3/147 | 0.000479694 | 0.002152138 | CYP1A1/CYP1B1/CYP2C19 | 3 |
| GO:0090280 | positive regulation of calcium ion import | 3/147 | 0.000479694 | 0.002152138 | CCL2/TRPV3/ATP2A1 | 3 |
| GO:1902644 | tertiary alcohol metabolic process | 3/147 | 0.000479694 | 0.002152138 | AKR1C3/AKR1C1/AKR1C2 | 3 |
| GO:0030574 | collagen catabolic process | 4/147 | 0.000486834 | 0.002174973 | MMP1/MMP3/MMP9/MMP8 | 4 |
| GO:0030857 | negative regulation of epithelial cell differentiation | 4/147 | 0.000486834 | 0.002174973 | STAT1/MMP9/CAV1/IFNG | 4 |
| GO:0090066 | regulation of anatomical structure size | 12/147 | 0.000490742 | 0.002186616 | ADRB2/ADRA1D/DRD1/ADRA1A/SLC6A4/CAV1/CRP/SPP1/ADRB3/F2R/GPER1/NPPB | 12 |
| GO:0071216 | cellular response to biotic stimulus | 8/147 | 0.0004915 | 0.002186616 | IL6/CCL2/CXCL8/SERPINE1/IL1A/CXCL11/CXCL10/NR1H4 | 8 |
| GO:0050796 | regulation of insulin secretion | 7/147 | 0.000497584 | 0.002209051 | DPP4/IL6/IFNG/NR1H4/CACNA1C/CACNA1D/GPER1 | 7 |
| GO:0030195 | negative regulation of blood coagulation | 4/147 | 0.0005279 | 0.002333872 | F2/PLAU/THBD/SERPINE1 | 4 |
| GO:1904894 | positive regulation of receptor signaling pathway via STAT | 4/147 | 0.0005279 | 0.002333872 | F2/CYP1B1/IL6/F2R | 4 |
| GO:0032370 | positive regulation of lipid transport | 5/147 | 0.000533876 | 0.002350502 | CAV1/SPP1/PON1/CYP19A1/CES1 | 5 |
| GO:0046928 | regulation of neurotransmitter secretion | 5/147 | 0.000533876 | 0.002350502 | CALM1/ADRA1A/PRKCB/CACNB2/GPER1 | 5 |
| GO:1900180 | regulation of protein localization to nucleus | 6/147 | 0.000545901 | 0.002398466 | F2/PKIA/CDK1/IFNG/PARP1/GLUL | 6 |
| GO:0010875 | positive regulation of cholesterol efflux | 3/147 | 0.000556457 | 0.002424764 | CAV1/PON1/CES1 | 3 |
| GO:0016137 | glycoside metabolic process | 3/147 | 0.000556457 | 0.002424764 | AKR1C3/AKR1C1/AKR1C2 | 3 |
| GO:0044241 | lipid digestion | 3/147 | 0.000556457 | 0.002424764 | NPC1L1/AKR1C1/SOAT2 | 3 |
| GO:2000479 | regulation of cAMP-dependent protein kinase activity | 3/147 | 0.000556457 | 0.002424764 | ADRB2/PKIA/CXCL10 | 3 |
| GO:0007589 | body fluid secretion | 5/147 | 0.000563316 | 0.002444614 | SLC6A3/CAV1/HK2/ADA/NPPB | 5 |
| GO:0032755 | positive regulation of interleukin-6 production | 5/147 | 0.000563316 | 0.002444614 | IL6/IFNG/IL1A/MMP8/F2R | 5 |
| GO:0042770 | signal transduction in response to DNA damage | 6/147 | 0.000568483 | 0.002462001 | CHEK1/CDK1/CDKN1A/CCNB1/CHEK2/E2F1 | 6 |
| GO:1900047 | negative regulation of hemostasis | 4/147 | 0.00057137 | 0.002469467 | F2/PLAU/THBD/SERPINE1 | 4 |
| GO:0006112 | energy reserve metabolic process | 5/147 | 0.000593959 | 0.002556679 | CALM1/PYGM/IGF2/ADRB3/ESRRB | 5 |
| GO:1904063 | negative regulation of cation transmembrane transport | 5/147 | 0.000593959 | 0.002556679 | CALM1/KCNH2/GSTM2/MMP9/CAV1 | 5 |
| GO:0046879 | hormone secretion | 9/147 | 0.000614137 | 0.00263587 | DPP4/IL6/IFNG/SPP1/CYP19A1/NR1H4/CACNA1C/CACNA1D/GPER1 | 9 |
| GO:0050729 | positive regulation of inflammatory response | 6/147 | 0.000615821 | 0.00263587 | IL6/PTGER3/IL2/SERPINE1/IFNG/MMP8 | 6 |
| GO:0008206 | bile acid metabolic process | 4/147 | 0.000617324 | 0.00263587 | NR1H4/SULT2A1/AKR1C1/CES1 | 4 |
| GO:0045744 | negative regulation of G protein-coupled receptor signaling pathway | 4/147 | 0.000617324 | 0.00263587 | ADRB2/MET/CXCL8/ADA | 4 |
| GO:0014909 | smooth muscle cell migration | 5/147 | 0.000625835 | 0.002666842 | BCL2/PLAU/SERPINE1/IGFBP3/NOX4 | 5 |
| GO:0090068 | positive regulation of cell cycle process | 9/147 | 0.000628806 | 0.002674134 | CDK1/CYP1A1/CDKN1A/CCNB1/IL1A/CHEK2/E2F1/IGF2/HSPA2 | 9 |

# Supplementary 14

**Core targets-Signal pathways of KEGG enrichment analysis**

**Supplementary table 14. Core targets-Signal pathways of KEGG enrichment analysis.**

| ID | Description | GeneRatio | pvalue | qvalue | geneID | Count |
| --- | --- | --- | --- | --- | --- | --- |
| hsa05031 | Amphetamine addiction | 14/135 | 4.63E-12 | 6.33E-10 | GRIA2/CALM1/MAOB/DRD1/SLC6A3/JUN/FOS/PRKCB/MAOA/CACNA1C/CACNA1D/GRIN2A/GRIN2D/GRIN3B | 14 |
| hsa05207 | Chemical carcinogenesis - receptor activation | 21/135 | 3.39E-11 | 2.32E-09 | PGR/AR/ADRB2/JUN/BCL2/CYP3A4/CYP1A1/CYP1B1/NR1I3/GSTM1/GSTM2/FOS/PRKCB/BIRC5/E2F1/ADRB3/FGF2/HSP90AA1/CACNA1C/CACNA1D/RXRG | 21 |
| hsa04933 | AGE-RAGE signaling pathway in diabetic complications | 15/135 | 7.47E-11 | 3.41E-09 | JUN/BCL2/STAT1/SELE/IL6/CCL2/CXCL8/PRKCB/THBD/SERPINE1/COL1A1/IL1A/COL3A1/NOX4/PIM1 | 15 |
| hsa05033 | Nicotine addiction | 10/135 | 6.61E-10 | 2.26E-08 | GRIA2/GABRA3/GABRA5/GABRB1/GABRD/GABRP/GABRQ/GRIN2A/GRIN2D/GRIN3B | 10 |
| hsa04020 | Calcium signaling pathway | 19/135 | 1.40E-08 | 3.82E-07 | CHRM2/CALM1/ADRB2/ADRA1D/DRD1/ADRA1A/MET/ERBB2/PTGER3/PRKCB/ADRB3/ATP2A1/F2R/FGF1/FGF2/CACNA1C/CACNA1D/GRIN2A/GRIN2D | 19 |
| hsa04218 | Cellular senescence | 15/135 | 4.03E-08 | 9.18E-07 | CHEK1/CALM1/CCNA2/CDK1/CDKN1A/IL6/CXCL8/CCNB1/SERPINE1/IL1A/CHEK2/E2F1/IGFBP3/CACNA1D/CDK6 | 15 |
| hsa05418 | Fluid shear stress and atherosclerosis | 14/135 | 6.52E-08 | 1.27E-06 | CALM1/JUN/BCL2/SELE/GSTM1/GSTM2/FOS/MMP9/CAV1/CCL2/THBD/IFNG/IL1A/HSP90AA1 | 14 |
| hsa05030 | Cocaine addiction | 9/135 | 8.93E-08 | 1.53E-06 | GRIA2/MAOB/DRD1/SLC6A3/JUN/MAOA/GRIN2A/GRIN2D/GRIN3B | 9 |
| hsa05219 | Bladder cancer | 8/135 | 2.93E-07 | 4.45E-06 | MMP1/CDKN1A/MMP9/ERBB2/CXCL8/E2F1/RPS6KA5/TYMP | 8 |
| hsa04657 | IL-17 signaling pathway | 11/135 | 3.93E-07 | 5.37E-06 | JUN/MMP1/MMP3/FOS/MMP9/IL6/CCL2/CXCL8/IFNG/CXCL10/HSP90AA1 | 11 |
| hsa05417 | Lipid and atherosclerosis | 16/135 | 4.97E-07 | 6.18E-06 | CALM1/OLR1/JUN/BCL2/MMP1/CYP1A1/SELE/MMP3/FOS/MMP9/IL6/CCL2/CXCL8/HSP90AA1/HSPA2/RXRG | 16 |
| hsa00980 | Metabolism of xenobiotics by cytochrome P450 | 10/135 | 5.73E-07 | 6.53E-06 | ADH1B/ADH1C/CYP3A4/CYP1A1/CYP1B1/GSTM1/GSTM2/SULT2A1/AKR1C1/CBR1 | 10 |
| hsa04080 | Neuroactive ligand-receptor interaction | 20/135 | 8.02E-07 | 8.44E-06 | CHRM2/GRIA2/F2/ADRB2/ADRA1D/DRD1/CHRM4/ADRA1A/PTGER3/ADRB3/F2R/GABRA3/GABRA5/GABRB1/GABRD/GABRP/GABRQ/GRIN2A/GRIN2D/GRIN3B | 20 |
| hsa05204 | Chemical carcinogenesis - DNA adducts | 9/135 | 1.86E-06 | 1.81E-05 | CYP3A4/CYP1A1/CYP1B1/GSTM1/GSTM2/CYP2C19/SULT2A1/CBR1/AKR1C2 | 9 |
| hsa04727 | GABAergic synapse | 10/135 | 1.98E-06 | 1.81E-05 | PRKCB/GLUL/CACNA1C/CACNA1D/GABRA3/GABRA5/GABRB1/GABRD/GABRP/GABRQ | 10 |
| hsa04115 | p53 signaling pathway | 9/135 | 3.01E-06 | 2.57E-05 | CHEK1/BCL2/CDK1/CDKN1A/CCNB1/SERPINE1/CHEK2/IGFBP3/CDK6 | 9 |
| hsa04024 | cAMP signaling pathway | 15/135 | 3.28E-06 | 2.64E-05 | CHRM2/GRIA2/CALM1/ADRB2/DRD1/JUN/FOS/PTGER3/ATP2A1/F2R/CACNA1C/CACNA1D/GRIN2A/GRIN2D/GRIN3B | 15 |
| hsa00910 | Nitrogen metabolism | 5/135 | 6.32E-06 | 4.80E-05 | GLUL/CA2/CA1/CA7/CA14 | 5 |
| hsa00140 | Steroid hormone biosynthesis | 8/135 | 6.82E-06 | 4.91E-05 | CYP3A4/CYP1A1/CYP1B1/AKR1C3/CYP19A1/CYP17A1/AKR1C1/AKR1C2 | 8 |
| hsa04728 | Dopaminergic synapse | 11/135 | 1.15E-05 | 7.86E-05 | GRIA2/CALM1/MAOB/DRD1/SLC6A3/FOS/PRKCB/MAOA/CACNA1C/CACNA1D/GRIN2A | 11 |
| hsa05161 | Hepatitis B | 12/135 | 1.52E-05 | 9.89E-05 | CCNA2/JUN/BCL2/STAT1/FOS/CDKN1A/MMP9/IL6/CXCL8/PRKCB/BIRC5/E2F1 | 12 |
| hsa05323 | Rheumatoid arthritis | 9/135 | 2.25E-05 | 0.000139981 | JUN/MMP1/MMP3/FOS/IL6/CCL2/CXCL8/IFNG/IL1A | 9 |
| hsa00982 | Drug metabolism - cytochrome P450 | 8/135 | 2.38E-05 | 0.00014131 | ADH1B/ADH1C/MAOB/CYP3A4/GSTM1/GSTM2/MAOA/CYP2C19 | 8 |
| hsa04713 | Circadian entrainment | 9/135 | 3.16E-05 | 0.000173017 | GRIA2/CALM1/FOS/PRKCB/RPS6KA5/CACNA1C/CACNA1D/GRIN2A/GRIN2D | 9 |
| hsa05215 | Prostate cancer | 9/135 | 3.16E-05 | 0.000173017 | AR/BCL2/MMP3/CDKN1A/PLAU/MMP9/ERBB2/E2F1/HSP90AA1 | 9 |
| hsa05202 | Transcriptional misregulation in cancer | 12/135 | 8.14E-05 | 0.000422489 | CCNA2/MET/MMP3/CDKN1A/PLAU/MMP9/IL6/RUNX1T1/CXCL8/IGFBP3/RXRG/HOXA10 | 12 |
| hsa05208 | Chemical carcinogenesis - reactive oxygen species | 13/135 | 8.34E-05 | 0.000422489 | MET/JUN/CYP1A1/CYP1B1/GSTM1/GSTM2/AKR1C3/FOS/NOX4/AKR1C1/CBR1/AKR1C2/COX7A1 | 13 |
| hsa04668 | TNF signaling pathway | 9/135 | 9.84E-05 | 0.000480997 | JUN/SELE/MMP3/FOS/MMP9/IL6/CCL2/CXCL10/RPS6KA5 | 9 |
| hsa04726 | Serotonergic synapse | 9/135 | 0.000120761 | 0.000569836 | PTGS1/MAOB/SLC6A4/PRKCB/MAOA/CYP2C19/CACNA1C/CACNA1D/GABRB1 | 9 |
| hsa05032 | Morphine addiction | 8/135 | 0.000129322 | 0.000589888 | DRD1/PRKCB/GABRA3/GABRA5/GABRB1/GABRD/GABRP/GABRQ | 8 |
| hsa01523 | Antifolate resistance | 5/135 | 0.000143761 | 0.000634597 | IL6/ABCG2/TYMS/FOLR2/SLC46A1 | 5 |
| hsa05144 | Malaria | 6/135 | 0.000167648 | 0.000715442 | MET/SELE/IL6/CCL2/CXCL8/IFNG | 6 |
| hsa04723 | Retrograde endocannabinoid signaling | 10/135 | 0.000172532 | 0.000715442 | GRIA2/PRKCB/CACNA1C/CACNA1D/GABRA3/GABRA5/GABRB1/GABRD/GABRP/GABRQ | 10 |
| hsa05223 | Non-small cell lung cancer | 7/135 | 0.000183714 | 0.000739407 | MET/CDKN1A/ERBB2/PRKCB/E2F1/CDK6/RXRG | 7 |
| hsa04261 | Adrenergic signaling in cardiomyocytes | 10/135 | 0.000192643 | 0.000753191 | CALM1/ADRB2/ADRA1D/ADRA1A/BCL2/RPS6KA5/ATP2A1/CACNA1C/CACNA1D/CACNB2 | 10 |
| hsa01524 | Platinum drug resistance | 7/135 | 0.000200409 | 0.00076179 | BCL2/GSTM1/GSTM2/CDKN1A/ERBB2/BIRC5/TOP2A | 7 |
| hsa01522 | Endocrine resistance | 8/135 | 0.000217176 | 0.00080321 | JUN/BCL2/FOS/CDKN1A/MMP9/ERBB2/E2F1/GPER1 | 8 |
| hsa05142 | Chagas disease | 8/135 | 0.000286222 | 0.001030716 | JUN/FOS/IL6/CCL2/CXCL8/IL2/SERPINE1/IFNG | 8 |
| hsa04620 | Toll-like receptor signaling pathway | 8/135 | 0.000326942 | 0.001147165 | JUN/STAT1/FOS/IL6/CXCL8/CXCL11/CXCL10/SPP1 | 8 |
| hsa00983 | Drug metabolism - other enzymes | 7/135 | 0.000354718 | 0.001213508 | CYP3A4/GSTM1/GSTM2/TYMP/CDA/TK1/CES1 | 7 |
| hsa04010 | MAPK signaling pathway | 14/135 | 0.000372871 | 0.0012445 | MET/JUN/FOS/ERBB2/PRKCB/IL1A/IGF2/RPS6KA5/FGF1/FGF2/CACNA1C/CACNA1D/CACNB2/HSPA2 | 14 |
| hsa04659 | Th17 cell differentiation | 8/135 | 0.000422604 | 0.001376904 | JUN/STAT1/FOS/IL6/IL2/IFNG/HSP90AA1/RXRG | 8 |
| hsa04066 | HIF-1 signaling pathway | 8/135 | 0.000449768 | 0.00143133 | BCL2/CDKN1A/IL6/ERBB2/PRKCB/SERPINE1/IFNG/HK2 | 8 |
| hsa05171 | Coronavirus disease - COVID-19 | 12/135 | 0.000475723 | 0.001449156 | F2/JUN/MMP1/STAT1/MMP3/FOS/IL6/CCL2/CXCL8/PRKCB/IL2/CXCL10 | 12 |
| hsa04915 | Estrogen signaling pathway | 9/135 | 0.000476549 | 0.001449156 | PGR/CALM1/JUN/BCL2/FOS/MMP9/HSP90AA1/GPER1/HSPA2 | 9 |
| hsa05162 | Measles | 9/135 | 0.000502447 | 0.001494694 | JUN/BCL2/STAT1/FOS/IL6/IL2/IL1A/CDK6/HSPA2 | 9 |
| hsa04724 | Glutamatergic synapse | 8/135 | 0.000607717 | 0.001769388 | GRIA2/PRKCB/GLUL/CACNA1C/CACNA1D/GRIN2A/GRIN2D/GRIN3B | 8 |
| hsa05321 | Inflammatory bowel disease | 6/135 | 0.000710371 | 0.00202518 | JUN/STAT1/IL6/IL2/IFNG/IL1A | 6 |
| hsa05224 | Breast cancer | 9/135 | 0.00075422 | 0.002106306 | PGR/JUN/FOS/CDKN1A/ERBB2/E2F1/FGF1/FGF2/CDK6 | 9 |
| hsa04151 | PI3K-Akt signaling pathway | 15/135 | 0.000784904 | 0.002148158 | CHRM2/MET/BCL2/CDKN1A/IL6/ERBB2/IL2/COL1A1/SPP1/IGF2/F2R/FGF1/FGF2/HSP90AA1/CDK6 | 15 |
| hsa04720 | Long-term potentiation | 6/135 | 0.000835245 | 0.002241112 | GRIA2/CALM1/PRKCB/CACNA1C/GRIN2A/GRIN2D | 6 |
| hsa04973 | Carbohydrate digestion and absorption | 5/135 | 0.001048294 | 0.002758668 | PRKCB/MGAM/HK2/SLC5A1/CACNA1D | 5 |
| hsa04110 | Cell cycle | 8/135 | 0.00117365 | 0.003030279 | CHEK1/CCNA2/CDK1/CDKN1A/CCNB1/CHEK2/E2F1/CDK6 | 8 |
| hsa05218 | Melanoma | 6/135 | 0.001221785 | 0.003096141 | MET/CDKN1A/E2F1/FGF1/FGF2/CDK6 | 6 |
| hsa05167 | Kaposi sarcoma-associated herpesvirus infection | 10/135 | 0.001456967 | 0.003624991 | CALM1/JUN/STAT1/FOS/CDKN1A/IL6/CXCL8/E2F1/FGF2/CDK6 | 10 |
| hsa04913 | Ovarian steroidogenesis | 5/135 | 0.001522163 | 0.003719571 | CYP1A1/CYP1B1/AKR1C3/CYP19A1/CYP17A1 | 5 |
| hsa05133 | Pertussis | 6/135 | 0.001619281 | 0.003887472 | CALM1/JUN/FOS/IL6/CXCL8/IL1A | 6 |
| hsa04270 | Vascular smooth muscle contraction | 8/135 | 0.001661265 | 0.003919501 | CALM1/ADRA1D/ADRA1A/PRKCB/CACNA1C/CACNA1D/NPPB/PLA2G1B | 8 |
| hsa05140 | Leishmaniasis | 6/135 | 0.00173247 | 0.004018218 | JUN/STAT1/FOS/PRKCB/IFNG/IL1A | 6 |
| hsa04022 | cGMP-PKG signaling pathway | 9/135 | 0.001852454 | 0.004224896 | CALM1/ADRB2/ADRA1D/ADRA1A/ADRB3/ATP2A1/CACNA1C/CACNA1D/NPPB | 9 |
| hsa05169 | Epstein-Barr virus infection | 10/135 | 0.001968917 | 0.004363014 | CCNA2/JUN/BCL2/STAT1/PSMD3/CDKN1A/IL6/CXCL10/E2F1/CDK6 | 10 |
| hsa01521 | EGFR tyrosine kinase inhibitor resistance | 6/135 | 0.001976781 | 0.004363014 | MET/BCL2/IL6/ERBB2/PRKCB/FGF2 | 6 |
| hsa05205 | Proteoglycans in cancer | 10/135 | 0.002194858 | 0.004767445 | MET/CDKN1A/PLAU/MMP9/ERBB2/CAV1/PRKCB/COL1A1/IGF2/FGF2 | 10 |
| hsa05164 | Influenza A | 9/135 | 0.00226736 | 0.004847973 | STAT1/IL6/CCL2/CXCL8/PRKCB/IFNG/IL1A/CXCL10/CDK6 | 9 |
| hsa04725 | Cholinergic synapse | 7/135 | 0.00273788 | 0.005763957 | CHRM2/CHRM4/BCL2/FOS/PRKCB/CACNA1C/CACNA1D | 7 |
| hsa00350 | Tyrosine metabolism | 4/135 | 0.002883886 | 0.005979349 | ADH1B/ADH1C/MAOB/MAOA | 4 |
| hsa05143 | African trypanosomiasis | 4/135 | 0.00319219 | 0.006519791 | SELE/IL6/PRKCB/IFNG | 4 |
| hsa00590 | Arachidonic acid metabolism | 5/135 | 0.003375935 | 0.006703391 | PTGS1/AKR1C3/CYP2C19/CBR1/PLA2G1B | 5 |
| hsa05226 | Gastric cancer | 8/135 | 0.003380056 | 0.006703391 | MET/BCL2/CDKN1A/ERBB2/E2F1/FGF1/FGF2/RXRG | 8 |
| hsa04976 | Bile secretion | 6/135 | 0.003609545 | 0.007056254 | CYP3A4/ABCG2/SLC5A1/CA2/NR1H4/SULT2A1 | 6 |
| hsa05034 | Alcoholism | 9/135 | 0.00397117 | 0.007653849 | CALM1/MAOB/DRD1/SLC6A3/PKIA/MAOA/GRIN2A/GRIN2D/GRIN3B | 9 |
| hsa04921 | Oxytocin signaling pathway | 8/135 | 0.00413305 | 0.007794721 | CALM1/JUN/FOS/CDKN1A/PRKCB/CACNA1C/CACNA1D/CACNB2 | 8 |
| hsa04929 | GnRH secretion | 5/135 | 0.004158184 | 0.007794721 | PRKCB/SPP1/CACNA1C/CACNA1D/GPER1 | 5 |
| hsa04970 | Salivary secretion | 6/135 | 0.004485644 | 0.008294933 | CALM1/ADRB2/ADRA1D/ADRA1A/PRKCB/ADRB3 | 6 |
| hsa04217 | Necroptosis | 8/135 | 0.005009582 | 0.009017405 | PYGM/BCL2/STAT1/IFNG/IL1A/PARP1/GLUL/HSP90AA1 | 8 |
| hsa00380 | Tryptophan metabolism | 4/135 | 0.005074024 | 0.009017405 | MAOB/CYP1A1/CYP1B1/MAOA | 4 |
| hsa05332 | Graft-versus-host disease | 4/135 | 0.005074024 | 0.009017405 | IL6/IL2/IFNG/IL1A | 4 |
| hsa05206 | MicroRNAs in cancer | 12/135 | 0.005502339 | 0.009653226 | MET/BCL2/CYP1B1/CDKN1A/PLAU/MMP9/ERBB2/PRKCB/E2F1/RPS6KA5/CDK6/PIM1 | 12 |
| hsa04924 | Renin secretion | 5/135 | 0.005737158 | 0.00993482 | CALM1/ADRB2/ADRB3/CACNA1C/CACNA1D | 5 |
| hsa05020 | Prion disease | 11/135 | 0.005808049 | 0.00993482 | PSMD3/IL6/CAV1/IL1A/CACNA1C/CACNA1D/HSPA2/GRIN2A/GRIN2D/GRIN3B/COX7A1 | 11 |
| hsa04061 | Viral protein interaction with cytokine and cytokine receptor | 6/135 | 0.006382947 | 0.010783407 | IL6/CCL2/CXCL8/IL2/CXCL11/CXCL10 | 6 |
| hsa05225 | Hepatocellular carcinoma | 8/135 | 0.00693912 | 0.011449992 | MET/GSTM1/GSTM2/CDKN1A/PRKCB/E2F1/IGF2/CDK6 | 8 |
| hsa05146 | Amoebiasis | 6/135 | 0.007018549 | 0.011449992 | IL6/CXCL8/PRKCB/COL1A1/IFNG/COL3A1 | 6 |
| hsa04930 | Type II diabetes mellitus | 4/135 | 0.007028534 | 0.011449992 | SLC2A4/HK2/CACNA1C/CACNA1D | 4 |
| hsa05214 | Glioma | 5/135 | 0.008140302 | 0.013105129 | CALM1/CDKN1A/PRKCB/E2F1/CDK6 | 5 |
| hsa04928 | Parathyroid hormone synthesis, secretion and action | 6/135 | 0.00842556 | 0.013406644 | BCL2/FOS/CDKN1A/PRKCB/RXRG/CYP27B1 | 6 |
| hsa05212 | Pancreatic cancer | 5/135 | 0.008599528 | 0.013526179 | STAT1/CDKN1A/ERBB2/E2F1/CDK6 | 5 |
| hsa05017 | Spinocerebellar ataxia | 7/135 | 0.009828609 | 0.015283722 | GRIA2/PSMD3/PRKCB/ATP2A1/GRIN2A/GRIN2D/GRIN3B | 7 |
| hsa05166 | Human T-cell leukemia virus 1 infection | 9/135 | 0.011790138 | 0.018127948 | CHEK1/CCNA2/JUN/FOS/CDKN1A/IL6/IL2/CHEK2/E2F1 | 9 |
| hsa00591 | Linoleic acid metabolism | 3/135 | 0.012082815 | 0.018371531 | CYP3A4/CYP2C19/PLA2G1B | 3 |
| hsa05022 | Pathways of neurodegeneration - multiple diseases | 15/135 | 0.012729038 | 0.019023611 | GRIA2/CALM1/SLC6A3/BCL2/PSMD3/IL6/PRKCB/IL1A/ATP2A1/NOX4/CACNA1C/CACNA1D/GRIN2A/GRIN2D/COX7A1 | 15 |
| hsa05163 | Human cytomegalovirus infection | 9/135 | 0.01278972 | 0.019023611 | CALM1/CDKN1A/IL6/CCL2/PTGER3/CXCL8/PRKCB/E2F1/CDK6 | 9 |
| hsa04610 | Complement and coagulation cascades | 5/135 | 0.013564355 | 0.01995887 | F2/PLAU/THBD/SERPINE1/F2R | 5 |
| hsa00240 | Pyrimidine metabolism | 4/135 | 0.013926731 | 0.020274077 | TYMS/TYMP/CDA/TK1 | 4 |
| hsa05210 | Colorectal cancer | 5/135 | 0.014213929 | 0.020474357 | JUN/BCL2/FOS/CDKN1A/BIRC5 | 5 |
| hsa04935 | Growth hormone synthesis, secretion and action | 6/135 | 0.014394209 | 0.02051806 | STAT1/FOS/PRKCB/IGFBP3/CACNA1C/CACNA1D | 6 |
| hsa04923 | Regulation of lipolysis in adipocytes | 4/135 | 0.014788717 | 0.020783422 | PTGS1/ADRB2/PTGER3/ADRB3 | 4 |
| hsa04260 | Cardiac muscle contraction | 5/135 | 0.014884128 | 0.020783422 | ATP2A1/CACNA1C/CACNA1D/CACNB2/COX7A1 | 5 |
| hsa05160 | Hepatitis C | 7/135 | 0.015831053 | 0.02188237 | STAT1/CDKN1A/IFNG/CLDN4/CXCL10/E2F1/CDK6 | 7 |
| hsa05410 | Hypertrophic cardiomyopathy | 5/135 | 0.017021109 | 0.023292043 | IL6/ATP2A1/CACNA1C/CACNA1D/CACNB2 | 5 |
| hsa04630 | JAK-STAT signaling pathway | 7/135 | 0.018499555 | 0.024649645 | BCL2/STAT1/CDKN1A/IL6/IL2/IFNG/PIM1 | 7 |
| hsa04658 | Th1 and Th2 cell differentiation | 5/135 | 0.018553598 | 0.024649645 | JUN/STAT1/FOS/IL2/IFNG | 5 |
| hsa05222 | Small cell lung cancer | 5/135 | 0.018553598 | 0.024649645 | BCL2/CDKN1A/E2F1/CDK6/RXRG | 5 |
| hsa04510 | Focal adhesion | 8/135 | 0.019022344 | 0.0250294 | MET/JUN/BCL2/ERBB2/CAV1/PRKCB/COL1A1/SPP1 | 8 |
| hsa04912 | GnRH signaling pathway | 5/135 | 0.019352913 | 0.025221842 | CALM1/JUN/PRKCB/CACNA1C/CACNA1D | 5 |
| hsa05415 | Diabetic cardiomyopathy | 8/135 | 0.020061039 | 0.025898064 | SLC2A4/MMP9/PRKCB/COL1A1/PARP1/COL3A1/ATP2A1/COX7A1 | 8 |
| hsa04926 | Relaxin signaling pathway | 6/135 | 0.020640285 | 0.026396823 | JUN/MMP1/FOS/MMP9/COL1A1/COL3A1 | 6 |

# Supplementary 15

**The results of drug and disease molecular docking**

**Supplementary table 15. The results of drug and disease molecular docking.**

| Ingredient | Molecular Formula | Molecular Weight | Crystal structure | Target | Affinity energy/(kj·mol^-1^) |
| --- | --- | --- | --- | --- | --- |
| quercetin | C_15_H_10_O_7_ | 302.23g/mol | 3ifd | CCL2 | -6.1 |
| quercetin | C_15_H_10_O_7_ | 302.23g/mol | 4xdx | CXCL8 | -7.5 |
| isoastragaloside ii | C_43_H_70_O_15_ | 827.00g/mol | 2yk9 | HSP90AA1 | -8.0 |
| Pinocembrin | C_15_H_12_O_4_ | 256.25g/mol |  |  | -8.0 |
| sucrose | C_12_H_22_O_11_ | 342.3g/mol |  |  | -5.9 |
| quercetin | C_15_H_10_O_7_ | 302.23g/mol | 3bes | IFNG | -7.4 |
| quercetin | C_15_H_10_O_7_ | 302.23g/mol | 1alu | IL6 | -7.0 |
| procurcumenol | C_15_H_22_O_2_ | 234.33g/mol |  |  | -6.5 |
| kaempferol | C_15_H_10_O_6_ | 286.24g/mol | 6y3v | JUN | -6.3 |
| quercetin | C_15_H_10_O_7_ | 302.23g/mol |  |  | -6.4 |
| quercetin | C_15_H_10_O_7_ | 302.23g/mol | 6esm | MMP9 | -10.7 |
| kaempferol | C_15_H_10_O_6_ | 286.24g/mol | 1bf5 | STAT1 | -9.8 |
| quercetin | C_15_H_10_O_7_ | 302.23g/mol |  |  | -9.4 |

# Supplementary 16

**The diagram of core signaling pathway**

**Figure 1. hsa04933:AGE-RAGE signaling pathway in diabetic complications.**

**
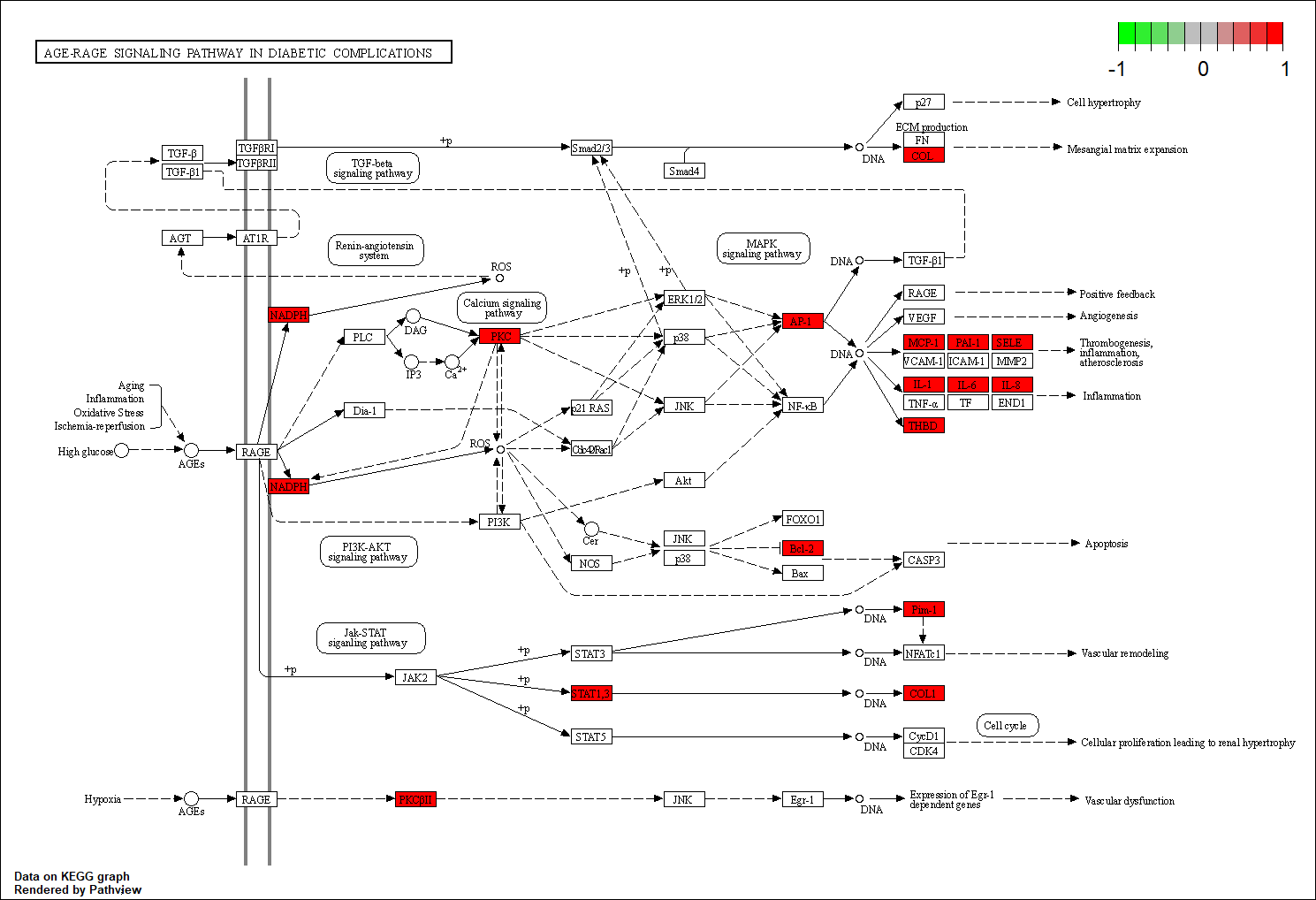
**

**Figure 2. hsa04020:Calcium signaling pathway.**

**
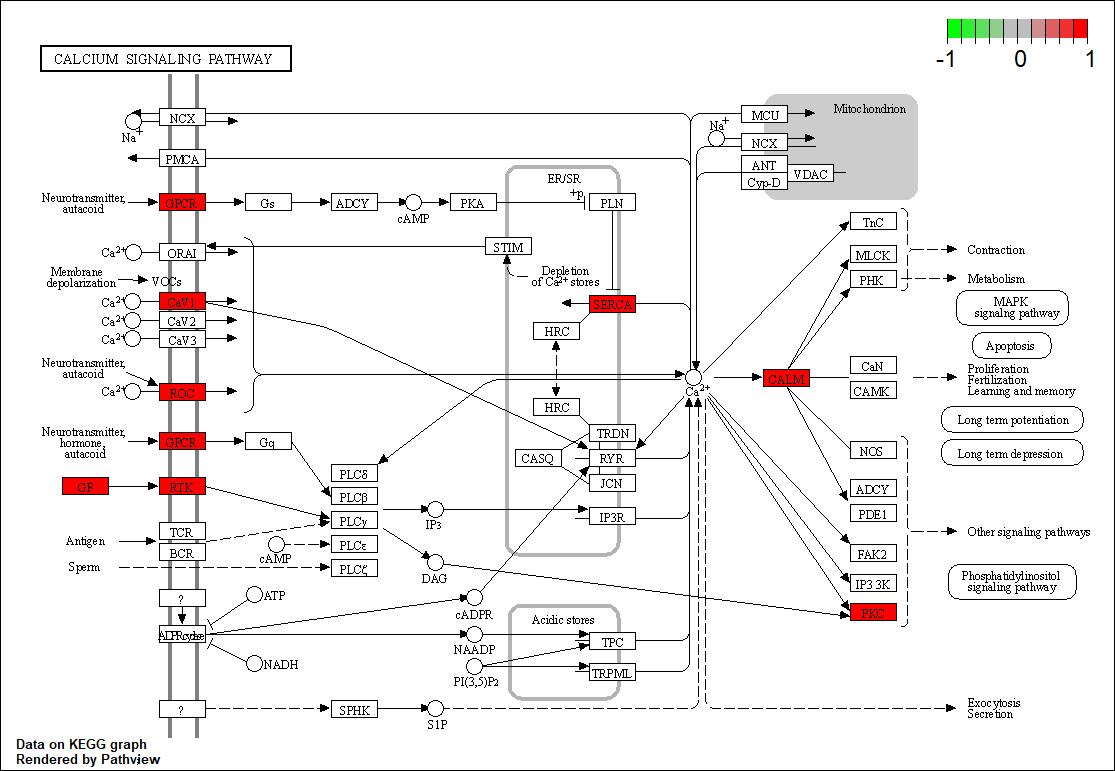
**

**Figure 3. hsa04151:PI3K-Akt signaling pathway.**

**
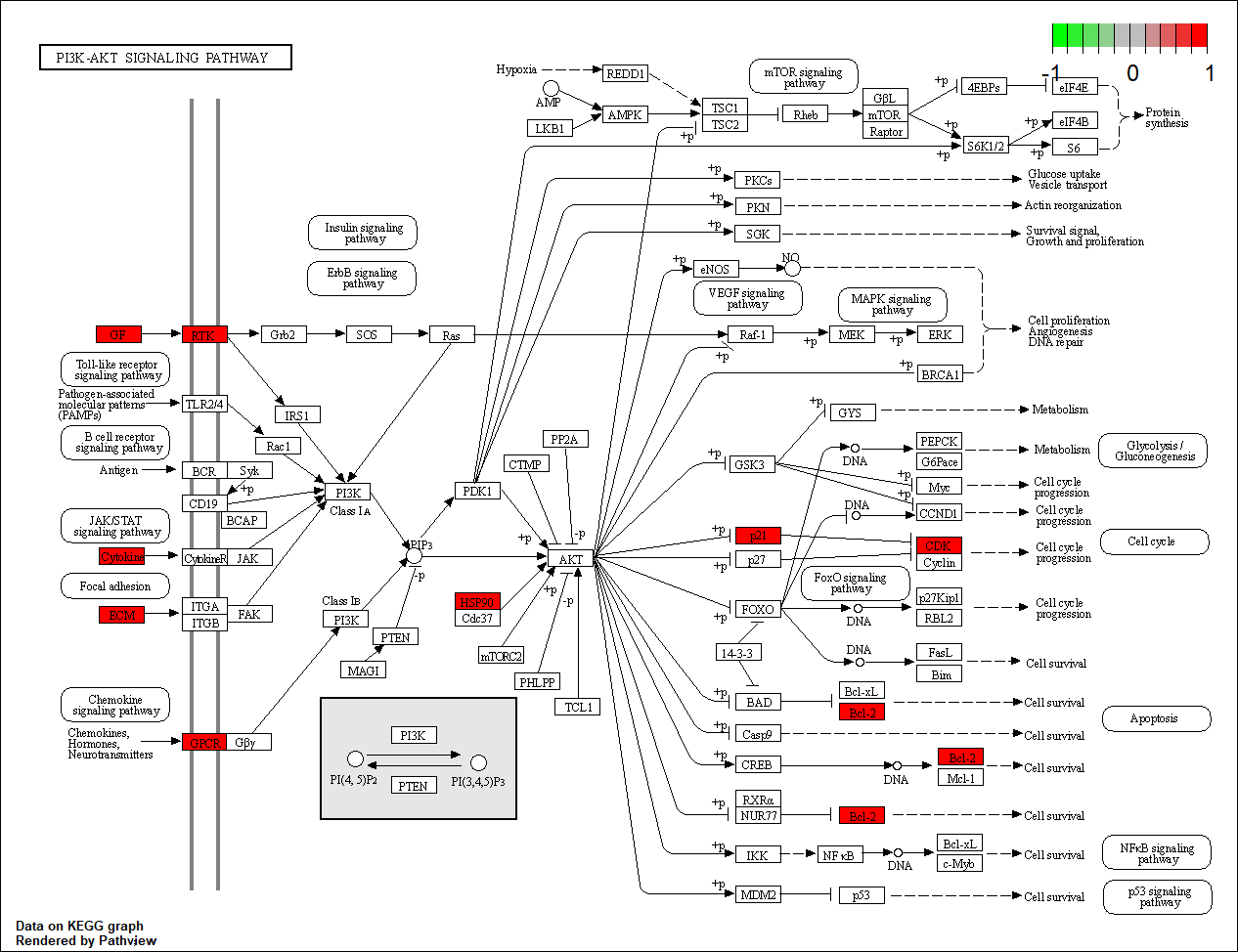
**

**Figure 4. hsa04010:MAPK signaling pathway.**

**
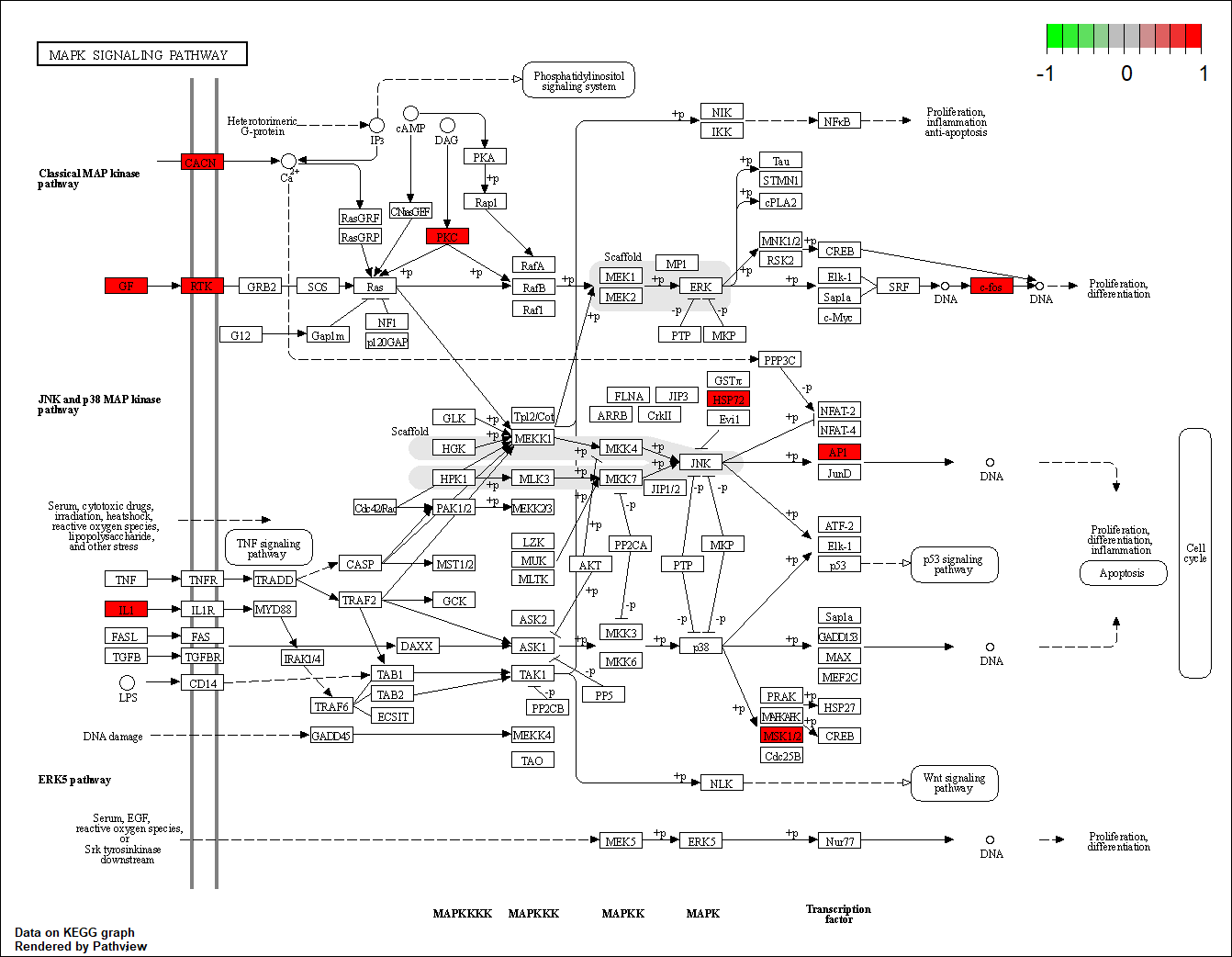
**

**Figure 5. hsa04657:IL-17 signaling pathway.**

**
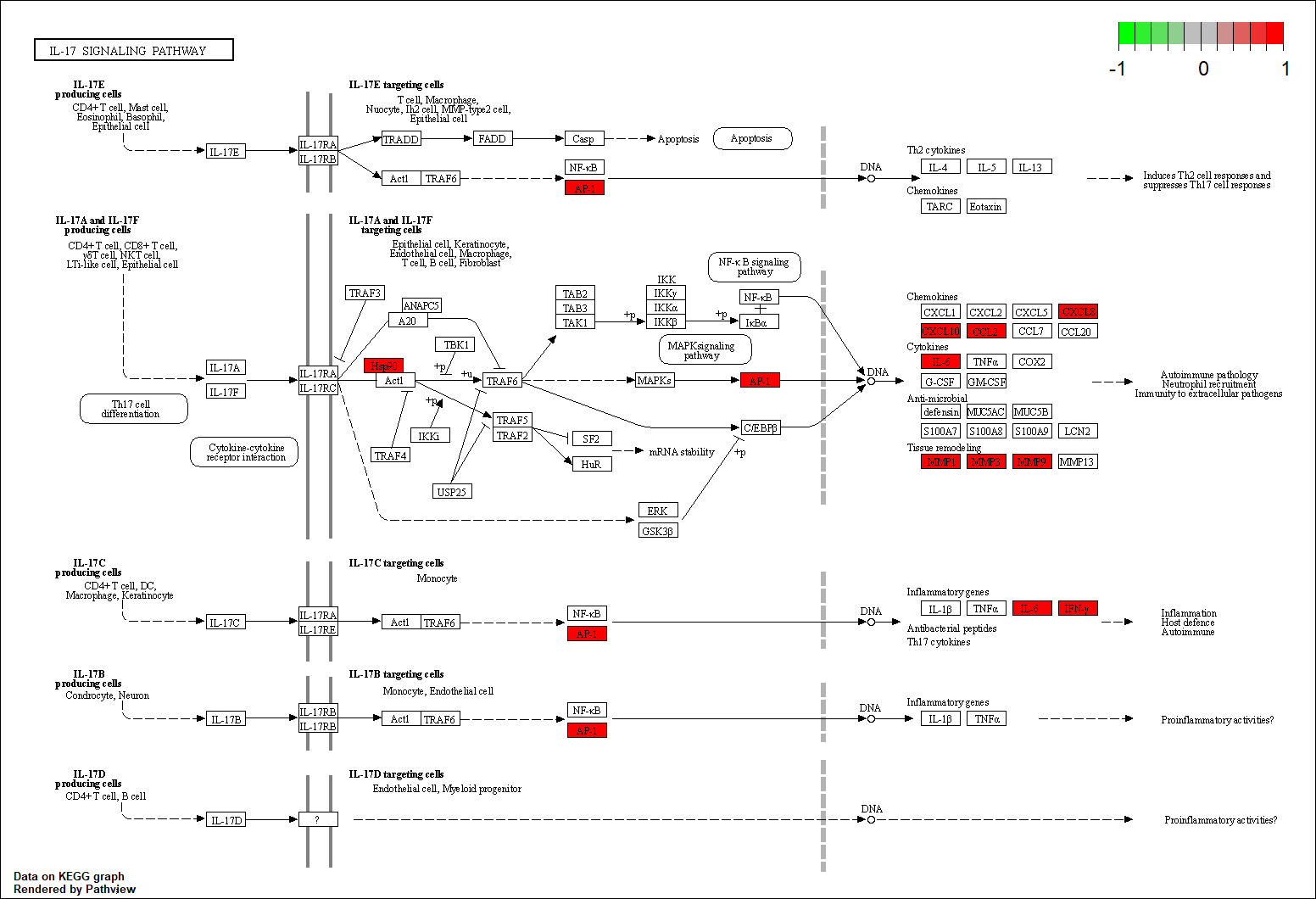
**

**Figure 6. hsa04115:p53 signaling pathway.**

**
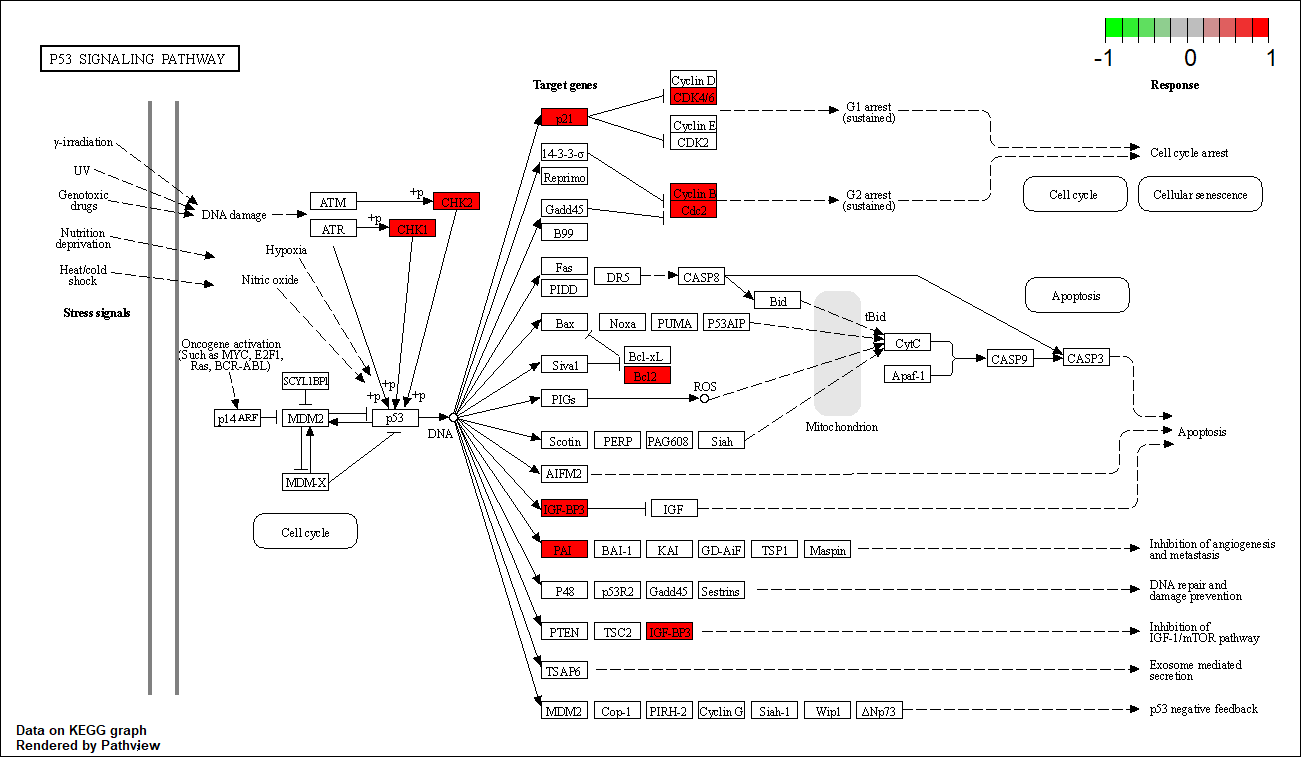
**

**Figure 7. hsa04668:TNF signaling pathway.**

**
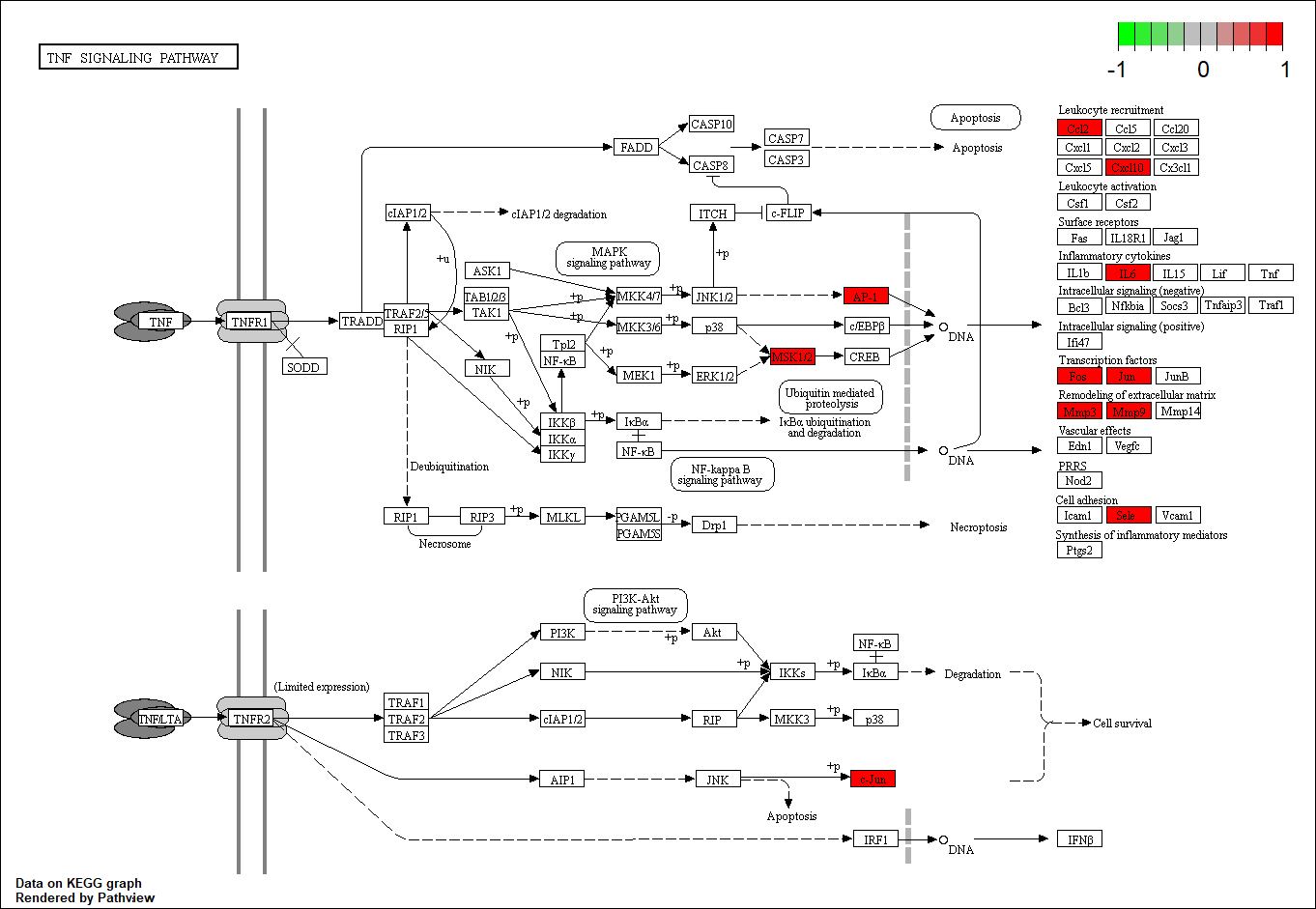
**

**Figure 8. hsa04620:Toll-like receptor signaling pathway.**

**
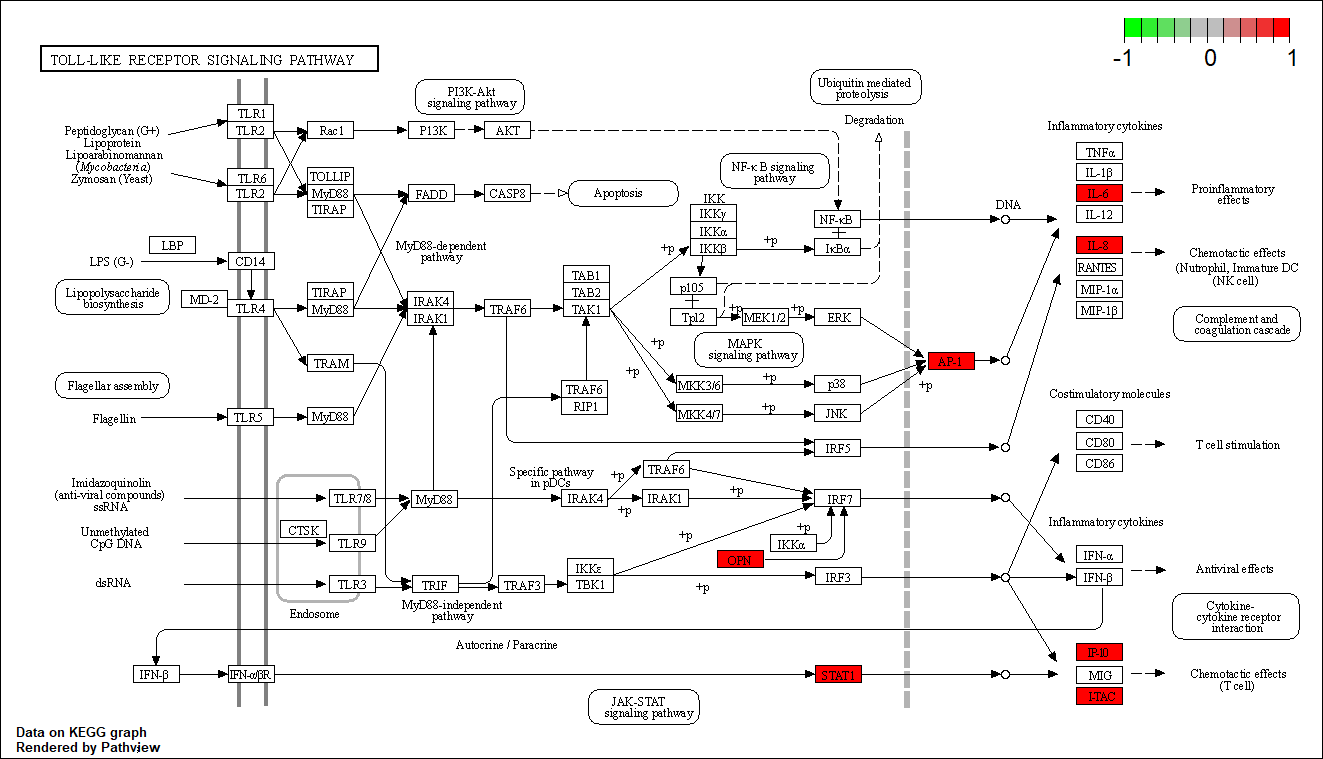
**

**Figure 9. hsa04066:HIF-1 signaling pathway.**

**
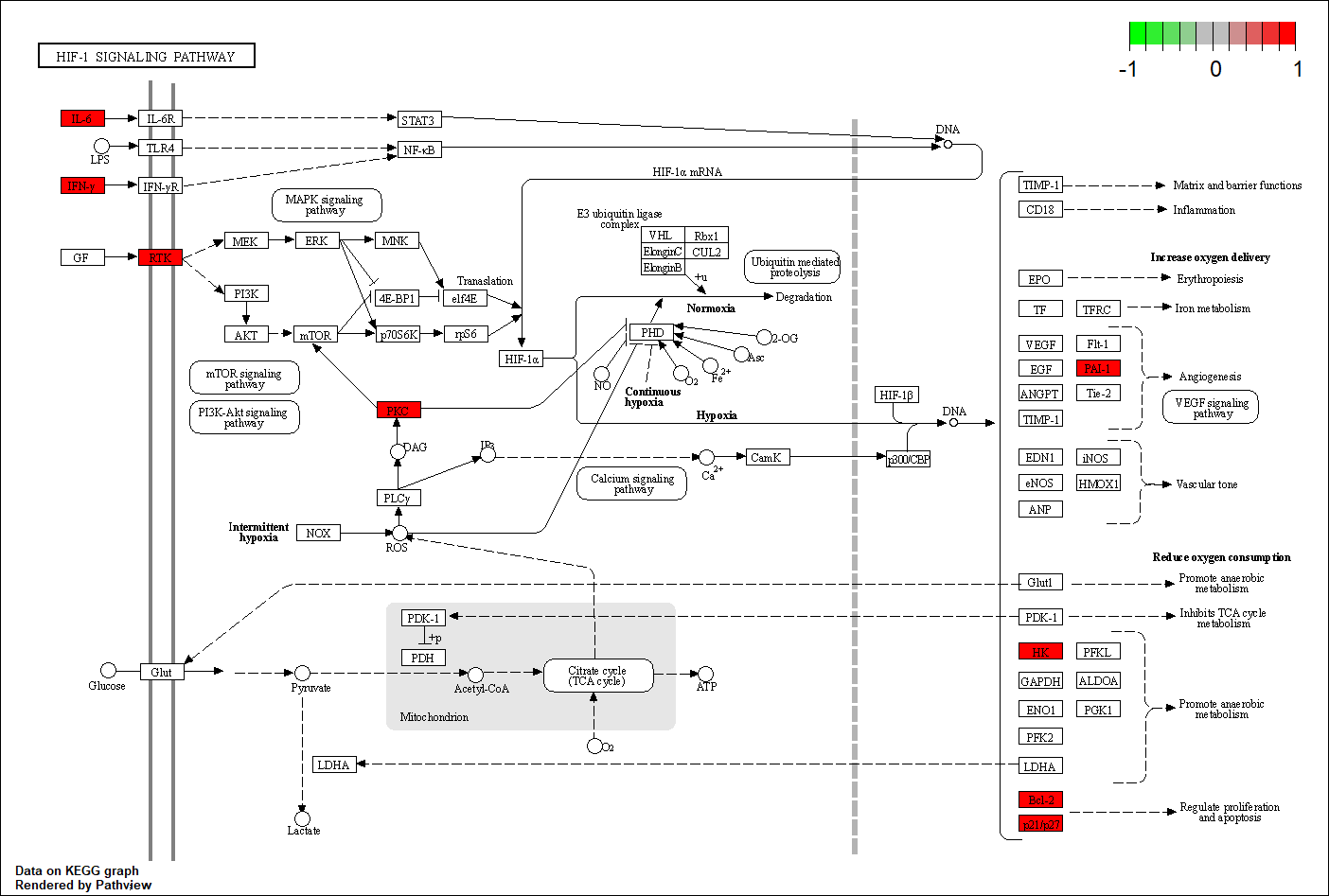
**
